# Structure-based design of nucleoside-derived analogues as sulfotransferase inhibitors†

**DOI:** 10.1039/c9ra07567d

**Published:** 2019-10-09

**Authors:** Neil M. Kershaw, Dominic P. Byrne, Hollie Parsons, Neil G. Berry, David G. Fernig, Patrick A. Eyers, Richard Cosstick

**Affiliations:** Department of Chemistry, University of Liverpool Liverpool L69 7ZD UK nkershaw@liverpool.ac.uk; Department of Biochemistry, Institute of Integrative Biology, University of Liverpool Liverpool L69 7ZB UK

## Abstract

Sulfotransferases (STs) catalyse the transfer of a sulfonyl group (‘sulfation’) from the enzyme co-factor 3′-phosphoadenosine 5′-phosphosulfate (PAPS) to a variety of biomolecules. Tyrosine sulfation of proteins and carbohydrate sulfation play a crucial role in many protein–protein interactions and cell signalling pathways in the extracellular matrix. This is catalysed by several membrane-bound STs, including tyrosylprotein sulfotransferase 1 (TPST1) and heparan sulfate 2-*O*-sulfotransferase (HS2ST1). Recently, involvement of these enzymes and their post-translational modifications in a growing number of disease areas has been reported, including inflammation, cancer and Alzheimer's disease. Despite their growing importance, the development of small molecules to probe the biological effect of TPST and carbohydrate ST inhibition remains in its infancy. We have used a structure-based approach and molecular docking to design a library of adenosine 3′,5′-diphosphate (PAP) and PAPS mimetics based upon 2′-deoxyadenosine and using 2′-deoxy-PAP as a benchmark. The use of allyl groups as masked methyl esters was exploited in the synthesis of PAP-mimetics, and click chemistry was employed for the divergent synthesis of a series of PAPS-mimetics. A suite of *in vitro* assays employing TPST1 and HS2ST, and a kinase counter screen, were used to evaluate inhibitory parameters and relative specificity for the STs.

## Abstract 2

Regulated sulfation of biomolecules by sulfotransferases (STs) plays a role in many biological processes with implications for a number of disease areas. A structure-based approach and molecular docking were used to design a library of ST inhibitors.

## Graphical abstract

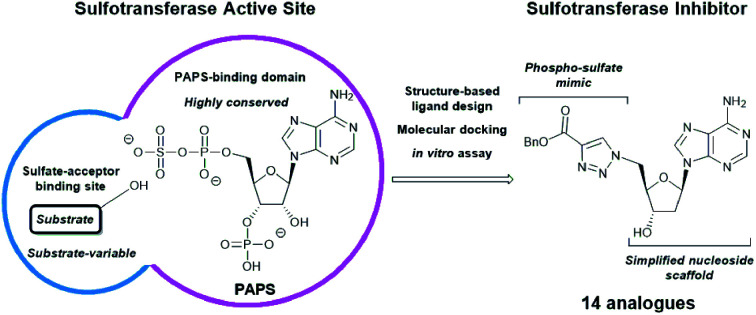

## Introduction

The post-translational covalent modification of biomolecules through the addition of a sulfonyl group (SO_3_^−^) to a hydroxyl or amino acceptor moiety is of importance in a very broad range of biological processes.^1,2^ The ubiquitous donor of activated sulfate is 3′-phosphoadenosine 5′-phosphosulfate (PAPS). Sulfation (also termed sulfonation) is catalysed by enzymes called sulfotransferases (STs) which are separated into two general classes. Cytosolic STs (SULTs) sulfate both small endogenous and exogenous compounds, including hormones, neurotransmitters, and a variety of pharmaceutical agents, and have critical roles in detoxification and excretion.^3,4^ In contrast, the membrane-associated STs are located in the Golgi apparatus and sulfate endogenous secreted macromolecules such as proteins and polysaccharides, including glycosaminoglycans (GAGs).^5,6^ The STs possess a structurally-conserved PAPS-binding domain, but exhibit distinct substrate binding sites, and in contrast with cytosolic STs, the membrane-associated STs exhibit a higher degree of substrate selectivity.^3,7^

**Fig. 1:**
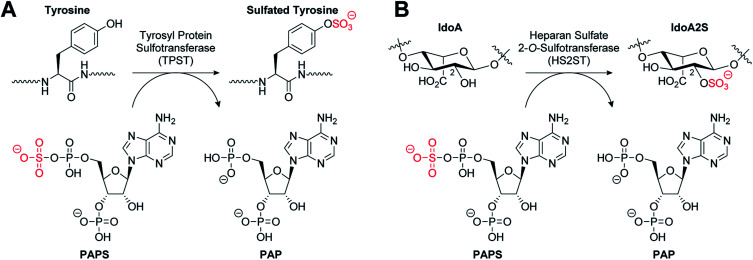
(A) Tyrosylprotein sulfotransferase catalysed sulfation of a tyrosine residue. (B) Heparan sulfate 2-*O*-sulfotransferase catalysed sulfation of an iduronic acid (IdoA) subunit of heparan sulfate.

Tyrosine sulfation is a relatively common post-translational modification of proteins catalysed by tyrosylprotein sulfotransferases 1 and 2 (TPST1 and 2) (Fig. 1A). Tyrosine sulfation was first reported in bovine fibrinogen in 1954.^8^ It has since been shown to play an important role in a wide range of biological processes.^9^ For example, it is implicated in the modulation of extracellular protein–protein interactions such as those involved in leukocyte adhesion^10–12^ and hemostasis.^13–15^ It also plays a role in visual function,^16^ agonist binding to hormone receptors^17^ and the proteolytic processing of bioactive peptides.^18^ Sulfation of several tyrosine residues in the N-terminal domain of the chemokine receptor CCR5 has been shown to be crucial in mediating HIV entry into cells.^19,20^ In addition, tyrosine sulfation of the Duffy antigen/receptor for chemokines (DARC) is important for the malaria parasite *Plasmodium vivax* binding and entry into erythrocytes.^21^

Heparan sulfates (HS) are highly sulfated GAGs that can exist either conjugated to proteins or as free, often extended, chain-forming fragments.^22^ HS has been shown to bind to extracellular growth factors, cytokines, enzymes and cell matrix proteins, and also to act as co-receptors to generate signalling-competent growth factor complexes.^23,24^ As a component of the extracellular matrix, HS has roles in important processes such as cell adhesion,^25^ viral invasion,^26^ and Alzheimer's disease.^27^ HS 2-*O*-sulfotransferase (HS2ST1) transfers a sulfonyl group to the 2-OH of iduronic acid (IdoA) and more rarely to glucuronic acid (GlcA) to give IdoA2S and GlcA2S, respectively (Fig. 1B).^28^ HS2ST-knockout mouse models have revealed a crucial role for 2-*O*-sulfated HS in kidney and eye development, and for nervous system function.^29^ Additionally, 2-*O*-sulfation of HS is essential for regulation of fibroblast growth factor (FGF)-2 signalling,^30^ and has been shown to play a role in prostate cancer cell proliferation *in vitro*.^31^

Given the importance that regulated sulfation plays in a plethora of biological processes, it is somewhat surprising that modulation of the TPSTs and carbohydrate STs using small molecules remains relatively understudied.^32–36^ In contrast, the SULTs have received significant attention, mainly focussed on estrogen (steroid) sulfotransferase (SULT1E) through modulation of the substrate binding site. Many human tumours are hormone-dependent, playing a central role in their development and survival. Estrogen sulfatase inhibition therefore represents an attractive therapeutic option in oncology. A range of inhibitors have been discovered based upon natural products,^37^ polychlorinated phenols,^38^ and compounds designed to be bi-substrate mimics.^39,40^ More recently, irreversible clinical steroid sulfatase inhibitors such as the orally active non-steroidal aryl sulfamate irosustat (STX64) have entered clinical trials.^41^ Related to sulfation, the reversible process of protein phosphorylation, which was also originally described in the 1950s, is catalysed by >500 diverse members of the human protein kinase superfamily.^42^ In contrast to sulfation, the kinase field is approaching maturity in the biomedical context, with over 50 kinase inhibitors approved in cancer and inflammatory disease.^43–47^ Of interest, we recently described facile real-time biochemical assays, which were previously developed for analysing phosphorylation in the kinase field, to evaluate tyrosine and carbohydrate sulfation *in vitro*. Using this high-throughput enzymatic approach, we discovered that HS2ST and the TPSTs were novel targets for several families of previously known protein kinase inhibitor, suggesting that the ATP and PAPS binding site of these classes of enzyme might share similar druggable features.^48,49^

In this report, we describe a structure-based approach to ST inhibitor design, based on our understanding of co-factor and substrate binding. Targeting the substrate binding site of the SULTS has previously been exploited in the design of small molecule inhibitors.^37–40^ However, this strategy would be significantly more challenging for the membrane-associated STs as the binding interactions are protein–protein/protein–polysaccharide in nature. We opted to target the PAPS binding site with the intention of developing a general ST inhibitor initially. Subsequent exploitation of differences in the substrate binding sites could then conceivably be used to develop selectivity once general inhibition had been achieved. It is noteworthy that many protein kinase inhibitors exert their action through competitive inhibition of the ATP (analogous to PAPS) binding site. We utilised the published crystal structures of TPST1 complexed to adenosine 3′-5′-diphosphate (PAP) and substrate peptides^50^ and HS2ST1 bound to PAP and a heptasaccharide.^51^ Analysis of the PAP-binding site and subsequent molecular docking were utilised to guide the design of a library of PAP and PAPS mimetics based upon 2′-deoxyadenosine. Compounds were subsequently assessed for inhibitory activity towards purified HS2ST1 and TPST1 in the presence of the sulfate donor PAPS using our microfluidic-shift assays. Finally, a counter screen against the canonical ATP-dependent Ser/Thr kinase protein kinase A (PKA) was performed to gauge relative specificity for ST inhibition.

## Results and discussion

### Crystallographic analysis, compound selection and molecular docking

The crystal structures of TPST1 and HS2ST (PDB IDs: 5WRI^50^ and 4NDZ^51^) bound to PAP and sulfate acceptor substrates were used to facilitate the rational design of potential enzyme inhibitors. Central to the nucleoside-binding domain of the STs are highly-conserved structural features termed the 5′-phosphosulfate-binding (5′-PSB) and 3′-phosphate-binding (3′-PB) loops (Fig. SI 1A and B†). These make a network of hydrogen bonds involving the protein surface and the 3′- and 5′-phosphates of PAP. The adenine ring is involved in a hydrophobic or π-stacking interaction and a hydrogen bond to N-6 is frequently observed.

**Fig. 2:**
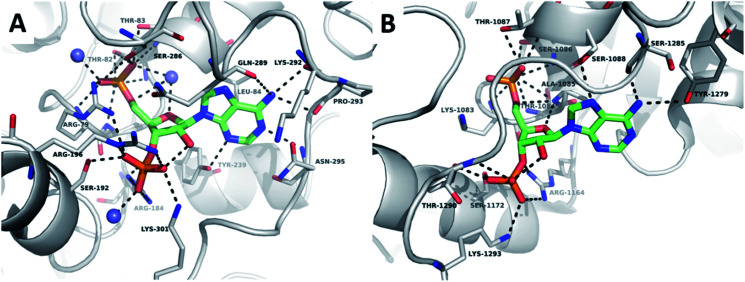
Crystallographic analysis of the sulfotransferase active site. (A) Nucleoside-binding domain of TPST1 complexed with PAP (PDB ID: 5WRI). TPST1 is rendered as grey cartoon. Residues interacting with PAP are labelled and rendered as thin sticks (carbon – grey, nitrogen – blue, oxygen – red). Crystallographic waters are rendered as slate spheres PAP is rendered as coloured sticks (carbon – green, nitrogen – blue, oxygen – red). Black dotted lines indicate hydrogen bonds. (B) Nucleoside-binding domain of HS2ST complexed with PAP (PDB ID: 4NDZ). HS2ST is rendered as grey cartoon. Residues interacting with PAP are labelled and rendered as thin sticks (carbon – grey, nitrogen – blue, oxygen – red). PAP is rendered as coloured sticks (carbon – green, nitrogen – blue, oxygen – red). Black dotted lines indicate hydrogen bonds.

Comparison of the structures of TPST1 and HS2ST bound to PAP reveals very similar hydrogen bonding interactions between the 3′-phosphate of PAP and the sidechains of a number of basic residues (Fig. 2A and B). The 5′-phosphate of PAP also forms a number of analogous interactions in both structures. However, in TPST1 a water molecule bridges the 4′- and 5′-oxygens, whereas in HS2ST a backbone interaction with Ala-1085 performs this function. The hydrophobic interaction with adenine is made by Leu-84 in TPST1 and Ala-1085 in HS2ST. N-6 makes a number of contacts in both structures. In TPST1, N-1 makes a backbone interaction with Asn-295 and N-3 makes a sidechain interactions with Tyr-239. In contrast, N-1 and N-3 do not make any contacts in HS2ST but N-7 accepts a hydrogen bond from the sidechain of Ser-1088. The 2′-hydroxyl of PAP forms a hydrogen with the 3′-phosphate but does not make any contacts to the protein in either structure.

**Fig. 3:**
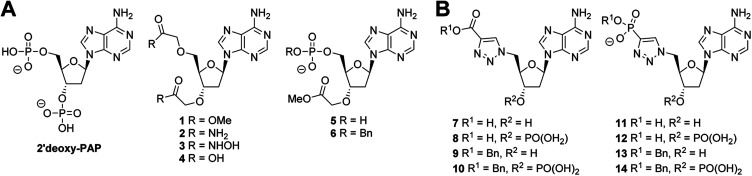
Summary of chemical structures. (A) PAP-mimetics (B) PAPS-mimetics.

Given that the 2′-hydroxyl does not appear to directly contribute to binding we reasoned that it would be feasible to remove it in our subsequent inhibitor design and thus focus on derivatives of 2′-deoxyadenosine. Although 2′-deoxyribonucleosides are known to preferentially adopt a different sugar pucker conformation compared with ribose nucleosides, the inversion barrier is small (*ca.* 20 kJ mol^−1^) and the conformers are in rapid equilibrium in solution.^52^ 2′-Deoxyadenosine has also previously served as a starting point for SULT inhibitor design.^40^ This strategy would have the additional benefit of reducing the need for the lengthy protecting group strategies required for selective modification of ribonucleosides. 2′-Deoxy-PAP represents the closest analogue to PAP based on this scaffold and would serve as a benchmark from which to compare our library of compounds (Fig. 3A). Surprisingly, this compound has not previously been assessed as a ST inhibitor.

A series of PAP-mimetics probing the binding requirements at the 3′- and 5′-positions were proposed, with isosteric replacement of the phosphates for less metabolically labile substituents. Four functional groups were selected, with differing p*K*_a_ values such that a range of ionisation states at a physiologically relevant pH could be assessed (1–4) (Fig. 3A). Bis-methyl ester 1 and bis-amide 2 would be non-ionised, whereas bis-hydroxamic acid 3 and bis-carboxylic acid 4 would be half- and fully-ionised, respectively. Compounds 5 and 6 were selected as a result of an unanticipated differentiation of the 3′- and 5′-hydroxyls during the synthesis of 1–4, *vide infra*.

Analogues of PAPS containing a hydrolytically stable isostere of the 5′-phosphosulfate were also considered. Compounds 7–14 were selected (Fig. 3B) bearing a triazole as a phosphate bioisostere,^53,54^ and either a carboxylate or phosphate terminus to mimic the sulfate of PAPS. The presence or absence of the 3′-phosphate would also be assessed (*e.g.*7*vs.*8). Compounds bearing a benzyl group on the 5′-carboxylate or phosphate terminus (6, 9–10, 13–14), were selected to investigate the possibility of occupying both the nucleoside and substrate binding sites. Whilst it was recognised that PAP and PAPS mimetics would not achieve selective inhibition of one ST over another, it could be anticipated that selectivity could be subsequently designed into a potent, general ST inhibitor.

A molecular docking study was used to predict the mode and relative strength of compound binding. A protocol was first developed that reproduced the binding pose of PAP with TPST1 and HS2ST accurately (see ESI for details,† RMSD, 0.04 Å and 0.13 Å respectively). This protocol was then employed to dock PAPS, 2′-deoxy-PAP and 1–14. Phosphates were docked in their monobasic form. Carboxylic acids were docked as carboxylates. All were predicted to bind in an analogous fashion to PAP in both TPST1 and HS2ST, and the docking scores were also very similar (Table SI 1, Fig. SI 2A–Q†). The 5′-triazolecarboxylate/phosphate of 7–14 overlaid well with the predicted binding pose for PAPS (Fig. SI 3†). The benzyl groups of 6, 9–10, 13–14 were predicted to span the region between the nucleoside and substrate binding sites (Fig. SI 4†). This provided confidence in the synthesis of 2′-deoxy-PAP and compounds 1–14.

### Synthesis

**Scheme 1:**
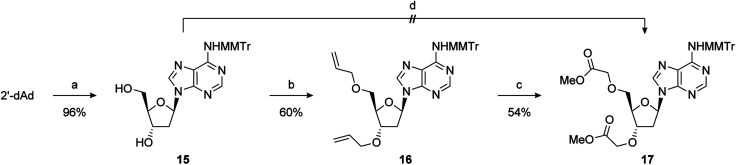
Reagents and conditions: (a) TMSCl, pyridine, MMTrCl, 0 °C to RT then aq. NH_3_; (b) NaHMDS, allyl bromide, TBAI, DMF, −20 °C to RT; (c) O_3_, NaOH, MeOH, DCM, −78 °C, then AcOH; (d) NaHMDS, methyl bromoacetate, TBAI, DMF, −20 °C to RT.

2′-Deoxy-PAP was synthesised using a modified literature procedure (see ESI for details†).^55^ It was envisaged that a suitably protected derivative of bis-methyl ester 1 would act as a point of late-stage diversification to access 1–4. The synthesis of this key intermediate (7) is summarised in Scheme 1. Monomethoxytrityl (MMTr) protection of 2′-deoxyadenosine using the transient protection protocol occurred in high yield to give 15.^56,57^ It had been expected that the required methylene methyl ester functionality at the 3′- and 5′-positions could be introduced though bis-alkylation of 15 using methyl bromoacetate.^58^ Unfortunately, under a range of conditions, only decomposition of the starting material was observed. The reason for decomposition is not clear, as the reaction was successful using *t*-butyl bromoacetate.^59^ However, due to the relatively harsh conditions required to remove the *t*-butyl groups, it did not prove possible to advance this material through to 17. An alternative strategy was devised using allyl groups as latent methyl esters.^60^ Double allylation of 15 proceeded smoothly, and was followed by a modified ozonolysis to provide key intermediate 17 in a 54% yield (31% overall from 2′-deoxyadenosine).^61–63^

**Scheme 2:**
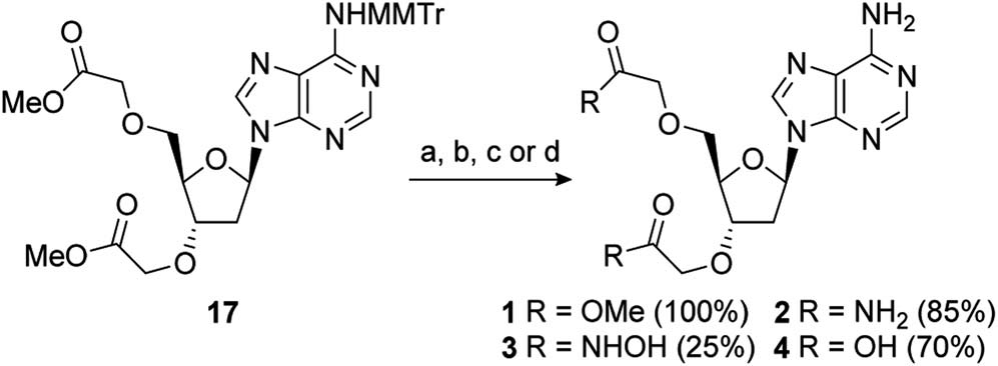
Reagents and conditions: (a) 80% aq. AcOH, RT; (b) (i) 6 M NH_3_, MeOH, RT; (ii) 80% aq. AcOH, RT; (c) (i) 50% wt. aq. NH_2_OH, MeOH, RT; (ii) 80% aq. AcOH, RT; (d) (i) 1 M aq. NaOH, MeOH, RT; (ii) 80% aq. AcOH, RT.

With 17 in hand, completion of the synthesis of 1–4 was achieved in short order. Accordingly, deprotection of the MMTr group using aqueous AcOH furnished bis-methyl ester 1 in quantitative yield.^64^ Treatment of 17 with 6 M methanolic ammonia followed by MMTr deprotection gave bis-amide 2 in an 85% yield over two steps. Conversion to bis-hydroxamic acid 3 was achieved in a similar fashion, albeit in lower yield. Finally, basic ester hydrolysis of 17 and MMTr deprotection proceeded in high yield to give bis-acid 4 (Scheme 2).

**Scheme 3:**
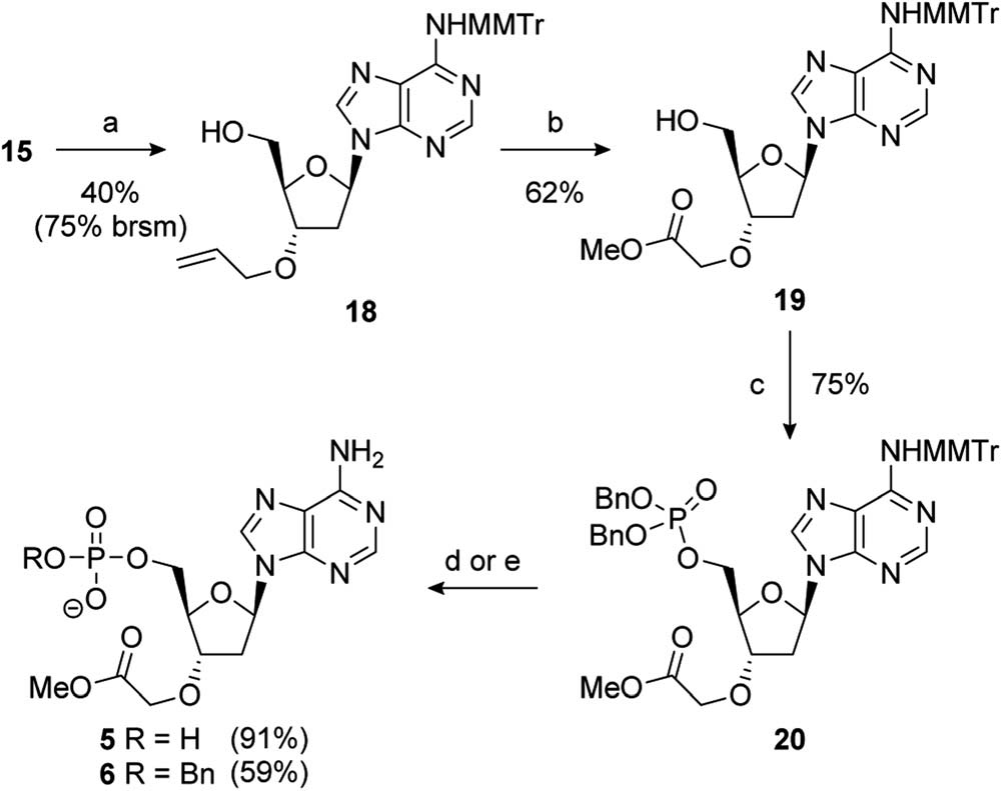
Reagents and conditions: (a) NaHMDS, allyl bromide, TBAI, DMF, −45 °C to RT; (b) O_3_, NaOH, MeOH, DCM, −78 °C; (c) i-Pr_2_NP(OBn)_2_, 1*H*-tetrazole, MeCN, 0 °C to RT then *m*CPBA; (d) (i) 10% Pd/C, H_2_, 1 atm, Et_3_N, MeOH, RT, then Na^+^-Dowex®; (ii) 80% aq. AcOH, RT; (e) (i) 80% aq. AcOH, RT; (ii) NaI, MeCN, 80 °C.

During the double allylation of 15, it was noticed that a small amount of a mono-allylated product was also produced. Acetylation of the purified product confirmed that allylation had occurred at the 3′-position (Fig. SI 5†). No mono-allylation at the 5′-position could be detected. Selective 2′-*O*-alkylation of unprotected ribonucleosides is an established process under a number of conditions.^65,66^ However, protection of the 5′-hydroxyl is normally required for selective modification of the 3′-hydroxyl of 2′-deoxynucleosides.^61^ We speculate that this selectivity is caused by steric hindrance of the 5′-OH by the bulky MMTr group as 15 is resistant to standard 5′-halodeoxygenation conditions whereas the unprotected nucleoside reacts smoothly. Further studies into this novel selectivity is currently underway. Although unexpected, it was recognised that this selectivity could potentially be exploited to differentiate the 3′- and 5′-positions and thus generate a number of non-symmetric PAP mimetics. After significant optimisation, conditions were developed to reliably furnish the 3′-allylated product 18 in a 40% yield (75% based on recovered starting material) (Scheme 3). Performing the addition of allyl bromide above −45 °C improved the conversion of starting material, but at the expense of the isolated yield of 18. This was then advanced to the orthogonally protected nucleoside 20 through a two-step procedure. First, the alkene was oxidatively cleaved, as previously described, and then the 5′-hydroxyl was converted to the dibenzylphosphate using standard phosphoramidite conditions.^67^

Removal of benzyl groups under hydrogenative conditions^68^ followed by MMTr deprotection proceeded smoothly to give 5 in a 91% yield. Alternatively, cleavage of the MMTr group followed by selective removal one of the benzyl groups, by treatment with sodium iodide in acetonitrile at elevated temperature, completed the synthesis of 6 in good overall yield (59%).^69^

**Scheme 4:**
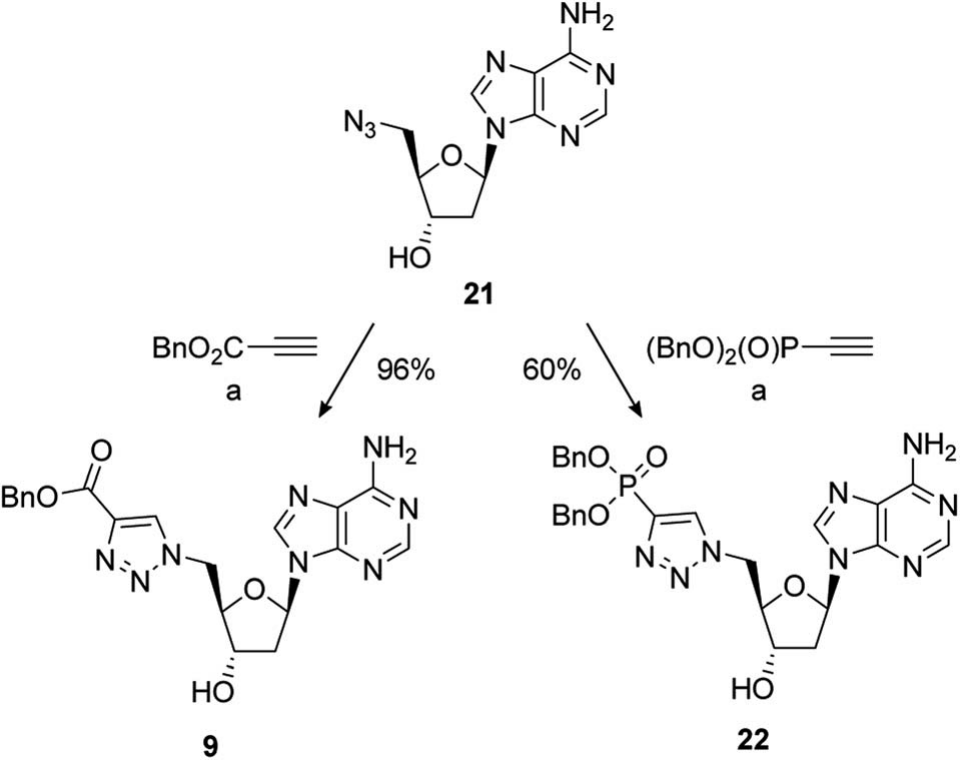
Reagents and conditions: (a) alkyne, 5 mol% CuSO_4_·5H_2_O, 10 mol% sodium ascorbate, *t*-BuOH/H_2_O (1 : 1), RT; removal of the benzyl group of 9 under hydrogenative conditions occurred in excellent yield to provide 7. Conversion of 9 to the 3′-dibenzylphosphate followed by exhaustive hydrogenation provided 8 in a 66% yield over the two steps. An alternative phosphate protecting group was required for the synthesis of 10. Accordingly, formation of the bis-cyanoethyl-protected 3′-phosphate afforded 23 in a 75% yield.^73^ This was followed by selective deprotection using the method of Gaffney and Reese^74^ to provide 10 in a 74% yield (Scheme 5).

The synthesis of PAPS mimetics 7–14 commenced from 2′-deoxy-5′-azidoadenosine (21)^70^*via* a copper-catalysed alkyne acetylene cycloaddition (CuAAC), or “click” coupling, with the appropriate alkyne partner.^71^21 was synthesised in two steps from 2′-deoxyadenosine (See ESI for details†). CuAAC under standard conditions with benzyl propiolate gave triazole 9 in excellent yield. Reaction of 21 with dibenzyl ethynylphosphonate^72^ also proceeded smoothly to provide 22 in good yield (Scheme 4).

**Scheme 5:**
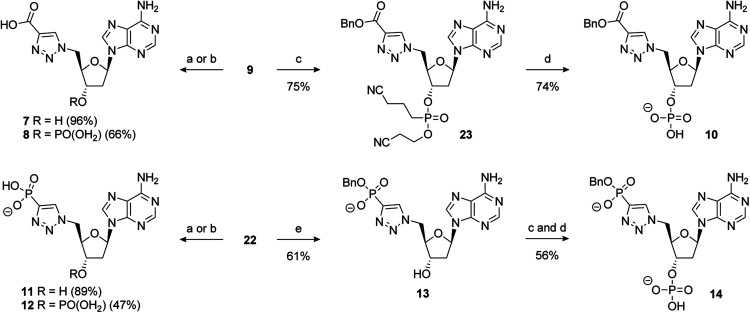
Reagents and conditions: (a) 10% Pd/C, H_2_, 1 atm, Et_3_N, MeOH, RT, then Na^+^-Dowex®; (b) (i) i-Pr_2_NP(OBn)_2_, 1*H*-tetrazole, MeCN, 0 °C to RT then *m*CPBA; (ii) 10% Pd/C, H_2_, 1 atm, Et_3_N, MeOH, RT, then Na^+^-Dowex®; (c) i-Pr_2_NP(OCH_2_CH_2_CN)_2_, 1*H*-tetrazole, MeCN, 0 °C to RT then *m*CPBA; (d) TMG, TMSCl, MeCN, RT, then aq. NH_3_; (e) NaI, MeCN, 80 °C; abbreviations: TMG = 1,1,3,3-tetramethylguanidine.

Hydrogenation of 22 gave 11 in excellent yield (89%). 22 could also be converted to the 3′-dibenzylphosphate which gave 12 upon global deprotection in a 47% yield. Treatment of 22 with sodium iodide in acetonitrile, as previously described, gave 13 in moderate yield. Finally, 3′-phosphorylation of 13 and deprotection gave 14 in a 56% yield over two steps (Scheme 5).

### Determination of ST inhibitory activity and a simple kinase counter-screen.

**Fig. 4:**
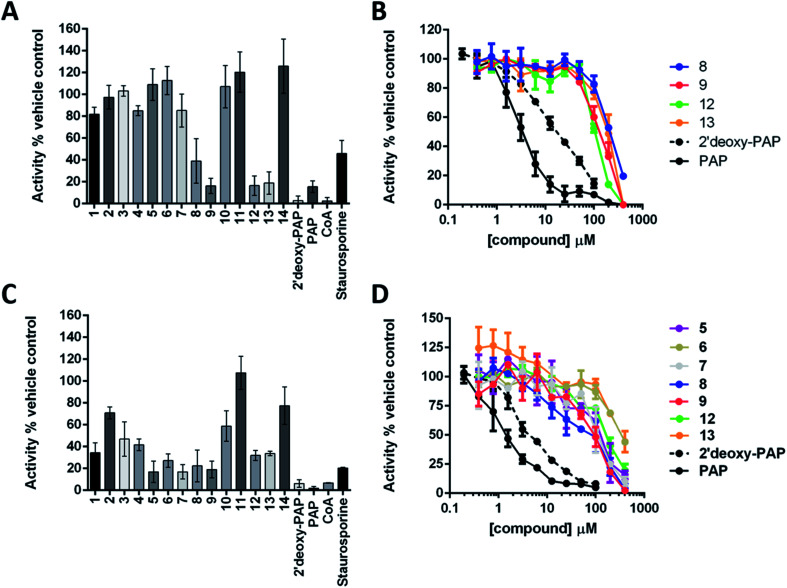
Summary of enzymatic inhibition assays against HS2ST and TPST1. (A) Enzymatic analysis of HS2ST1 inhibition by a panel of PAP- and PAPS-mimetic compounds. MBP-tagged HS2ST (20 nM) was incubated with PAPS (5 μM) in the presence of the appropriate nucleoside analogue (400 μM). HS2ST sulfotransferase activity was assayed using a fluorescent hexasaccharide substrate (2 μM) and normalised to DMSO (4% v/v) or buffer control. Data shown is mean and SD of 4 repeat experiments. (B) Full dose–response curves for selected compounds. HS2ST (20 nM) was incubated with increasing concentration of the indicated compound in the presence of PAPS (5 μM) for 15 min at 20 °C. HS2ST activity calculated as previously described. Data from two independent experiments are combined. (C) Enzymatic analysis of TPST1 inhibition by a panel of PAP- and PAPS-mimetic compounds. TPST1 (0.1 μM) was incubated with PAPS (5 μM) in the presence of the appropriate nucleoside analogue (400 μM) for 30 min at 20 °C. TPST1 activity was measured using fluorescently-labelled CC4-tide (2 μM) and normalised to DMSO (4% v/v) or buffer control. (D) Full dose–response curves for selected compounds. TPST1 (0.1 μM) was incubated with increasing concentrations of the indicated compound in the presence of PAPS (5 μM) for 30 min at 20 °C. TPST1 activity was measured using CC4-tide and normalised to DMSO or buffer controls as previously described. The data shown is from duplicate experiments.

The ability of 2′-deoxy-PAP and 1–14 to inhibit HS2ST and TPST1 were initially evaluated by *in vitro* enzyme assay, using PAP and coenzyme A (CoA) as the positive controls (both are competitive PAPS inhibitors), and the pan-kinase inhibitor staurosporine A as a negative control. Compounds were initially screened at 400 μM in the presence 20 nM HS2ST and 0.1 μM TPST1 respectively. Full dose–response curves were then obtained for the most active compounds. The results are summarised in Fig. 4A–D.

2′-Deoxy-PAP exhibited moderate inhibitory activity against HS2ST (IC_50_ = 12.7 μM ± 1.2) and TPST1 (IC_50_ = 3.6 μM ± 1.2) compared with PAP (IC_50_ = 2.0 μM and 1.5 μM respectively) (Fig. 4A–D). This demonstrates that the 2′-hydroxyl is not essential for binding. However, despite not appearing to make any contacts with the protein, its absence does reduce potency somewhat. This raises the possibility that the hydrogen bond from the 2′-hydroxyl to the 3′-phosphate (Fig. 2A and B) may function as a conformational lock,^75^ reducing the entropic loss on binding to the protein and warrants further study.

None of the PAP-mimetics (1–6) showed any inhibitory activity against HS2ST (Fig. 4A). Carboxylic acid derivatives can make electrostatic interaction with residues such as a lysine and arginine, which are often present in phosphate-binding protein domains.^76^ However, the trigonal geometry of carboxylates differs from the tetrahedral phosphate group. This may preclude efficient binding and alternative isosteres are currently being investigated. Several of the triazole-containing PAPS-mimetics (8, 9, 12 and 13) were weak inhibitors of HS2ST with IC_50_ values ranging from 100 to 300 μM (Fig. 4B). An identical structure–activity relationship (SAR) was observed for both the triazole-carboxylate (7–10) and triazole-phosphate (11–14) series. For compounds bearing a free carboxylate or phosphate (7, 8, 11 and 12), 3′-phosphorylation was essential for activity (*e.g.*7*vs.*8). In contrast, the corresponding benzylated analogues (9, 10, 13 and 14) showed activity only in the absence of 3′-phosphorylation (*e.g.*9*vs.*10).

Similar results were obtained using TPST1, although a broader range of inhibitory activities, compared with HS2ST, was observed (Fig. 4C). PAP-mimetics 5 and 6, and PAPS-mimetics 7–9, 12 and 13 were weak inhibitors with IC_50_ ranging from 100 to 300 μM (Fig. 4D). Comparing 5 and 6, the presence a benzyl group on the 5′-phosphate led to a decrease in activity. The SAR for PAPS-mimetics 7–14 was very similar to that observed against HS2ST although compound 7, which bears a free carboxylate and lacks a 3′-phosphate, also showed activity against TPST1.

**Fig. 5:**
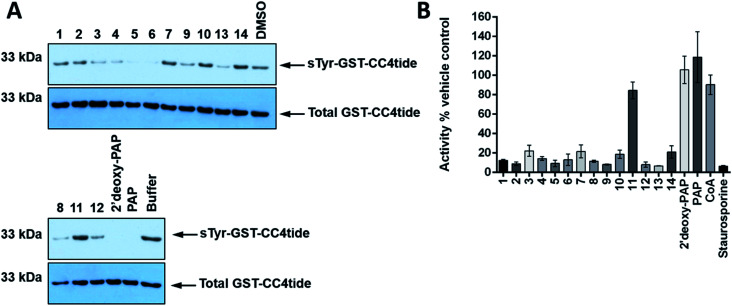
Immunological evaluation of TPST1 inhibition towards substrate and a Ser/Thr protein kinase counter-screen. (A) Immunoblots evaluating TPST1 sulfotransferase activity in the presence of a panel of PAP- and PAPS-mimetic compounds. GST-CC4-tide (1 μg) was incubated in the presence of TPST1 (0.2 μg), PAPS (5 μM), and a fixed concentration of the indicated compound (400 μM) for 15 min. After termination of the reaction using SDS-PAGE sample buffer, tyrosine sulfation was visualised by immunoblotting using a monoclonal sulfotyrosine antibody (top panel), with equal GST-CC4-tide and TPST1 loading confirmed using an antibody to detect 6xHis tagged proteins (bottom panel). (B) Enzymatic inhibition of PKA catalytic activity by a panel of PAP- and PAPS-mimetic compounds. PKA kinase (1 nM) was incubated with ATP (5 μM) in the presence of the appropriate nucleoside analogue (400 μM) for 30 min at 20 °C. PKA activity was calculated in real-time using fluorescently-labelled substrate peptide (2 μM) and normalised to DMSO (4% v/v) or buffer control.^77^ Data in B is mean and SD of 3 individual experiments. Staurosporine is included at 40 μM as a generic inhibitor of kinase activity. For A, similar results were seen in two independent experiments.

In order to further assess TPST1 inhibition by 2′-deoxy-PAP and compounds 1–14 using a complementary assay, immunoblotting of a TPST1 protein substrate (GST-CC4tide) was implemented with a monoclonal antibody that specifically recognises sulfotyrosine, using PAP as positive control (Fig. 5A). Compounds were screened at 400 μM in the assay. In common with the TP real-time ST1 enzyme assay using a synthetic peptide, a broad range of inhibitory activities was observed. For example, 2′-deoxy-PAP, and PAP-mimetics 5 and 6 showed complete suppression of tyrosine sulfation at this concentration. In contrast, bis-hydroxamic acid 3, bis-acid 4, and 8, 9, 12 and 13 showed partial suppression of tyrosine sulfation. However, PAPS-mimetic 7 did not show any activity in this assay.

Finally, a simple counter-screen was set up employing PKA. This was carried out in order to assess relative specificity for ST inhibition, and using the generic kinase inhibitor staurosporine A as a positive control and PAP and CoA as negative controls, neither of which bind nor inhibit PKA. Compounds were screened at 400 μM in the presence 1 nM PKA. The results are summarised in Fig. 5B. Interestingly, 2′-deoxy-PAP showed no activity against PKA, which demonstrates clearly that selective ST inhibition can be achieved from the 2′-deoxyadenosine scaffold. With the exception of 11, which was inactive in all of the ST assays, 1–14 all showed some inhibitory activity towards PKA indicating a lack of specificity for STs over a generic protein kinase.

Given that members of this first generation set of compounds are only modest inhibitors of TPST1 and HS2ST, and are derived from 2′-deoxyadenosine, it is perhaps unsurprising that this promiscuous inhibitory activity towards a protein kinase is observed. It is noteworthy that 2′-deoxy-PAP retained much of the ST inhibitory activity compared with PAP and also showed no PKA inhibition. Further work into relevant structural features of PAP and 2′-deoxy-PAP that engender ST-selective inhibition is ongoing, and the PAP- and PAPS-mimetics described in this study provide useful starting points for future development.

## Conclusions

Analysis of the nucleoside-binding domains of TPST1 and HS2ST and molecular docking identified 2′-deoxy-PAP as a structurally simplified analogue PAP. This led to the design of a library of potential inhibitors of the STs based on 2′-deoxyadenosine. Divergent syntheses of a range PAP- and PAPS-mimetics have been successfully developed. Evaluation of the inhibitory activity of the synthetic targets against HS2ST and TPST1 was carried out using our previously reported *in vitro* enzyme assays and a complementary immunoblot technique. A PKA counter-screen was also developed to assess specificity for ST inhibition. 2′-Deoxy-PAP, which to our knowledge has not previously been assessed as an ST inhibitor, was demonstrated to have attenuated activity towards HS2ST and TPST1 compared with PAP. This raises questions surrounding the intramolecular hydrogen bond between the 2′-hydroxyl and 3′-phosphate of PAP and the possibility that it may play a pre-organisational role in binding. PAPS-mimetics 8, 9, 12 and 13 were identified in all ST assays and, whilst weak inhibitors, represent opportunities for further investigation. We have previously identified, low/sub-micromolar sulfotransferase inhibitors such as rottlerin and suramin (non nucleoside-derived).^48,49^ The next step will be to obtain co-crystal structures of these and 8, 9, 12 and 13 with TPST1 and HS2ST which we are currently undertaking using published protocols. This will allow further structure-based compound design to be used. Interestingly, a broader range of inhibitory activities was shown against TPST1 (which sulfates proteins) relative to HS2ST (which sulfates glycans), which hints at the possibility of achieving selective ST inhibition in the future, perhaps by additional targeting of the distinct substrate-binding pockets in these enzymes.

## Conflicts of interest

There are no conflicts to declare.

## Supplementary Material

RA-009-C9RA07567D-s001
