# Supplementary material for: Structure-based design of nucleoside-derived analogues as sulfotransferase inhibitors
Source: RSC Adv. 2019 Oct 9;9(55):32165–73. doi: 10.1039/c9ra07567d (PMC9072872; doi:10.1039/c9ra07567d)
Supplement: RA-009-C9RA07567D-s001 [file RA-009-C9RA07567D-s001.pdf]

Electronic Supplementary Material (ESI) for RSC Advances.
This journal is © The Royal Society of Chemistry 2019

Structure-based design of nucleoside-derived analogues as sulfotransferase inhibitors

Neil M. Kershaw,*[a] Dominic P. Byrne,[b] Hollie Parsons,[a] Neil G. Berry,[a] David G. Fernig,[b] Patrick A. Eyers[b] and Richard Cosstick[a]

[a]Department of Chemistry, University of Liverpool, Liverpool L69 7ZD, U.K.

[b]Department of Biochemistry, Institute of Integrative Biology, University of Liverpool, Liverpool L69 7ZB, U.K.

Supporting Information

Table of Contents

Fig. SI 1. Comparison of the nucleoside-binding sites of TPST1 and HS2ST S1

Table SI 1. Molecular Docking S2

Fig. SI 2A-Q. Docking poses of PAP, PAPS, 2'-deoxy-PAP and 1-14 in TPST1 and HS2ST S3-19

Fig. SI 3. Molecular docking results of PAPS and compound 12 in TPST1 and HS2ST showing overlay of the 5'-triazolephosphate (12) and 5'-phosphosufate (PAPS) S20

Fig. SI 4. PAP(S)- and substrate-binding sites of TPST1 and HS2ST and molecular docking results of compound 9 S21

Fig. SI 5. ^{1}H NMR of 18 and subsequent acetylation product S22

Materials and Methods S23-39

References S40

NMR Spectra S41-113

Fig. SI 1. Comparison of the nucleoside-binding sites of TPST1 and HS2ST

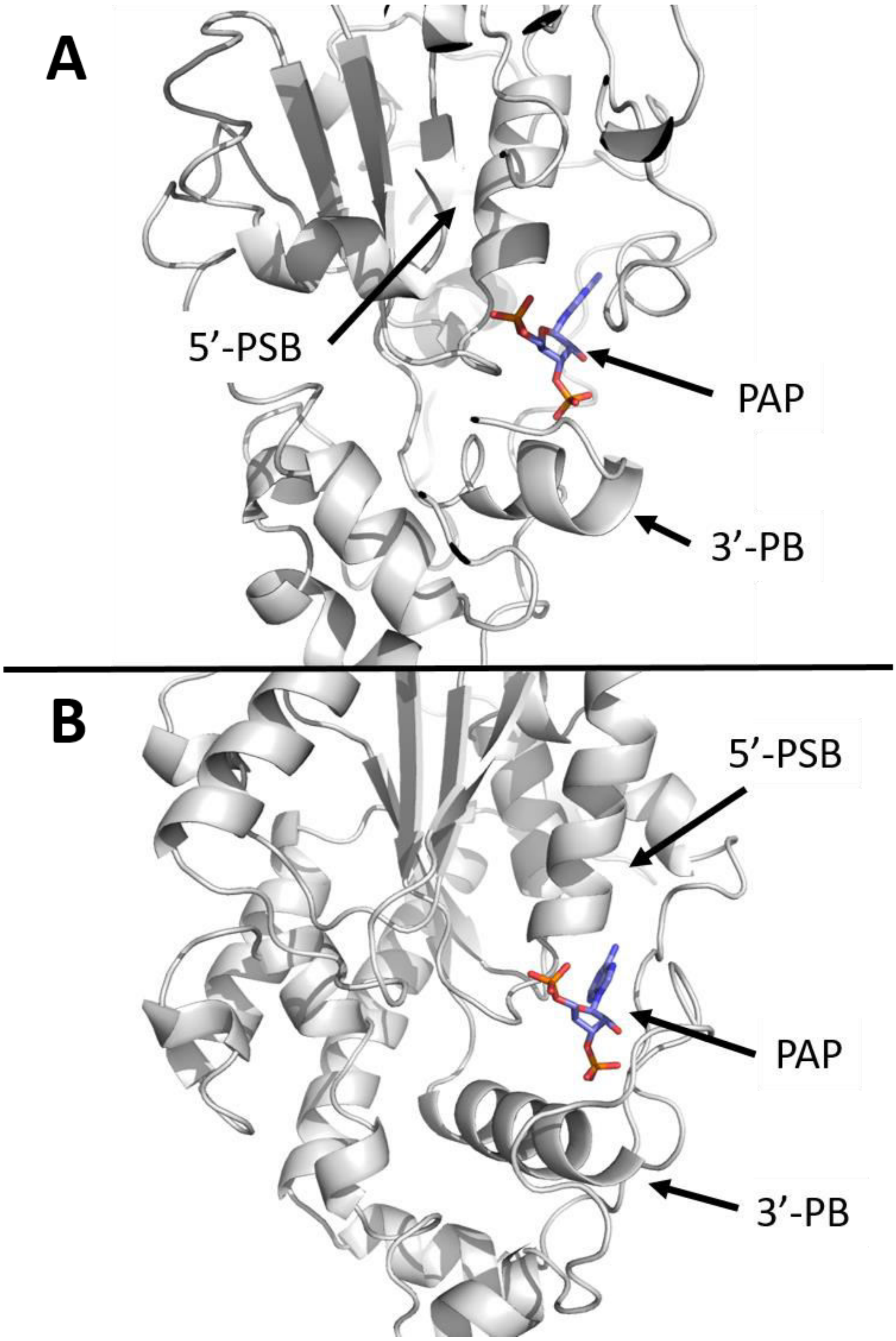

(A) Structure of human TPST1 complexed with PAP (PDB ID: 5WRI) highlighting the 5'-phosphosulfate-binding (5'-PSB) and 3'-phosphate-binding (3'-PB) motifs (protein rendered as grey cartoon). PAP is rendered as coloured sticks (carbon–slate, nitrogen–blue, oxygen–red).

(B) Structure of chicken MBP-HS2ST complexed with PAP (PDB ID: 4NDZ) highlighting the 5'-phosphosulfate-binding (5'-PSB) and 3'-phosphate-binding (3'-PB) motifs (protein rendered as grey cartoon). PAP is rendered as coloured sticks (icarbon–slate, nitrogen–blue, oxygen–red).

Table SI 1. Molecular Docking

Compound	Average ChemPLP (±standard deviation)	
	TPST1	HS2ST
**PAP**	122 (2.7)	103 (1.4)
**PAPS**	129 (5.6)	89 (4.6)
**2'-deoxyPAP**	133 (1.4)	103 (2.1)
**1**	94 (4.3)	86 (6.4)
**2**	93 (4.1)	86 (4.8)
**3**	97 (5.2)	85 (3.6)
**4**	118 (3.5)	98 (2.6)
**5**	111 (3.9)	87 (2.8)
**6**	103 (5.4)	100 (5.6)
**7**	83.3 (3.8)	82 (1.6)
**8**	116 (2.8)	109 (2.4)
**9**	91 (5.7)	96 (2.9)
**10**	127 (2.0)	124 (2.0)
**11**	87 (2.6)	84 (1.9)
**12**	111 (3.7)	105 (5.0)
**13**	92 (6.5)	103 (3.8)
**14**	117 (7.6)	128 (4.7)

Docking models for PAP, PAPS, 2-deoxy-PAP and compounds **1-14** were built using Spartan16 (http://wavefun.com) and energy minimised using the Merck molecular forcefield. Phosphates were built as their monobasic form. Carboxylic acids were built as carboxylates. GOLD 5.2 (CCDC Software) was used to dock molecules,[1] with the binding site defined as 6 Å around of any atom of PAP, using co-ordinates from human TPST1 PDB ID: 5WRI[2] and chicken MBP-HS2ST PDB ID: 4NDZ.[3] A generic algorithm with ChemPLP as the fitness function[4] was used. Each ligand was set to undergo 10 GA runs with no early termination allowed, lone pairs were not saved and all solutions were kept. Protons were added to the protein and all but the water molecules present in the active site were removed. Default settings were retained for the 'ligand flexibility' and 'fitness and search options' however, 'GA settings' were changed to 200%.

Fig. SI 2A. Docking pose of PAP in TPST1 and HS2ST

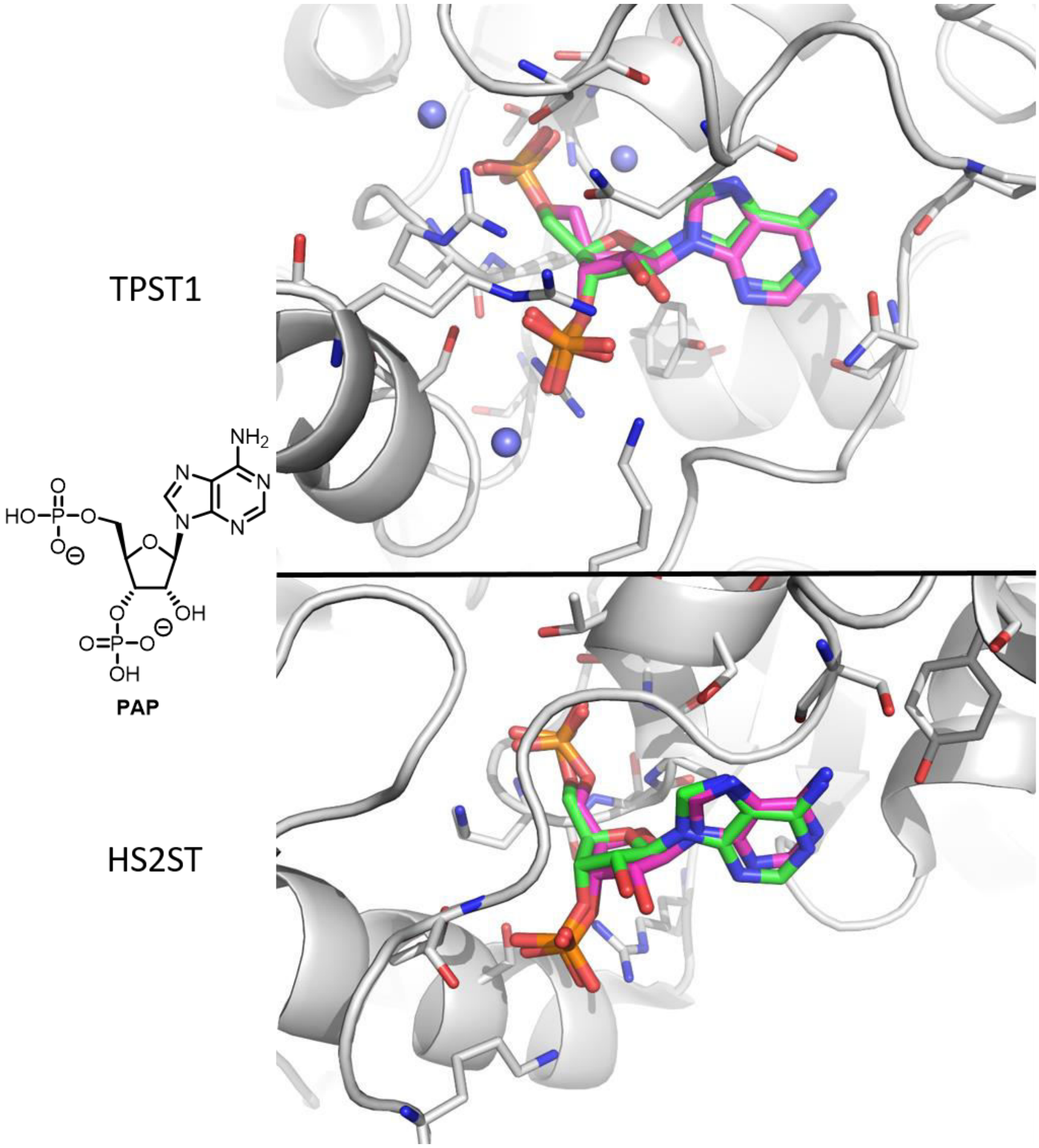

Molecular docking results of PAP in TPST1 and HS2ST. Protein is rendered as grey cartoon. Residues interacting with PAP are labelled and rendered as thin sticks (carbon–grey, nitrogen–blue, oxygen–red). Crystallographic waters are rendered as slate spheres. The crystallographic PAP is shown as a reference and is rendered as coloured sticks (carbon–green, nitrogen–blue, oxygen–red, phosphorus–orange). Docked PAP is rendered as coloured sticks (carbon–magenta, nitrogen–blue, oxygen–red, phosphorus–orange).

Fig. SI 2B. Docking pose of PAPS in TPST1 and HS2ST

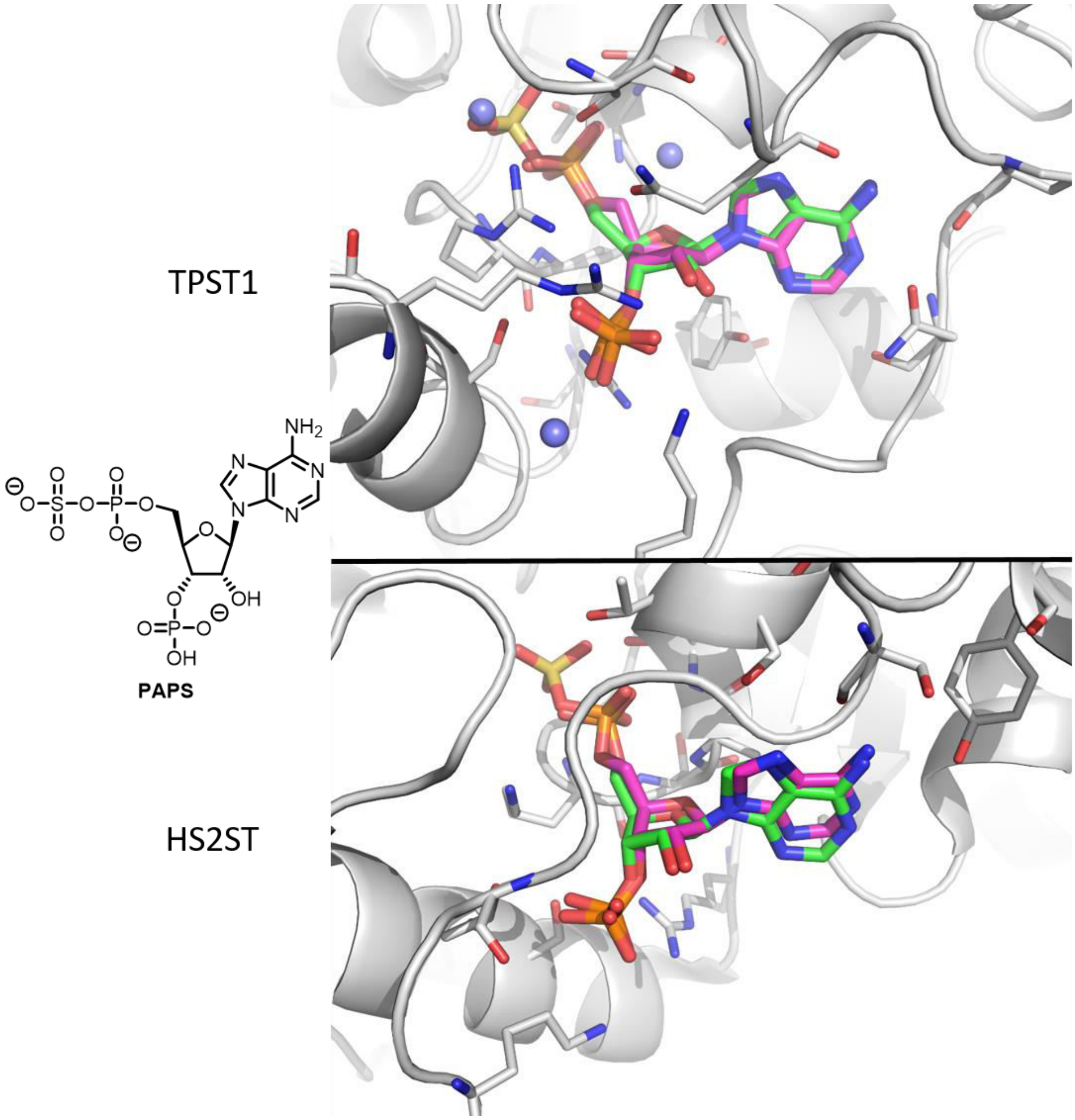

Molecular docking results of PAPS in TPST1 and HS2ST. Protein is rendered as grey cartoon. Residues interacting with PAP are labelled and rendered as thin sticks (carbon–grey, nitrogen–blue, oxygen–red). Crystallographic waters are rendered as slate spheres. PAP is shown as a reference and is rendered as coloured sticks (carbon–green, nitrogen–blue, oxygen–red, phosphorus–orange). PAPS is rendered as coloured sticks (carbon–magenta, nitrogen–blue, oxygen–red, phosphorus–orange, sulfur–yellow).

Fig. SI 2C. Docking pose of 2'-deoxy-PAP in TPST1 and HS2ST

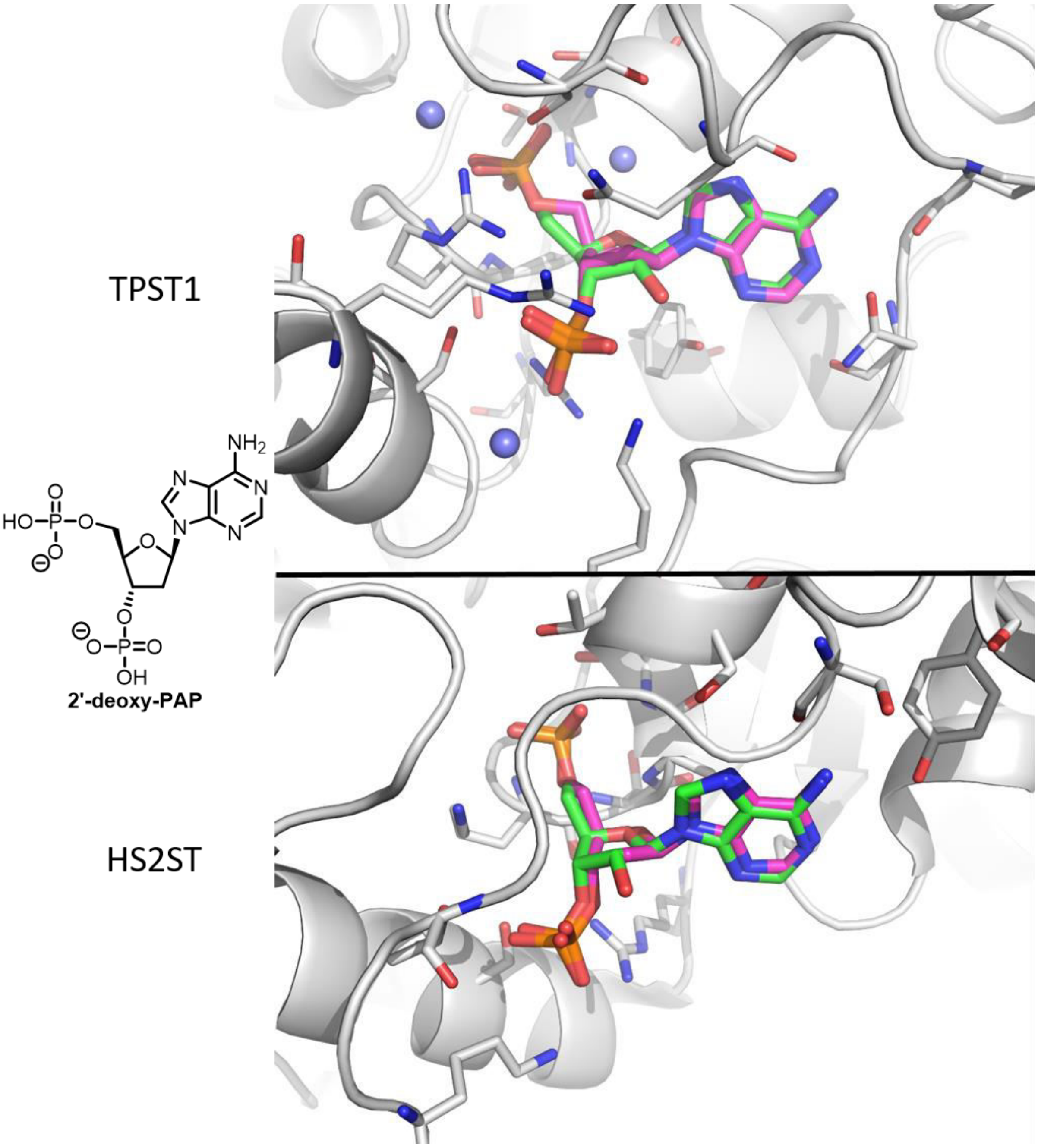

Molecular docking results of 2'-deoxy-PAP in TPST1 and HS2ST. Protein is rendered as grey cartoon. Residues interacting with PAP are labelled and rendered as thin sticks (carbon–grey, nitrogen–blue, oxygen–red). Crystallographic waters are rendered as slate spheres. PAP is shown as a reference and is rendered as coloured sticks (carbon–green, nitrogen–blue, oxygen–red, phosphorus–orange). 2'-deoxy-PAP is rendered as coloured sticks (carbon–magenta, nitrogen–blue, oxygen–red, phosphorus–orange).

Fig. SI 2D. Docking pose of compound 1 in TPST1 and HS2ST

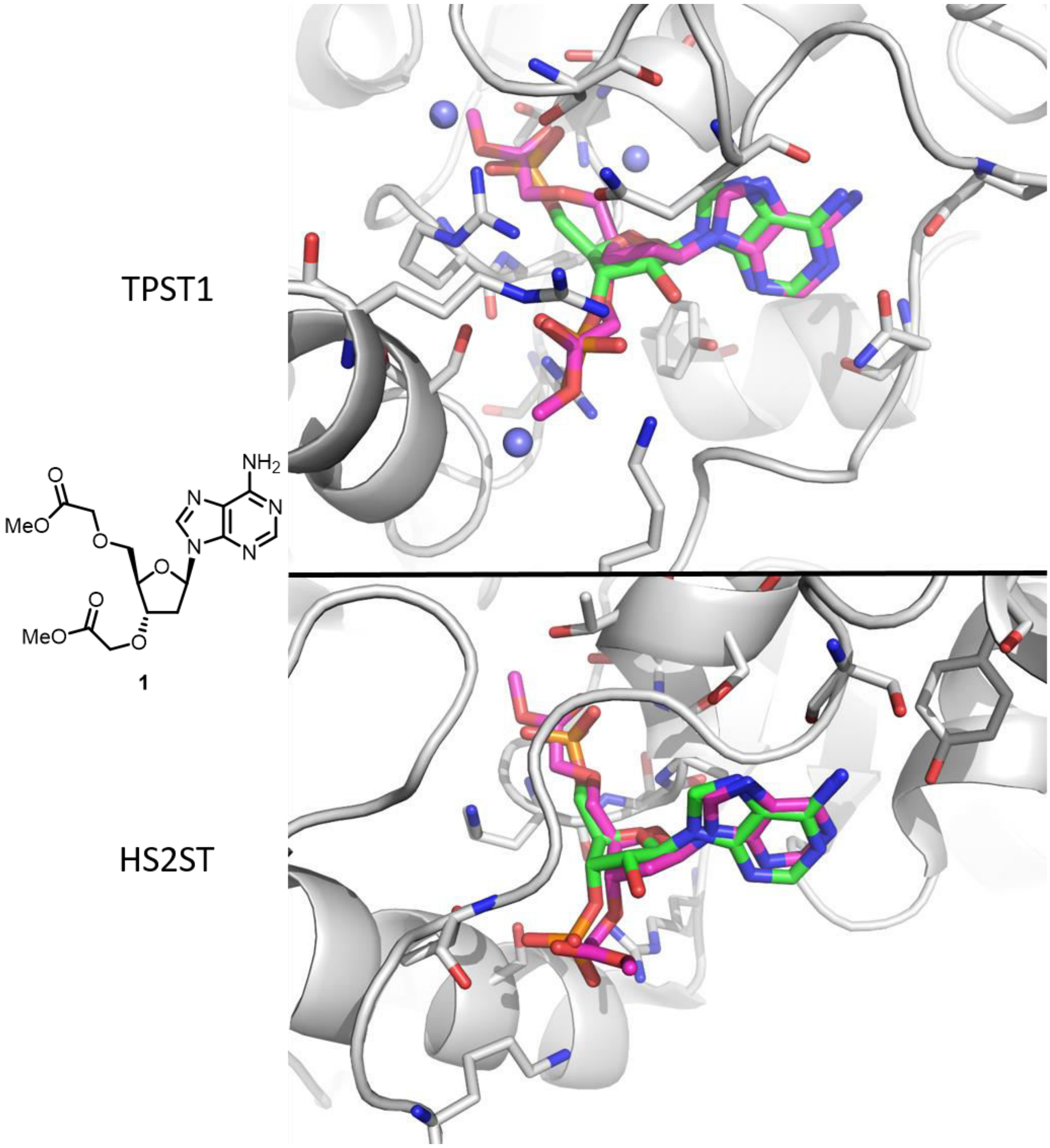

Molecular docking results of **1** in TPST1 and HS2ST. Protein is rendered as grey cartoon. Residues interacting with PAP are labelled and rendered as thin sticks (carbon–grey, nitrogen–blue, oxygen–red). Crystallographic waters are rendered as slate spheres. PAP is shown as a reference and is rendered as coloured sticks (carbon–green, nitrogen–blue, oxygen–red, phosphorus–orange). **1** is rendered as coloured sticks (carbon–magenta, nitrogen–blue, oxygen–red).

Fig. SI 2E. Docking pose of compound 2 in TPST1 and HS2ST

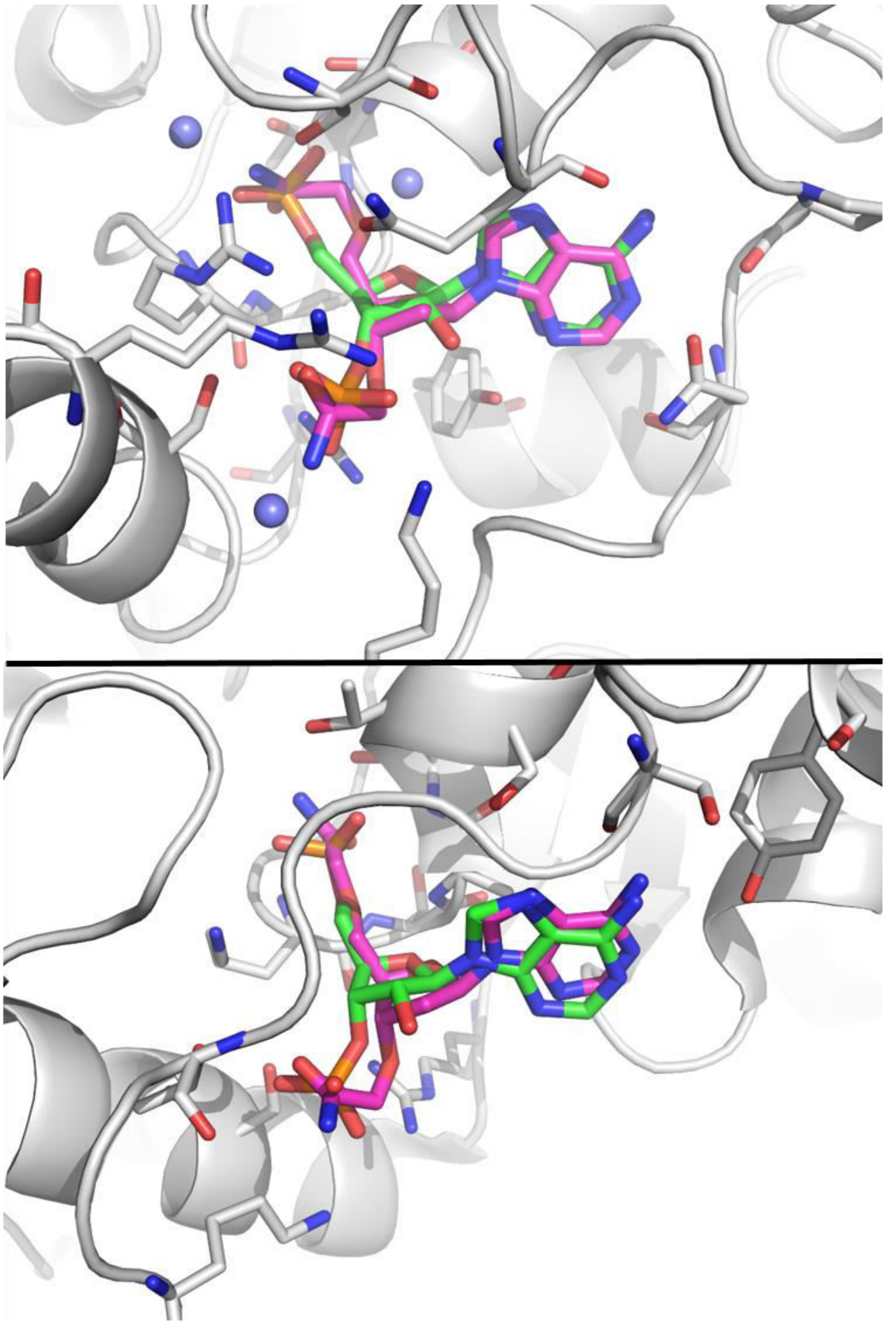

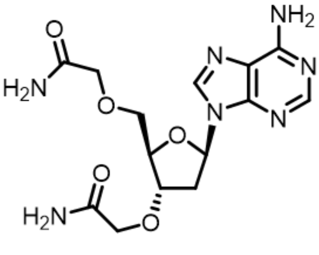

2

Molecular docking results of **2** in TPST1 and HS2ST. Protein is rendered as grey cartoon. Residues interacting with PAP are labelled and rendered as thin sticks (carbon–grey, nitrogen–blue, oxygen–red). Crystallographic waters are rendered as slate spheres. PAP is shown as a reference and is rendered as coloured sticks (carbon–green, nitrogen–blue, oxygen–red, phosphorus–orange). **2** is rendered as coloured sticks (carbon–magenta, nitrogen–blue, oxygen–red).

Fig. SI 2F. Docking pose of compound 3 in TPST1 and HS2ST

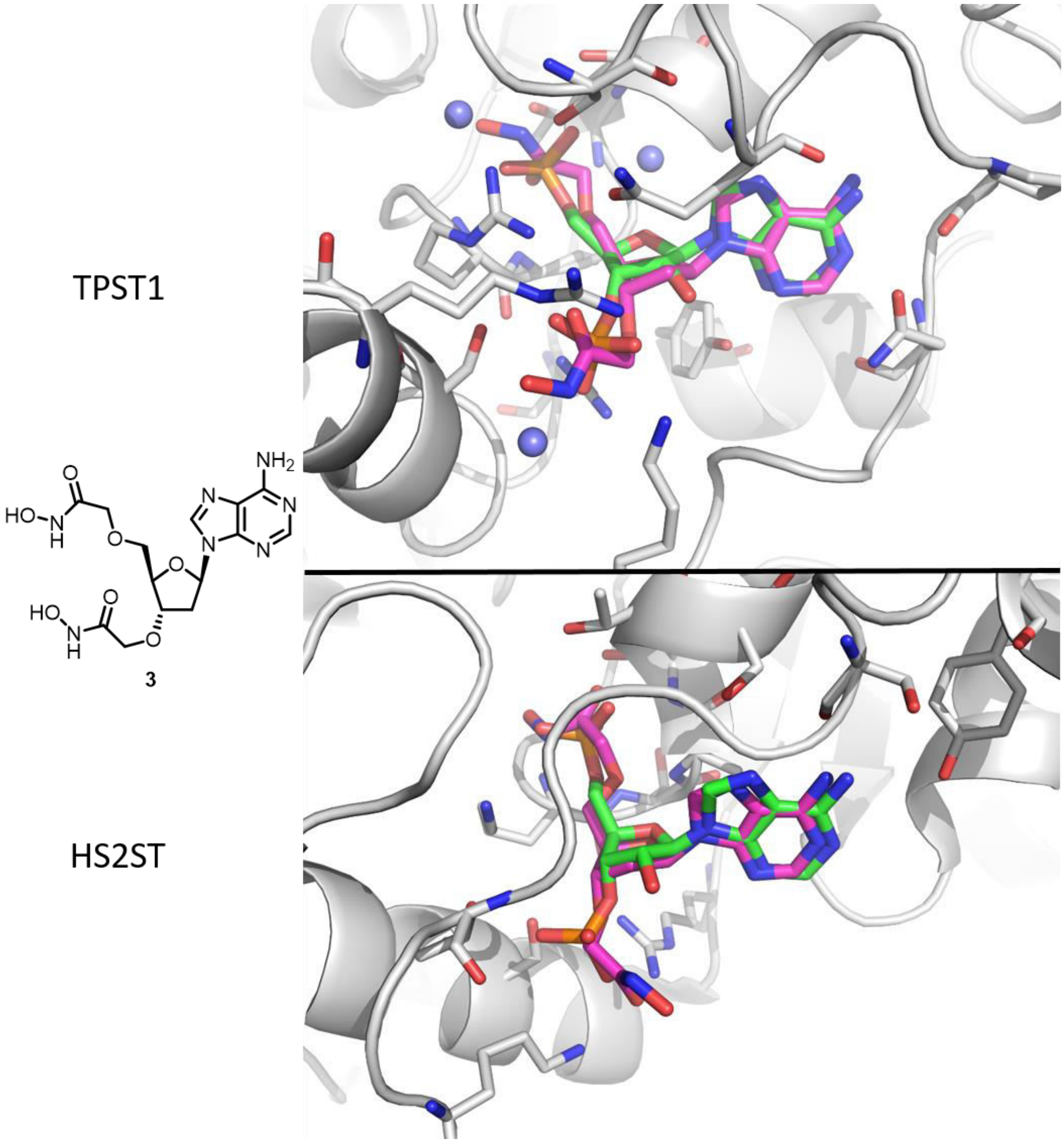

Molecular docking results of **3** in TPST1 and HS2ST. Protein is rendered as grey cartoon. Residues interacting with PAP are labelled and rendered as thin sticks (carbon–grey, nitrogen–blue, oxygen–red). Crystallographic waters are rendered as slate spheres. PAP is shown as a reference and is rendered as coloured sticks (carbon–green, nitrogen–blue, oxygen–red, phosphorus–orange). **3** is rendered as coloured sticks (carbon–magenta, nitrogen–blue, oxygen–red).

Fig. SI 2G. Docking pose of compound 4 in TPST1 and HS2ST

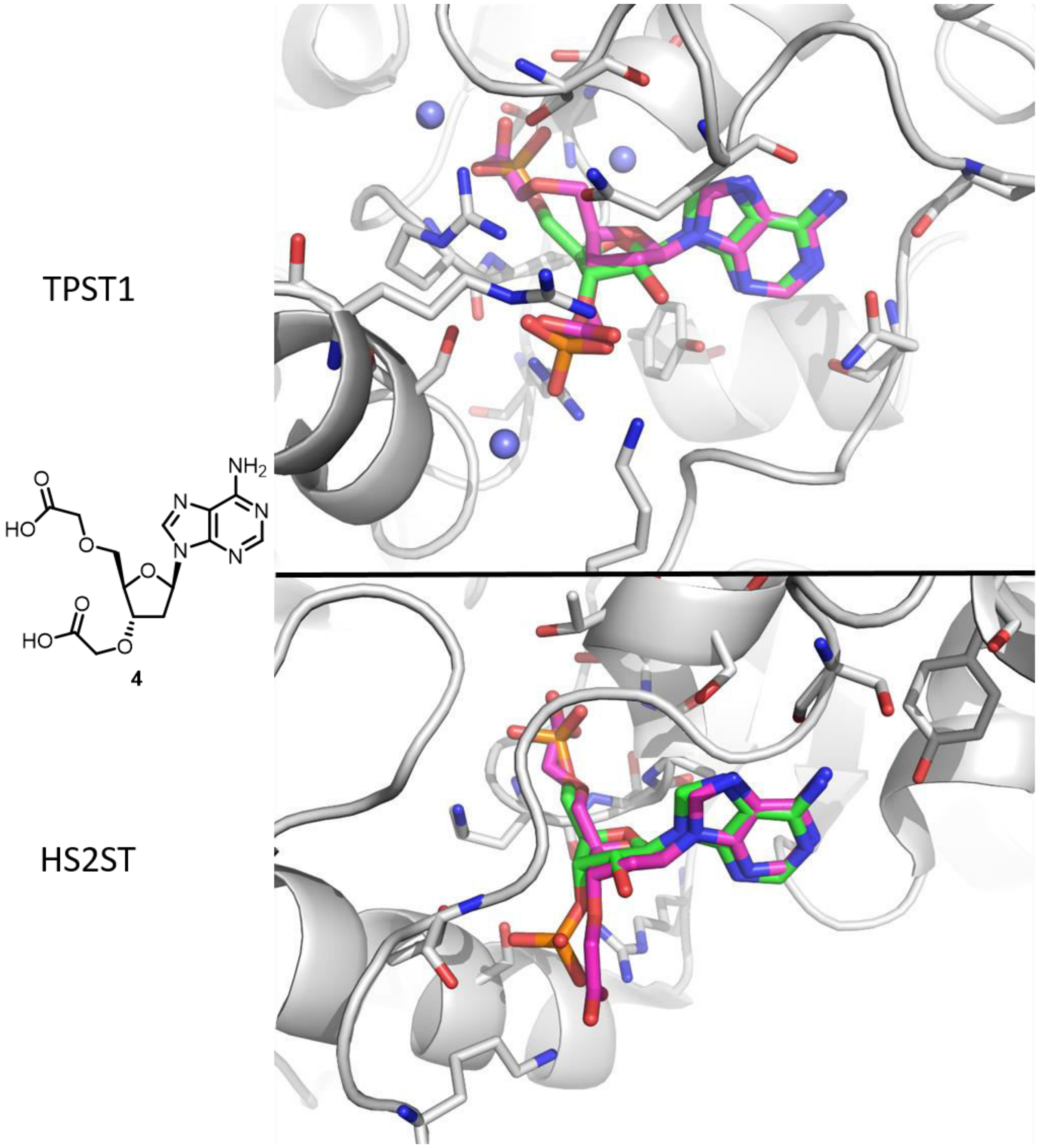

Molecular docking results of **4** in TPST1 and HS2ST. Protein is rendered as grey cartoon. Residues interacting with PAP are labelled and rendered as thin sticks (carbon–grey, nitrogen–blue, oxygen–red). Crystallographic waters are rendered as slate spheres. PAP is shown as a reference and is rendered as coloured sticks (carbon–green, nitrogen–blue, oxygen–red, phosphorus–orange). **4** is rendered as coloured sticks (carbon–magenta, nitrogen–blue, oxygen–red).

Fig. SI 2H. Docking pose of compound 5 in TPST1 and HS2ST

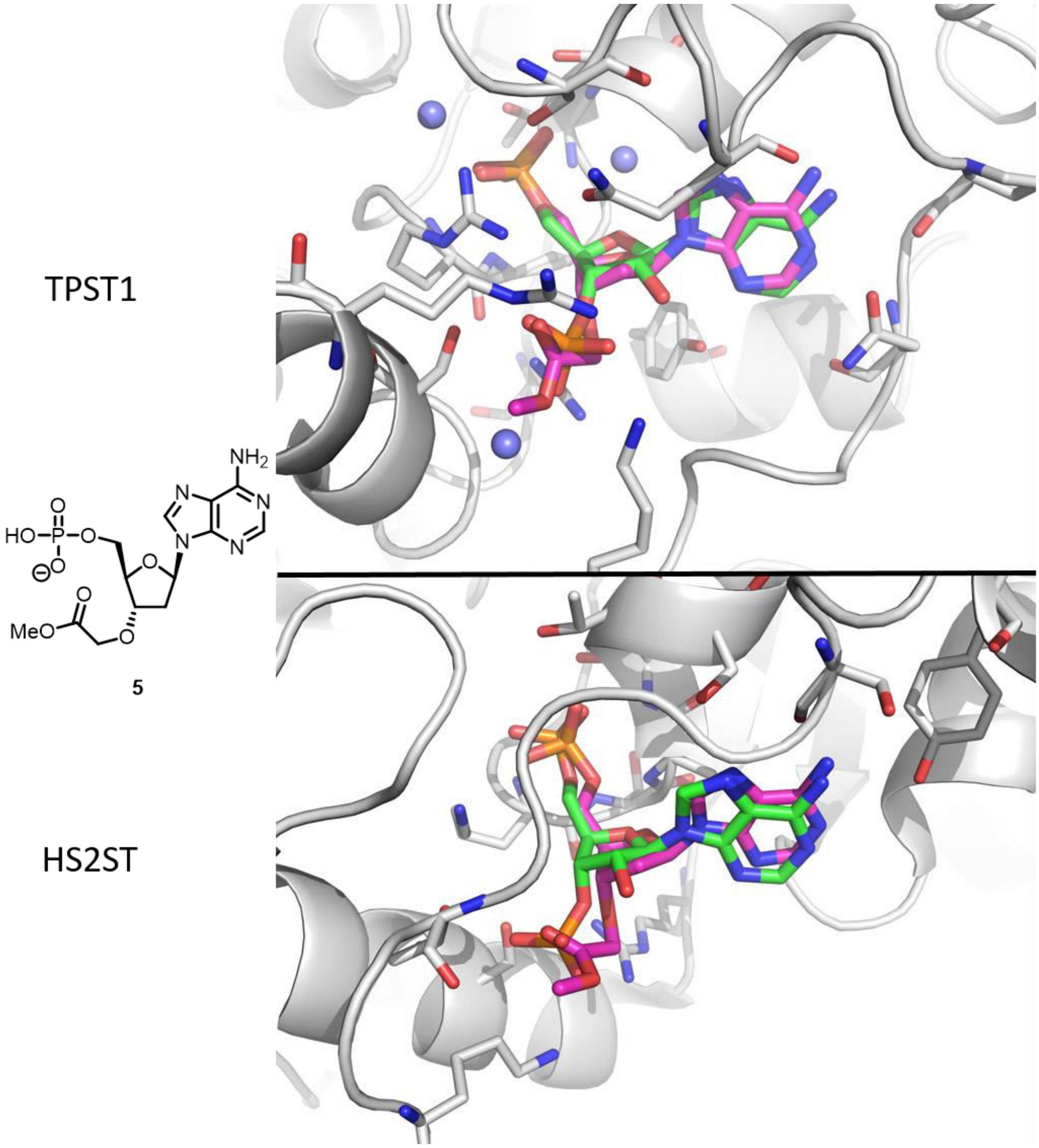

Molecular docking results of **5** in TPST1 and HS2ST. Protein is rendered as grey cartoon. Residues interacting with PAP are labelled and rendered as thin sticks (carbon–grey, nitrogen–blue, oxygen–red). Crystallographic waters are rendered as slate spheres. PAP is shown as a reference and is rendered as coloured sticks (carbon–green, nitrogen–blue, oxygen–red, phosphorus–orange). **5** is rendered as coloured sticks (carbon–magenta, nitrogen–blue, oxygen–red, phosphorus–orange).

Fig. SI 2I. Docking pose of compound 6 in TPST1 and HS2ST

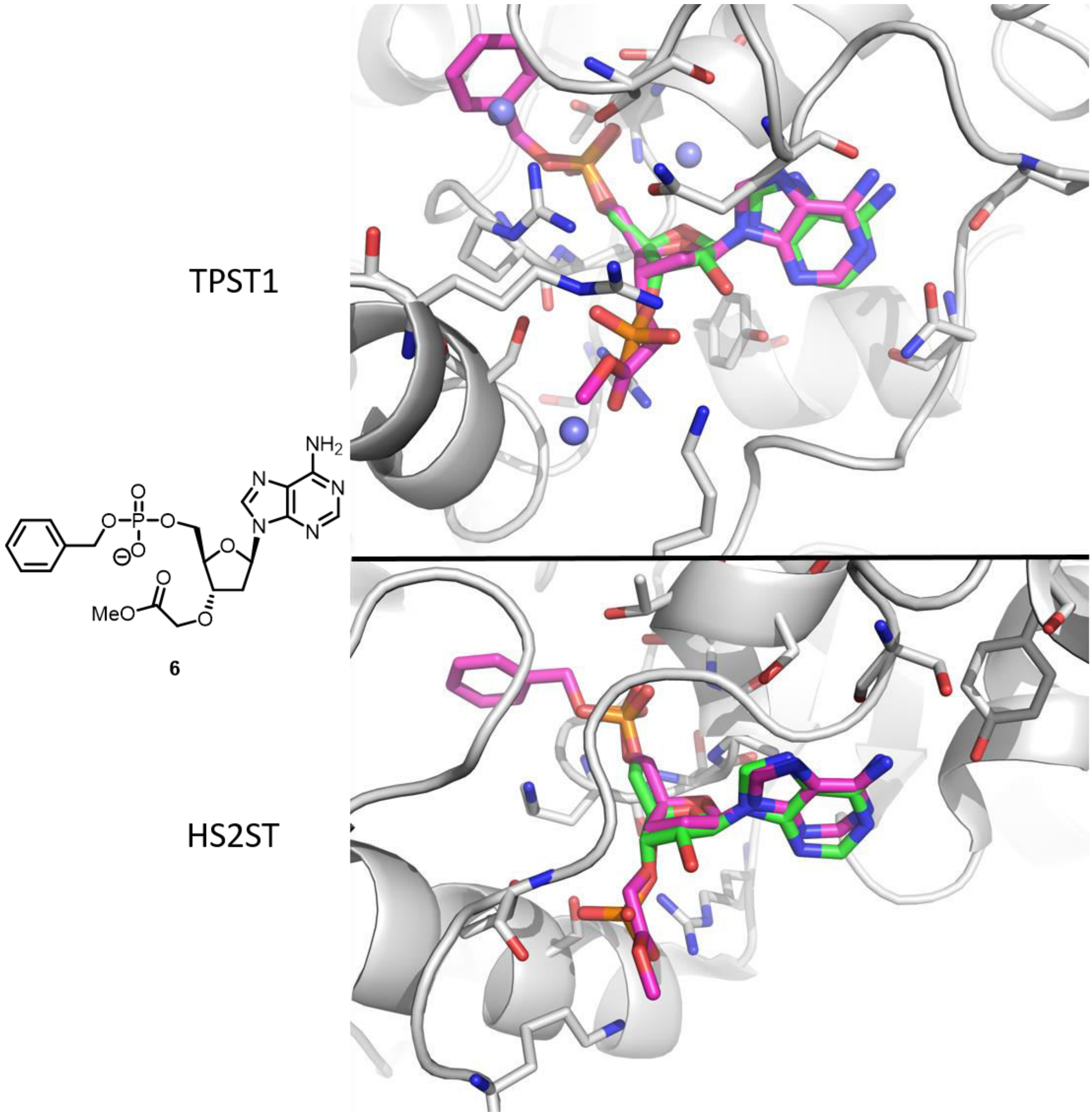

Molecular docking results of **6** in TPST1 and HS2ST. Protein is rendered as grey cartoon. Residues interacting with PAP are labelled and rendered as thin sticks (carbon–grey, nitrogen–blue, oxygen–red). Crystallographic waters are rendered as slate spheres. PAP is shown as a reference and is rendered as coloured sticks (carbon–green, nitrogen–blue, oxygen–red, phosphorus–orange). **6** is rendered as coloured sticks (carbon–magenta, nitrogen–blue, oxygen–red, phosphorus–orange).

Fig. SI 2J. Docking pose of compound 7 in TPST1 and HS2ST

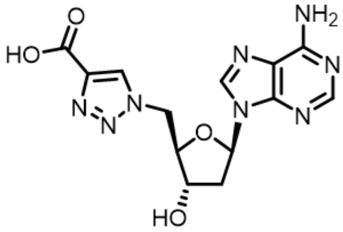

7

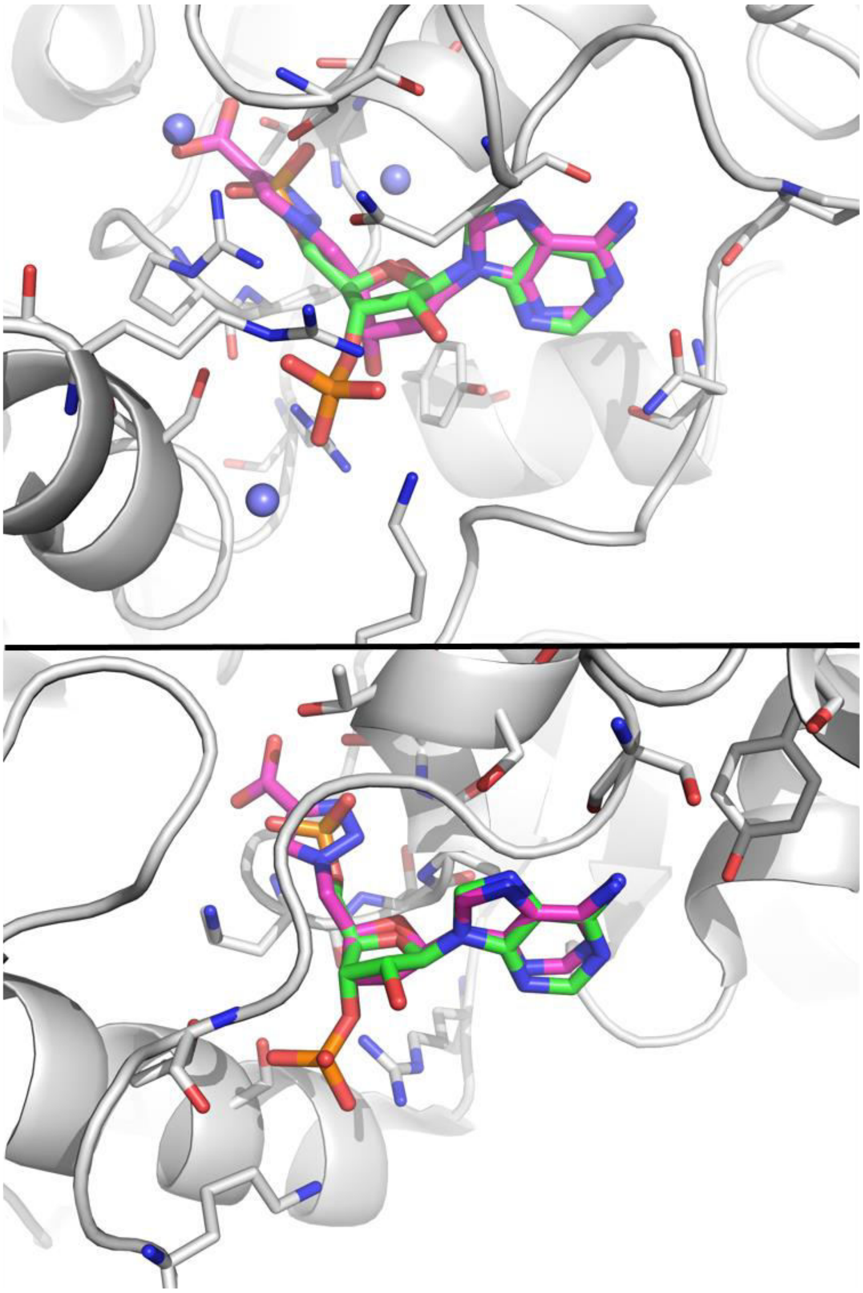

Molecular docking results of **7** in TPST1 and HS2ST. Protein is rendered as grey cartoon. Residues interacting with PAP are labelled and rendered as thin sticks (carbon–grey, nitrogen–blue, oxygen–red). Crystallographic waters are rendered as slate spheres. PAP is shown as a reference and is rendered as coloured sticks (carbon–green, nitrogen–blue, oxygen–red, phosphorus–orange). **7** is rendered as coloured sticks (carbon–magenta, nitrogen–blue, oxygen–red).

Fig. SI 2K. Docking pose of compound 8 in TPST1 and HS2ST

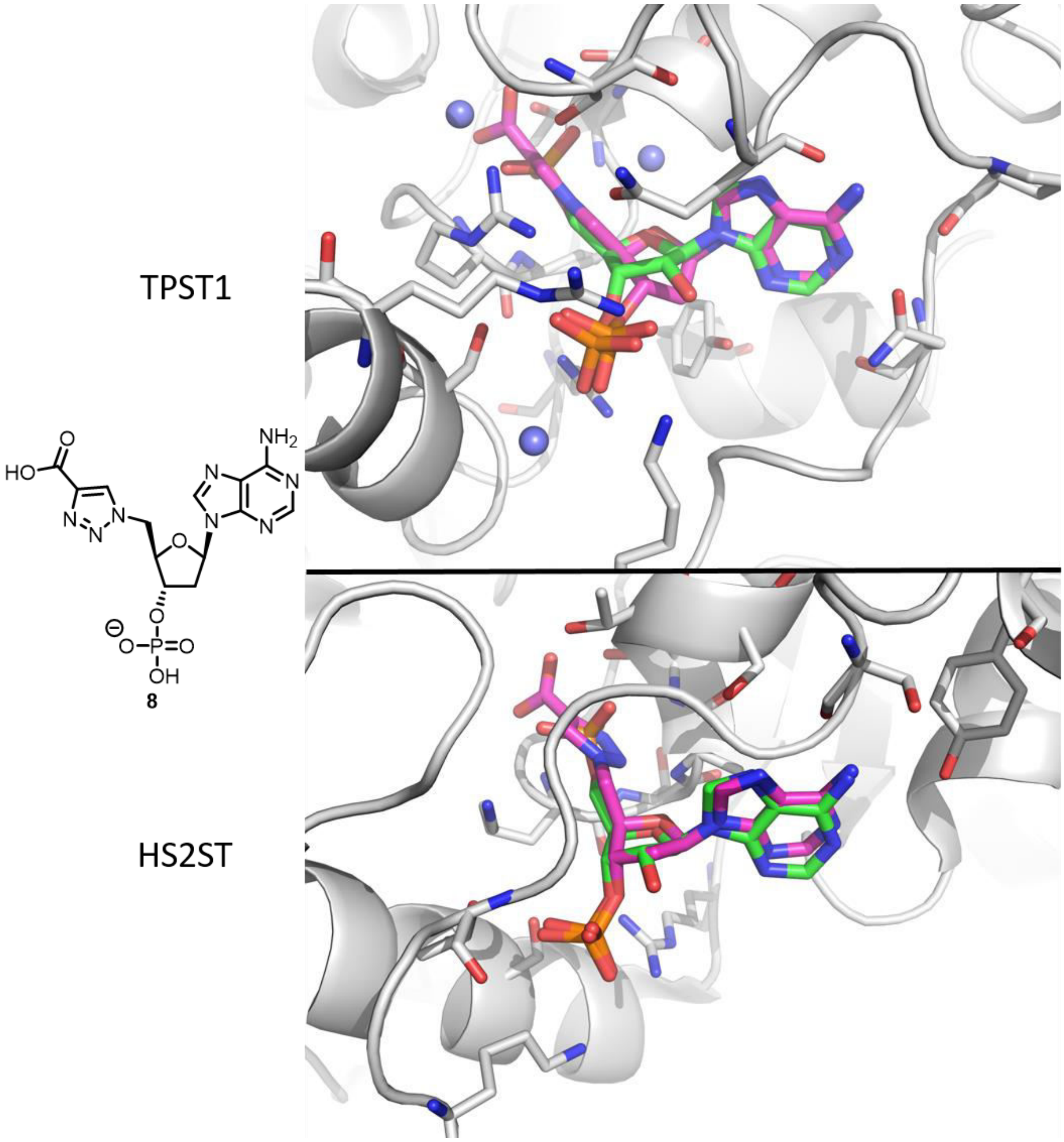

Molecular docking results of **8** in TPST1 and HS2ST. Protein is rendered as grey cartoon. Residues interacting with PAP are labelled and rendered as thin sticks (carbon–grey, nitrogen–blue, oxygen–red). Crystallographic waters are rendered as slate spheres. PAP is shown as a reference and is rendered as coloured sticks (carbon–green, nitrogen–blue, oxygen–red, phosphorus–orange). **8** is rendered as coloured sticks (carbon–magenta, nitrogen–blue, oxygen–red, phosphorus–orange).

Fig. SI 2L. Docking pose of compound 9 in TPST1 and HS2ST

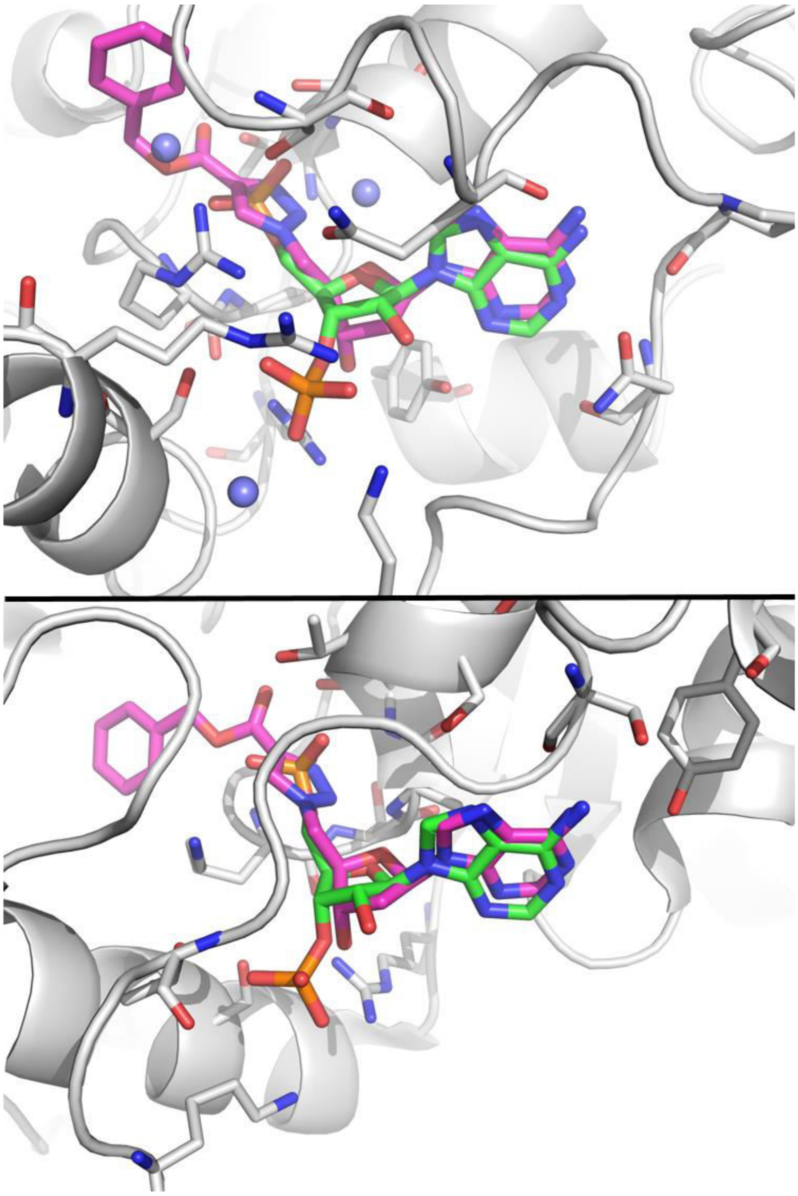

TPST1

HS2ST

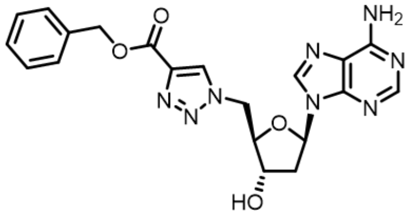

9

Molecular docking results of **8** in TPST1 and HS2ST. Protein is rendered as grey cartoon. Residues interacting with PAP are labelled and rendered as thin sticks (carbon–grey, nitrogen–blue, oxygen–red). Crystallographic waters are rendered as slate spheres. PAP is shown as a reference and is rendered as coloured sticks (carbon–green, nitrogen–blue, oxygen–red, phosphorus–orange). **8** is rendered as coloured sticks (carbon–magenta, nitrogen–blue, oxygen–red).

Fig. SI 2M. Docking pose of compound 10 in TPST1 and HS2ST

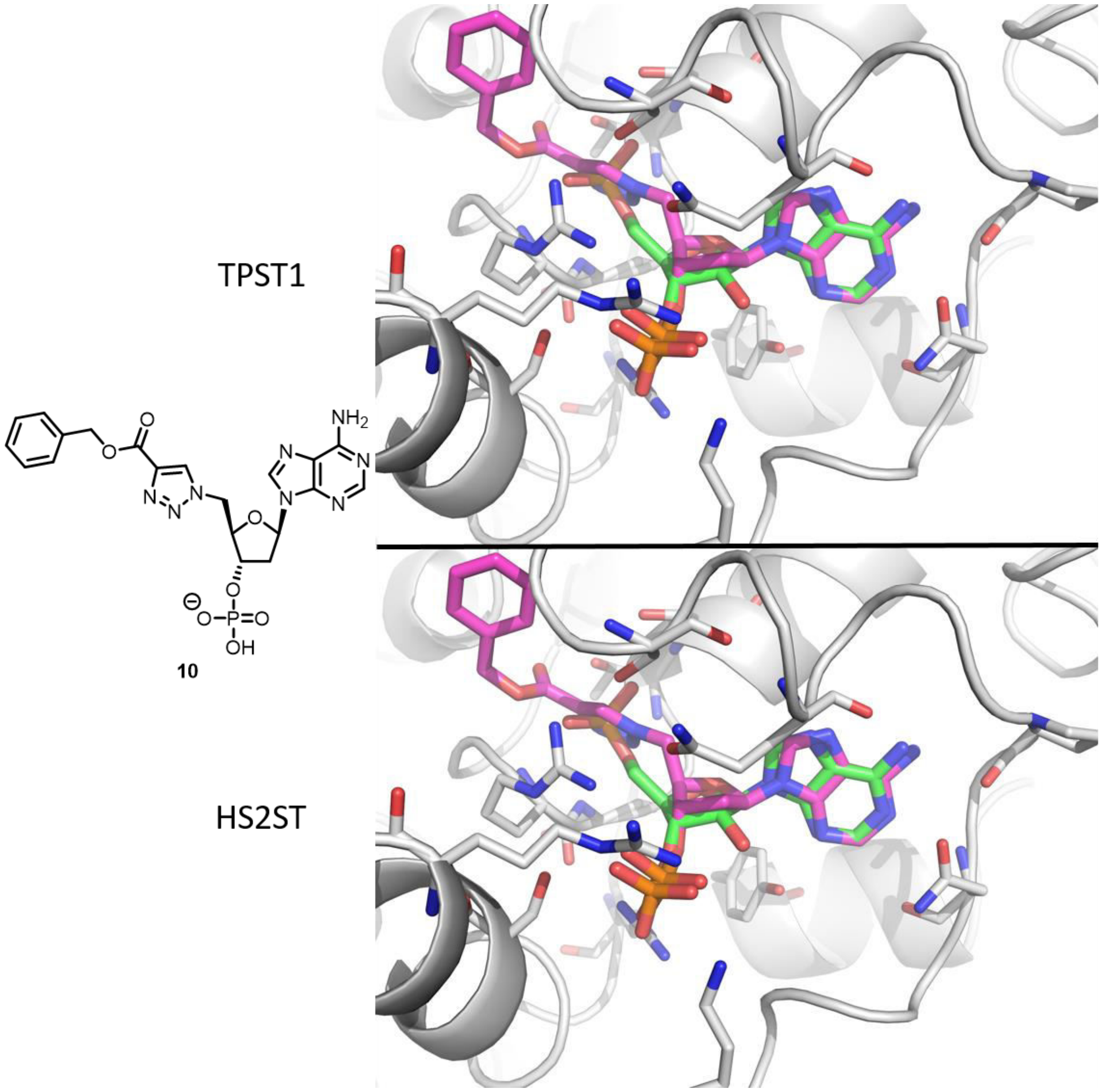

Molecular docking results of **10** in TPST1 and HS2ST. Protein is rendered as grey cartoon. Residues interacting with PAP are labelled and rendered as thin sticks (carbon–grey, nitrogen–blue, oxygen–red). Crystallographic waters are rendered as slate spheres. PAP is shown as a reference and is rendered as coloured sticks (carbon–green, nitrogen–blue, oxygen–red, phosphorus–orange). **10** is rendered as coloured sticks (carbon–magenta, nitrogen–blue, oxygen–red, phosphorus–orange).

Fig. SI 2N. Docking pose of compound 11 in TPST1 and HS2ST

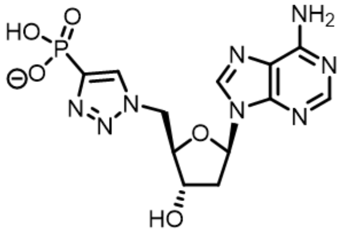

11

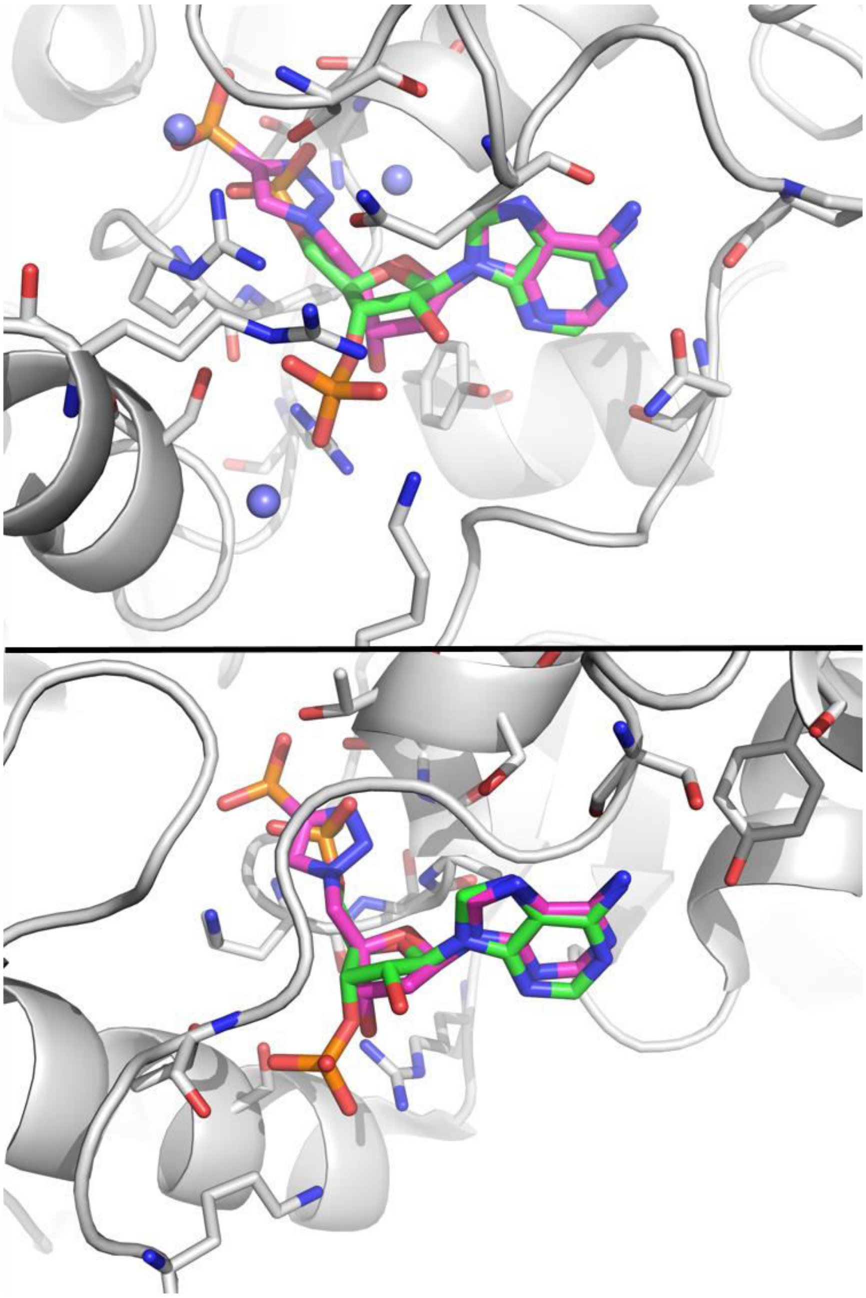

Molecular docking results of **11** in TPST1 and HS2ST. Protein is rendered as grey cartoon. Residues interacting with PAP are labelled and rendered as thin sticks (carbon–grey, nitrogen–blue, oxygen–red). Crystallographic waters are rendered as slate spheres. PAP is shown as a reference and is rendered as coloured sticks (carbon–green, nitrogen–blue, oxygen–red, phosphorus–orange). **11** is rendered as coloured sticks (carbon–magenta, nitrogen–blue, oxygen–red, phosphorus–orange).

Fig. SI 2O. Docking pose of compound 12 in TPST1 and HS2ST

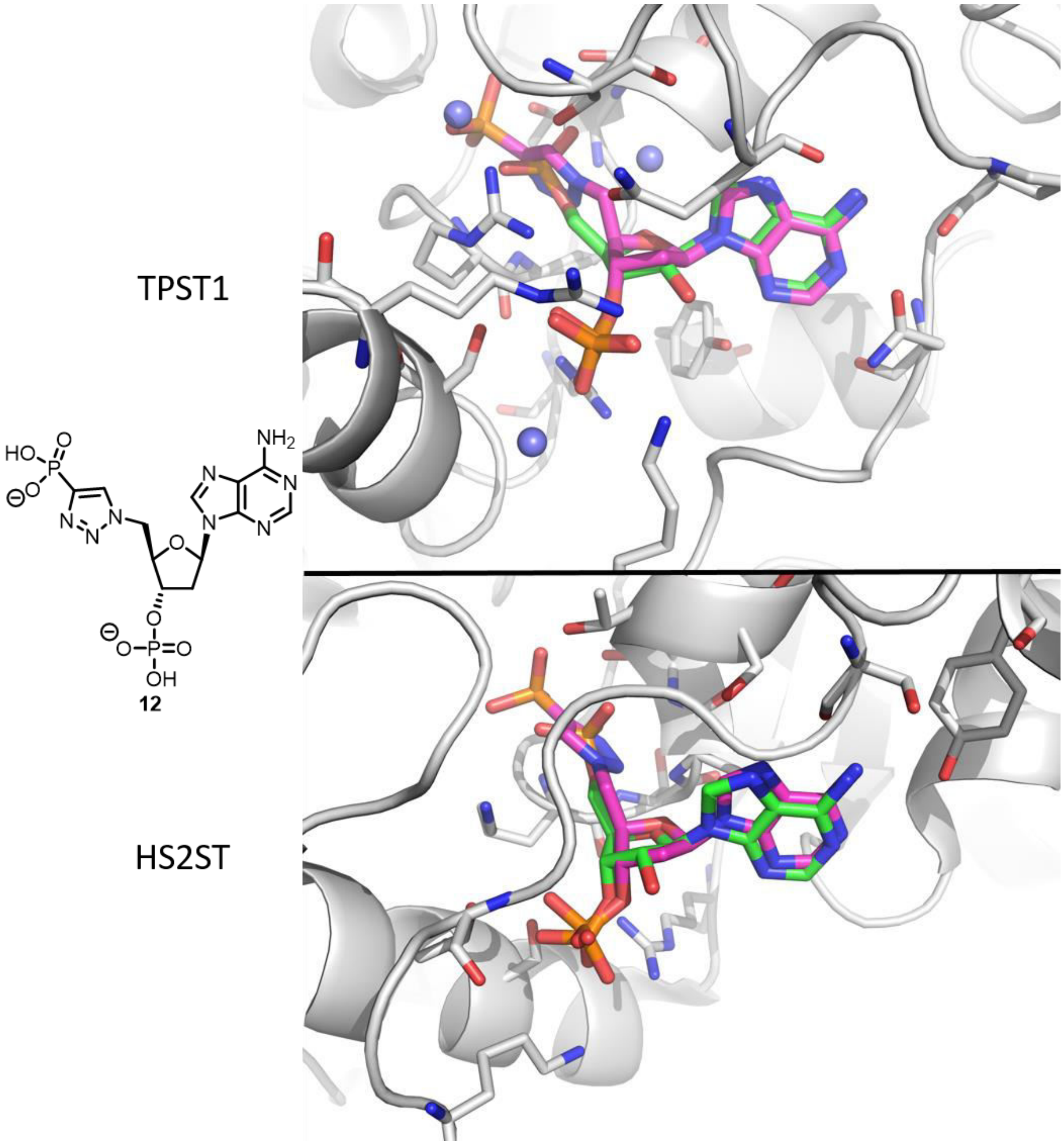

Molecular docking results of **12** in TPST1 and HS2ST. Protein is rendered as grey cartoon. Residues interacting with PAP are labelled and rendered as thin sticks (carbon–grey, nitrogen–blue, oxygen–red). Crystallographic waters are rendered as slate spheres. PAP is shown as a reference and is rendered as coloured sticks (carbon–green, nitrogen–blue, oxygen–red, phosphorus–orange). **12** is rendered as coloured sticks (carbon–magenta, nitrogen–blue, oxygen–red, phosphorus–orange).

Fig. SI 2P. Docking pose of compound 13 in TPST1 and HS2ST

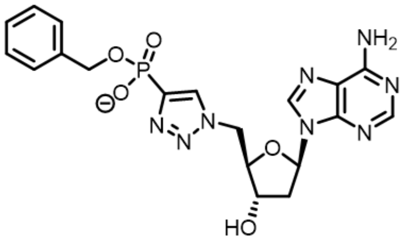

13

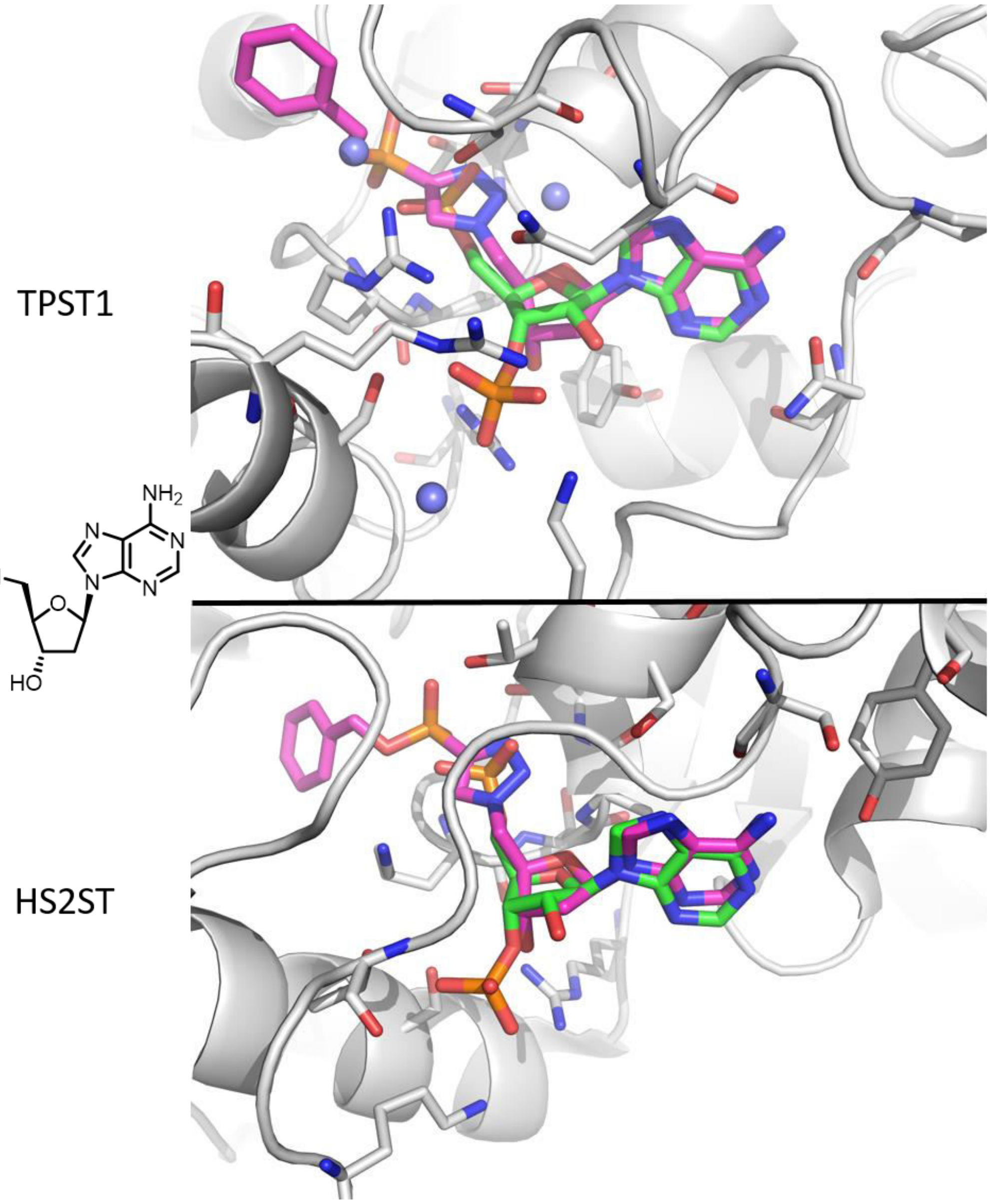

Molecular docking results of **13** in TPST1 and HS2ST. Protein is rendered as grey cartoon. Residues interacting with PAP are labelled and rendered as thin sticks (carbon–grey, nitrogen–blue, oxygen–red). Crystallographic waters are rendered as slate spheres. PAP is shown as a reference and is rendered as coloured sticks (carbon–green, nitrogen–blue, oxygen–red, phosphorus–orange). **13** is rendered as coloured sticks (carbon–magenta, nitrogen–blue, oxygen–red, phosphorus–orange).

Fig. SI 2Q. Docking pose of compound 14 in TPST1 and HS2ST

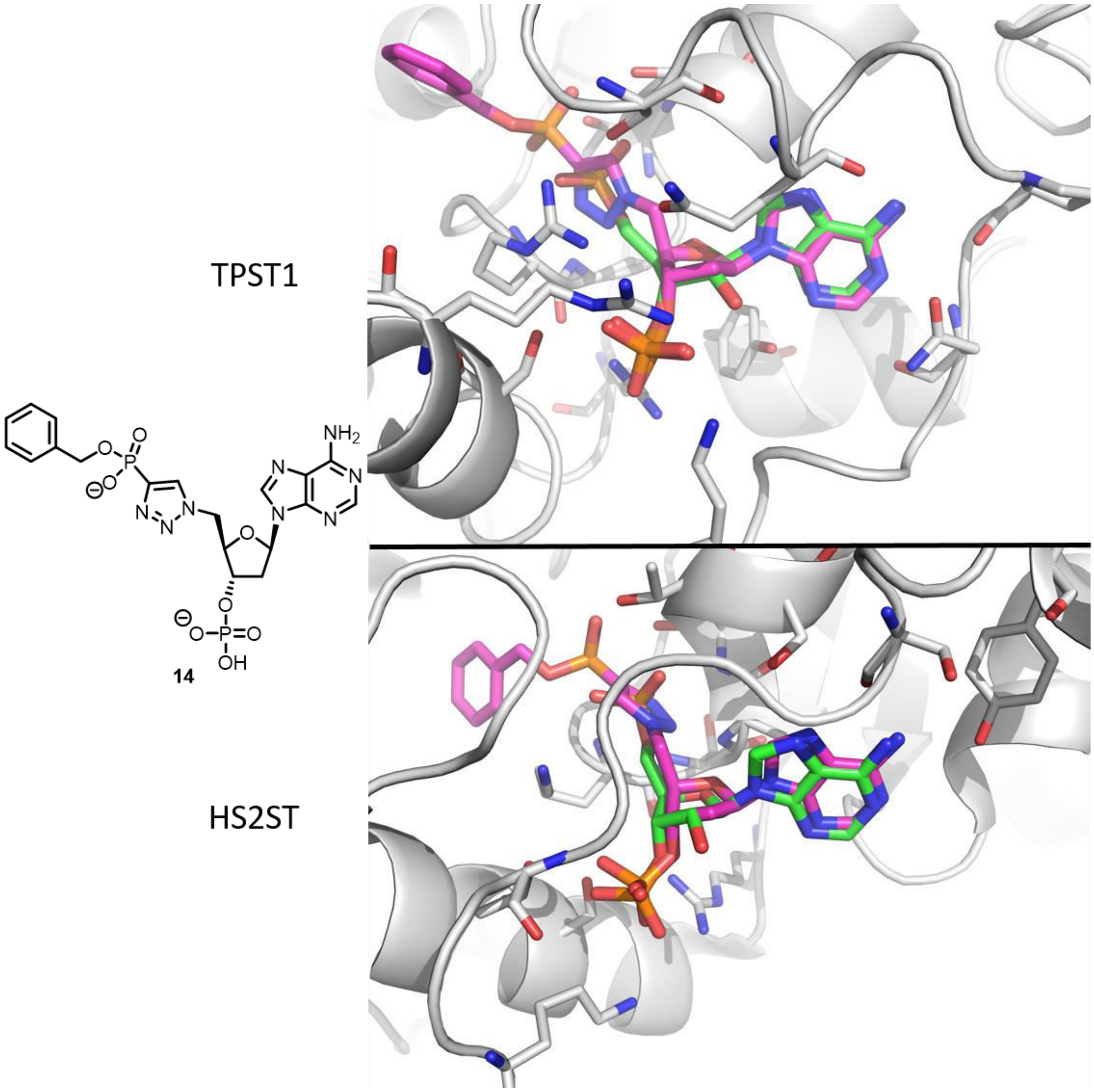

Molecular docking results of **14** in TPST1 and HS2ST. Protein is rendered as grey cartoon. Residues interacting with PAP are labelled and rendered as thin sticks (carbon–grey, nitrogen–blue, oxygen–red). Crystallographic waters are rendered as slate spheres. PAP is shown as a reference and is rendered as coloured sticks (carbon–green, nitrogen–blue, oxygen–red, phosphorus–orange). **14** is rendered as coloured sticks (carbon–magenta, nitrogen–blue, oxygen–red, phosphorus–orange).

Fig. SI 3. Molecular docking results of PAPS and compound 12 in TPST1 and HS2ST showing overlay of the 5'-triazolephosphate (12) and 5'-phosphosufate (PAPS)

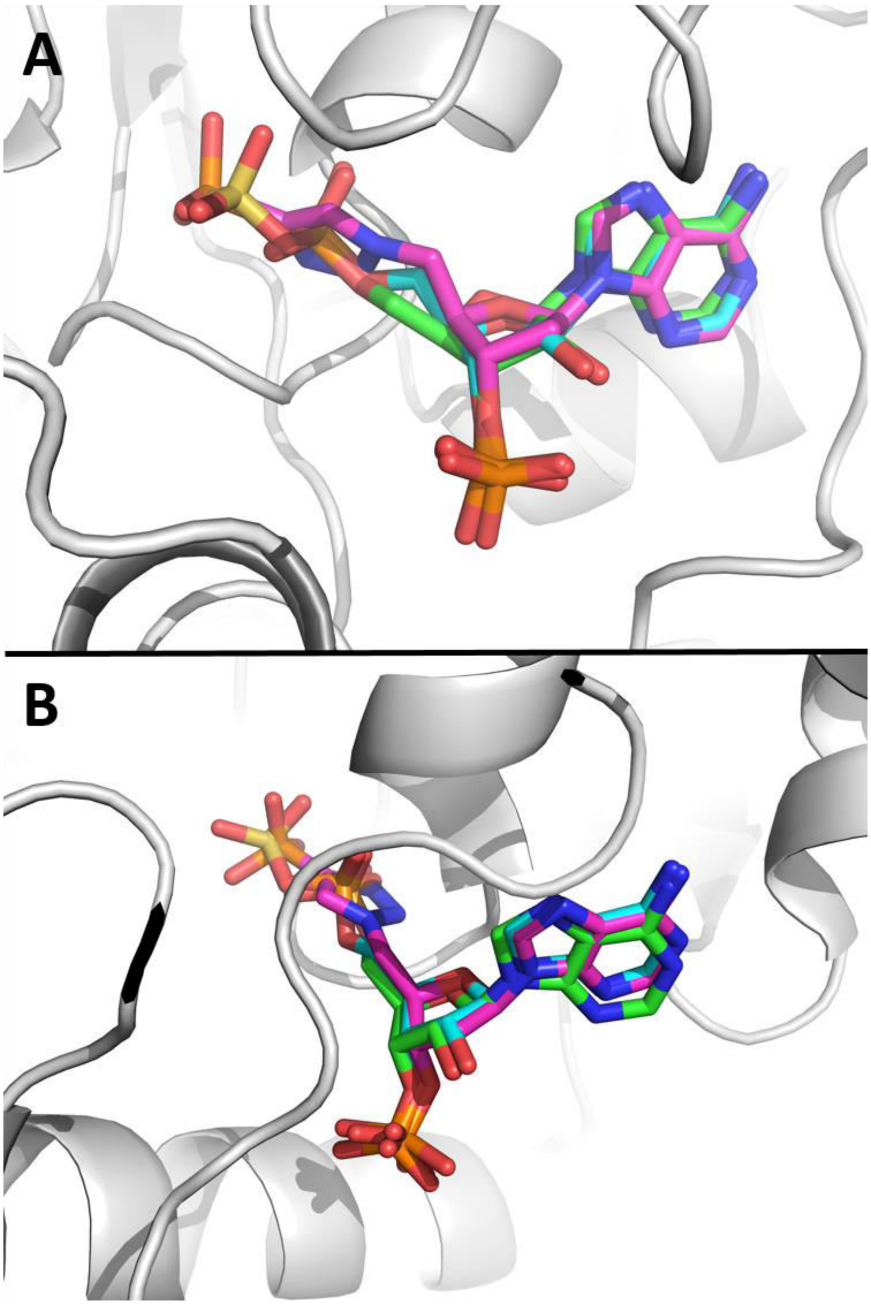

(A) Overlay of the molecular docking results of PAPS and **12** into TPST1. Protein is rendered as grey cartoon. PAP is shown as a reference and is rendered as coloured sticks (carbon–green, nitrogen–blue, oxygen–red, phosphorus–orange). PAPS is rendered as coloured sticks (carbon–cyan, nitrogen–blue, oxygen–red, phosphorus–orange, sulfur–yellow). **12** is rendered as coloured sticks (carbon–magenta, nitrogen–blue, oxygen–red, phosphorus–orange).

(B) Overlay of the molecular docking results of PAPS and **12** into HS2ST. Protein is rendered as grey cartoon. PAP is shown as a reference and is rendered as coloured sticks (carbon–green, nitrogen–blue, oxygen–red, phosphorus–orange). PAPS is rendered as coloured sticks (carbon–cyan, nitrogen–blue, oxygen–red, phosphorus–orange, sulfur–yellow). **12** is rendered as coloured sticks (carbon–magenta, nitrogen–blue, oxygen–red, phosphorus–orange).

Fig. SI 4. PAP(S)- and substrate-binding sites of TPST1 and HS2ST and molecular docking results of compound 9

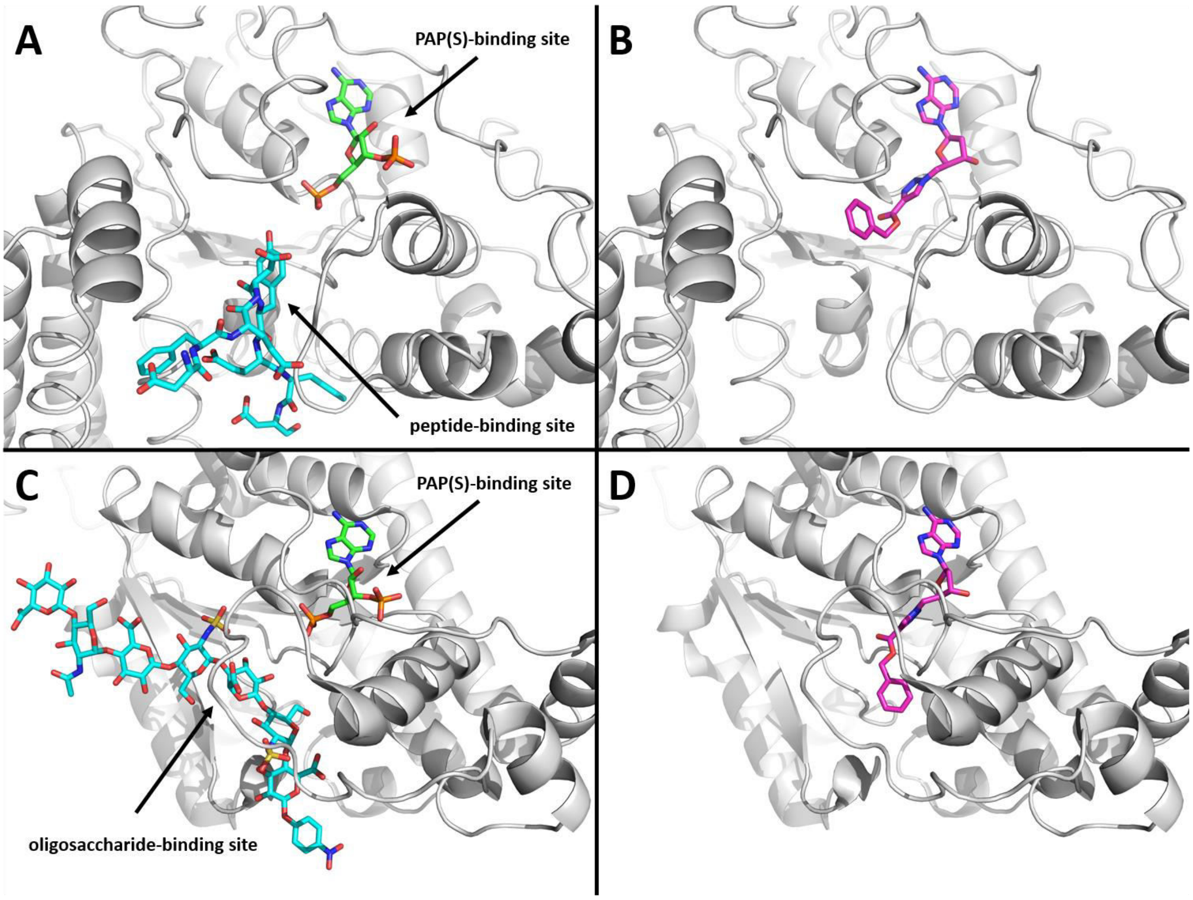

(A) PAP(S)- and peptide-binding sites of TPST1. Protein is rendered as grey cartoon. PAP is rendered as coloured sticks (carbon–green, nitrogen–blue, oxygen–red, phosphorus–orange). The CC4 substrate peptide is rendered as coloured sticks (carbon–cyan, nitrogen–blue, oxygen–red).

(B) Molecular docking results of **9** in TPST1. Protein is rendered as grey cartoon. **9** is rendered as coloured sticks (carbon–magenta, nitrogen–blue, oxygen–red).

(C) PAP(S)- and oligosaccharide-binding sites of HS2ST. Protein is rendered as grey cartoon. PAP is rendered as coloured sticks (carbon–green, nitrogen–blue, oxygen–red, phosphorus–orange). The oligosaccharide substrate is rendered as coloured sticks (carbon–cyan, nitrogen–blue, oxygen–red, sulfur–yellow).

(D) Molecular docking results of **9** in HS2ST. Protein is rendered as grey cartoon. **9** is rendered as coloured sticks (carbon–magenta, nitrogen–blue, oxygen–red).

Fig. SI 5. ¹H NMR of 18 and subsequent acetylation product

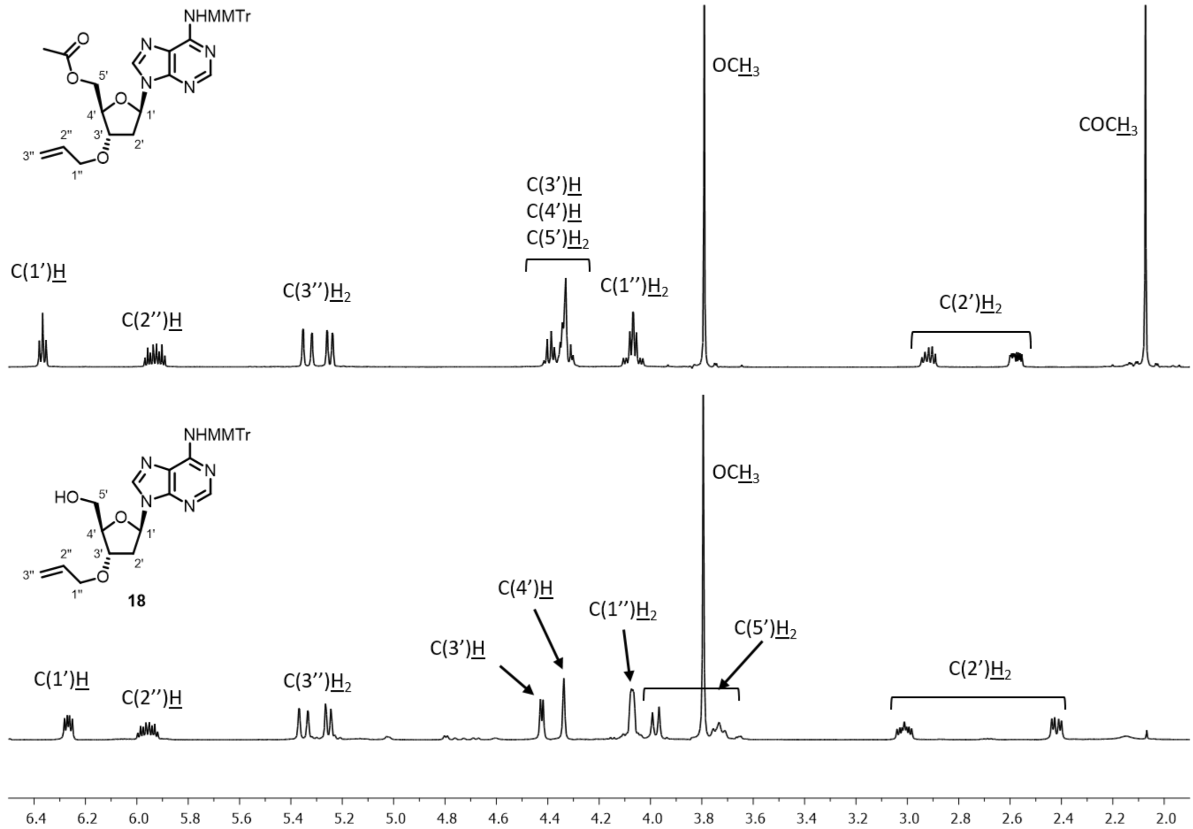

All assignments supported by COSY

Materials and Methods

Biology

Chemicals and Compounds

N-sulfated, fluorescein-tagged HS2ST1 hexasaccharide glycan substrate (GlcNS-GlcA-GlcNS-IdoA-GlcNS-GlcA-fluorescein, where S=sulfation) containing L-IdoA at the third residue from the reducing end (to which a linker and the fluorophore were conjugated) was purchased from GLYCAN therapeutics. TPST1 peptide substrate (CC4-tide, 5-FAM-EDFEDYEFDG-$CONH_2$) was synthesised using solid-phase Fmoc chemistry (Pepceuticals, Leicester, UK). All standard laboratory biochemicals, were purchased from Sigma, and were of the highest analytical quality. PAPS (adenosine 3'-phosphate 5'-phosphosulfate, lithium salt hydrate, PAP (adenosine 3',5'-diphosphate, disodium salt), CoA (coenzymeA, sodium salt), ATP (adenosine 5'-triphosphate, disodium salt hydrate) were all purchased from Sigma and stored at -80 °C to minimise degradation.

Recombinant Protein Production and SDS-PAGE

Human TPST1 (residues Lys43-Leu360) enzyme was purified as a recombinant protein containing an N-terminal 6xHis tag as previously described.[5] Chicken HS2ST (isoform 1), which exhibits ~92% identity to human HS2ST, was expressed in the Rosetta-gami (DE3) strain of *E. coli* from a modified pMAL-c2x plasmid encoding an N-terminal Maltose Binding Protein (MBP) affinity tag.[6] Glutathione-*S*-transferase (GST) tagged CC4-tide (EDFEDYEFDG) was cloned into pOPINJ (OPPF-UK) and affinity purified from BL21 (DE3) pLysS *E. coli* using Glutathione-Sepharose 4B (GE Healthcare).[5] For SDS-PAGE, proteins were denatured in Laemmli sample buffer, heated at 95 °C for 5 min and then analysed by SDS-PAGE with 10% (v/v) polyacrylamide gels. Gels were stained and de-stained using a standard Coomassie Brilliant Blue protocol. To evaluate protein sulfation by immunoblotting, standard western blotting procedures were followed using monoclonal anti-sulfotyrosine antibody clone o-1C-A2 (Millipore) generated using a phage display procedure and sulfotyrosine selection peptide antigens[7] in the presence of appropriate positive and negative controls, and modifications visualised using ECL reagent.

Microfluidics-Based Sulfation Assay

Non-radioactive microfluidic mobility shift peptide and carbohydrate sulfation assays were performed in solution with a 12-sipper chip coated with SR8 reagent and a Perkin Elmer EZ Reader II system[8] using an EDTA-based separation buffer and real-time kinetic evaluation of substrate sulfation. Pressure and voltage settings were adjusted manually to afford optimal separation of the sulfated product and non-sulfated substrate, with a sample (sip) volume of 20 nL, and total assay times appropriate for the experiment. The fluorophore tagged TPST1 peptide substrate and HS2ST1 glycan substrate where detected by the EZ Reader *via* LED-induced fluorescence.

Individual sulfation assays were assembled in a 384 well plate in a volume of 80 µL in the presence of the indicated concentration of PAPS or various test compounds, 50 mM HEPES, 0.015% (v/v) Brij-35 and 5 mM $MgCl_2$. The degree of substrate sulfation was directly calculated using EZ Reader software by measuring the sulfo-product:non-sulfated substrate ratio at each time-point. The activity of the sulfotransferase enzymes in the presence of biochemicals and small molecule inhibitors was quantified in 'kinetic mode' by monitoring the amount of sulfated product generated over the assay time, relative to control assay with no additional inhibitor molecule (DMSO or buffer control). Data was normalized with respect to these control assays, with sulfate incorporation into the substrate limited to ~ 20% to prevent depletion of PAPS and to ensure assay linearity. K_m and IC_{50} values were determined by non-linear regression analysis with GraphPad Prism software.

Chemistry

General Experimental Details

Unless stated, all materials were purchased from commercial sources (Acros, Aldrich, Alfa Aesar, Fluorochem and Carbosynth) and used without any further treatment. Anhydrous solvents were obtained by passage through drying columns supplied by BBraun Ltd. High-boiling solvents were removed from the reaction crudes employing rotary evaporators connected with high-vacuum pumps. Flash column chromatography was performed using silica gel (Aldrich 40-63 µm, 230-400 mesh).

Thin layer chromatography was performed using UV254 sensitive, silica gel coated, aluminium TLC plates purchased from Merck. Visualization was achieved by UV fluorescence or either basic $KMnO_4$ solution or acidic, ethanolic phenol and heat.

All NMR spectra were recorded on a Bruker Avance 500 MHz spectrometer in the deuterated solvent stated. Chemical shifts are reported in ppm and coupling constants (*J*) are reported in Hz. ^{1}H nuclear magnetic resonance (NMR) spectra were recorded at 500 MHz. ^{13}C NMR spectra were recorded at 126 MHz. 31P NMR spectra were recorded at 202 MHz. All ^{13}C and ^{31}P NMR spectra were proton decoupled. Chemical shifts (δ) are given in parts per million (ppm). Peaks are described as singlets (s), doublets (d), triplets (t), quartets (q), multiplets (m) and broad (br.). Coupling constants (*J*) are quoted to the nearest 0.5 Hz. All assignments of NMR spectra are based on 2D NMR data (COSY).

Mass spectra were recorded using a Micromass LCT Mass Spectrometer in the ES ionisation mode. Samples were injected using a direct infusion syringe pump.

The numbering of compound structures does not necessarily reflect the numbering contained in the systematic names.

Experimental Procedures and Data

General Procedure A: Phosphitylation and Oxidation

The stated nucleoside (1.0 eq.) was dried by azeotrope with anhydrous pyridine three times and was then dissolved in anhydrous MeCN (20 mL/mmol) and cooled to 0 °C. The stated phosphoramidite (1.15 eq. per hydroxyl) was added followed by 5-(ethylthio)-1*H*-tetrazole (0.25 M in MeCN, 1.73 eq. per hydroxyl (1.5 eq. w.r.t. phosphoramidite)) and the reaction mixture was allowed to warm to room temperature. A white precipitate rapidly formed. The reaction mixture was stirred at room temperature until TLC showed complete consumption of starting material. A solution of mCPBA (1.15 eq. per hydroxyl) in MeCN was added at 0 °C and the reaction mixture was allowed to warm to room temperature. Upon complete consumption of starting material (TLC) the reaction mixture was diluted with EtOAc and washed with 1 M aq. $Na_2S_2O_3$, sat. aq. $NaHCO_3$ and brine, then dried over $MgSO_4$ and concentrated *in vacuo*. The residue was then purified as stated.

General Procedure B: Hydrogenative Debenzylation

The stated nucleoside (1.0 eq.) was dissolved in MeOH (10–20 mL/mmol). Et_3N (3.0 eq. per phosphate / benzyl ester) was added followed by 10% Pd/C (30 mol% per phosphate / benzyl ester). The reaction mixture was evacuated and back-filled with H_2 three times and stirred under and atmosphere of H_2 for 48 hours. The catalyst was removed by filtration through a pad of Celite® and the filtrate was concentrated *in vacuo*. The residue was dissolved in H_2O (10 mL/mmol) and Na^+-Dowex® (500 mg/100 mg nucleoside) was added, and the mixture was stirred at room temperature overnight then filtered and lyophilised to give the desired compound.

General Procedure C: Monomethoxytrityl Deprotection

The stated nucleoside (1.0 eq.) was dissolved in 80% v/v aq. AcOH (approx. 20 mL/mmol) and stirred at room temperature until TLC showed complete consumption of starting material (typically 1 hour). The reaction was quenched be addition of MeOH and AcOH was removed by azeotrope with water. The resulting aqueous solution was washed twice with Et_2O and concentrated *in vacuo*. The residue was then purified as stated.

General Procedure D: Mono Debenzylation

The stated nucleoside (1.0 eq.) was dissolved in anhydrous MeCN (20 mL/mmol). NaI (2.5 eq.) and heated to 80 °C until TLC showed complete consumption of starting material (typically 2-4 hours). The reaction mixture was concentrated *in vacuo* and purified as stated.

General Procedure E: Click Coupling

2'-Deoxy-5'-azidoadenosine (1.0 eq.) and the stated alkyne (1.5 eq.) were dissolved in a 1:1 mixture of ^{t}BuOH:H_2O (10 mL/mmol). 0.5 M aq. $CuSO_4$ (10 mol%) and 0.5 M aq. sodium ascorbate (20 mol%) were sequentially added and the reaction mixture was stirred at room temperature for 16 hours. The reaction mixture was concentrated *in vacuo*. The residue was then purified as stated.

General Procedure F: Bis-cyanoethylphosphate Deprotection

The stated nucleoside (1.0 eq.) was dissolved in anhydrous MeCN (10 - 20 mL/mmol) to which was then added *N,N,N',N'*-tetramethylguanidine (5.0 eq.) and trimethylsilyl chloride (4 eq.). The reaction mixture was stirred at room temperature for 16 hours. The reaction mixture was concentrated *in vacuo* and the residue was dissolved in MeOH (*ca.* 10 mL/mmol). Saturated methanolic ammonia (*ca.* 1 mL) was added and the solution was stirred for 5 min then concentrated *in vacuo*. The residue was dissolved in MeOH and the product was precipitated by the addition of acetone then collected by filtration. The solid was dissolved in H_2O (10 mL/mmol) and Na^+-Dowex® (500 mg/100 mg nucleoside) was added, and the mixture was stirred at room temperature overnight then filtered and lyophilised to give the desired compound.

2'-Deoxy-3′-phosphoadenosine 5′-phosphate (2-Deoxy-PAP)

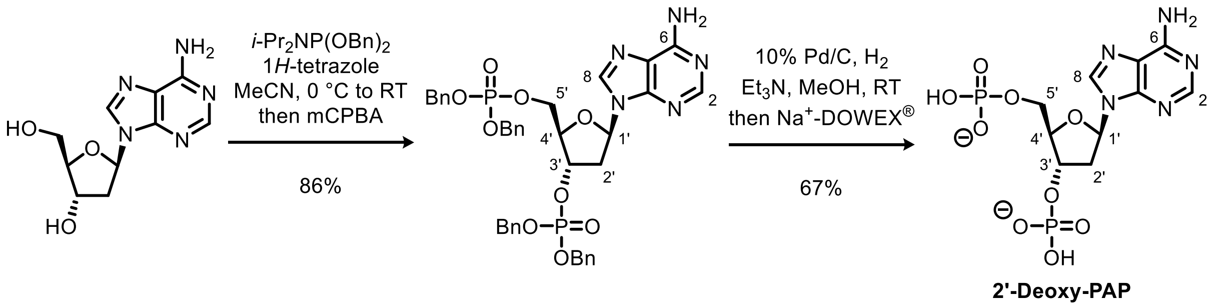

2'-Deoxyadenosine (538 mg, 2.00 mmol) was subjected to General Procedure A using dibenzyl *N,N*-diisopropylphosphoramidite. Purification by flash column (SiO_2; gradient elution, CH_2Cl_2:MeOH; 98:2 to 95:5) gave the product as a colourless oil (1.33 g, 86%). R_f = 0.25 (CH_2Cl_2:MeOH; 95:5).^{1}H (500 MHz, MeOD) δ 8.14 (1H, s, C(2)H or C(8)H), 8.13 (1H, s, C(2)H or C(8)H), 7.38 – 7.20 (20H, m, 20 x ArH), 6.31 (1H, ~t, *J* 7.0, C(1')H), 5.19 – 5.13 (1H, m, C(3')H), 5.12 – 5.03 (4H, m, 2 x benzylic CH$_2$), 4.96 – 4.88 (4H, m, 2 x benzylic CH$_2$), 4.26 – 4.21 (1H, m, C(4')H), 4.21 – 4.15 (1H, m, one of C(5')H$_2$), 4.15 – 4.08 (1H, m, one of C(5')H$_2$), 2.89 – 2.80 (1H, m, one of C(2')H$_2$), 2.54 (1H, ddd, *J* 14.0, 6.5, 3.0, one of C(2')H$_2$).^{13}C NMR (126 MHz, MeOD) δ 157.3, 153.9, 150.3, 141.0, 136.9 – 136.8 (m, 4 x q), 129.9 – 129.1 (m, 20 x ArCH), 120.7, 85.6, 84.7 – 84.6 (m), 78.9 (d, *J* 5.5), 71.2 (d, *J* 60.0),

70.9 (d, *J* 4.5), 67.6 (d, *J* 5.5), 38.4 (d, *J* 4.0). ^{31}P NMR (202 MHz, MeOD) δ -1.52, -2.28.HRMS: (ESI+) Calculated for $C_{38}H_{40}N_5O_9P_2$: 772.2307. Found $[M+H]^+$: 772.2321 (1.85 ppm).

The benzyl-protected 3',5'-bisphosphate (1.20 g, 1.56 mmol) was subjected to General Procedure B. The product was obtained as a white solid (474 mg, 67%). ^{1}H NMR (500 MHz, D_2O) δ 8.39 (1H, s, C(2)H or C(8)H), 8.03 (1H, s, C(2)H or C(8)H), 6.37 (1H, t, *J* 6.9, C(1')H), 4.87 – 4.82 (1H, m, C(3')H), 4.36 – 4.32 (1H, m, C(4')H), 4.03 – 3.83 (2H, m, C(5')H_2), 2.76 – 2.66 (2H, m, C(2')H_2). ^{13}C NMR (126 MHz, D_2O) δ 155.2, 152.4, 148.5, 140.0, 118.3, 85.7 (dd, *J* 8.1, 6.0), 83.67, 74.58 (d, *J* 4.5), 64.3 (d, *J* 4.6), 38.81. ^{31}P NMR (202 MHz, D_2O) δ 2.36, 2.03. HRMS: (ESI+) Calculated for $C_{10}H_{16}N_5O_9P_2$: 412.0429. Found $[M+H]^+$: 412.0432 (0.79 ppm).

The spectroscopic properties were consistent with the data available in the literature.[9]

N-Monomethoxytrityl-2'-deoxyadenosine (15)

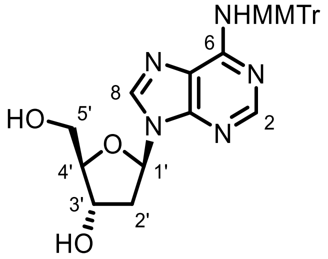

15

2'-Deoxyadenosine monohydrate (5.00 g, 18.6 mmol) was dried by evaporation from anhydrous pyridine three times. The dried nucleoside was dissolved in anhydrous pyridine (93 mL). The reaction mixture was cooled to 0 °C and trimethylsilyl chloride (10.8 mL, 85.4 mmol) was added dropwise. The reaction mixture was allowed to warm to room temperature and stirred for 1 hour. The reaction mixture was cooled to 0 °C and 4-Methoxytriphenylmethyl chloride (11.5 g, 37.1 mmol) was added in several portions. The reaction mixture was allowed to warm to room temperature and stirred for 7 days. The reaction mixture was cooled to 0 °C and water (45 mL) was added followed by 28% aq. NH_3 (4.5 mL). The reaction mixture was allowed to warm to room temperature then concentrated *in vacuo*. The residue was partitioned between water and CH_2Cl_2. The organic layer was washed with water three times then dried over $MgSO_4$ and concentrated *in vacuo*. Purification by flash column chromatography (SiO_2; gradient elution, CH_2Cl_2:MeOH; 99:1 to 9:1) gave the title compound as a colourless foam (9.30 g, 96%). R_f = 0.20 (CH_2Cl_2:MeOH; 9:1). ^{1}H NMR (500 MHz, $CDCl_3$) δ 8.01 (1H, s, C(2)H or C(8)H), 7.80 (1H, s, C(2)H or C(8)H), 7.37 – 7.22 (12H, m ,12 x ArH), 7.07 (1H, s, NH), 6.85 – 6.78 (2H, m, 2 x ArH), 6.67 (1H, d, *J* 11.5, C(5')OH)), 6.31 (1H, dd, *J* 9.0, 5.5, C(1')H), 4.79 (1H, br s, C(3')H), 4.21 (1H, s, C(4')H), 3.95 (1H, d, *J* 13.0, one of C(5')H_2), 3.80 (3H, s, OCH_3), 3.76 (1H, ~t, *J* 12.5, one of C(5')H_2), 3.14 – 3.06 (1H, m, one of C(2')H_2), 2.29 (1H, dd, *J* 13.5, 5.5, one of C(2')H_2) 2.20 (1H, br s, C(3')OH). ^{13}C NMR (126 MHz, $CDCl_3$) δ 158.4, 154.6, 151.6, 147.4, 145.00, 144.97, 139.6, 137.1, 130.3, 129.0, 128.0, 127.0, 122.4, 113.3, 89.8, 87.9, 73.3, 71.2, 63.5, 55.3, 40.9. HRMS: (ESI+) Calculated for $C_{30}H_{30}N_5O_4$: 524.2292. Found $[M+H]^+$: 524.2287 (-0.95 ppm).

***N*-Monomethoxytrityl-2'-deoxy-3'-allyloxy-5'-allyloxyadenosine (16)**

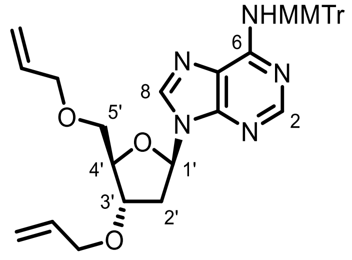

16

15 (3.30 g, 6.31 mmol) and tetrabutylammonium iodide (466 mg, 1.26 mmol) was dissolved in DMF (32 mL) and cooled to -20 °C. NaHMDS (1 M in THF, 6.94 mL, 6.94 mmol) was added dropwise and the solution was allowed to warm to 0 °C over 30 minutes then cooled to -20 °C. Allyl bromide (600 µL, 6.94 mmol) was added and the solution was allowed to warm to 0 °C and stirred for 1 hour then cooled to -20 °C. NaHMDS (1 M in THF, 6.94 mL, 6.94 mmol) was added dropwise and the solution was allowed to warm to 0 °C over 30 minutes then cooled to -20 °C. Allyl bromide (600 µL, 6.94 mmol) was added and the solution was allowed to warm to 0 °C and stirred for 2 hours. The reaction mixture was partitioned between water (250 mL) and EtOAc (75 mL). The aqueous layer was extracted with EtOAc (3 x 75 mL). The combined organics were washed with water (250 mL) and brine (250 mL) then dried over $MgSO_4$ and concentrated *in vacuo*. Purification by flash column chromatography (SiO_2; gradient elution, Hexane:EtOAc; 9:1 to 2:1) gave the title compound as a pale yellow foam (2.30 g, 60%). R_f = 0.25 (Hexane:EtOAc; 1:1). ^{1}H NMR (500 MHz, $CDCl_3$) δ 8.15 (1H, s, C(2)H or C(8)H), 8.08 (1H, s, C(2)H or C(8)H), 7.39 – 7.22 (12H, m, 12 x ArH), 6.95 (1H, s, NH), 6.83 – 6.79 (2H, m, 2 x ArH), 6.47 (1H, ~t, *J* 6.5, C(1')H), 5.98 – 5.88 (2H, m, 2 x CH=CH_2), 5.36 – 5.21 (4H, m, 2 x CH=CH_2), 4.36 – 4.32 (1H, m, C(3')H), 4.31 – 4.28 (1H, m, C(4')H), 4.10 – 4.01 (4H, m, 2 x allylic CH_2), 3.79 (3H, s, OCH_3), 3.73 (1H, dd, *J* 10.5, 3.5, one of C(5')H_2), 3.67 (1H, dd, *J* 10.5, 3.5, one of C(5')H_2), 2.74 (1H, ddd, *J* 13.5, 7.0, 6.0, one of C(2')H_2), 2.58 (1 H, ddd, *J* 13.5, 6.0, 3.0, one of C(2')H_2). ^{13}C NMR (126 MHz, $CDCl_3$) δ 158.2, 154.0, 152.1, 148.5, 145.2, 138.5, 137.2, 134.12, 134.11, 130.1, 128.8, 127.8, 126.7, 121.1, 117.5, 117.3, 113.1, 84.4, 83.9, 79.2, 72.3, 70.9, 70.23, 70.19, 55.1, 38.1. HRMS: (ESI+) Calculated for $C_{36}H_{38}N_5O_4$: 604.2918. Found $[M+H]^+$: 604.2925 (1.16 ppm).

***N*-Monomethoxytrityl-2'-deoxy-3'-(2''-methoxy-2''-oxoethoxy)-5'-(2'''-methoxy-2'''-oxoethoxy)-adenosine (17)**

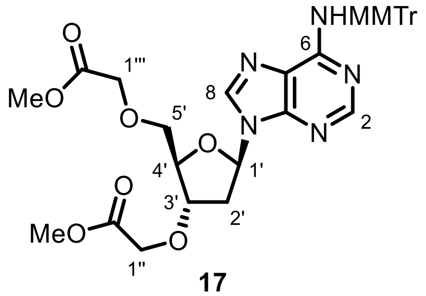

17

16 (1.00 g, 1.66 mmol) was dissolved in CH_2Cl_2 (42 mL). NaOH (2.5 M in MeOH, 13 mL) was added and the solution was cooled to -78 °C. Ozone was bubbled through the reaction mixture which rapidly became orange. Over the course of 2 hours at -78 °C, the colour faded to pale yellow and a white precipitate formed. Nitrogen was bubbled through the reaction mixture for 10 minutes then warmed to room temperature. Water (50 mL) was added and the mixture was carefully acidified with 2 M aq. HCl. The product was extracted with CH_2Cl_2 (3 x 50 mL). The combined organics were dried over $MgSO_4$

and concentrated *in vacuo*. Purification by flash column chromatography (SiO_2; Hexane:EtOAc; 2:1) gave the title compound as a pale yellow oil (597 mg, 54%). R_f = 0.18 (Hexane:EtOAc; 1:1). ^{1}H (500 MHz, $CDCl_3$) δ 8.28 (1H, s, C(2)H or C(8)H), 8.05 (1H, s, C(2)H or C(8)H), 7.37 – 7.21 (12H, m, 12 x ArH), 6.95 (1H, s, NH), 6.80 (2H, d, *J* 8.5, 2 x ArH), 6.47 (1H, ~t, *J* 7.0, C(1')H), 4.52 – 4.50 (1H, m, C(3')H), 4.37 – 4.35 (1H, m, C(4')H), 4.19 (2H, ABq, J_{AB} 16.5, C(1'')H_2 or C(1''')H_2), 4.15 (2H, ABq, J_{AB} 16.5, C(1'')H_2 or C(1''')H_2), 3.84 (1H, dd, *J* 10.0, 3.5, one of C(5')H_2), 3.81 (1H, dd, *J* 10.0, 3.5, one of C(5')H_2), 3.78 (3H, s, OCH_3), 3.772 (3H, s, OCH_3), 3.766 (3H, s, OCH_3), 3.10 (1H, ddd, *J* 13.5, 7.0, 6.0, one of C(2')H_2), 2.62 (1H, ddd, *J* 13.5, 6.0, 3.0, one of C(2')H_2). ^{13}C NMR (126 MHz, $CDCl_3$) δ 170.5, 170.4, 158.3, 154.1, 152.2, 148.6, 145.3, 138.9, 137.3, 130.3, 129.0, 127.9, 126.9, 121.2, 113.1, 84.5, 83.8, 80.9, 71.4, 71.0, 68.4, 66.8, 55.3, 52.03, 51.98, 38.0. HRMS: (ESI$^+$) Calculated for $C_{36}H_{38}N_5O_8$: 668.2715. Found $[M+H]^+$: 668.2722 (-1.04 ppm).

2'-Deoxy-3'-(2''-methoxy-2''-oxoethoxy)-5'-(2'''-methoxy-2'''-oxoethoxy)-adenosine (1)

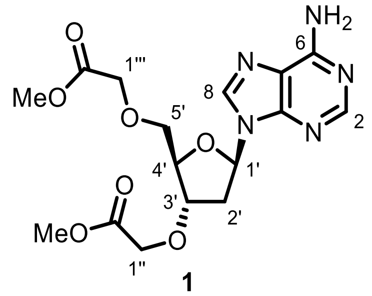

1

17 (100 mg, 0.15 mmol) was subjected to General Procedure C. Purification by flash column chromatography (SiO_2; gradient elution CH_2Cl_2:MeOH; 99:1 to 9:1) gave the title compound as a colourless foam (59 mg, 100%). R_f = 0.25 (CH_2Cl_2:MeOH; 9:1). ^{1}H (500 MHz, $CDCl_3$) δ 8.33 (1H, s, C(2)H or C(8)H), 8.27 (1H, s, C(2)H or C(8)H), 6.47 (1H, ~t, *J* 7.0, C(1')H), 6.46 (2H, br s, NH_2), 4.49 – 4.46 (1H, m, C(3')H), 4.33 – 4.32 (1H, m, C(4')H), 4.16 (2H, ABq, J_{AB} 16.5, C(1'')H_2 or C(1''')H_2), 4.12 (2H, ABq, J_{AB} 17.0, C(1'')H_2 or C(1''')H_2), 3.80 (1H, dd, *J* 10.5, 3.5, one of C(5')H_2), 3.76 (1H, dd, *J* 10.0, 3.0, one of C(5')H_2), 3.75 (3H, s, CO_2CH_3), 3.74 (3H, s, CO_2CH_3), 2.72 (1H, ~dt, *J* 13.5, 6.5, one of C(2')H_2), 2.61 (1H, ddd, *J* 13.5, 6.0, 3.0, one of C(2')H_2). ^{13}C NMR (126 MHz, $CDCl_3$) δ 170.53, 170.46, 155.7, 152.8, 149.6, 139.4, 119.7, 84.5, 83.9, 80.9, 71.4, 68.4, 66.8, 52.07, 52.05, 38.3. HRMS: (ESI$^+$) Calculated for $C_{16}H_{22}N_5O_7$: 396.1514. Found $[M+H]^+$: 396.1503 (2.72 ppm).

2'-Deoxy-3'-(2''-amino-2''-oxoethoxy)-5'-(2'''-amino-2'''-oxoethoxy)-adenosine (2)

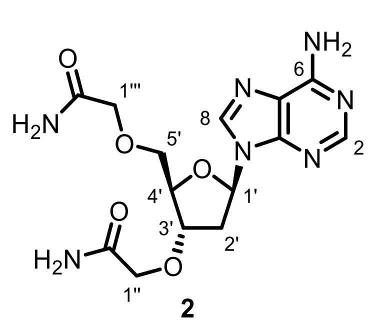

2

17 (127 mg, 0.19 mmol) was dissolved in 6 M NH_3 in MeOH (5 mL) and stirred at room temperature overnight. The reaction mixture was then concentrated *in vacuo* and subjected to General Procedure C. Trituration of the residue with Et_2O gave the title compound as a colourless foam (58 mg, 85% over 2 steps). R_f = 0.1 (CH_2Cl_2:MeOH; 9:1). ^{1}H (500 MHz, MeOD) δ 8.35 (1H, s, C(2)H or C(8)H), 8.22 (1H,

s, C(2)H or C(8)H), 6.47 (1H, dd, *J* 8.0, 6.0, C(1')H), 4.54 – 4.47 (1H, m , C(3')H), 4.40 – 4.34 (1H, m, C(4')H), 4.10 (2H, ABq, J_{AB} 15.5, C(1")H_2 or C(1"')H_2), 4.03 (2 H, s, C(1")H_2 or C(1"')H_2), 3.82 (2H, d, *J* 4.5, C(5')H_2), 2.96 (1 H, ddd, *J* 14.0 8.0, 6.0, one of C(2')H_2), 2.67 (1 H, ddd, *J* 14.0, 6.0, 2.5, one of C(2')H_2). ^{13}C NMR (126 MHz, MeOD) δ 175.2, 175.1, 157.4, 153.9, 150.5, 141.2, 85.9, 84.9, 82.0, 72.7, 71.3, 69.3, 37.7. HRMS: (ESI$^+$) Calculated for $C_{14}H_{19}N_7NaO_5$: 388.1339. Found [M+Na]$^+$: 388.1339 (0.29 ppm).

2'-Deoxy-3'-(2"-hydroxyamino-2"-oxoethoxy)-5'-(2"'-hydroxyamino-2"'-oxoethoxy)-adenosine (3)

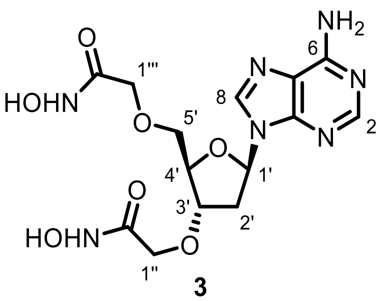

3

17 (215 mg, 0.32 mmol) was dissolved in MeOH (5 mL). 50% wt. aq. NH_2OH (0.80 mL, 12.8 mmol) was added and the reaction mixture was stirred at room temperature overnight. The reaction mixture was then concentrated *in vacuo* and subjected to General Procedure C. Trituration of the residue with Et_2O gave the title compound as a colourless foam (32 mg, 25% over 2 steps). R_f = 0.2 (CH_2Cl_2:MeOH; 4:1). ^{1}H (500 MHz, DMSO) δ 8.36 (1H, s, C(2)H or C(8)H), 8.15 (1H, s, C(2)H or C(8)H), 7.36 – 7.19 (4H, m, NH_2, 2 x NH and 2 x OH), 6.36 (1H, dd, *J* 8.0, 6.5, C(1')H), 4.40 – 4.33 (1H, m, C(4')H), 4.29 – 4.23 (1H, m), 3.93 (2H, s, C(1")H_2 or C(1"')H_2), 3.86 (2H, s, C(1")H_2 or C(1"')H_2), 3.71 – 3.60 (2H, m, C(5')H_2), 2.91 (1H, ddd, *J* 13.5, 8.0, 6.0, one of C(2')H_2), 2.54 (1H, ~dd, *J* 13.5, 5.5, one of C(2')H_2). ^{13}C NMR (126 MHz, DMSO) δ 171.34, 171.28, 156.1, 156.0, 152.7, 149.3, 139.6, 119.2, 83.5, 82.6, 80.4, 71.1, 70.1, 68.0, 35.7. HRMS: (ESI$^+$) Calculated for $C_{14}H_{20}N_7O_7$: 398.1430. Found [M+H]$^+$: 398.1432 (0.58 ppm).

2'-Deoxy-3'-(carboxymethoxy)-5'-(carboxymethoxy)-adenosine (4)

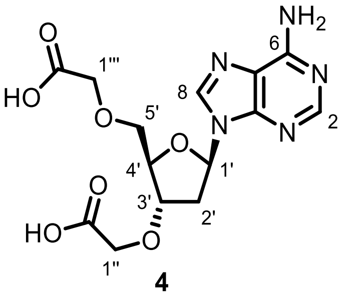

4

17 (317 mg, 0.48 mmol) was dissolved in MeOH (4 mL). 1 M aq. NaOH (1.40 mL, 1.40 mmol) was added and the reaction mixture was stirred at room temperature overnight. The reaction mixture was then concentrated *in vacuo* and the residue was dissolved in water (5 mL). The pH was carefully adjusted to *ca*. 2 by addition of 1 M aq. HCl. The product was extracted with EtOAc (3 x 10 mL). The combined organics were dried over $MgSO_4$ and concentrated *in vacuo*. The residue was subjected to General Procedure C. Trituration of the residue with Et_2O gave the title compound as a white solid (122 mg, 70% over 2 steps). R_f = 0.1 (CH_2Cl_2:MeOH + 1% AcOH; 3:1).^{1}H (500 MHz, DMSO) δ 12.76 (2H,

br s, 2 x CO_2H), 8.42 (1H, s, C(2)H or C(8)H), 8.15 (1H, s, C(2)H or C(8)H), 7.26 (2H, br s, NH_2), 6.33 (1H, dd, *J* 8.0, 6.0, C(1')H), 4.41 – 4.35 (1H, m, C(3')H), 4.19 – 4.22 (1H, m, C(4')H), 4.15 (2H, s, C(1'')H_2 or C(1''')H_2), 4.06 (2H, s, C(1'')H_2 or C(1''')H_2), 3.71 (1H, dd, *J* 10.5, 4.5, one of C(5')H_2), 3.65 (1H, dd, *J* 10.5, 4.5, one of C(5')H_2), 2.82 (1H, ddd, *J* 13.5, 8.0, 5.5, one of C(2')H_2), 2.54 (1H, ddd, *J* 13.5, 6.0, 2.0, one of C(2')H_2). ^{13}C NMR (126 MHz, DMSO) δ 171.6 (2 x q), 156.0, 152.7, 149.3, 139.3, 119.0, 83.3, 83.1, 80.5, 71.0, 67. 9, 65.9, 36.3. HRMS: (ESI$^+$) Calculated for $C_{14}H_{18}N_5O_7$: 368.1212. Found $[M+H]^+$: 368.1211 (-0.19 ppm).

***N*-Monomethoxytrityl-2'-deoxy-3'-allyloxyadenosine (18)**

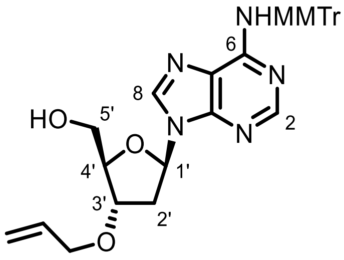

18

15 (3.00 g, 5.74 mmol) and tetrabutylammonium iodide (211 mg, 0.57 mmol) was dissolved in DMF (29 mL) and cooled to -20 °C. NaHMDS (1 M in THF, 6.31 mL, 6.31 mmol) was added dropwise and the solution was allowed to warm to 0 °C over 30 minutes then cooled to -40 °C. Allyl bromide (550 µL, 6.31 mmol) was added and the solution was allowed to warm to 0 °C and stirred for 2 hours. The reaction mixture was partitioned between water (250 mL) and EtOAc (75 mL). The aqueous layer was extracted with EtOAc (3 x 75 mL). The combined organics were washed with water (250 mL) and brine (250 mL) then dried over $MgSO_4$ and concentrated *in vacuo*. Purification by flash column chromatography (SiO_2; gradient elution, Hexane:EtOAc; 5:1 to 1:2) gave the title compound as a pale yellow foam (1.30 g, 40% (75% brsm)). R_f = 0.2 (Hexane:EtOAc; 1:2). ^{1}H (500 MHz, $CDCl_3$) δ 8.03 (1H, s, C(2)H or C(8)H), 7.80 (1H, s, C(2)H or C(8)H), 7.38 – 7.24 (12H, m, 12 x ArH), 7.09 (1H, s, NH), 6.82 (2H, d, *J* 9.0, 2 x ArH), 6.69 (1H, d, *J* 11.0, C(5')OH), 6.27 (1H, dd, *J* 10.0, 5.5, C(1')H), 5.96 (1H, ddt, *J* 17.0, 10.5, 5.5, CH=CH_2), 5.38 – 5.32 (1H, m, one of CH=CH_2), 5.28 – 5.23 (1H, m, one of CH=CH_2), 4.42 (1H, d, *J* 5.0, C(3')H), 4.34 (1H, s, C(4')H), 4.12 – 4.03 (2H, m, allylic CH_2), 3.98 (1H, d, *J* 12.5, one of C(5')H_2), 3.79 (3H, s, OCH_3), 3.73 (1H, m, one of C(5')H_2), 3.01 (1H, ddd, *J* 14.0, 10.0, 5.0, one of C(2')H_2), 2.42 (1H, dd, *J* 14.0, 5.5, one of C(2')H_2). ^{13}C NMR (126 MHz, $CDCl_3$) δ 158.4, 154.7, 151.7, 147.4, 145.1, 145.0, 139.7, 137.0, 134.2, 130.2, 128.9, 128.0, 127.0, 122.6, 117.5, 113.3, 88.1, 87.2, 80.6, 71.2, 70.1, 63.9, 55.3, 38.0. HRMS: (ESI+) Calculated for $C_{33}H_{34}N_5O_4$: 564.2605. Found $[M+H]^+$: 564.2601 (-0.71 ppm).

N-Monomethoxytrityl-2'-deoxy-3'-(2''-methoxy-2''-oxoethoxy)-adenosine (19)

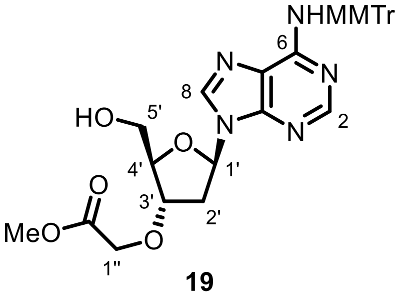

19

18 (1.30 g, 2.31 mmol) was dissolved in CH_2Cl_2 (46 mL). NaOH (2.5 M in MeOH, 9.20 mL) was added and the solution was cooled to -78 °C. Ozone was bubbled through the reaction mixture which rapidly became orange. Over the course of 2 hours at -78 °C, the colour faded to pale yellow and a white precipitate formed. Nitrogen was bubbled through the reaction mixture for 10 minutes then warmed to room temperature. Water (50 mL) was added and the mixture was carefully acidified with 2 M aq. HCl. The product was extracted with CH_2Cl_2 (3 x 50 mL). The combined organics were dried over $MgSO_4$ and concentrated *in vacuo*. Purification by flash column chromatography (SiO_2; gradient elution, Hexane:EtOAc; 1:1 to 1:2) gave the title compound as a pale yellow oil (851 mg, 62%). R_f = 0.15 (Hexane:EtOAc; 1:2). ^{1}H (500 MHz, $CDCl_3$) δ 8.02 (1H, s, C(2)H or C(8)H), 7.82 (1H, s, C(2)H or C(8)H), 7.41 – 7.19 (12H, m, 12 x ArH), 7.11 (1H, s, NH), 6.81 (2H, d, *J* 9.0, 2 x ArH), 6.66 (1H, br s, C(5')OH), 6.29 (1H, dd, *J* 9.5, 5.5, C(1')H), 4.45 (1H, d, *J* 5.0, C(3')H), 4.38 (1H, s, C(4')H), 4.18 (2H, ABq, J_{AB} 16.0, C(1'')H_2), 3.96 (1H, d, *J* 12.5, one of C(5')H_2), 3.79 – 3.73 (7H, m, OCH_3, CO_2CH_3 and one of C(5')H_2), 3.01 (1H, ddd, J 13.5, 9.5, 5.0, one of C(2')H_2), 2.46 (1H, dd, *J* 13.5, 5.5, one of C(2')H_2). ^{13}C NMR (126 MHz, $CDCl_3$) δ 170.4, 158.3, 154.5, 151.5, 147.4, 145.0, 144.9, 139.7, 137.0, 130.1, 128.8, 127.9, 126.9, 122.5, 113.2, 87.8, 87.0, 82.3, 71.1, 66.6, 63.7, 55.2, 52.0, 37.7. HRMS: (ESI+) Calculated for $C_{33}H_{34}N_5O_6$: 596.2504. Found $[M+H]^+$: 596.2510 (1.00 ppm).

N-Monomethoxytrityl-2'-deoxy-3'-(2''-methoxy-2''-oxoethoxy)-adenosine-5'-dibenzylphosphate (20)

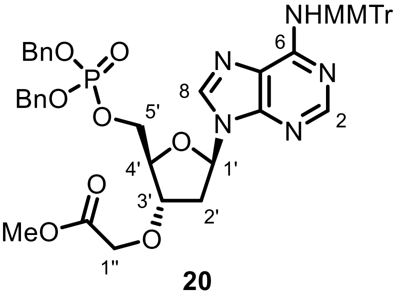

20

19 (800 mg, 1.34 mmol) was subjected to General Procedure B using dibenzyl *N*,*N*-diisopropylphosphoramidite. Purification by flash column chromatography (SiO_2; gradient elution, CH_2Cl_2:MeOH; 99:1 to 95:5) gave the title compound as a colourless oil (862 mg, 75%). R_f = 0.2 (CH_2Cl_2:MeOH; 95:5). ^{1}H (500 MHz, $CDCl_3$) δ 8.04 (1H, s, C(2)H or C(8)H), 7.96 (1H, s, C(2)H or C(8)H), 7.40 – 7.22 (22H, m, 22 x ArH), 6.95 (1H, s, NH), 6.83 – 6.79 (2H, m, 2 x ArH), 6.36 (1H, dd, *J* 7.5, 6.0, C(1')H), 5.10 – 4.98 (4H, m, 2 x benzylic CH_2), 4.33 – 4.27 (2H, m, C(3')H and C(4')H), 4.27 – 4.16 (2H, m, C(5')H_2), 4.10 (2H, ABq, J_{AB} 16.5, C(1'')H_2), 3.79 (3H, s, OCH_3), 3.76 (3H, s, CO_2CH_3), 2.75 (1H, ddd, *J* 13.5, 7.5, 6.0, one of C(2')H_2), 2.54 (1H, ddd, *J* 13.5, 6.0, 2.5, one of C(2')H_2). ^{13}C NMR (126 MHz, $CDCl_3$) δ 170.3, 158.4, 154.2, 152.4, 148.6, 145.3, 138.6, 137.3, 135.71 (d, *J* 2.7), 135.66 (d, *J* 2.8), 130.3, 129.0, 128.8, 128.7, 128.2, 128.0, 127.0, 121.5, 113.2, 84.8, 83.0 (d, *J* 8.1), 80.6, 71.1, 69.67 (d, *J* 5.4),

69.66 (d, *J* 5.4), 66.9, 66.7 (d, *J* 5.7), 55.3, 52.1, 37.0. ^{31}P NMR (126 MHz, $CDCl_3$) δ -0.78. HRMS: (ESI$^+$) Calculated for $C_{47}H_{47}N_5O_9P$: 856.3106. Found $[M+H]^+$: 856.3116 (-1.23 ppm).

2'-Deoxy-3'-(2''-methoxy-2''-oxoethoxy)-adenosine-5'-phosphate (5)

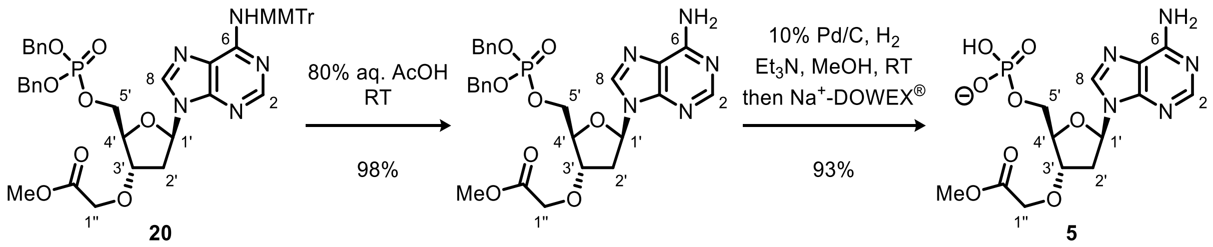

20 (400 mg, 0.47 mmol) was subjected to General Procedure C. The residue was purified by flash column chromatography (SiO_2; gradient elution, CH_2Cl_2:MeOH; 95:1 to 9:1) to give the MMTr-deprotected nucleoside as a colourless oil (267 mg, 98%). R_f = 0.15 (CH_2Cl_2:MeOH; 9:1). ^{1}H (500 MHz, $CDCl_3$) δ 8.30 (1H, s, C(2)<u>H</u> or C(8)<u>H</u>), 8.02 (1H, s, C(2)<u>H</u> or C(8)<u>H</u>), 7.34 – 7.29 (10H, m, 10 x Ar<u>H</u>), 6.39 (1H, dd, *J* 7.5, 6.0, C(1')<u>H</u>), 6.26 (2H, br. s, N<u>H</u>$_2$), 5.09 – 4.98 (4H, m, 2 x benzylic C<u>H</u>$_2$), 4.33 – 4.27 (2H, m, C(3')<u>H</u> and C(4')<u>H</u>), 4.26 – 4.16 (2H, m, C(5')<u>H</u>$_2$), 4.10 (2H, ABq, J_{AB} 16.5, C(1'')<u>H</u>$_2$), 3.75 (3H, s, CO_2C<u>H</u>$_3$), 2.71 (1H, ddd, *J* 13.5, 7.5, 5.5, one of C(2')<u>H</u>$_2$), 2.56 (1H, ddd, *J* 13.5, 6.0, 2.5, one of C(2')<u>H</u>$_2$). ^{13}C NMR (126 MHz, $CDCl_3$) δ 170.3, 155.8, 153.1, 149.6, 139.0, 135.6 (d, *J* 5.5), 128.8, 128.7, 128.7, 128.11, 128.09, 120.1, 84.7, 83.0 (d, *J* 8.0), 80.5, 69.7 – 69.6 (m, 2 x benzylic <u>C</u>H$_2$), 66.9, 66.8 (d, J 6.0), 52.1, 37.2. ^{31}P NMR (202 MHz, CDC13) δ -0.88. HRMS: (ESI+) Calculated for $C_{27}H_{30}N_5NaO_8P$: 606.1735. Found $[M+Na]^+$: 606.1740 (0.80 ppm).

The MMTr-deprotected nucleoside (267 mg, 0.46 mmol) subjected to General Procedure B. The title compound was obtained as a white solid (181 mg, 93%). ^{1}H (500 MHz, D_2O) δ 8.34 (1H, s, C(2)<u>H</u> or C(8)<u>H</u>), 8.00 (1H, s, C(2)<u>H</u> or C(8)<u>H</u>), 6.31 (1H, ~t, *J* 7.0, C(1')<u>H</u>), 4.52 – 4.43 (1H, m, C(3')<u>H</u>), 4.37 (1H, s, C(4')<u>H</u>), 4.30 (2H, s, C(1'')<u>H</u>$_2$), 3.95 – 3.85 (2H, m, C(5')<u>H</u>$_2$), 3.73 (3H, s, CO_2C<u>H</u>$_3$), 2.75 – 2.60 (2H, m, C(2')<u>H</u>$_2$). ^{13}C NMR (126 MHz, D_2O) δ 172.9, 155.2, 152.4, 148.4, 139.9, 118.2, 84.3 (d, *J* 9.0), 83.8, 81.3, 66.4, 64.5 (d, *J* 4.5), 52.6, 36.9. ^{31}P NMR (202 MHz, D_2O) δ 2.30. HRMS: (ESI+) Calculated for $C_{13}H_{19}N_5O_8P$: 404.0977. Found $[M+H]^+$: 404.0971 (-1.41 ppm).

2'-Deoxy-3'-(2''-methoxy-2''-oxoethoxy)-adenosine-5'-benzylphosphate (6)

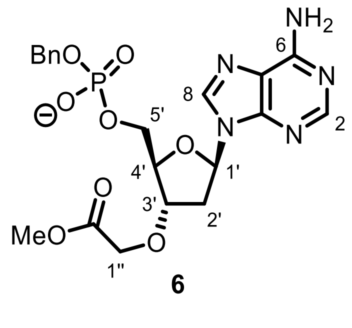

6

20 (400 mg, 0.47 mmol) was subjected to General Procedure D. The residue was triturated with acetone and the product was collected by filtration. This was then subjected to General Procedure C. The residue was triturated with Et_2O then dissolved in 6 M NH_3 in MeOH (10 mL). The solvent was removed *in vacuo* and the residue was dissolved in water (10 mL). Na$^+$ Dowex® (1.0 g) was added and the mixture was stirred for 6 hours. The resin was removed by filtration and the filtrate was lyophilised to give the product as a white solid (142 mg, 91%). ^{1}H (500 MHz, CD_3OD) δ 8.44 (1H, s, C(2)<u>H</u> or C(8)<u>H</u>), 8.19

(1H, s, C(2)H or C(8)H), 7.31 – 7.19 (5H, m, 5 x ArH), 6.43 (1H, ~t, *J* 6.5, C(1')H), 4.39 – 4.35 (1H, m, C(3')H), 4.31 – 4.28 (1H, m, C(4')H), 4.19 (2H, ABq, J_{AB} 16.5, C(1'')H_2), 4.13 – 4.05 (2H, s, C(5')H_2), 3.74 (3H, s, CO_2CH_3), 2.75 (1H, dt, *J* 13.1, 6.5, one of C(2')H_2), 2.60 (1H, dd, *J* 13.2, 4.8, one of C(2')H_2) (benzylic CH_2 obscured by water peak (confirmed by HSQC)). ^{13}C NMR (126 MHz, MeOD) δ 171.1, 155.8, 152.4, 139.8, 138.01, 137.95, 127.9, 127.3, 127.0, 84.3, 84.1 (d, *J* 8.0), 80.8, 67.2, 66.0, 65.4, 51.0, 36.9 (2 x quaternaries absent). ^{31}P NMR (202 MHz, MeOD) δ -3.24. HRMS: (ESI$^+$) Calculated for $C_{20}H_{25}N_5O_8P$: 494.1435. Found $[M+H]^+$: 494.141 (-1.07 ppm).

2'-Deoxy-5'-azidoadenosine (21)

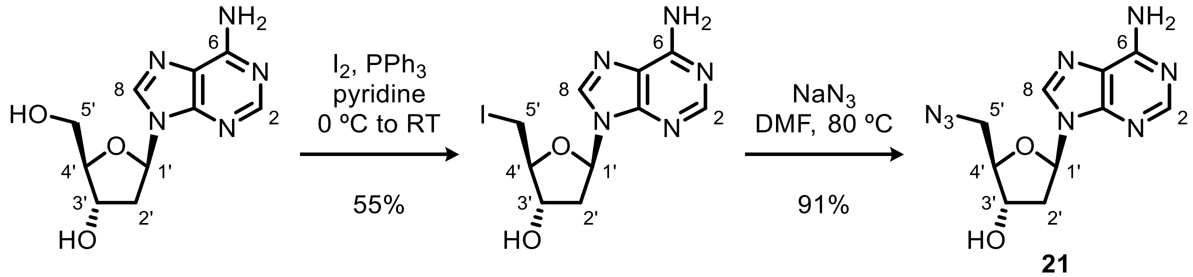

21

2'-Deoxyadenosine monohydrate (5.40 g, 20.0 mmol) was dried by evaporation from anhydrous pyridine three times. The dried nucleoside was dissolved in anhydrous pyridine (63 mL) and cooled to 0 °C. A pre-cooled (0 °C) solution of I_2 (7.60 g, 30.0 mmol) and PPh_3 (7.90 g, 30.0 mmol) in pyridine (30 mL) was added dropwise over 30 minutes. The reaction mixture was allowed to warm to room temperature and stirred for 2 hours. The reaction mixture was concentrated *in vacuo* then partition between 1 M aq. $Na_2S_2O_3$ (50 mL) and a 7:3 mixture of $CHCl_3$ and 2-propanol (100 mL). The product was extracted with a 7:3 mixture of $CHCl_3$ and 2-propanol (5 x 100 mL). The combined organics were dried over $MgSO_4$ and concentrated *in vacuo*. Purification by flash column chromatography (SiO_2; gradient elution, CH_2Cl_2:MeOH; 98:2 to 9:1) gave 2'-deoxy-5'-iodoadenosine as a pale yellow foam (3.90 g, 55%). R_f = 0.25 (CH_2Cl_2:MeOH; 9:1). ^{1}H (500 MHz, MeOD) δ 8.29 (1H, s, C(2)H or C(8)H), 8.20 (1H, s, C(2)H or C(8)H), 6.43 (1H, ~t, *J* 7.0, C(1')H), 4.59 – 4.47 (1H, m, C(3')H), 4.08 – 3.96 (1H, m, C(4')H), 3.53 (1H, dd, *J* 10.5, 6.5, one of C(5')H_2), 3.44 (1H, dd, *J* 10.5, 5.5, one of C(5')H_2), 2.97 (1H, dt, *J* 13.5, 6.5, one of C(2')H_2), 2.47 (1H, ddd, *J* 13.5, 6.5, 3.5, one of C(2')H_2). HRMS: (ESI$^+$) Calculated for $C_{10}H_{12}IN_5NaO_2$: 383.9938. Found $[M+Na]^+$: 383.9936 (-0.74ppm).

2'-Deoxy-5'-iodoadenosine (3.90 g, 10.8 mmol) was dissolved in DMF (36 mL). NaN_3 (1.40 g, 21.6 mmol) was added and the reaction mixture was heated to 80 °C and stirred for 6 hours. The reaction mixture was concentrated in vacuo to *ca*. 10 mL (CAUTION: DO NOT ALLOW TO CONCENTRATE TO DRYNESS). The reaction mixture was concentrated *in vacuo* then partition between water (50 mL) and a 7:3 mixture of $CHCl_3$ and 2-propanol (100 mL). The product was extracted with a 7:3 mixture of $CHCl_3$ and 2-propanol (5 x 100 mL). The combined organics were dried over $MgSO_4$ and concentrated in vacuo. Purification by flash column chromatography (SiO_2; gradient elution, CH_2Cl_2:MeOH; 95:5 to 9:1) gave gave the title compound as a pale yellow foam (2.70 g, 91%). R_f = 0.25 (CH_2Cl_2:MeOH; 9:1). ^{1}H (500 MHz, CD_3OD) δ 8.30 (1H, s, C(2)H or C(8)H), 8.21 (1H, s, C(2)H or C(8)H), 6.44 (1H, ~t, *J* 6.5, C(1')H), 4.58 (1H, dt, *J* 6.5, 4.0, C(3')H), 4.10 (1H, dt, *J* 6.0, 4.0, C(4')H), 3.63 (1H, dd, *J* 13.0, 6.0, one of C(5')H_2), 3.56 (1H, dd, *J* 13.0, 4.0, one of C(5')H_2), 2.95 (1H, dt, *J* 13.5, 6.5, one of C(2')H_2), 2.48 (1H, ddd, *J* 13.5, 6.5, 4.0, one of C(2')H_2). ^{13}C NMR (126 MHz, CD_3OD) δ 155.9, 152.4, 149.0, 139.8, 119.2, 85.8, 84.3, 71.5, 51.9, 38.8. HRMS: (ESI$^+$) Calculated for $C_{10}H_{12}N_8NaO_2$: 299.0975. Found $[M+Na]^+$: 299.0983 (-2.58 ppm).

The spectroscopic properties were consistent with the data available in the literature.[10]

2'-Deoxy-5'-(4''-((benzyloxy)carbonyl)-1*H*-1,2,3-triazol-1-yl)-adenosine (9)

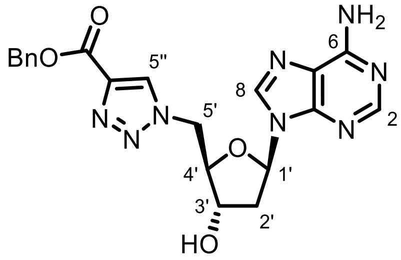

9

21 (750 mg, 2.72 mmol) was subjected to General Procedure E using benzyl propiolate. Purification by flash column chromatography (SiO_2; gradient elution, CH_2Cl_2:MeOH; 98:2 to 94:6) gave the title compound as a pale yellow foam (1.14 g, 96%). R_f = 0.2 (CH_2Cl_2:MeOH; 95:5). ^{1}H NMR (500 MHz, MeOD) δ 8.21 (1H, s, C(5'')H), 8.14 (1H, s, C(2)H or C(8)H), 8.13 (1H, s, C(2)H or C(8)H), 7.34 – 7.24 (5H, m, 5 x ArH), 6.36 (1H, dd, *J* 7.0, 6.0, C(1')H), 5.26 (2H, ABq, J_{AB} 12.5, benzylic CH_2), 4.90 (1H, dd, *J* 14.5, 6.0, one of C(5')H_2), 4.78 (1H, dd, *J* 14.5, 4.0, one of C(5')H_2), 4.74 – 4.69 (1H, m, C(3')H), 4.32 (1H, dt, *J* 6.0, 4.5, C(4')H), 2.96 (1H, ~dt, *J* 13.5, 6.0, one of C(2')H_2), 2.51 (1 H, ddd, *J* 13.5, 7.0, 5.5, one of C(2')H_2). ^{13}C NMR (126 MHz, MeOD) δ 161.6, 157.1, 153.8, 150.1, 141.6, 140.0, 136.8, 130.8, 129.5, 129.3, 129.2, 120.6, 85.8, 85.6, 72.1, 67.8, 52.5, 39.3. HRMS: (ESI$^+$) Calculated for $C_{20}H_{20}N_8NaO_2$: 459.1500. Found [M+Na]$^+$: 459.1509 (-1.96 ppm).

2'-Deoxy-5'-(4''-(bis(benzyloxy)phosphoryl)-1*H*-1,2,3-triazol-1-yl)-adenosine (22)

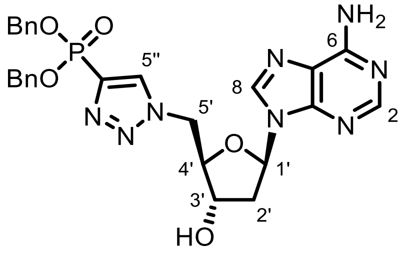

22

21 (1.00 g, 3.62 mmol) was subjected to General Procedure E using dibenzyl ethynylphosphonate. Purification by flash column chromatography (SiO_2; gradient elution, CH_2Cl_2:MeOH; 95:5 to 9:1) gave the title compound as a pale yellow foam (1.22 g, 60%). R_f = 0.25 (CH_2Cl_2:MeOH; 9:1). ^{1}H NMR (500 MHz, MeOD) δ 8.20 (2H, s, C(5'')H and C(2)H or C(8)H), 8.12 (1H, s, C(2)H or C(8)H), 7.22 (10H, s, 10 x ArH), 6.38 (1H, ~t, *J* 6.5, C(1')H), 5.08 – 4.97 (4H, m, 2 x benzylic CH_2), 4.93 – 4.88 (2H, m, C(5')H_2), 4.69 – 4.64 (1H, m, C(3')H), 4.34 – 4.28 (1H, m, C(4')H), 2.88 (1H, dt, *J* 13.5, 6.5, one of C(2')H_2), 2.44 (1H, ddd, *J* 13.5, 6.5, 4.0, one of C(2')H_2). ^{13}C NMR (126 MHz, MeOD) δ 157.3, 153.9, 150.2, 141.4, 137.2 (d, *J* 244.5), 136.9, 136.8, 133.5 (d, *J* 34.0), 129.6, 129.5, 129.1, 120.8, 86.11, 86.05, 72.8, 69.8 – 69.7 (m, 2 x benzylic CH_2), 53.0, 39.6. ^{31}P NMR (202 MHz, MeOD) δ 8.23. HRMS: (ESI$^+$) Calculated for $C_{26}H_{27}N_8NaO_5P$: 585.1734. Found [M+Na]$^+$: 585.1736 (-0.32 ppm).

2'-Deoxy-5'-(4''-carboxy-1*H*-1,2,3-triazol-1-yl)-adenosine (7)

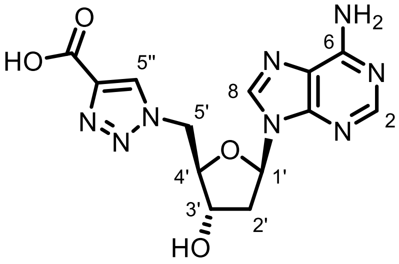

7

9 (100 mg, 0.23 mmol) was dissolved in MeOH (5 mL). 10% Pd/C (49 mg, 0.046 mmol) was added and the reaction mixture was evacuated and back-filled with H_2 three times. The reaction mixture was stirred under and atmosphere of H_2 for 48 hours. The catalyst was removed by filtration through a pad of Celite® and 6 M NH_3 in MeOH (5 mL) was added. The filtrate was concentrated *in vacuo*. Trituration with Et_2O gave the title compound as a white solid (81 mg, 96%). ^{1}H (500 MHz, D_2O) δ 8.05 (1H, s, C(2)H, C(8)H or C(5'')H), 7.76 (1H, s, C(2)H, C(8)H or C(5'')H), 7.66 (1H, s, C(2)H, C(8)H or C(5'')H), 6.21 (1H, br. s, C(1')H), 4.64 (1H, d, *J* 15.0, one of C(5')H_2), 4.62 – 4.54 (1H, m, C(3')H), 4.35 – 4.29 (1H, m, C(4')H), 2.73 – 2.67 (1H, m, one of C(2')H_2), 2.55 (1 H, ~dt, *J* 14.0, 7.0, one of C(2')H_2). ^{13}C NMR (126 MHz, D_2O) δ 155.5, 152.7, 148.4, 144.5, 139.2, 128.1, 118.7, 83.5, 83.2, 70.0, 50.2, 37.1 (1 x quaternary absent). HRMS: (ESI$^+$) Calculated for $C_{13}H_{15}N_8O_4$: 347.1221. Found $[M+H]^+$: 347.1227 (1.51 ppm).

2'-Deoxy-3'-*O*-phosphoryl-5'-(4''-carboxy-1*H*-1,2,3-triazol-1-yl)-adenosine (8)

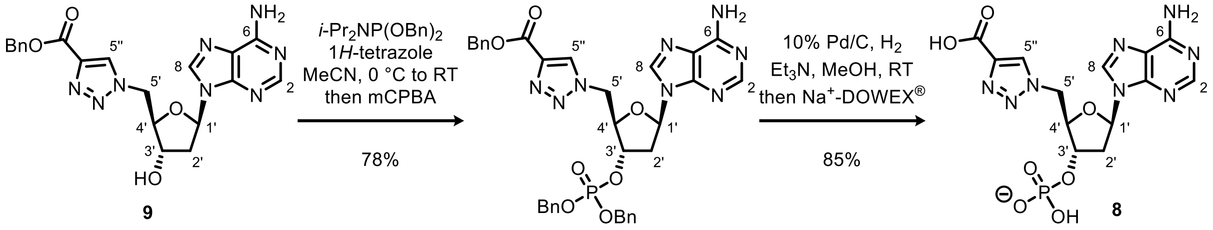

9 (162 mg, 0.37 mmol) was subjected to General Procedure A using dibenzyl *N*,*N*-diisopropyl phosphoramidite. Purification by flash column (SiO_2; gradient elution, CH_2Cl_2:MeOH; 98:2 to 9:1) gave the product as a colourless oil (201 mg, 78%). R_f = 0.25 (CH_2Cl_2:MeOH; 9:1). ^{1}H (500 MHz, CDCl3) δ 8.27 (1H, s, C(2)H, C(8)H or C(5'')H), 7.92 (1H, s, C(2)H, C(8)H or C(5'')H), 7.71 (1H, s, C(2)H, C(8)H or C(5'')H), 7.43 – 7.27 (15H, m, 15 x ArH), 6.17 (2H, s, NH_2), 6.13 (1H, ~t, *J* 6.5, C(1')H), 5.34 (2H, s, $COCH_2Ph$), 5.28 – 5.22 (1H, m, C(3')H), 5.17 – 5.04 (4H, m, $PO(CH_2Ph)_2$), 4.86 (1H, dd, *J* 14.0, 7.5, one of C(5')H_2), 4.71 (1H, dd, *J* 14.0, 3.0, one of C(5')H_2), 4.45 – 4.40 (1H, m, C(4')H), 3.08 – 3.00 (1H, m, one of C(2')H_2), 2.48 (1H, ddd, *J* 14.0, 6.5, 3.5, one of C(2')H_2). ^{13}C NMR (126 MHz, CDCl3) δ 160.4, 155.9, 153.1, 149.2, 139.9, 139.8, 135.5, 135.4 – 135.3 (m, 2 x q), 129.1, 129.0, (d, *J* 5.5), 128.8 (d, *J* 3.2), 128.6, 128.5, 128.4, 128.3 (d, *J* 3.4), 120.7, 85.0, 83.3 (d, *J* 6.1), 70.1 – 70.0 (m, 2 x benzylic CH_2), 66.8, 51.3, 36.6 (d, *J* 4.5). ^{31}P NMR (202 MHz, CDCl3) δ -1.75. HRMS: (ESI$^+$) Calculated for $C_{34}H_{34}N_8O_7P$: 697.2294. Found $[M+H]^+$: 697.2299 (0.78 ppm).

The benzyl-protected nucleoside (201 mg, 0.29 mmol) subjected to General Procedure B. The title compound was obtained as a white solid (109 mg, 85%). ^{1}H (500 MHz, D_2O) δ 8.06 (1H, s, C(2)H, C(8)H or C(5'')H), 7.88 (1H, s, C(2)H, C(8)H or C(5'')H), 7.78 (1H, s, C(2)H, C(8)H or C(5'')H), 6.26 (1H, s, C(1')H), 4.91 (1H, s, C(3')H), 4.49 (1H, s, C(4')H), 2.76 – 2.57 (2H, m, C(2')H_2) (C(5')H_2 obscured by residual solvent peak (confirmed by COSY and HSQC). ^{13}C NMR (126 MHz, D_2O) δ

155.5, 152.6, 148.7, 139.5, 128.2, 118.8, 83.7, 83.3 (d, *J* 7.0), 73.6 (d, *J* 5.0), 50.7, 36.7 (triazole quaternary absent). ^{31}P NMR (202 MHz, D_2O) δ 0.88. HRMS: (ESI^+) Calculated for $C_{13}H_{16}N_8O_7P$: 427.0885. Found $[M+H]^+$: 427.0880 (-1.18 ppm).

2'-Deoxy-3'-O-(bis-2-cyanoethyl)-phosphoryl-5'-(4-((benzyloxy)carbonyl)-1*H*-1,2,3-triazol-1-yl)-adenosine (23)

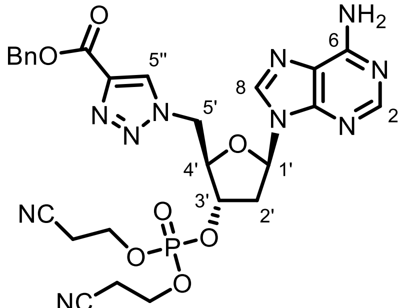

23

9 (300 mg, 0.69 mmol) was subjected to General Procedure A using bis(2-cyanoethyl)-*N*,*N*-diisopropylphosphoramidite. Purification by flash column chromatography (SiO_2; gradient elution, CH_2Cl_2:MeOH; 98:2 to 9:1) gave the title compound as a colourless oil (320 mg, 75%). R_f = 0.25 (CH_2Cl_2:MeOH; 95:5). ^{1}H (500 MHz, $CDCl_3$) δ 8.26 (1H, s, C(2)H, C(8)H or C(5'')H), 7.99 (1H, s, C(2)H, C(8)H or C(5'')H), 7.85 (1H, s, C(2)H, C(8)H or C(5'')H), 7.41 – 7.23 (5H, m, 5 x ArH), 6.34 (1H, ~t, *J* 6.5, C(1')H), 6.30 (2H, br. s, NH_2), 5.54 – 5.46 (1H, m, C(3')H), 5.35 – 5.27 (2H, m, 2 x benzylic CH_2), 5.02 (1H, dd, *J* 14.5, 6.5, one of C(5')H_2), 4.89 (1H, dd, *J* 14.5, 4.0, one of C(5')H_2), 4.66 – 4.58 (1H, m, C(4')H), 4.43 – 4.33 (4H, m, 2 x OCH_2CH_2CN), 3.36 – 3.28 (1H, m, one of C(2')H_2), 2.89 – 2.75 (5H, m, one of C(2')H_2 and 2 x OCH_2CH_2CN). ^{13}C NMR (126 MHz, $CDCl_3$) δ 160.2, 155.9, 153.0, 149.0, 140.1, 139.7, 135.4, 129.4, 128.6, 128.4, 120.5, 116.8, 84.7, 82.9 (d, *J* 6.7), 77.9 (d, *J* 5.3), 66.9 – 63.0 (m, 2 x CH_2), 51.0, 36.6, 19.8 (d, *J* 7.3). ^{31}P NMR (202 MHz, $CDCl_3$) δ -3.34. HRMS: (ESI^+) Calculated for $C_{26}H_{28}N_{10}O_7P$: 623.1886. Found $[M+H]^+$: 623.1884 (-0.25 ppm).

2'-Deoxy-3'-*O*-phosphoryl-5'-(4-((benzyloxy)carbonyl)-1*H*-1,2,3-triazol-1-yl)-adenosine (10)

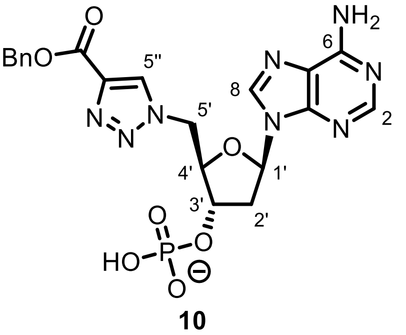

10

23 (255 mg, 0.41 mmol) was subjected to General Procedure F. The title compound was obtained as a white solid (163 mg, 74%). ^{1}H (500 MHz, D_2O) δ 7.96 (1H, s, C(2)H, C(8)H or C(5'')H), 7.83 (1H, s, C(2)H, C(8)H or C(5'')H), 7.81 (1H, s, C(2)H, C(8)H or C(5'')H), 7.30 – 7.14 (5H, m, 5 x ArH), 6.16 (1H, dd, *J* 7.0, 3.5, C(1')H), 5.10 (2H, ABq, J_{AB} 12.5, benzylic CH_2), 4.98 – 4.89 (1H, m, C(3')H), 4.82 (1H, dd, *J* 15.0, 4.0, one of C(5')H_2), 4.48 – 4.37 (1H, m, C(4')H), 2.84 – 2.77 (1H, m, one of C(2')H_2), 2.68 (1H, ~dt, *J* 14.5, 7.0, one of C(2')H_2) (one of C(5')H_2 obscured by water peak (confirmed by COSY and HSQC)). ^{13}C NMR (126 MHz, D_2O) δ 160.6, 155.0, 152.4, 148.4, 139.3, 138.0, 134.7, 130.1, 128.6,

128.5, 127.9, 118.4, 83.5, 82.8 (d, *J* 7.4), 73.2, 67.2, 50.6, 36.6. ^{31}P NMR (202 MHz, D_2O) δ 0.84. HRMS: (ESI$^+$) Calculated for $C_{20}H_{22}N_8O_7P$: 517.1355. Found $[M+H]^+$: 517.1357 (0.47 ppm).

2'-Deoxy-5'-(4''-phosphoryl)-1*H*-1,2,3-triazol-1-yl)-adenosine (11)

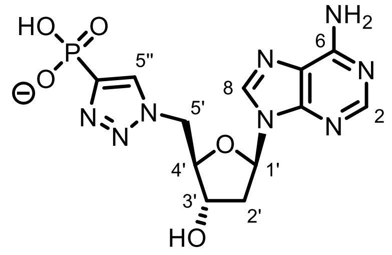

11

22 (113 mg, 0.20 mmol) was subjected to General Procedure B. The title compound was obtained as a white solid (72 mg, 86%). ^{1}H (500 MHz, D_2O) δ 8.11 (1H, s, C(2)H, C(8)H or C(5'')H), 8.07 (1H, s, C(2)H, C(8)H or C(5'')H), 8.03 (1H, br. s, C(2)H, C(8)H or C(5'')H), 6.34 (1H, t, *J* 6.5, C(1')H), 4.64 (1H, s, C(3')H), 4.49 (1H, s, C(4')H), 2.69 – 2.62 (1H, m, one of C(2')H_2), 2.62 – 2.54 (1H, m, one of C(2')H_2) (C(5')H_2 obscured by residual solvent peak (confirmed by COSY and HSQC)). ^{13}C NMR (126 MHz, D_2O) δ 155.2, 152.5, 139.7, 84.1, 83.7, 71.3, 51.4 (br.), 38.1 (4 x quaternaries absent). ^{31}P NMR (202 MHz, D_2O) δ 0.30. HRMS: (ESI$^+$) Calculated for $C_{12}H_{16}N_8O_5P$: 383.0987. Found $[M+H]^+$: 383.0991 (1.11 ppm).

2'-Deoxy-3'-*O*-phosphoryl-5'-(4''-phosphoryl)-1*H*-1,2,3-triazol-1-yl)-adenosine (12)

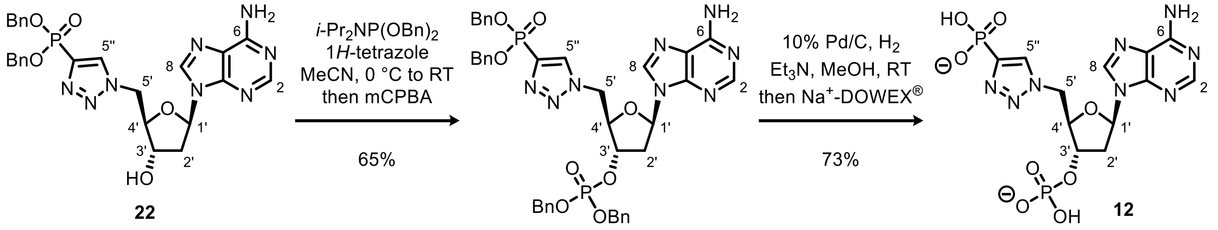

22 (538 mg, 2.00 mmol) was subjected to General Procedure A using dibenzyl *N*,*N*-diisopropyl phosphoramidite. Purification by flash column (SiO_2; gradient elution, CH_2Cl_2:MeOH; 98:2 to 95:5) gave the product as a colourless oil (1.33 g, 86%). R_f = 0.3 (CH_2Cl_2:MeOH; 9:1). ^{1}H (500 MHz, $CDCl_3$) δ 8.31 (1H, s, C(2)H or C(8)H), 7.83 (1H, s, C(5'')H), 7.69 (1H, s, C(2)H or C(8)H), 7.48 – 7.24 (20H, m, 20 x ArH), 6.13 (1H, ~t, *J* 7.0, C(1')H), 5.97 (2H, br. s, NH_2), 5.27 – 5.20 (1H, m, C(3')H), 5.19 – 5.03 (8H, m, 4 x benzylic CH_2), 4.84 (1H, dd, *J* 14.0, 8.0, one of C(5')H_2), 4.74 (1H, dd, *J* 14.0, 3.0, one of C(5')H_2), 4.44 – 4.38 (1H, m, C(4')H), 3.02 – 2.94 (1H, m, one of C(2')H_2), 2.47 (1H, ddd, *J* 14.0, 7.0, 3.0, one of C(2')H_2). ^{13}C NMR (126 MHz, $CDCl_3$) δ 156.0, 153.1, 149.2, 139.7, 137.2 (d, *J* 242.0), 135.81, 135.76, 135.44 – 135.35 (m, 2 x q), 131.9 (d, *J* 34.0), 129.0 (d, *J* 5.0), 128.9 (d, *J* 3.0), 128.6, 128.5, 128.4 (d, *J* 4.0), 128.1, 120.7, 84.9, 83.5 (d, *J* 6.0), 77.7 (d, *J* 5.5), 70.1 (d, *J* 6.0), 68.5 – 68.4 (m), 51.4, 36.8 (d, *J* 4.5), 36.8. ^{31}P NMR (202 MHz, $CDCl_3$) δ 7.24, -1.69. HRMS: (ESI$^+$) Calculated for $C_{40}H_{40}N_8NaO_8P_2$: 845.2348. Found $[M+Na]^+$: 845.2355 (0.88 ppm).

The benzyl-protected nucleoside (1.20 g, 1.46 mmol) was subjected to General Procedure B. The title compound was obtained as a white solid (539 mg, 73%). ^{1}H (500 MHz, D_2O) δ 8.00 – 7.95 (2H, m, 2 of C(2)H, C(8)H and C(5'')H), 7.89 (1H, s, C(2)H, C(8)H or C(5'')H), 6.25 (1H, t, *J* 6.5, C(1')H), 4.83 – 4.76 (1H, m, C(3')H), 4.55 – 4.48 (1H, m, C(4')H), 2.67 – 2.59 (1H, m, one of C(2')H_2), 2.57 – 2.48

(1H, m, one of C(2')H$_2$) (C(5')H$_2$ obscured by residual solvent peak (confirmed by COSY and HSQC)). ^{13}C NMR (126 MHz, D_2O) δ 155.2, 152.5, 146.2 (d, *J* 206.0), 139.8, 128.8 (d, *J* 27.5), 118.5, 84.0 (d, *J* 6.0), 83.8, 74.1 (d, *J* 4.5), 51.5, 37.4. ^{31}P NMR (202 MHz, D_2O) δ 2.04, 0.42. HRMS: (ESI$^+$) Calculated for $C_{12}H_{17}N_8O_8P_2$: 463.0650. Found [M+H]$^+$: 463.0652 (0.42 ppm).

2'-Deoxy-5'-(4''-benzyloxyphosphoryl)-1*H*-1,2,3-triazol-1-yl)-adenosine (13)

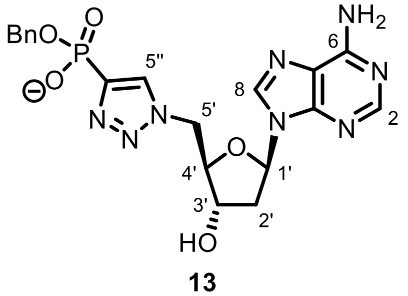

13

22 (250 mg, 0.44 mmol) was subjected to General Procedure D. The residue was purified by flash column (SiO_2; gradient elution, CH_2Cl_2:MeOH; 95:5 to 70:30) to give the title compound colourless glass (134 mg, 61%). R_f = 0.15 (CH_2Cl_2:MeOH; 70:30). ^{1}H (500 MHz, D_2O) δ 7.91 (1H, s, C(2)H, C(8)H or C(5'')H), 7.89 (1H, s, C(2)H, C(8)H or C(5'')H), 7.81 (1H, s, C(2)H, C(8)H or C(5'')H), 7.03 – 6.97 (3H, m, 3 x ArH), 6.88 (2H, br. s, 2 x ArH), 6.15 (1H, ~t, *J* 6.0, C(1')H), 4.59 – 4.53 (1H, m, , one of benzylic CH$_2$), 4.52 – 4.44 (2H, m, C(3')H and one of benzylic CH$_2$), 4.39 – 4.34 (1H, m, C(4')H), 2.48 – 2.35 (2H, m, C(2')H$_2$) (C(5')H$_2$ obscured by residual solvent peak (confirmed by COSY and HSQC)). ^{13}C NMR (126 MHz, D_2O) δ 155.1, 152.4, 148.1, 141.4 (d, *J* 218.5), 139.1, 136.6 (d, *J* 7.0), 130.8 (d, *J* 29.0), 128.2, 127.8, 127.2, 118.4, 83.7, 83.5, 71.00, 66.8 (d, *J* 5.0), 51.0, 38.1. ^{31}P NMR (202 MHz, D_2O) δ 2.60. HRMS: (ESI$^+$) Calculated for $C_{19}H_{21}N_8NaO_5P$: 495.1276. Found [M+Na]$^+$: 495.1281 (1.07 ppm).

2'-Deoxy-3'-*O*-phosphoryl-5'-(4''-benzyloxyphosphoryl)-1*H*-1,2,3-triazol-1-yl)-adenosine (14)

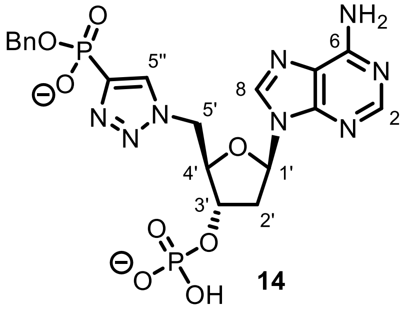

14

13 (300 mg, 0.61 mmol) was subjected to General Procedure A using bis(2-cyanoethyl)-*N*,*N*-diisopropylphosphoramidite. The residue was passed through a pad of SiO_2, eluting with CH_2Cl_2:MeOH; 9:1. Fractions containing product were combined and concentrated *in vacuo*. The residue was subjected to General Procedure F. The title compound was obtained as a white solid (202 mg, 56%). ^{1}H (500 MHz, D_2O) δ 8.22 (1H, s, C(2)H, C(8)H or C(5'')H), 8.20 (1H, s, C(2)H, C(8)H or C(5'')H), 8.03 (1H, s, C(2)H, C(8)H or C(5'')H), 7.19 – 7.11 (3H, m, 3 x ArH), 6.93 (2H, d, *J* 6.5, 2 x ArH), 6.40 (1H, ~t, *J* 6.5, C(1')H), 5.09 – 5.01 (2H, m, C(3')H and one of C(5')H$_2$), 4.98 – 4.93 (1H, m, one of C(5')H$_2$), 4.85 – 4.80 (1H, m, C(4')H), 4.63 (1H, dd, *J* 12.0, 7.5, one of benzylic CH$_2$), 4.52 (1H, dd, *J* 12.0, 8.0, one of benzylic CH$_2$), 2. 86 (1 H, ddd, *J* 13.5, 6.5, 2.5, one of C(2')H$_2$), 2.65 (1H, dt, *J* 13.5, 7.0, one of C(2')H$_2$). ^{13}C NMR (126 MHz, D_2O) δ 155.3, 152.7, 148.5, 139.7, 136.4, 131.3 (d, *J*

29.0), 128.5, 128.2, 127.5, 118.7, 84.1, 83.9 (d, *J* 5.0), 74.2 (d, *J* 4.0), 67.2 (d, *J* 5.0), 52.0, 37.9 (d, *J* 2.5) (triazole quaternary absent). ^{31}P NMR (202 MHz, D_2O) δ 3.84, 3.05. HRMS: (ESI^+) Calculated for $C_{19}H_{22}N_8NaO_8P_2$: 575.0939. Found $[M+Na]^+$: 575.0942 (0.52 ppm).

References

1. G. Jones, P. Willett, R. C. Glen, A. R. Leach, and R. Taylor, *J. Mol. Biol.*, 1997, **267**, 727–748.
2. S. Tanaka, T. Nishiyori, H. Kojo, R. Otsubo, M. Tsuruta, K. Kurogi, M. C. Liu, M. Suiko, Y. Sakakibara and Y. Kakuta, *Sci. Rep.*, 2017, **7**, 8776–8776.
3. C. Liu, J. Sheng, J. M. Krahn, L. Perera, Y. Xu, P. H. Hsieh, W. Dou, J. Liu and L. C. Pedersen, *J. Biol. Chem.*, 2014, **289**, 13407–13418.
4. O. Korb, T. Stützle and T. E. Exner, *J. Chem. Inf. Model.*, 2009, **49**, 84–96.
5. D. P. Byrne, Y. Li, P. Ngamlert, K. Ramakrishnan, C. E. Eyers, C. Wells, D. H. Drewry, W. J. Zuercher, N. G. Berry, D. G. Fernig and P. A. Eyers, *Biochem. J.*, 2018, **475**, 2435–2455.
6. D. P. Byrne, Y. Li, P. Ngamlert, K. Ramakrishnan, C. E. Eyers, C. Wells, D. H. Drewry, W. J. Zuercher, N. G. Berry, D. G. Fernig and P. A. Eyers, *Biochem. J.*, 2018, **475**, 2417–2433.
7. J. W. Kehoe, N. Velappan, M. Walbolt, J. Rasmussen, D. King, J. Lou, K. Knopp, P. Pavlik, J. D. Marks, C. R. Bertozzi and A. R. Bradbury, *Mol. Cell Proteomics*, 2006, **5**, 2350–2363.
8. L. J. Blackwell, S. Birkos, R. Hallam, G. Van De Carr, J. Arroway, C. M. Suto and W. P. Janzen, *Methods Mol. Biol.*, 2009, **565**, 225–237.
9. B. L Hevroni, A. H. Sayer, E. Blum and B. Fischer, *Inorg. Chem.*, 2014, **53**, 1594–1605.
10. M. Mattarella, L. Berstis, K. K. Baldridge, and J. S. Siegel, *Bioconjug. Chem.*, 2014, **25**, 115–128.

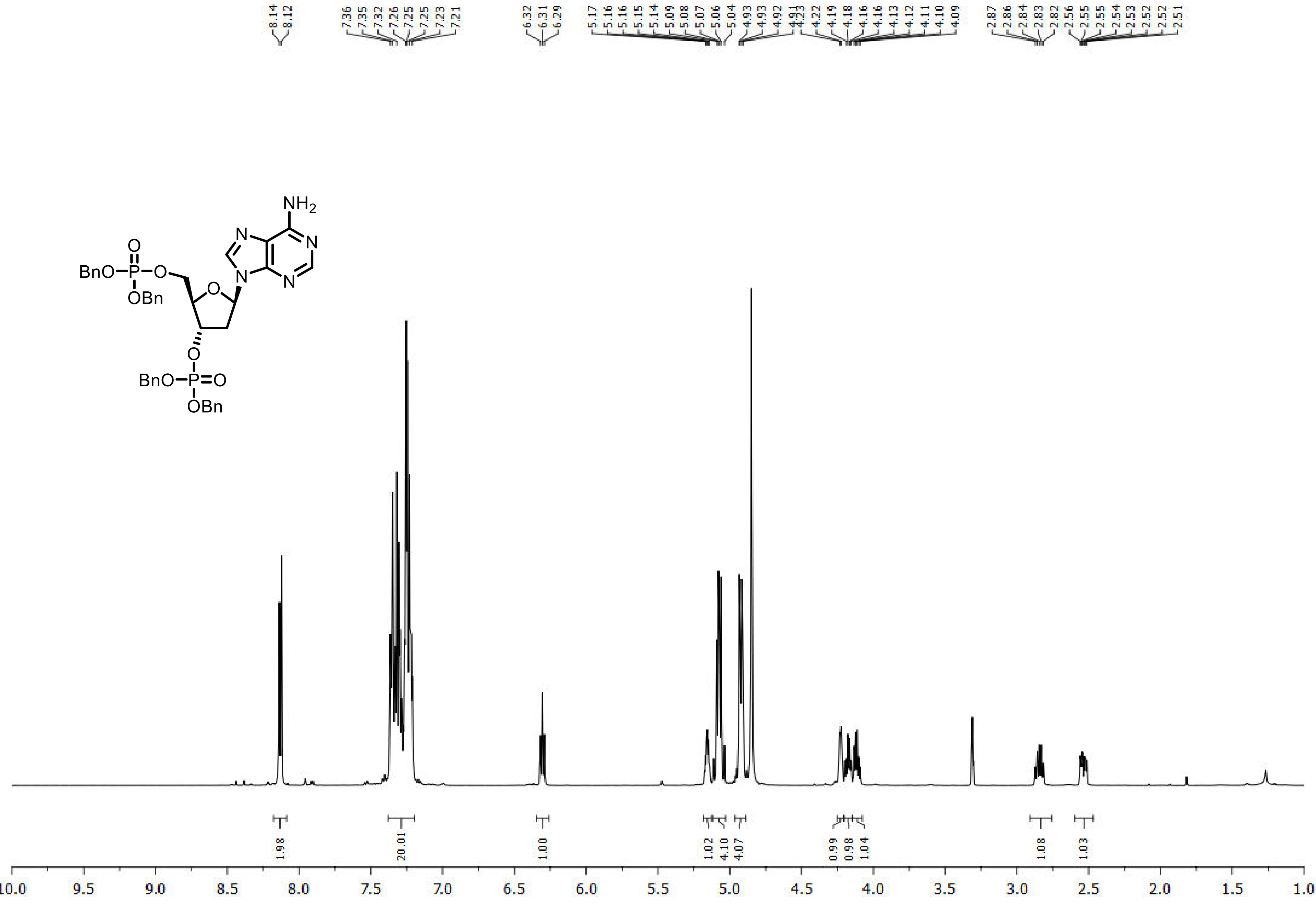
8.14
8.12
7.36
7.35
7.32
7.26
7.25
7.25
7.23
7.21
6.32
6.31
6.29
5.17
5.16
5.16
5.15
5.14
5.09
5.08
5.07
5.06
5.04
4.93
4.93
4.92
4.91
4.23
4.22
4.19
4.18
4.16
4.16
4.13
4.12
4.11
4.10
4.09
2.87
2.86
2.84
2.83
2.82
2.56
2.55
2.55
2.54
2.53
2.52
2.52
2.51
NH2
N
N
N
N
O
BnO
P
O
OBn
O
O
BnO
P
O
OBn
1.98
20.01
1.00
1.02
4.10
4.07
0.99
0.98
1.04
1.08
1.03
10.0
9.5
9.0
8.5
8.0
7.5
7.0
6.5
6.0
5.5
5.0
4.5
4.0
3.5
3.0
2.5
2.0
1.5
1.0

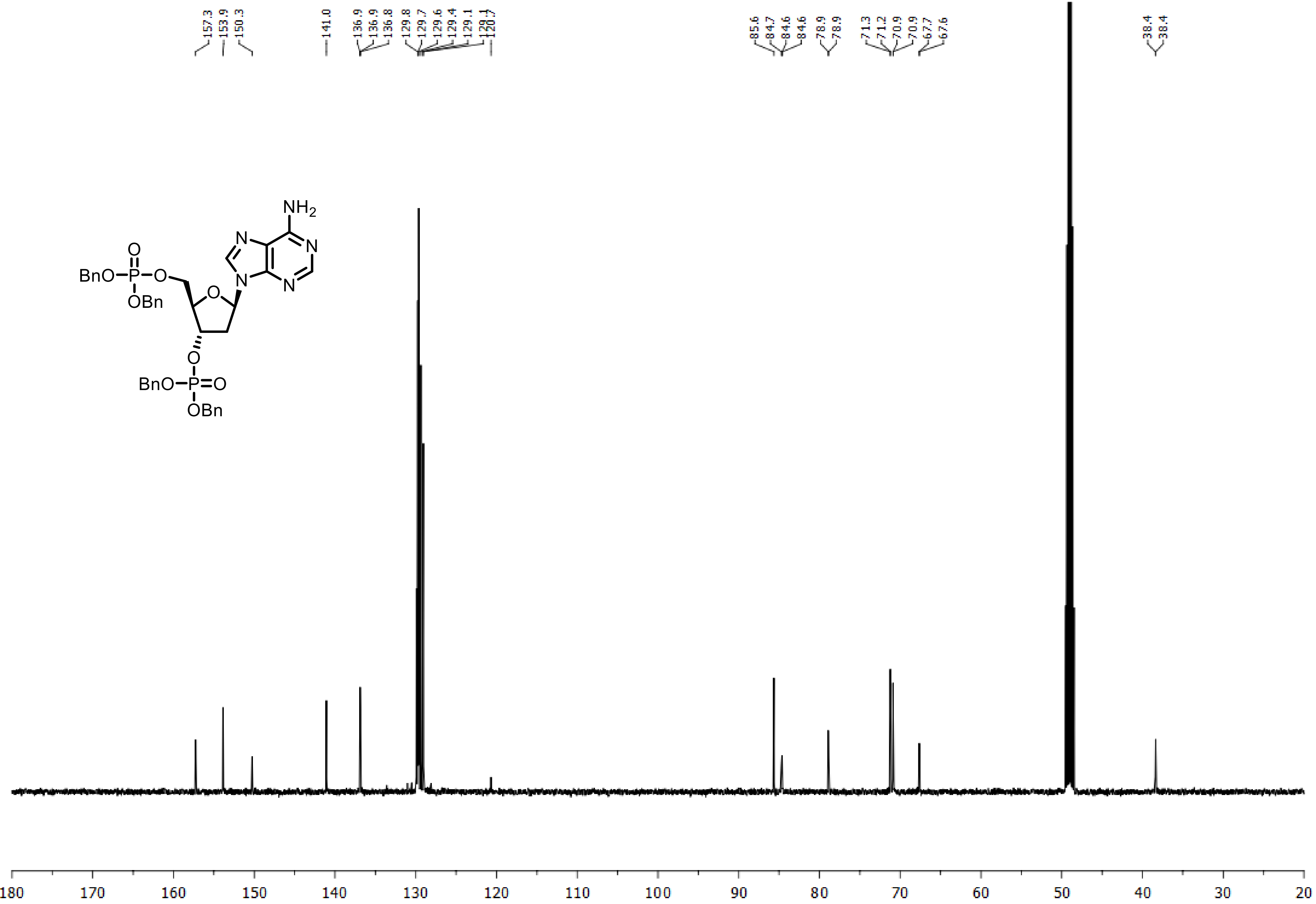
157.3
153.9
150.3
141.0
136.9
136.9
136.8
129.8
129.7
129.6
129.4
129.1
128.1
120.7
85.6
84.7
84.6
84.6
78.9
78.9
71.3
71.2
70.9
70.9
67.7
67.6
38.4
38.4
NH2
N
N
N
N
O
BnO
P
O
OBn
O
O
BnO
P
O
OBn
180
170
160
150
140
130
120
110
100
90
80
70
60
50
40
30
20

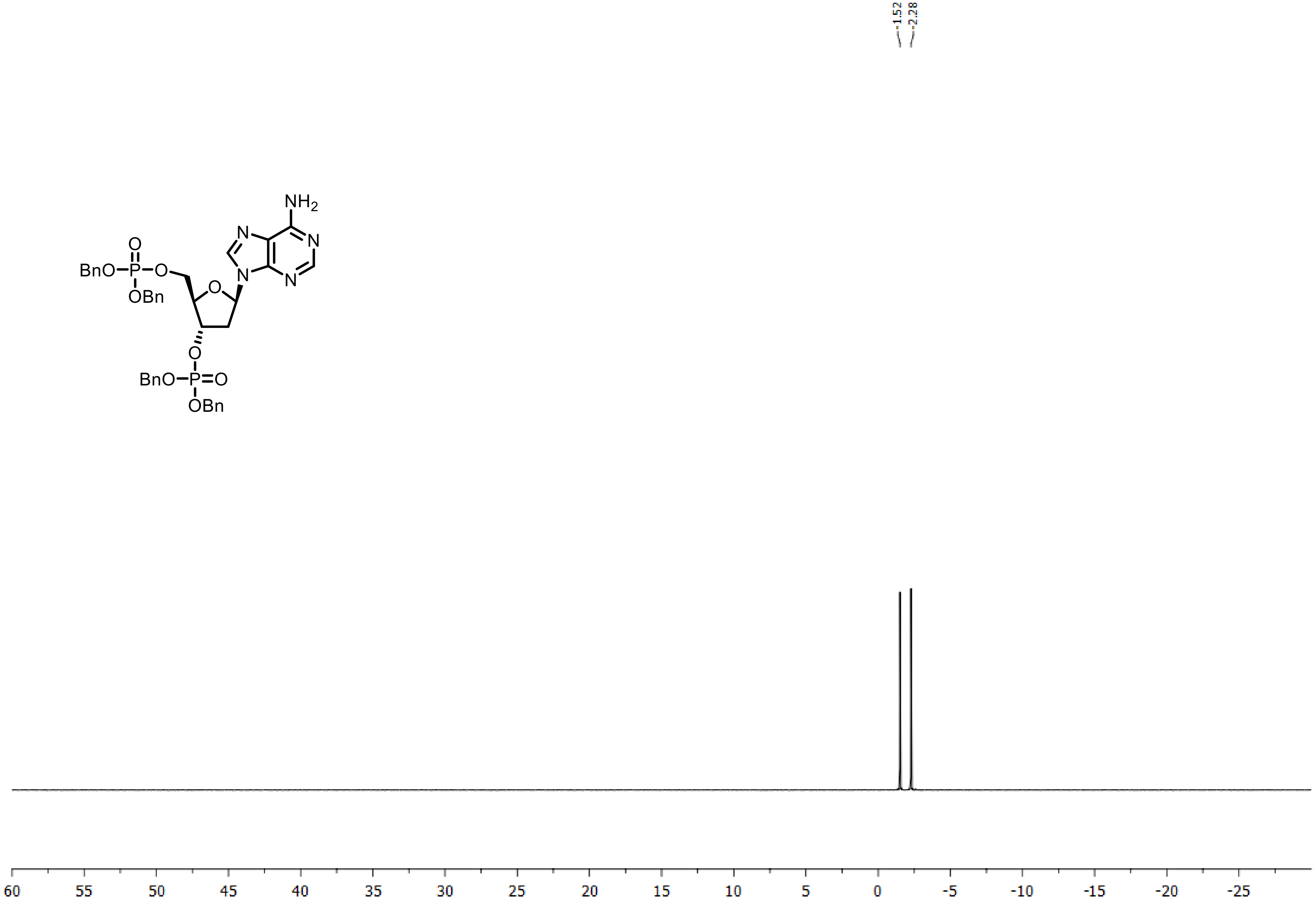

-1.52
-2.28
NH2
N
N
N
N
O
BnO
P
O
OBn
O
O
BnO
P
O
OBn
60 55 50 45 40 35 30 25 20 15 10 5 0 -5 -10 -15 -20 -25

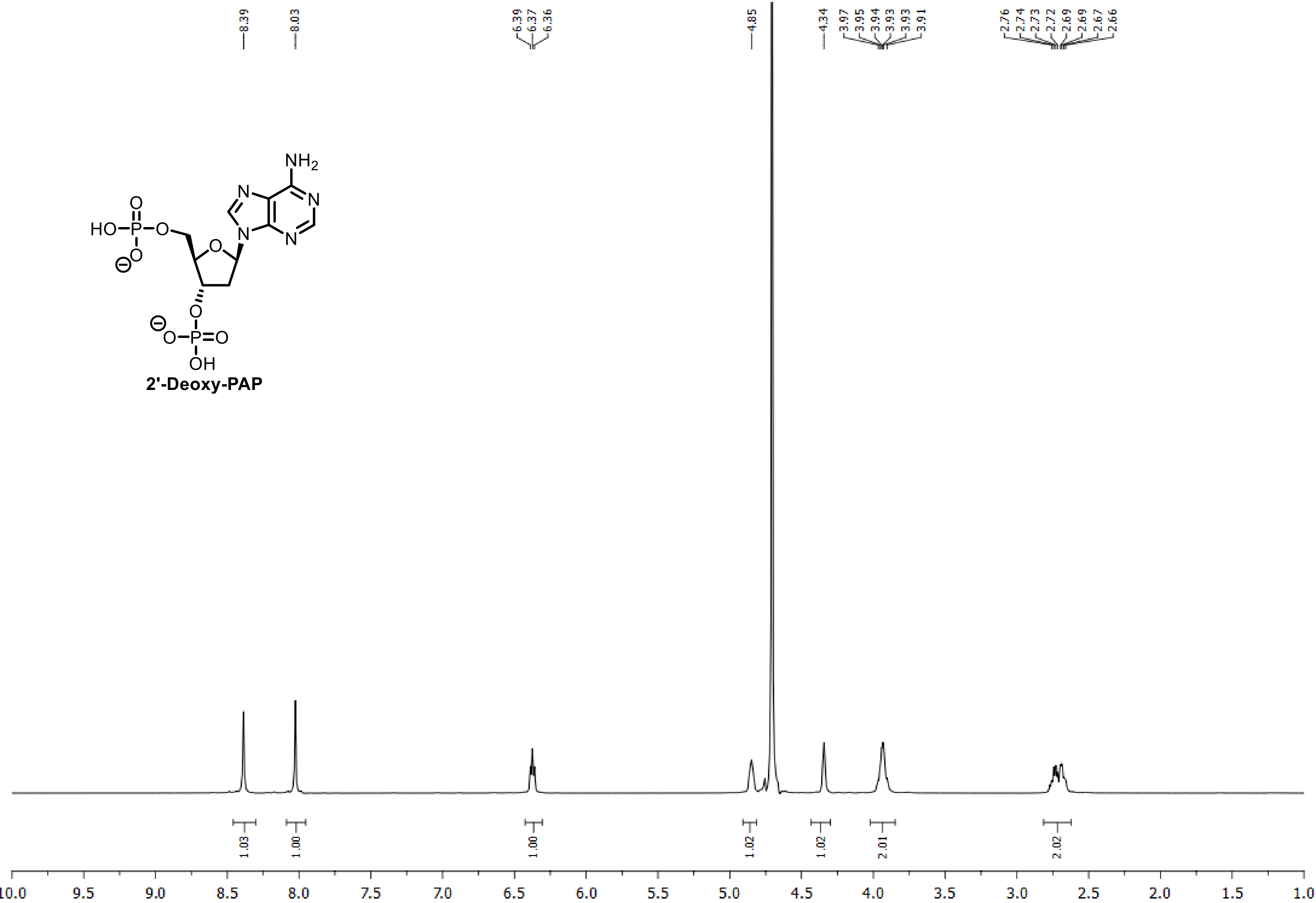
8.39
8.03
6.39
6.37
6.36
4.85
4.34
3.97
3.95
3.94
3.93
3.93
3.91
2.76
2.74
2.73
2.72
2.69
2.69
2.67
2.66
2'-Deoxy-PAP
1.03
1.00
1.00
1.02
1.02
2.01
2.02
10.0 9.5 9.0 8.5 8.0 7.5 7.0 6.5 6.0 5.5 5.0 4.5 4.0 3.5 3.0 2.5 2.0 1.5 1.0

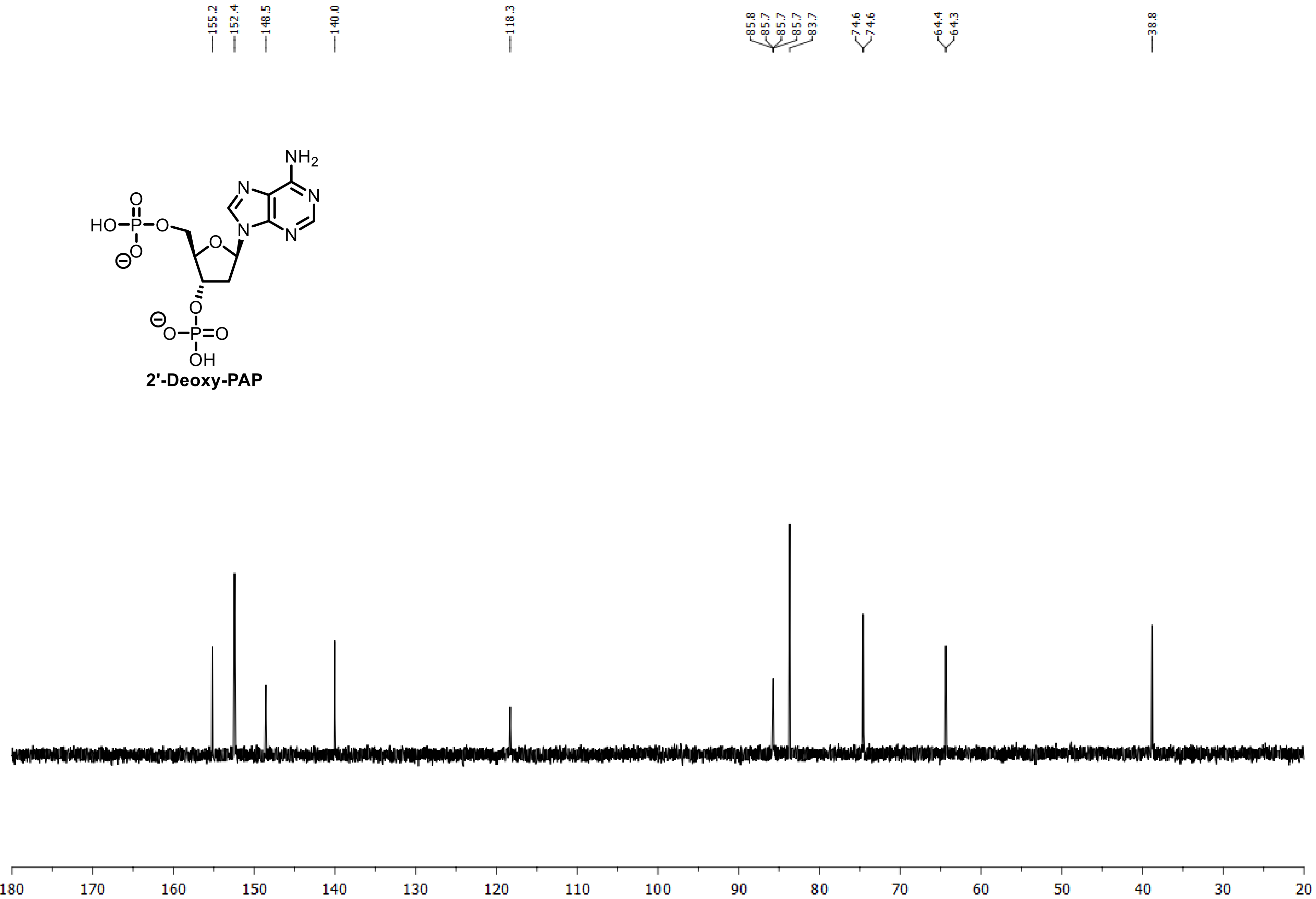
155.2
152.4
148.5
140.0
118.3
85.8
85.7
85.7
85.7
83.7
74.6
74.6
64.4
64.3
38.8
NH2
N
HO
P
O
2'-Deoxy-PAP
180 170 160 150 140 130 120 110 100 90 80 70 60 50 40 30 20

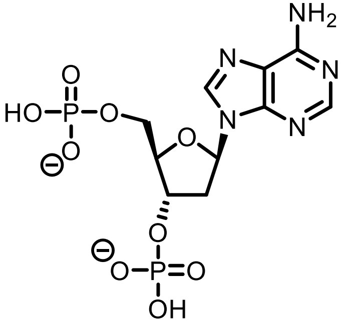
NH2
N
N
N
N
O
HO
P
O
O
O
O
O
P
O
OH

2'-Deoxy-PAP

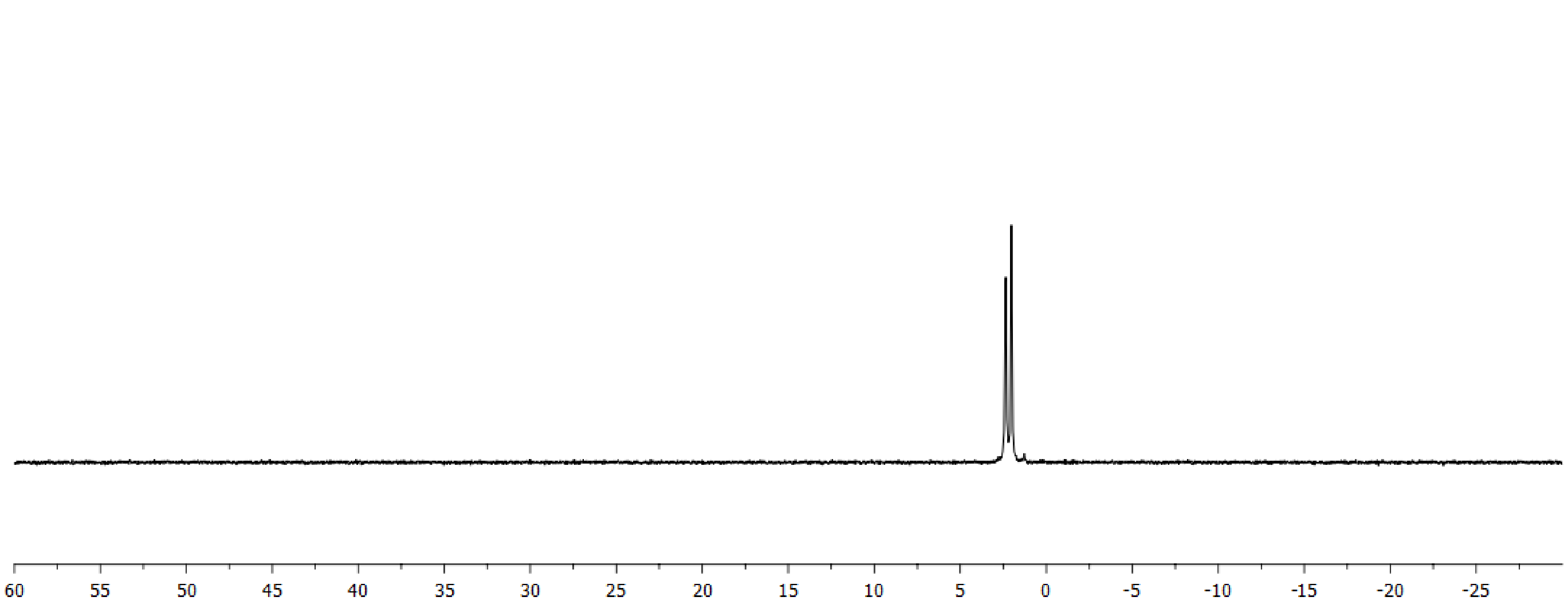
2.36
2.03
60
55
50
45
40
35
30
25
20
15
10
5
0
-5
-10
-15
-20
-25

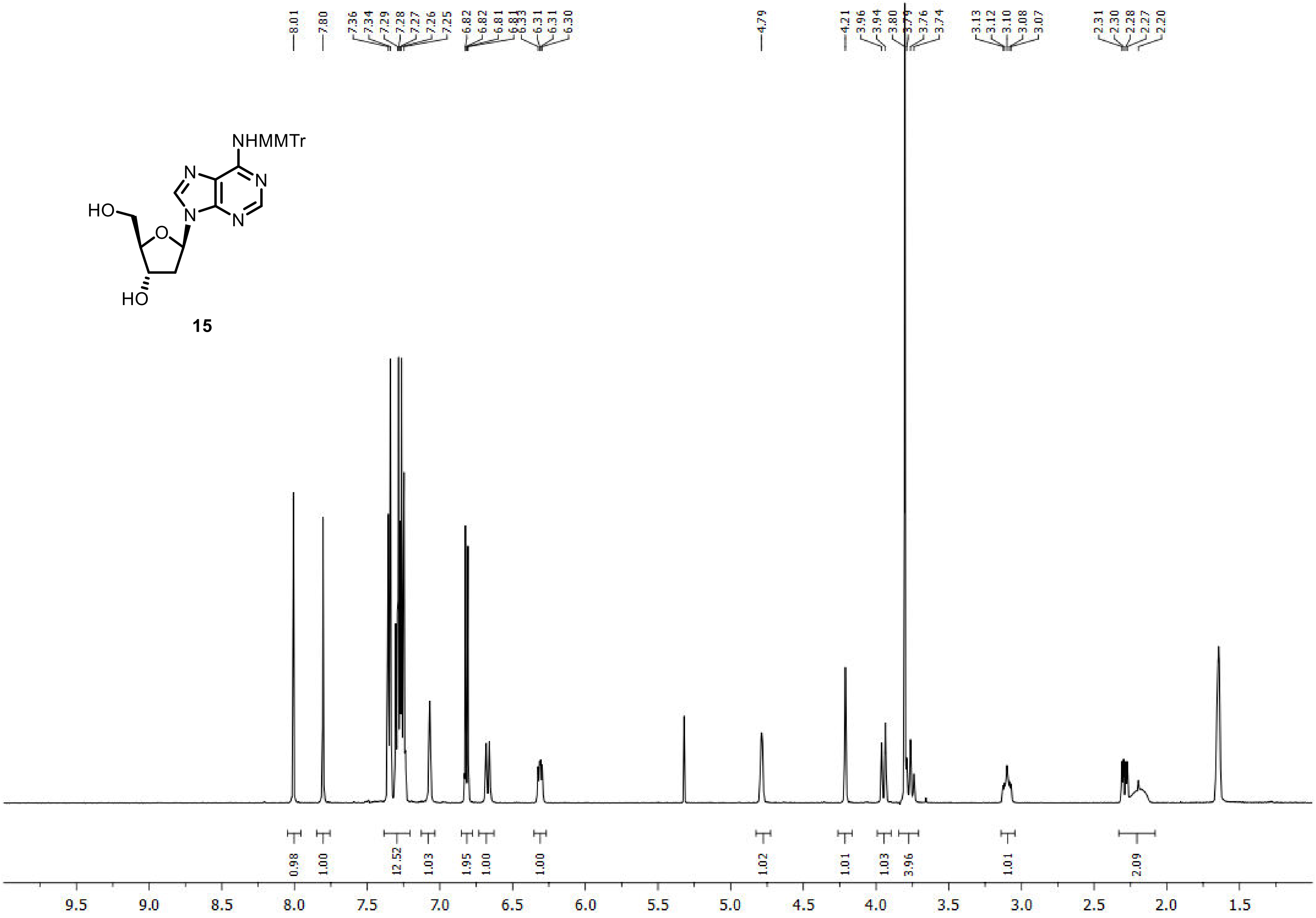

15

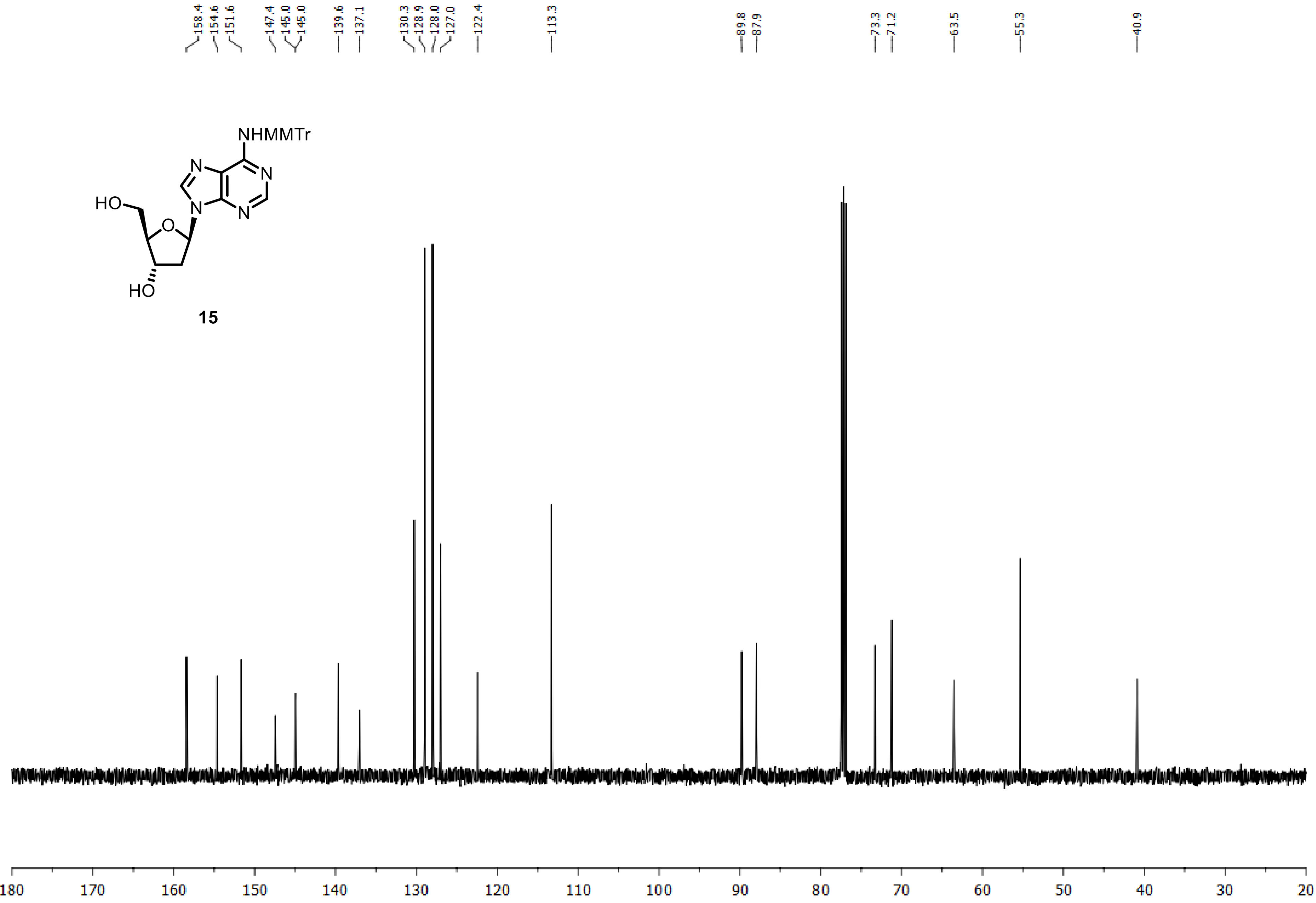

158.4
154.6
151.6
147.4
145.0
145.0
139.6
137.1
130.3
128.9
128.0
127.0
122.4
113.3
89.8
87.9
73.3
71.2
63.5
55.3
40.9
NHMMTr
N
N
N
N
HO
O
HO
15
180
170
160
150
140
130
120
110
100
90
80
70
60
50
40
30
20

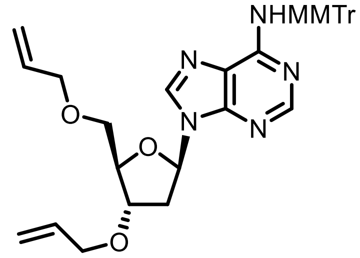
NHMMTr
N
N
N
N
O
O
O

16

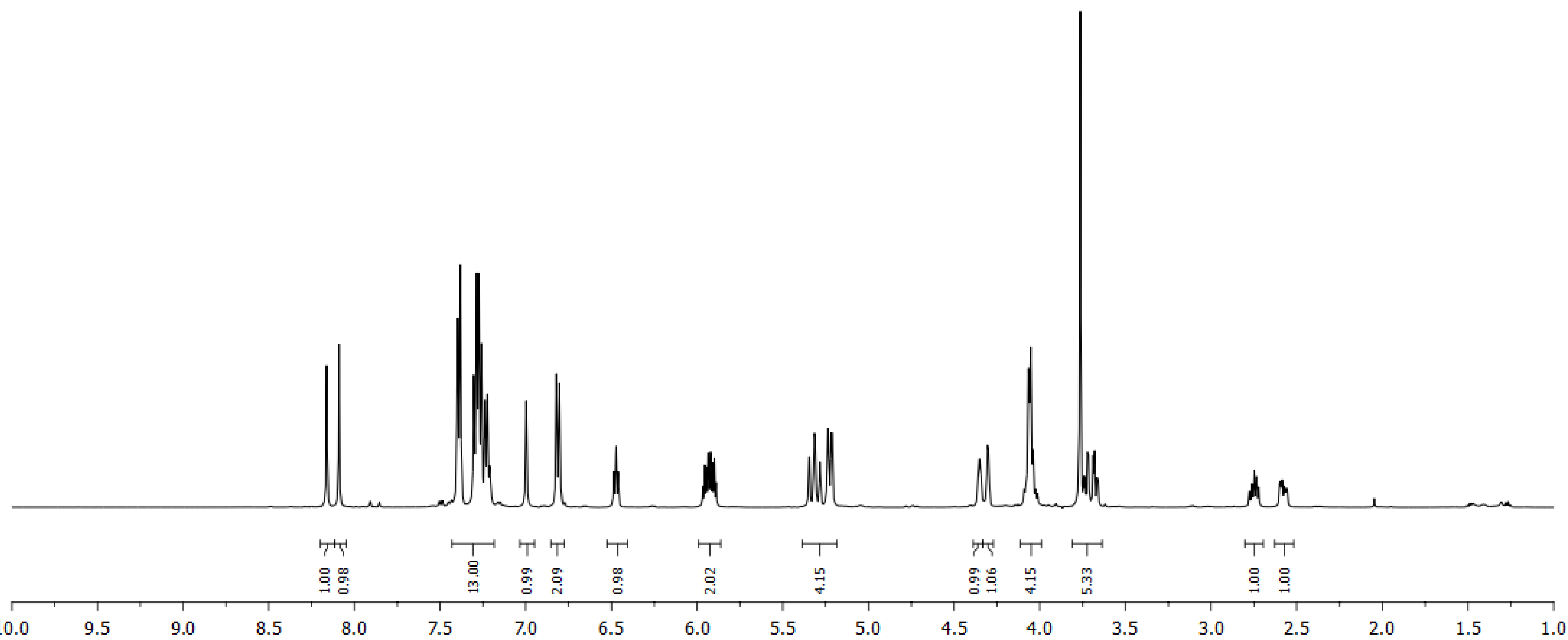
8.16 8.09 7.40 7.38 7.30 7.29 7.27 7.26 7.24 7.22 7.21 7.00 6.82 6.80 6.49 6.47 6.46 5.96 5.94 5.93 5.92 5.91 5.90 5.35 5.31 5.31 5.24 5.23 5.21 5.21 4.36 4.35 4.35 4.31 4.30 4.06 4.05 3.76 3.74 3.72 3.72 3.69 3.68 2.78 2.76 2.75 2.73 2.72 2.60 2.59 2.59 2.58 2.57 2.57 2.56 2.56
1.00 0.98 13.00 0.99 2.09 0.98 2.02 4.15 0.99 1.06 4.15 5.33 1.00 1.00
10.0 9.5 9.0 8.5 8.0 7.5 7.0 6.5 6.0 5.5 5.0 4.5 4.0 3.5 3.0 2.5 2.0 1.5 1.0

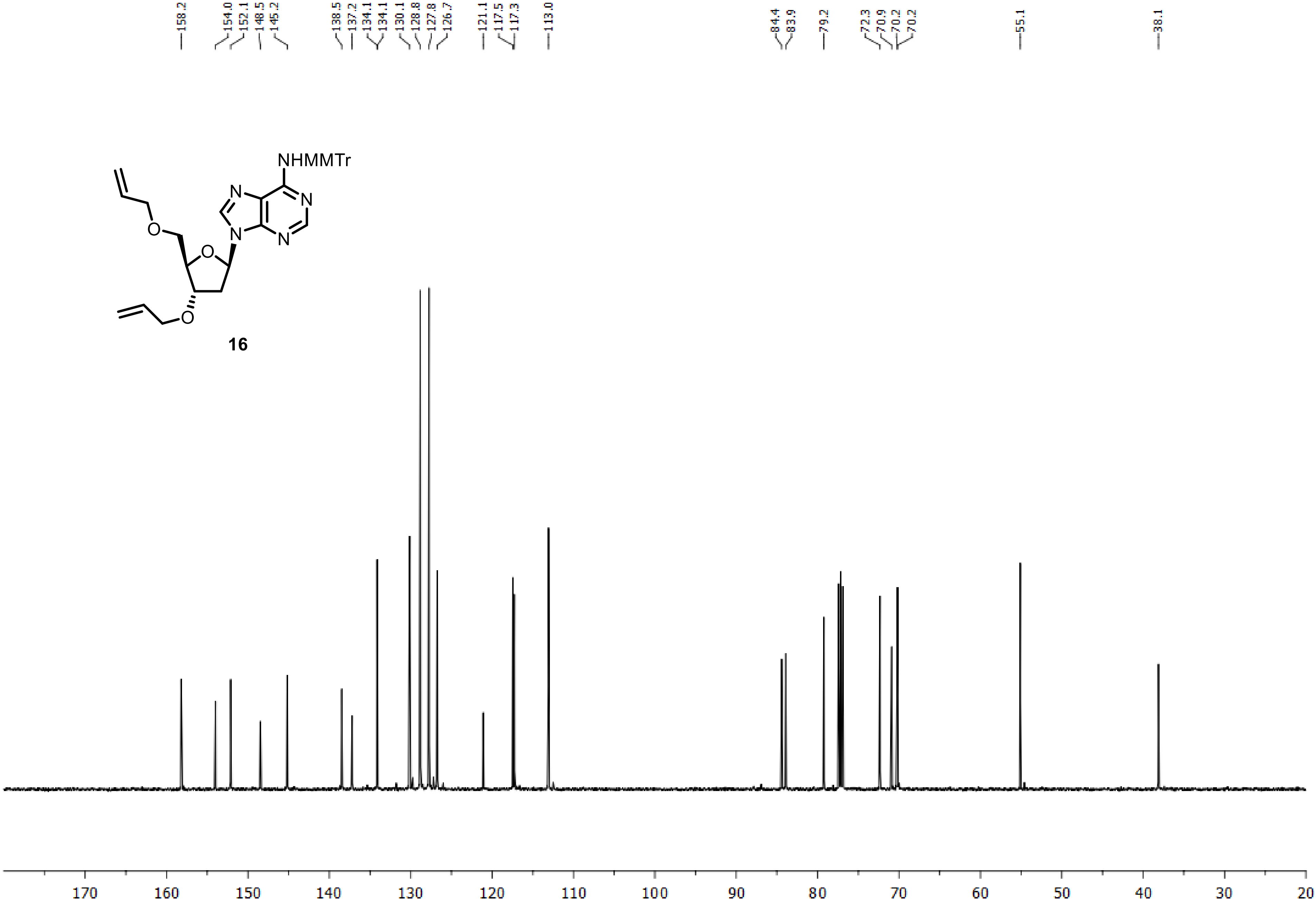
158.2
154.0
152.1
148.5
145.2
138.5
137.2
134.1
134.1
130.1
128.8
127.8
126.7
121.1
117.5
117.3
113.0
84.4
83.9
79.2
72.3
70.9
70.2
70.2
55.1
38.1
NHMMTr
N
N
N
N
O
O
O
16
170 160 150 140 130 120 110 100 90 80 70 60 50 40 30 20

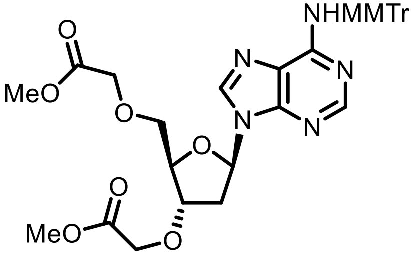
NHMMTr
MeO
O
N
MeO

17

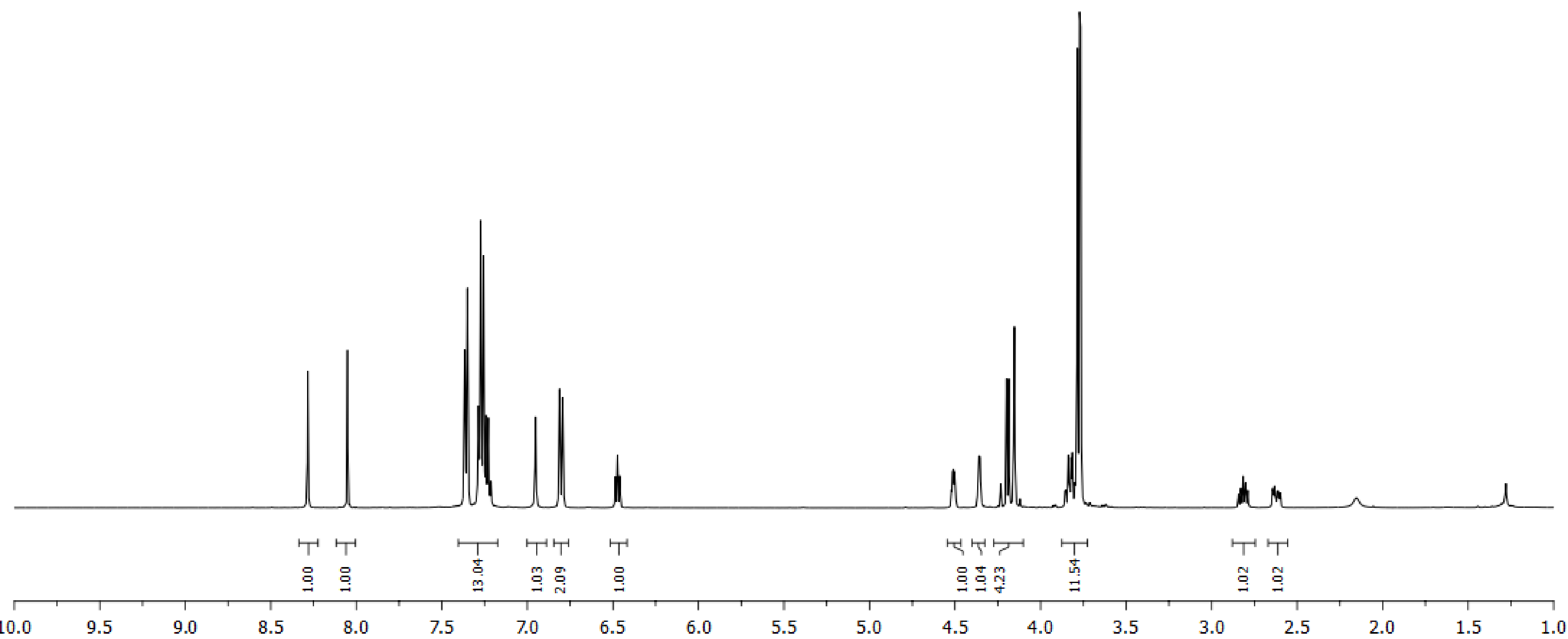
8.28
8.05
7.37
7.35
7.29
7.27
7.26
7.24
7.23
7.21
6.95
6.81
6.79
6.49
6.47
6.46
4.52
4.51
4.51
4.50
4.50
4.37
4.36
4.35
4.35
4.23
4.20
4.19
4.15
4.15
3.84
3.82
3.81
3.78
3.77
3.77
2.84
2.83
2.82
2.80
2.79
2.64
2.64
2.63
2.63
2.62
2.61
2.60
2.60
1.00
1.00
13.04
1.03
2.09
1.00
1.00
1.04
4.23
11.54
1.02
1.02
10.0
9.5
9.0
8.5
8.0
7.5
7.0
6.5
6.0
5.5
5.0
4.5
4.0
3.5
3.0
2.5
2.0
1.5
1.0

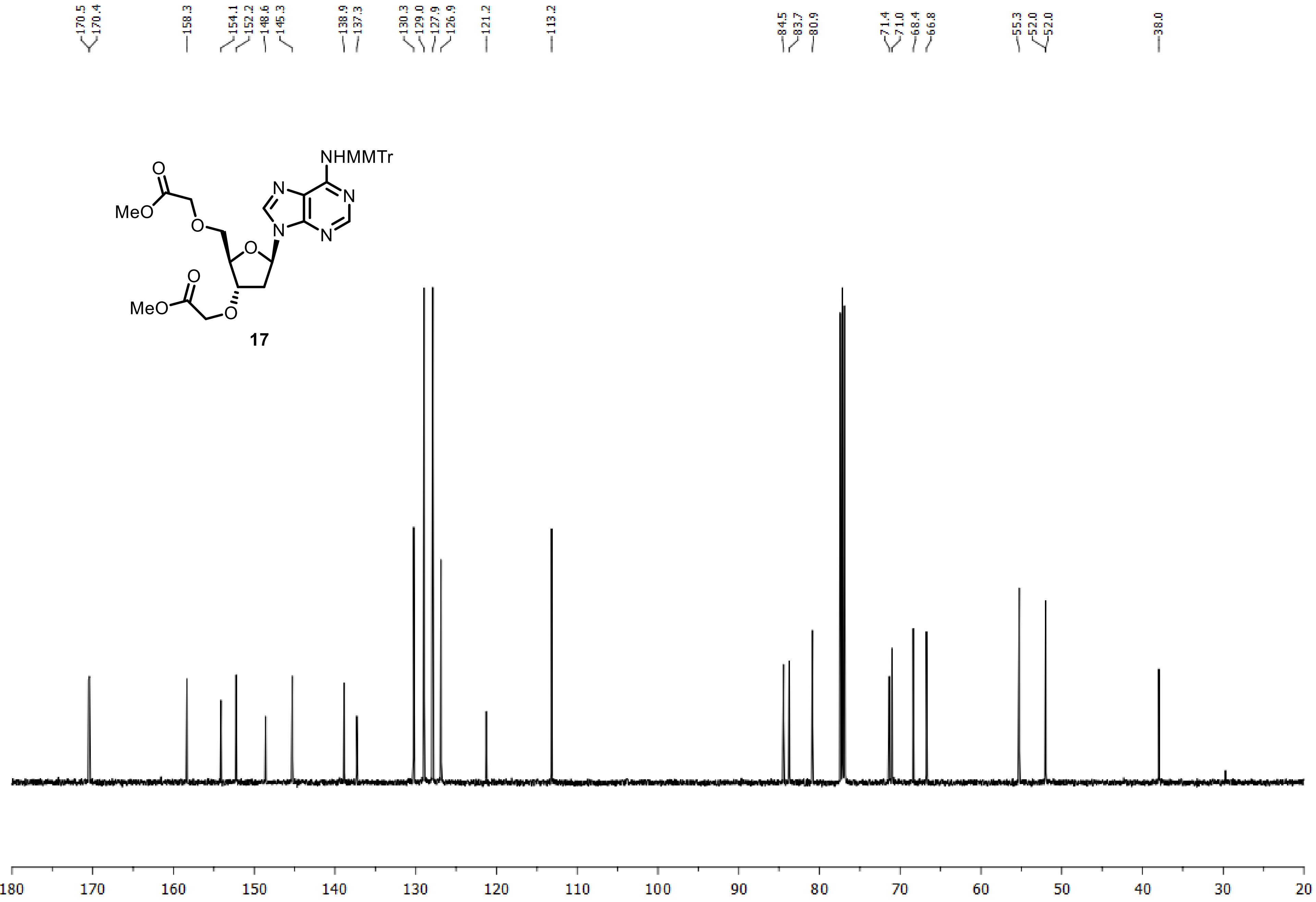
170.5
170.4
158.3
154.1
152.2
148.6
145.3
138.9
137.3
130.3
129.0
127.9
126.9
121.2
113.2
84.5
83.7
80.9
71.4
71.0
68.4
66.8
55.3
52.0
52.0
38.0
NHMMTr
MeO
MeO
17
180
170
160
150
140
130
120
110
100
90
80
70
60
50
40
30
20

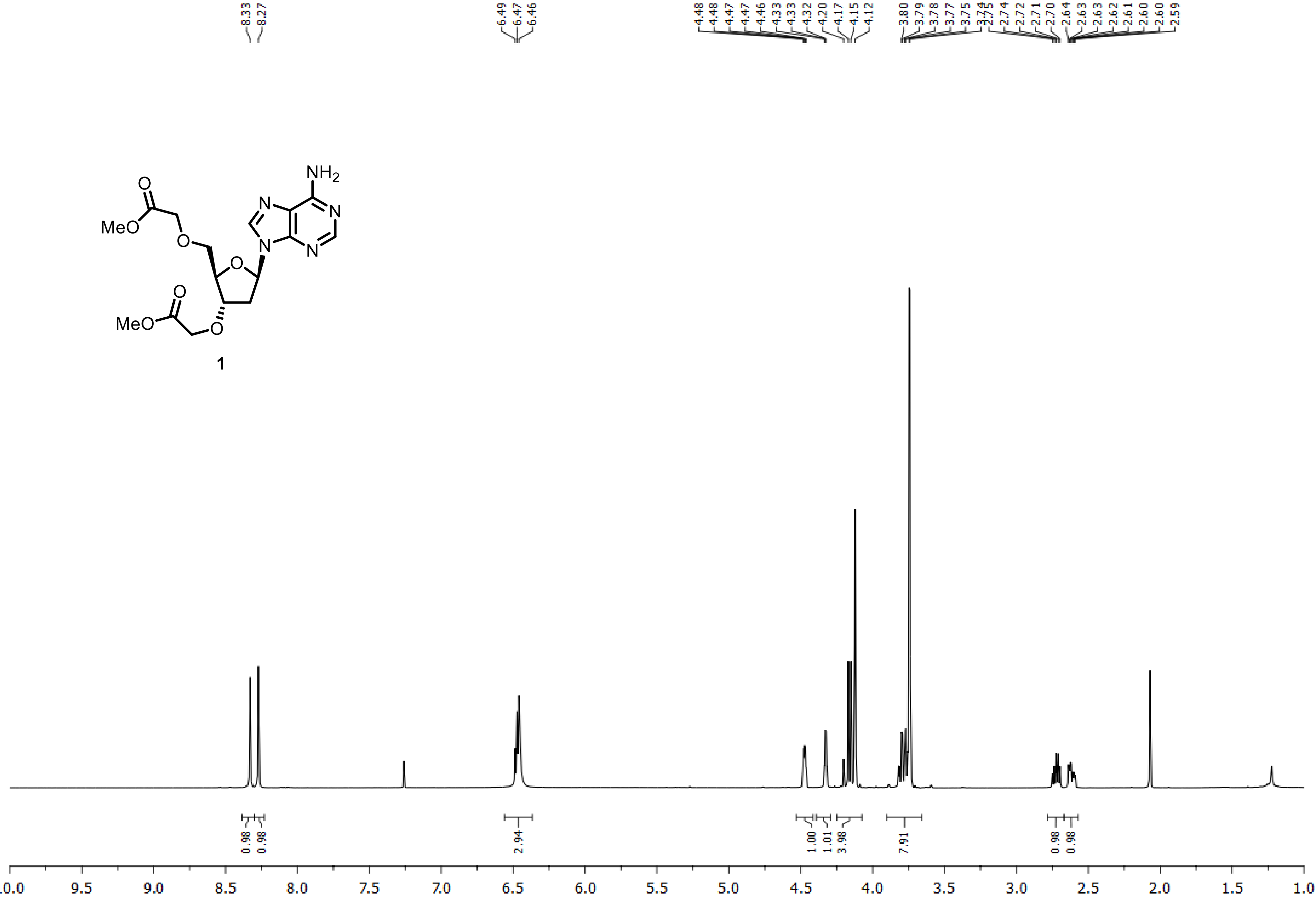

MeO
O
O
O
N
N
N
N
NH2
MeO
O
O
1
10.0
9.5
9.0
8.5
8.0
7.5
7.0
6.5
6.0
5.5
5.0
4.5
4.0
3.5
3.0
2.5
2.0
1.5
1.0

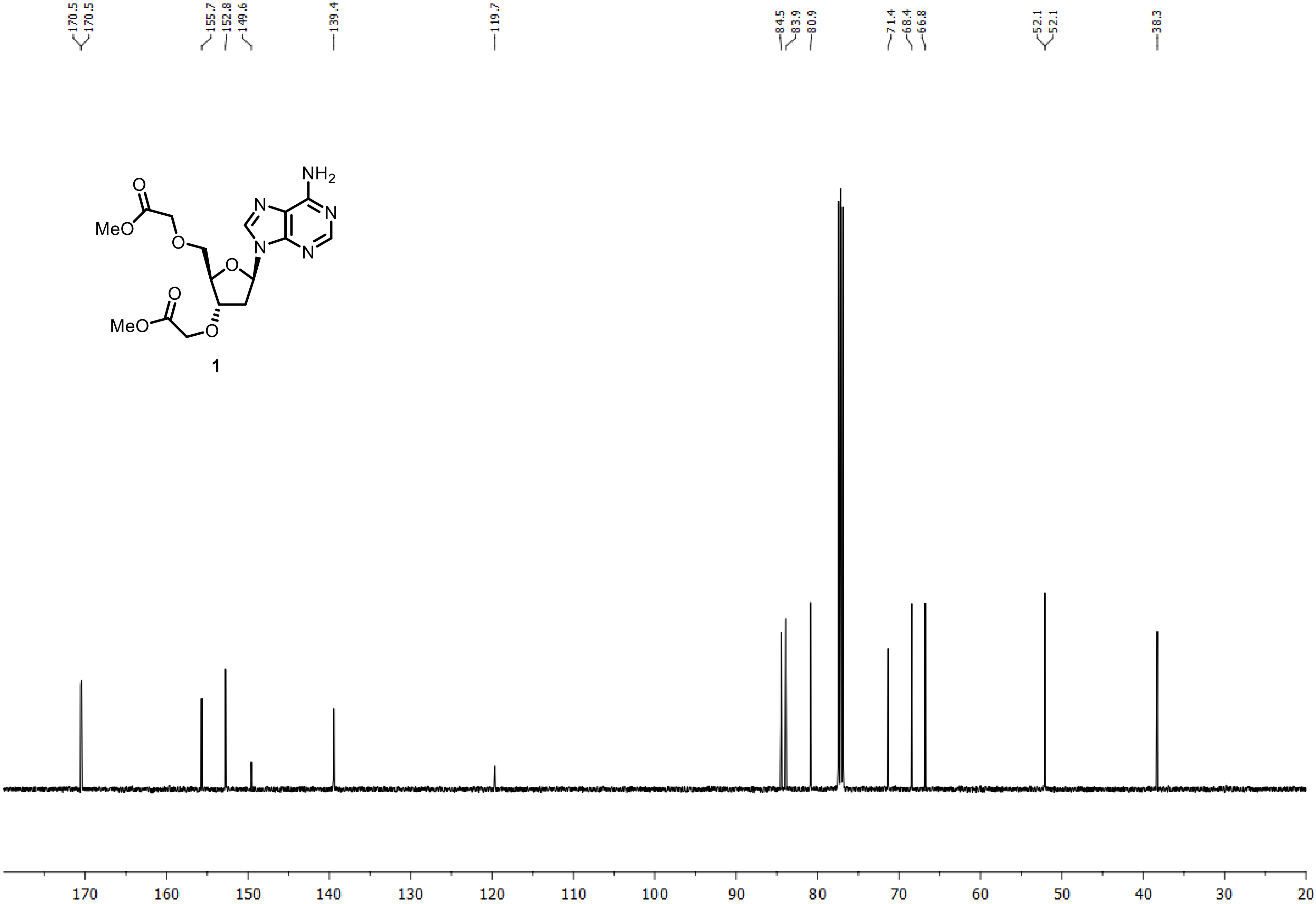

170.5
170.5
155.7
152.8
149.6
139.4
119.7
84.5
83.9
80.9
71.4
68.4
66.8
52.1
52.1
38.3
NH2
N
N
N
N
O
MeO
O
O
O
MeO
O
1
170
160
150
140
130
120
110
100
90
80
70
60
50
40
30
20

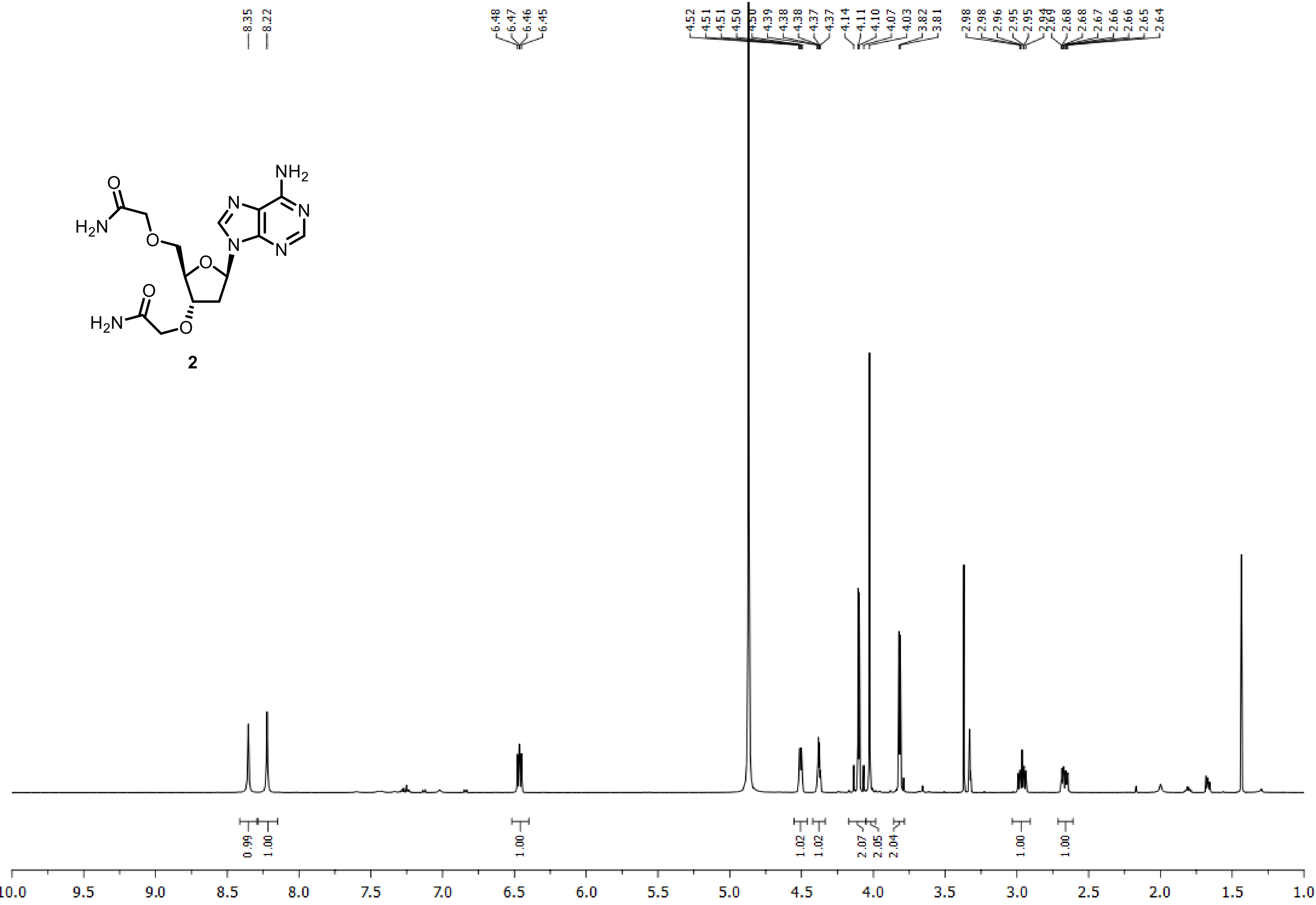
8.35
8.22
6.48
6.47
6.46
6.45
4.52
4.51
4.51
4.50
4.50
4.39
4.38
4.38
4.37
4.37
4.14
4.11
4.10
4.07
4.03
3.82
3.81
2.98
2.98
2.96
2.95
2.95
2.94
2.69
2.68
2.68
2.67
2.66
2.66
2.65
2.64
NH2
N
N
N
N
O
H2N
O
O
H2N
O
O
2
0.99
1.00
1.00
1.02
1.02
2.07
2.05
2.04
1.00
1.00
10.0
9.5
9.0
8.5
8.0
7.5
7.0
6.5
6.0
5.5
5.0
4.5
4.0
3.5
3.0
2.5
2.0
1.5
1.0

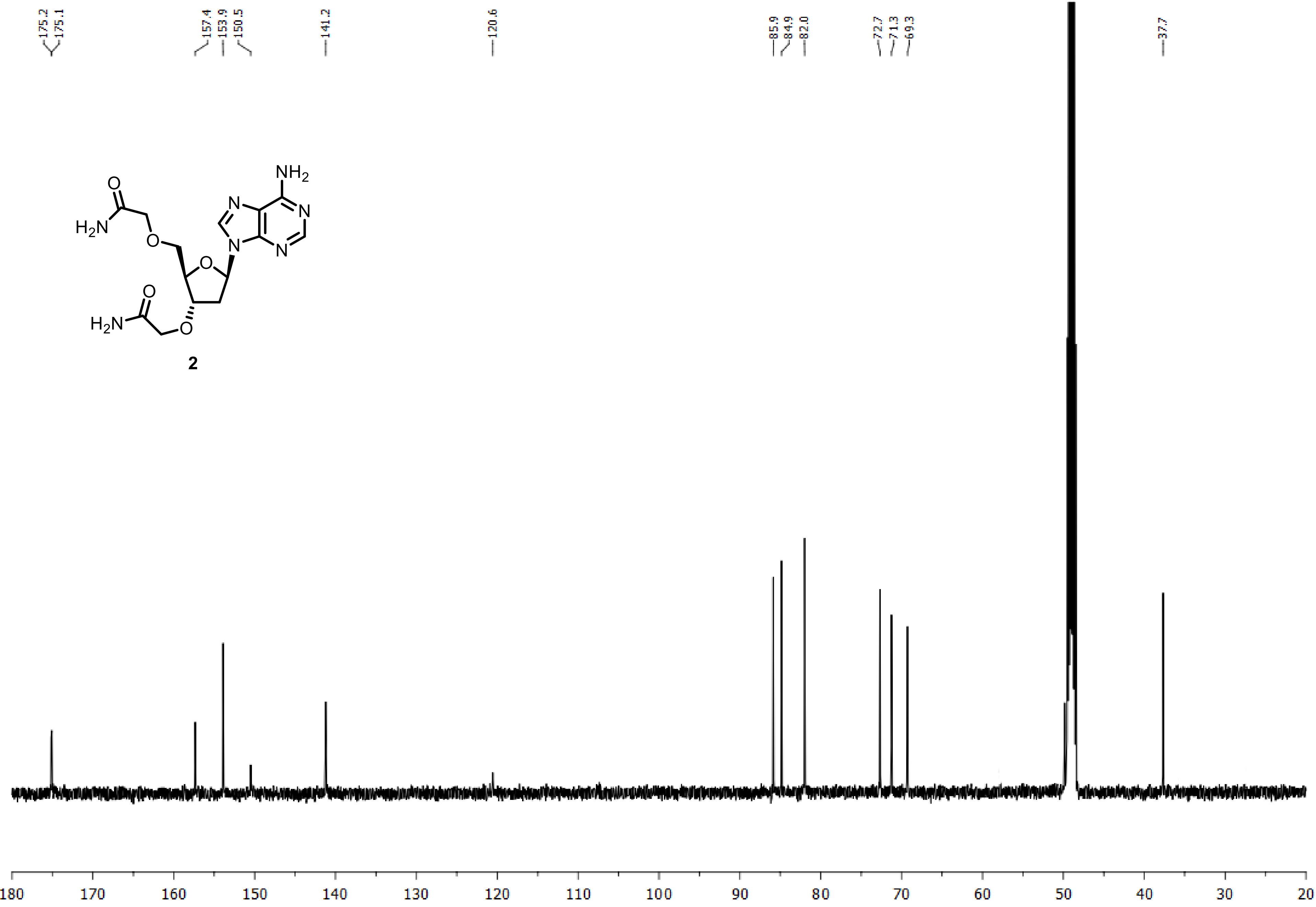
175.2
175.1
157.4
153.9
150.5
141.2
120.6
85.9
84.9
82.0
72.7
71.3
69.3
37.7
NH2
N
O
H2N
2
180 170 160 150 140 130 120 110 100 90 80 70 60 50 40 30 20

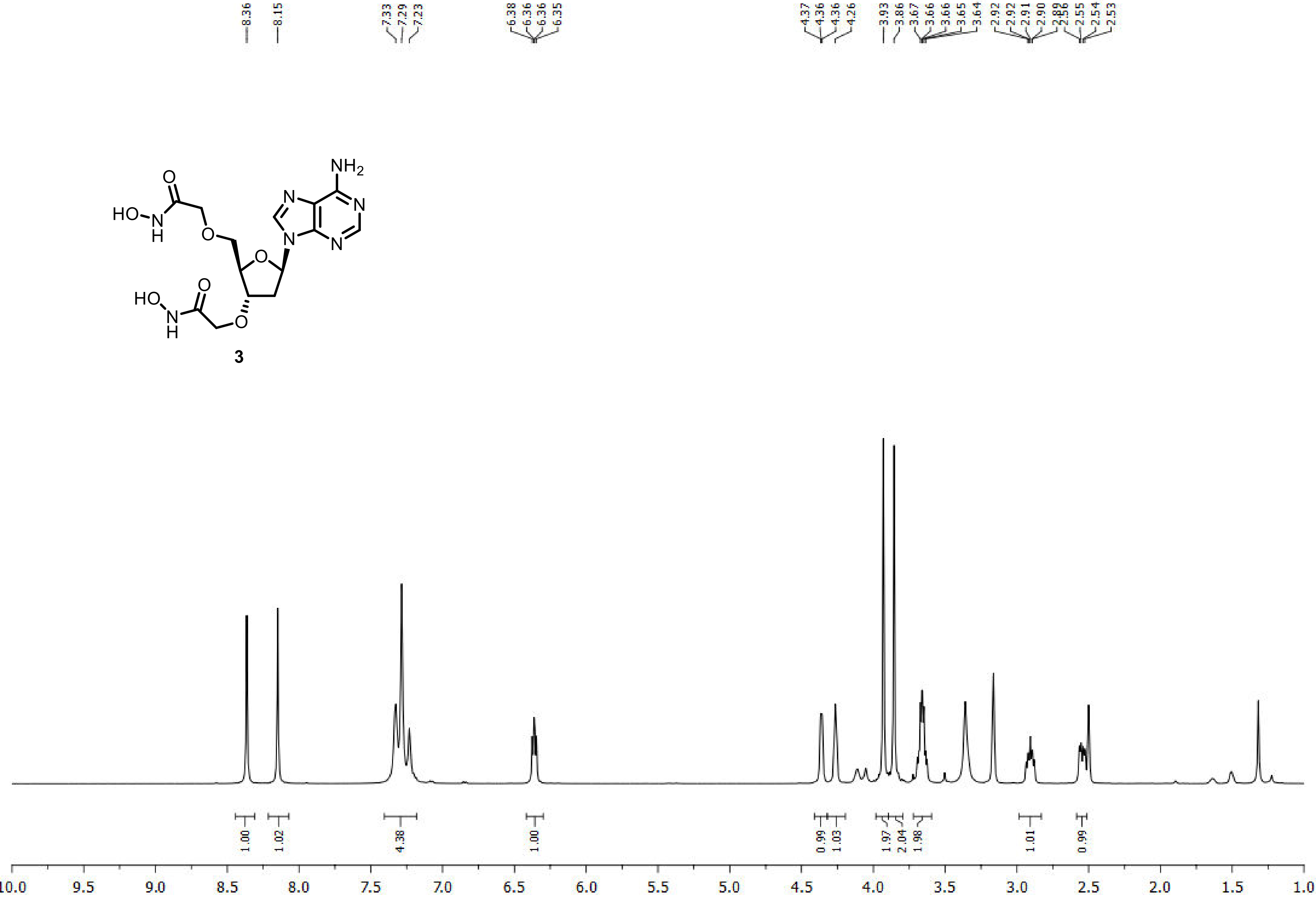
8.36
8.15
7.33
7.29
7.23
6.38
6.36
6.36
6.35
4.37
4.36
4.36
4.26
3.93
3.86
3.67
3.66
3.66
3.65
3.64
2.92
2.92
2.91
2.90
2.89
2.56
2.55
2.54
2.53
NH2
HO
N
H
O
N
N
N
N
3
1.00
1.02
4.38
1.00
0.99
1.03
1.97
2.04
1.98
1.01
0.99
10.0
9.5
9.0
8.5
8.0
7.5
7.0
6.5
6.0
5.5
5.0
4.5
4.0
3.5
3.0
2.5
2.0
1.5
1.0

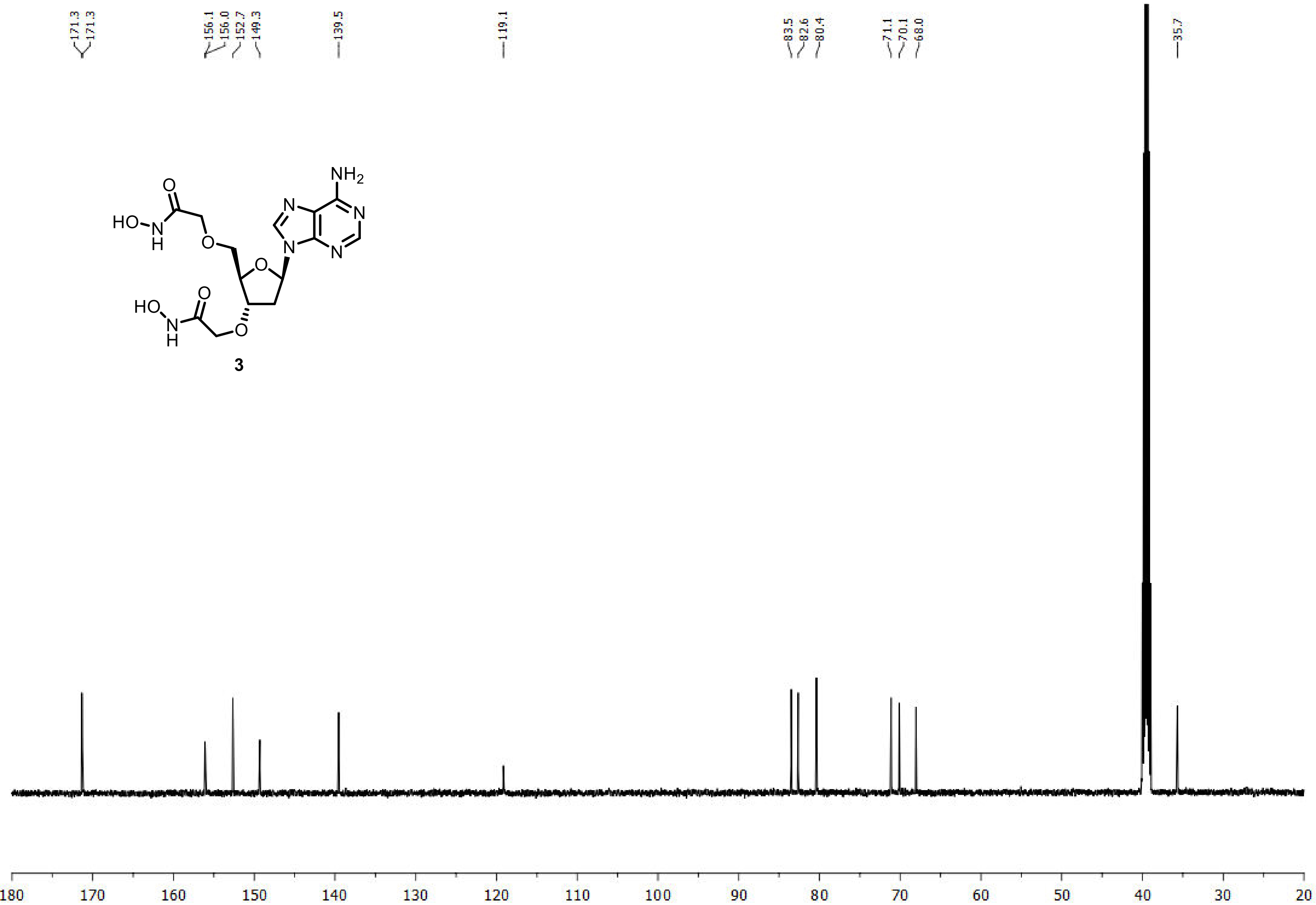

171.3
171.3
156.1
156.0
152.7
149.3
139.5
119.1
83.5
82.6
80.4
71.1
70.1
68.0
35.7
NH2
N
N
N
N
O
HO
N
H
O
O
O
HO
N
H
O
180
170
160
150
140
130
120
110
100
90
80
70
60
50
40
30
20

3

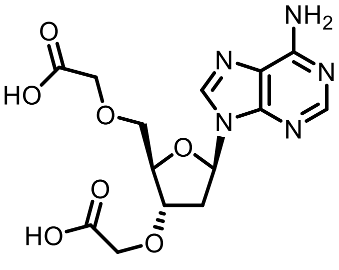

NH2
N
N
N
N
O
O
HO
O
O
O
HO

4

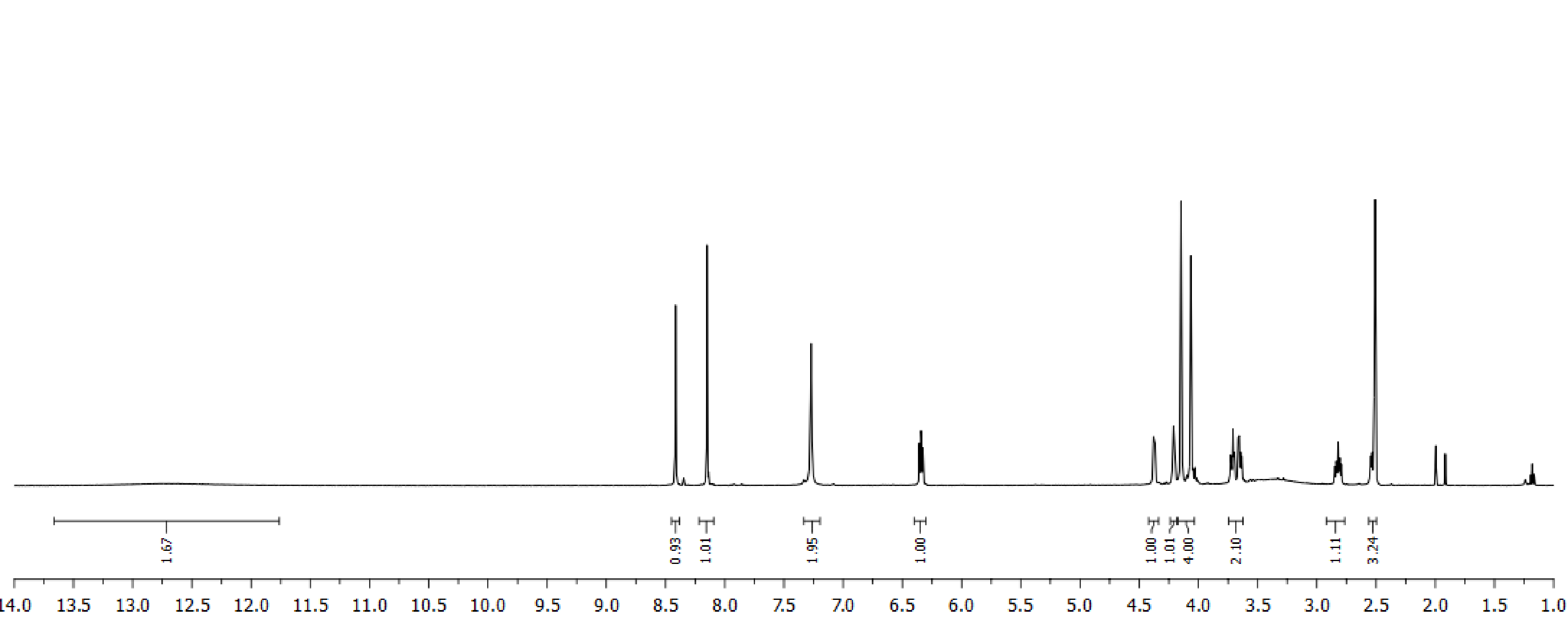

12.76
8.42
8.15
7.27
6.36 6.34 6.34 6.33
4.38 4.37 4.37 4.21 4.21 4.15 4.06
3.71 3.70 3.66 3.65 2.85 2.84 2.83 2.82 2.81 2.80 2.79 2.55 2.54 2.54 2.53
1.67
0.93
1.01
1.95
1.00
1.00 1.01 4.00
2.10
1.11
3.24
14.0 13.5 13.0 12.5 12.0 11.5 11.0 10.5 10.0 9.5 9.0 8.5 8.0 7.5 7.0 6.5 6.0 5.5 5.0 4.5 4.0 3.5 3.0 2.5 2.0 1.5 1.0

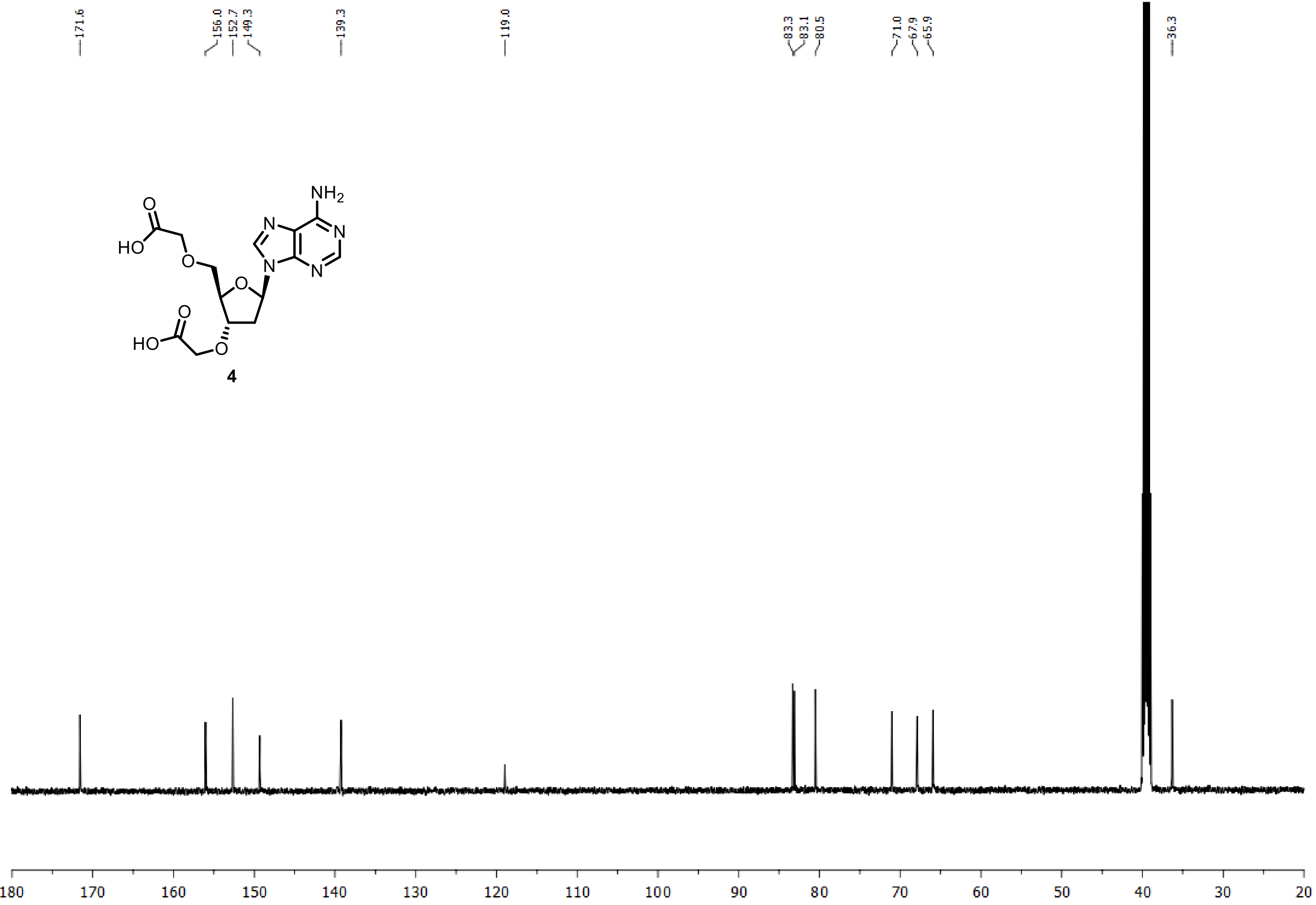
171.6
156.0
152.7
149.3
139.3
119.0
83.3
83.1
80.5
71.0
67.9
65.9
36.3
NH2
N
HO
O
4
180 170 160 150 140 130 120 110 100 90 80 70 60 50 40 30 20

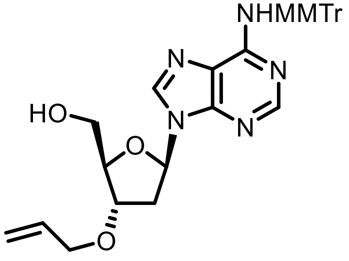

18

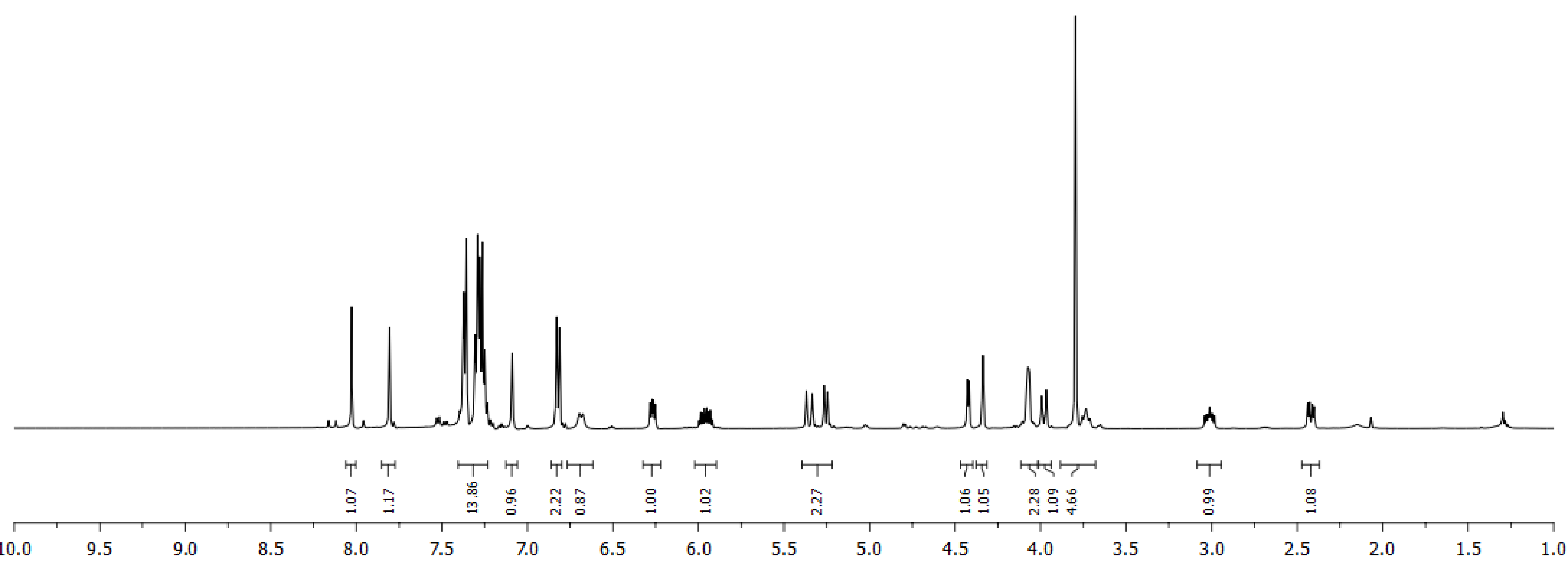

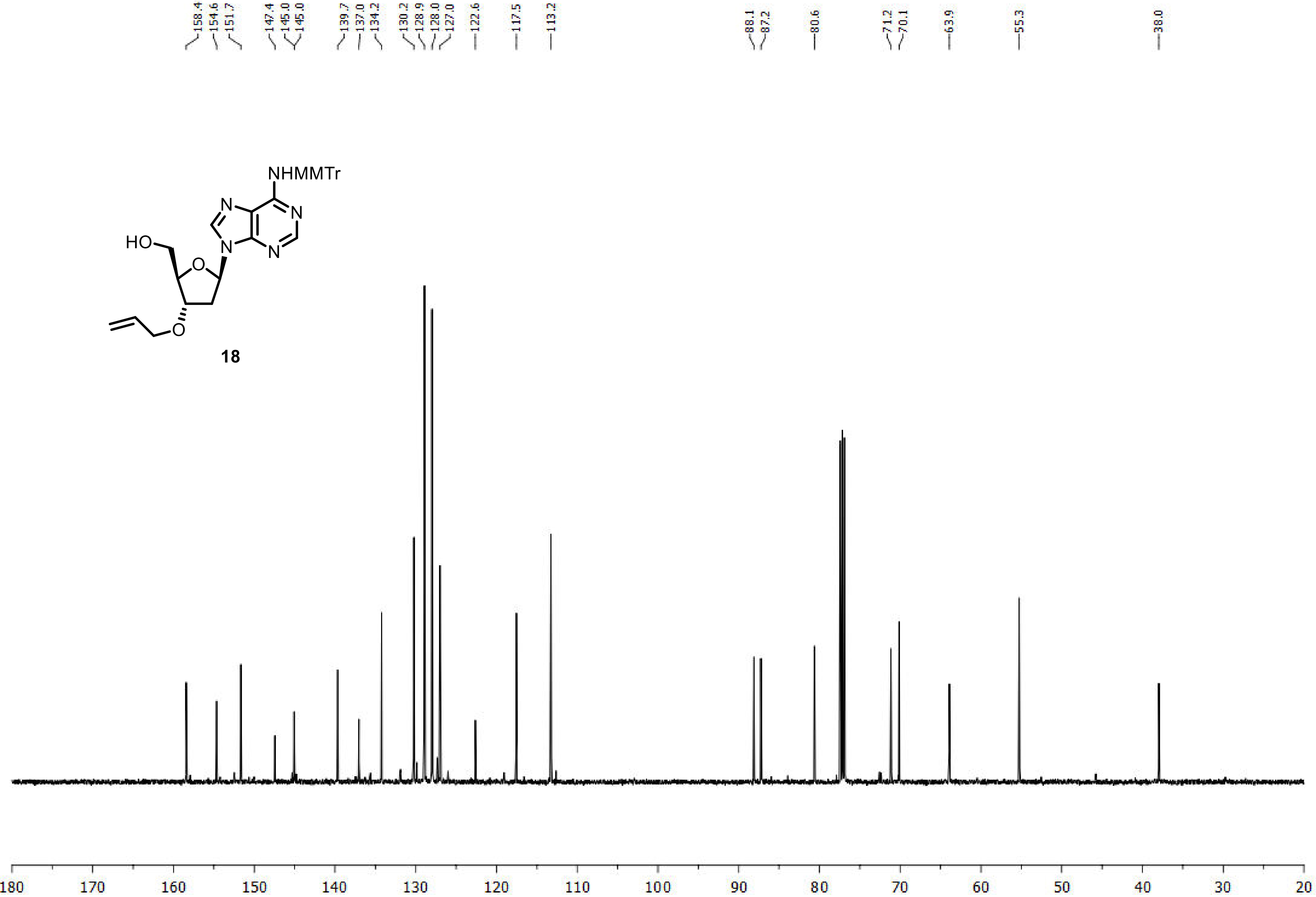
158.4
154.6
151.7
147.4
145.0
145.0
139.7
137.0
134.2
130.2
128.9
128.0
127.0
122.6
117.5
113.2
88.1
87.2
80.6
71.2
70.1
63.9
55.3
38.0
NHMMTr
N
N
N
N
HO
O
O
18
180
170
160
150
140
130
120
110
100
90
80
70
60
50
40
30
20

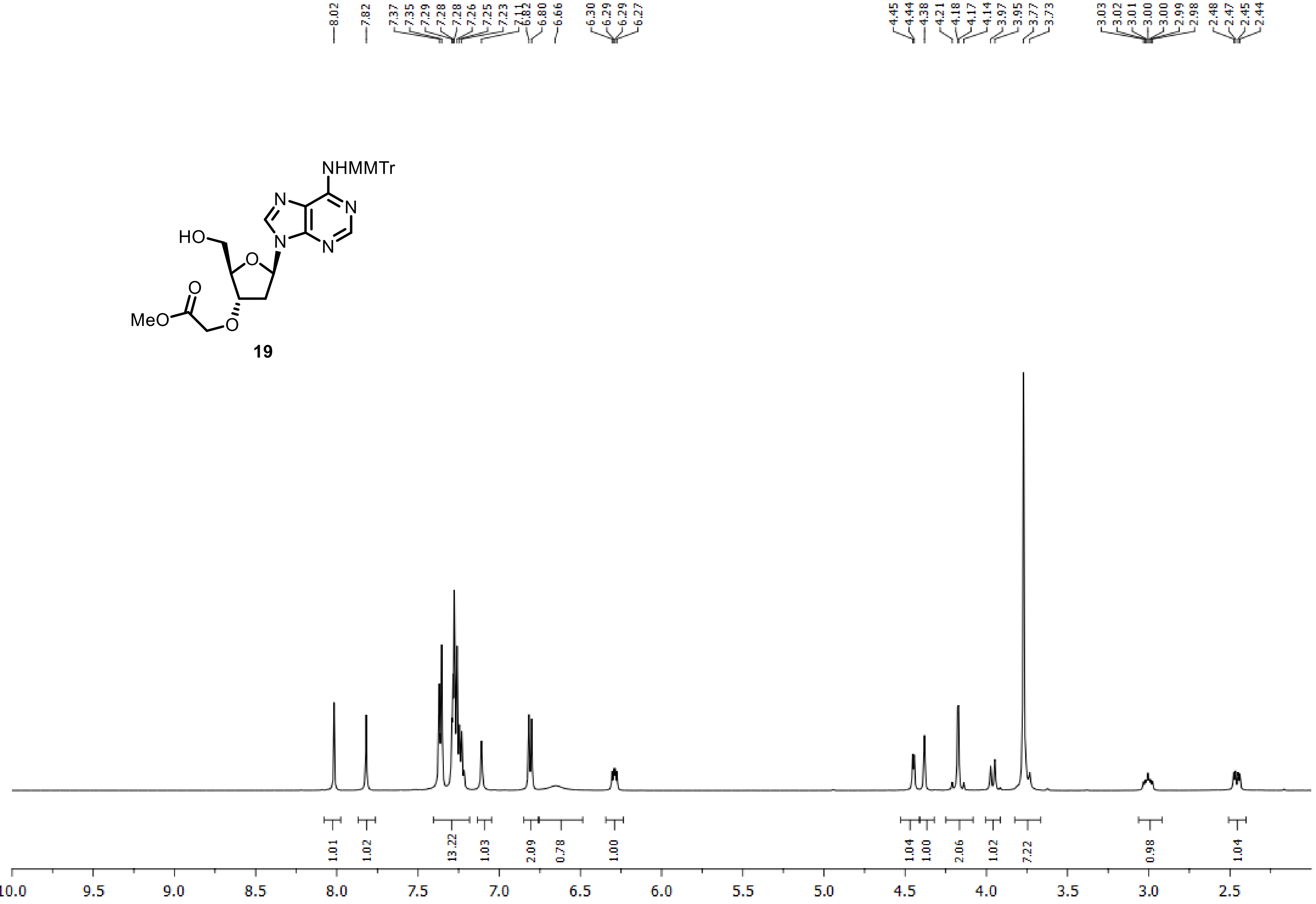
NHMMTr
N
N
N
N
HO
O
O
MeO
O
19
10.0 9.5 9.0 8.5 8.0 7.5 7.0 6.5 6.0 5.5 5.0 4.5 4.0 3.5 3.0 2.5

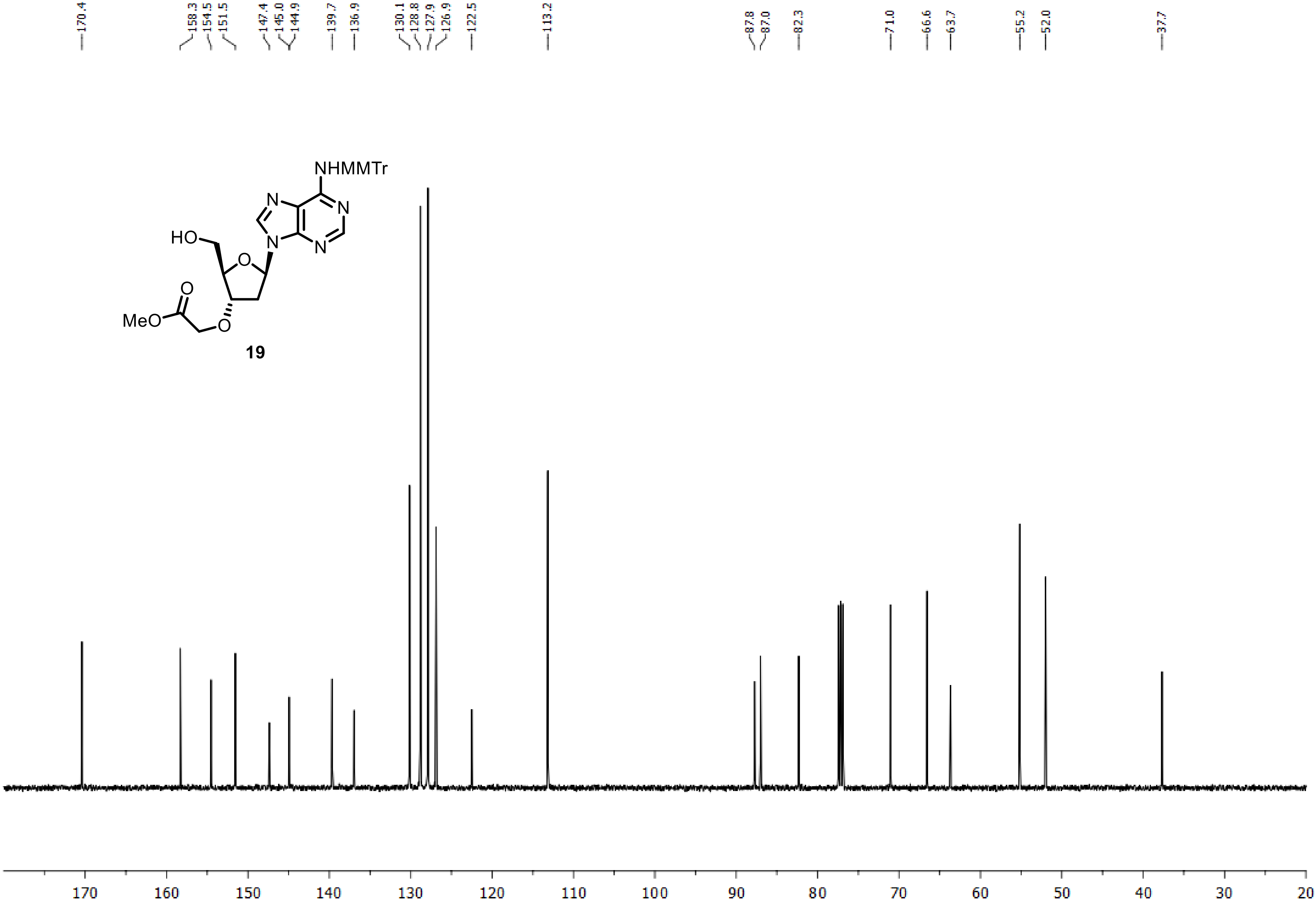
170.4
158.3
154.5
151.5
147.4
145.0
144.9
139.7
136.9
130.1
128.8
127.9
126.9
122.5
113.2
87.8
87.0
82.3
71.0
66.6
63.7
55.2
52.0
37.7
NHMMTr
N
N
N
N
HO
O
O
MeO
O
19
170 160 150 140 130 120 110 100 90 80 70 60 50 40 30 20

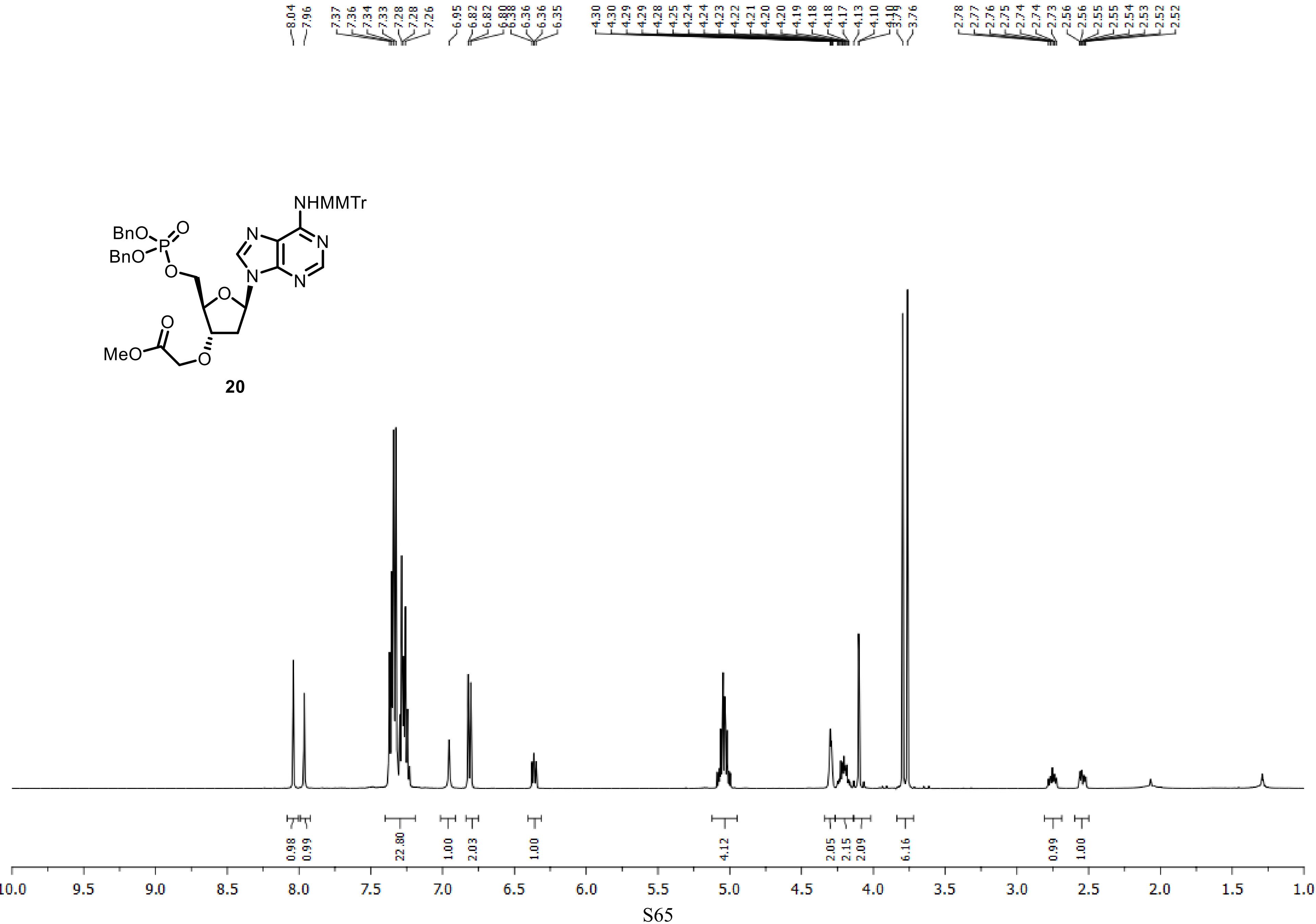
NHMMTr
BnO
BnO
MeO
20

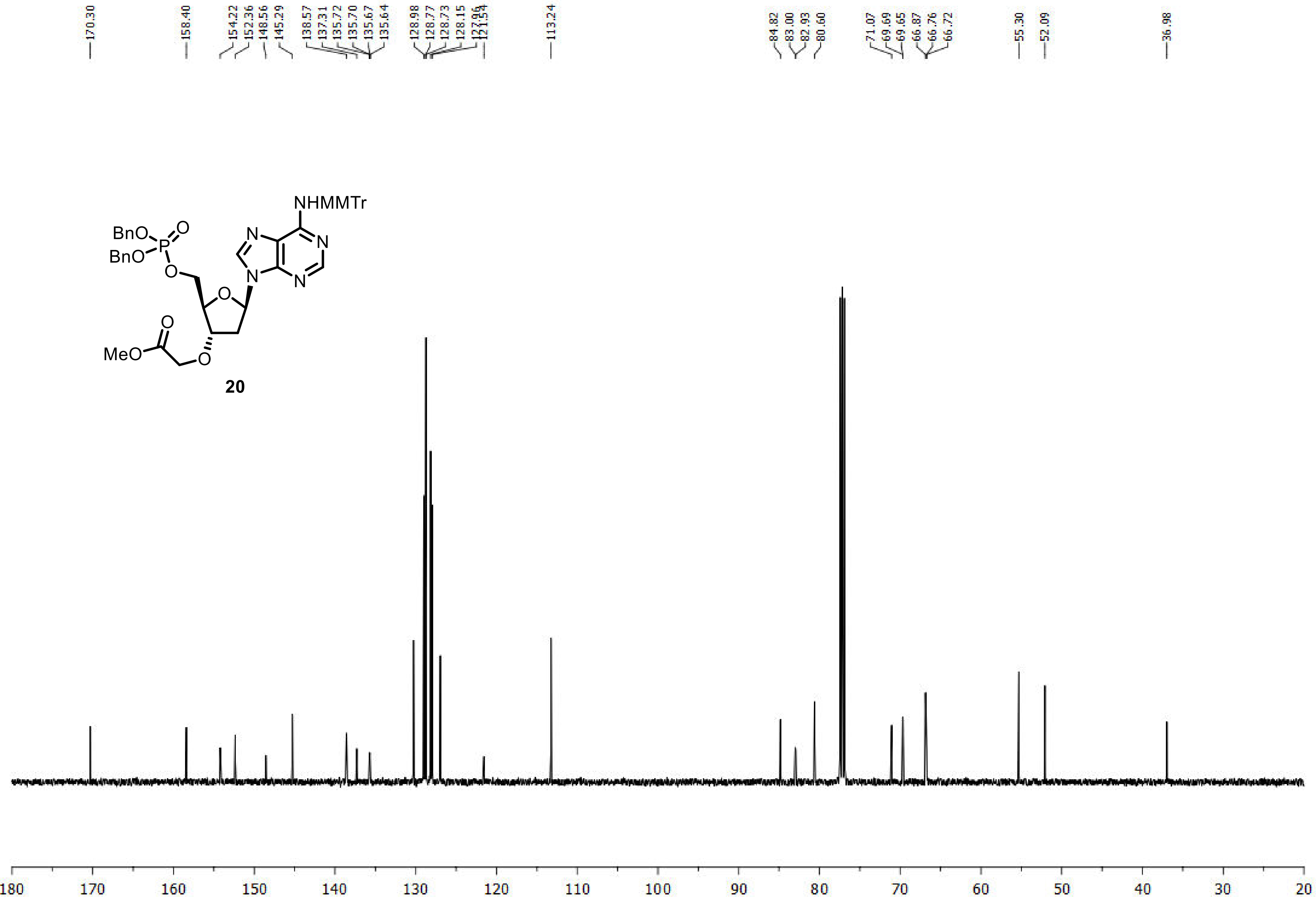
170.30
158.40
154.22
152.36
148.56
145.29
138.57
137.31
135.72
135.70
135.67
135.64
128.98
128.77
128.73
128.15
121.54
113.24
84.82
83.00
82.93
80.60
71.07
69.69
69.65
66.87
66.76
66.72
55.30
52.09
36.98
NHMMTr
BnO
BnO
P
O
N
MeO
20
180
170
160
150
140
130
120
110
100
90
80
70
60
50
40
30
20

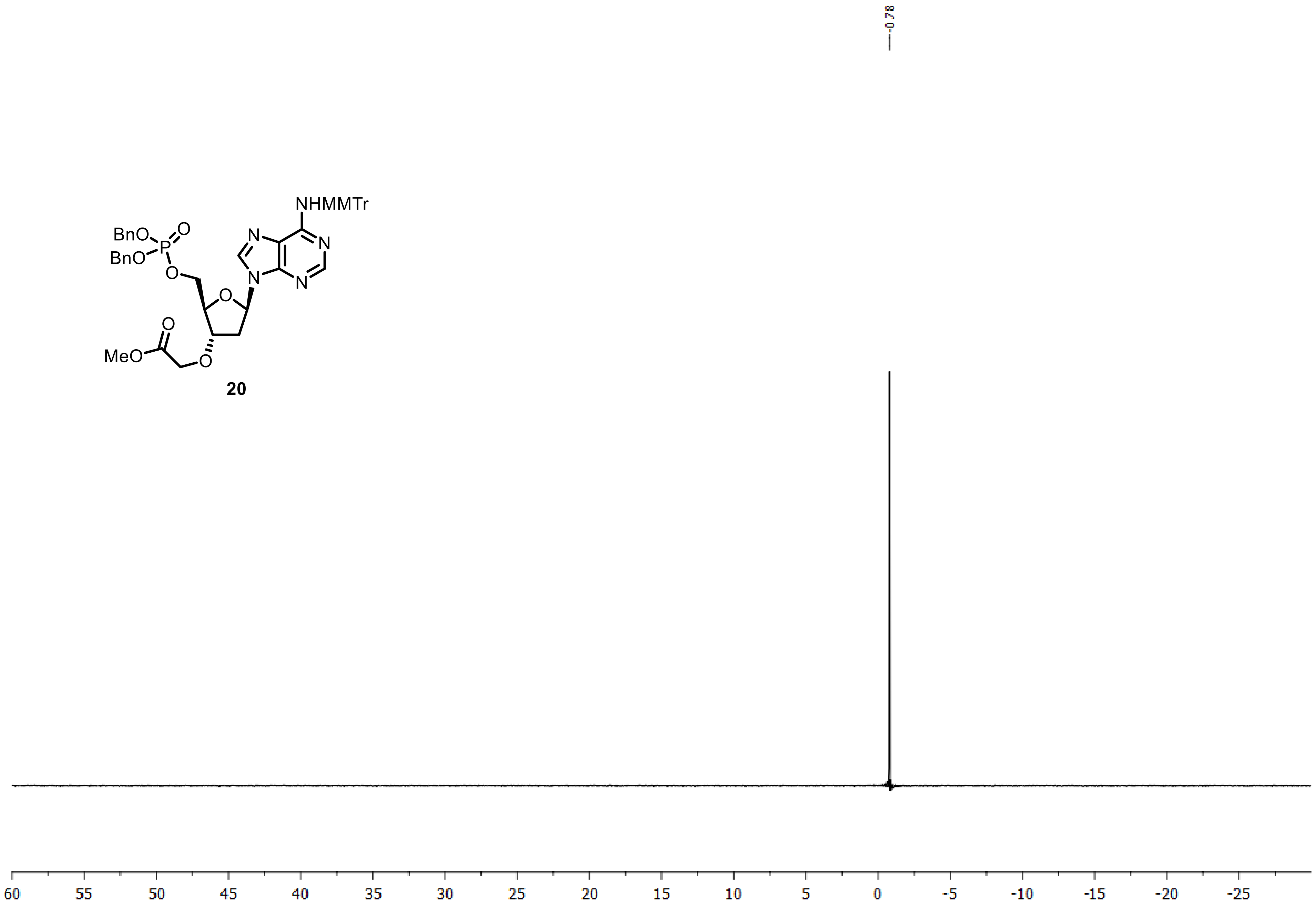
-0.78
NHMMTr
BnO
BnO
MeO
20
60 55 50 45 40 35 30 25 20 15 10 5 0 -5 -10 -15 -20 -25

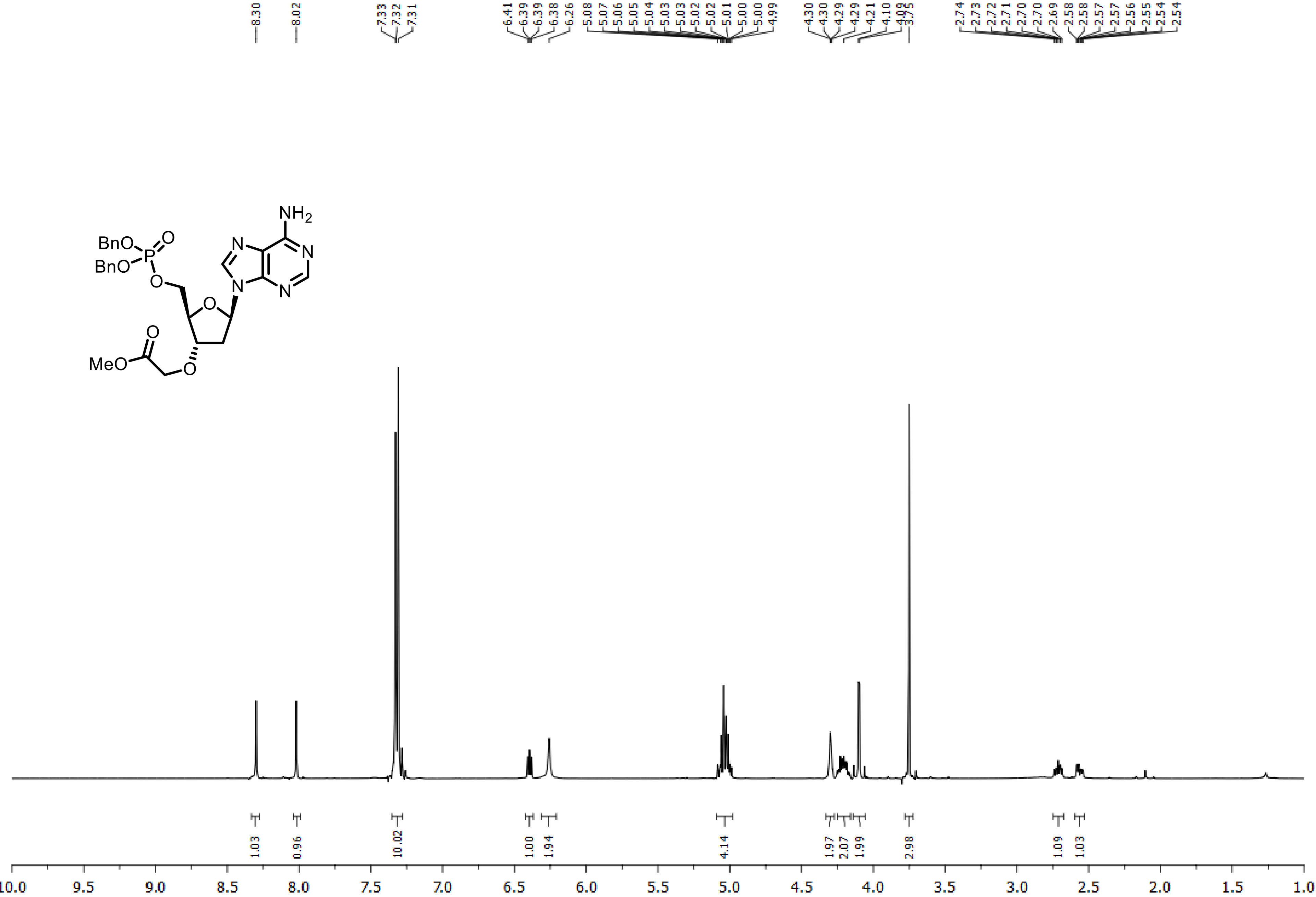
8.30
8.02
7.33 7.32 7.31
6.41 6.39 6.39 6.38 6.26
5.08 5.07 5.06 5.05 5.04 5.03 5.03 5.02 5.02 5.01 5.00 5.00 4.99
4.30 4.30 4.29 4.29 4.21 4.10 4.09 3.75
2.74 2.73 2.72 2.71 2.70 2.70 2.69 2.58 2.58 2.57 2.57 2.56 2.55 2.54 2.54
NH2
BnO
BnO
P
O
N
MeO
1.03 0.96 10.02 1.00 1.94 4.14 1.97 2.07 1.99 2.98 1.09 1.03
10.0 9.5 9.0 8.5 8.0 7.5 7.0 6.5 6.0 5.5 5.0 4.5 4.0 3.5 3.0 2.5 2.0 1.5 1.0

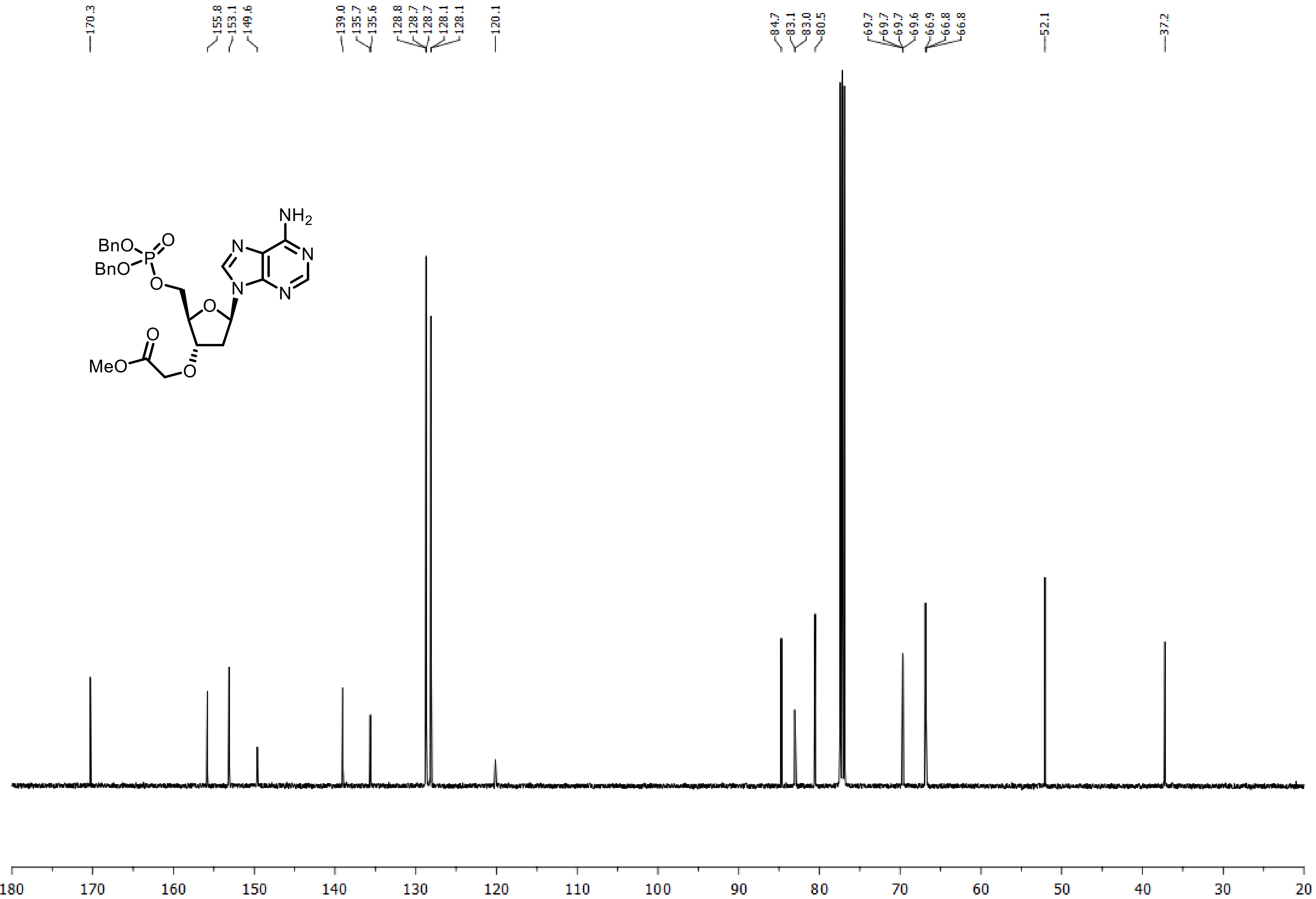
170.3
155.8
153.1
149.6
139.0
135.7
135.6
128.8
128.7
128.7
128.1
128.1
120.1
84.7
83.1
83.0
80.5
69.7
69.7
69.7
69.6
66.9
66.8
66.8
52.1
37.2
NH2
N
N
N
N
BnO
BnO
P
O
O
O
O
O
MeO
180 170 160 150 140 130 120 110 100 90 80 70 60 50 40 30 20

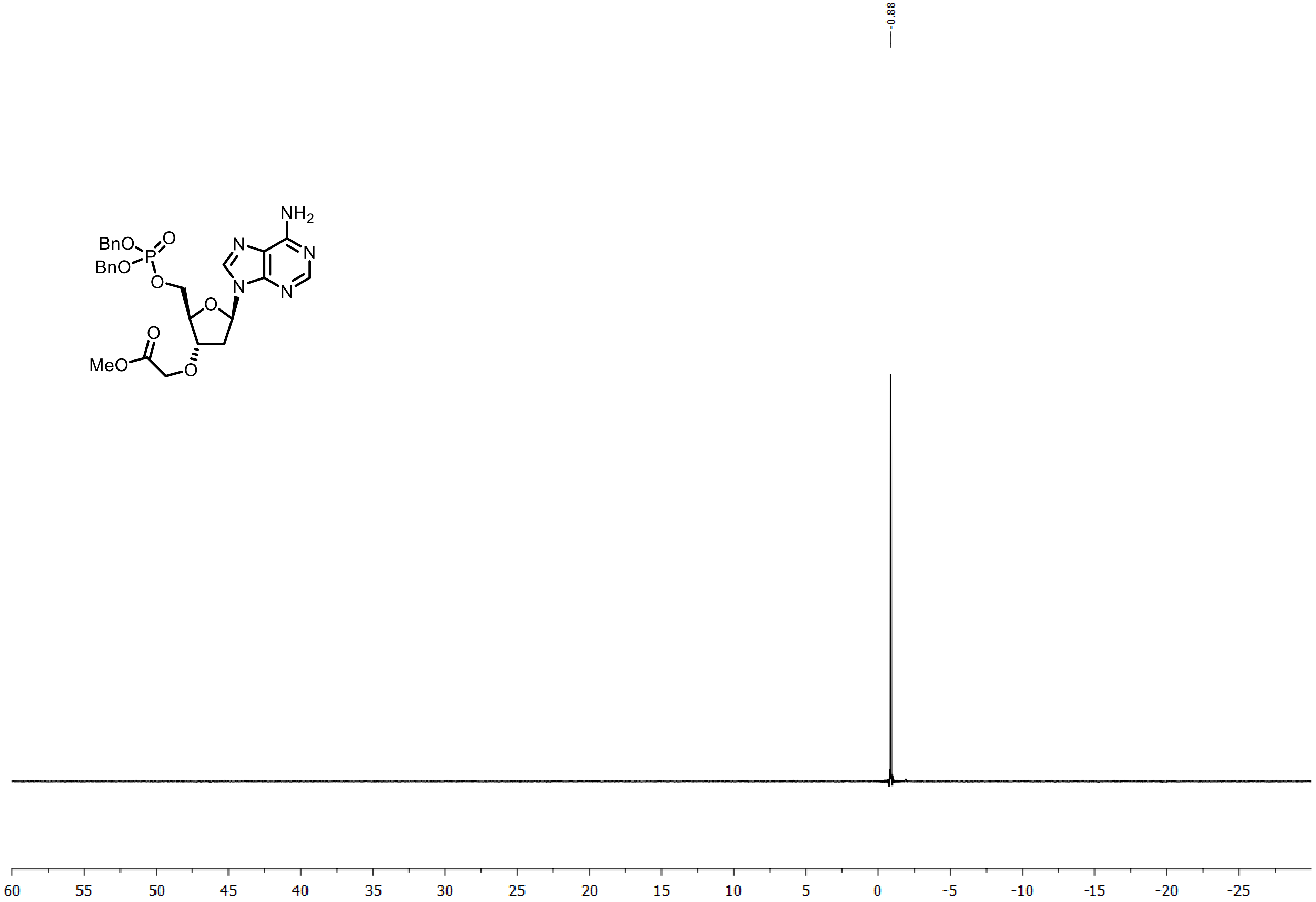
-0.88
NH2
N
N
N
N
BnO
BnO
P
O
O
O
O
MeO
60 55 50 45 40 35 30 25 20 15 10 5 0 -5 -10 -15 -20 -25

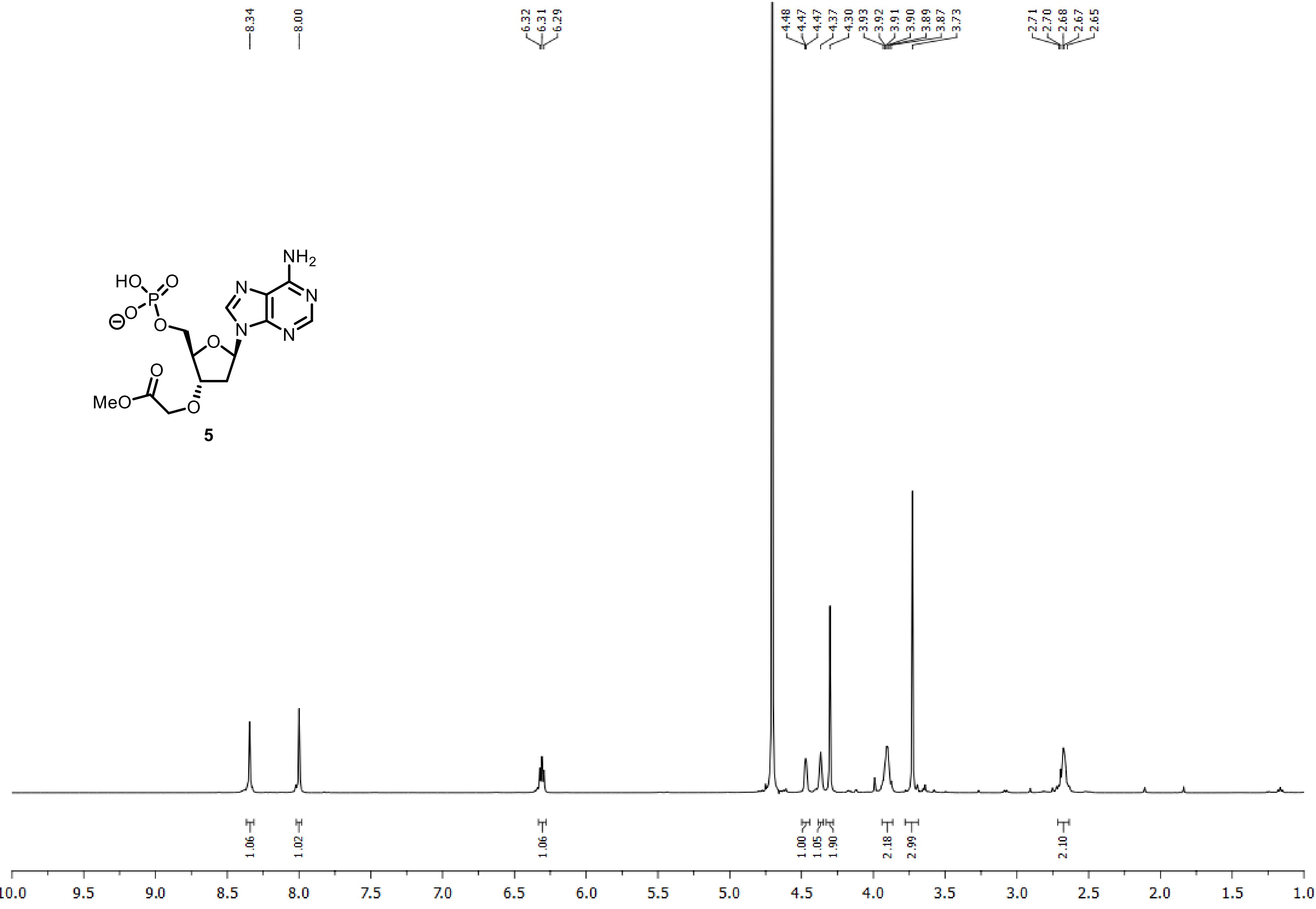
8.34
8.00
6.32
6.31
6.29
4.48
4.47
4.47
4.37
4.30
3.93
3.92
3.91
3.90
3.89
3.87
3.73
2.71
2.70
2.68
2.67
2.65
1.06
1.02
1.06
1.00
1.05
1.90
2.18
2.99
2.10
10.0
9.5
9.0
8.5
8.0
7.5
7.0
6.5
6.0
5.5
5.0
4.5
4.0
3.5
3.0
2.5
2.0
1.5
1.0
NH2
HO
O
P
O
O
N
N
N
N
MeO
O
O
O
5

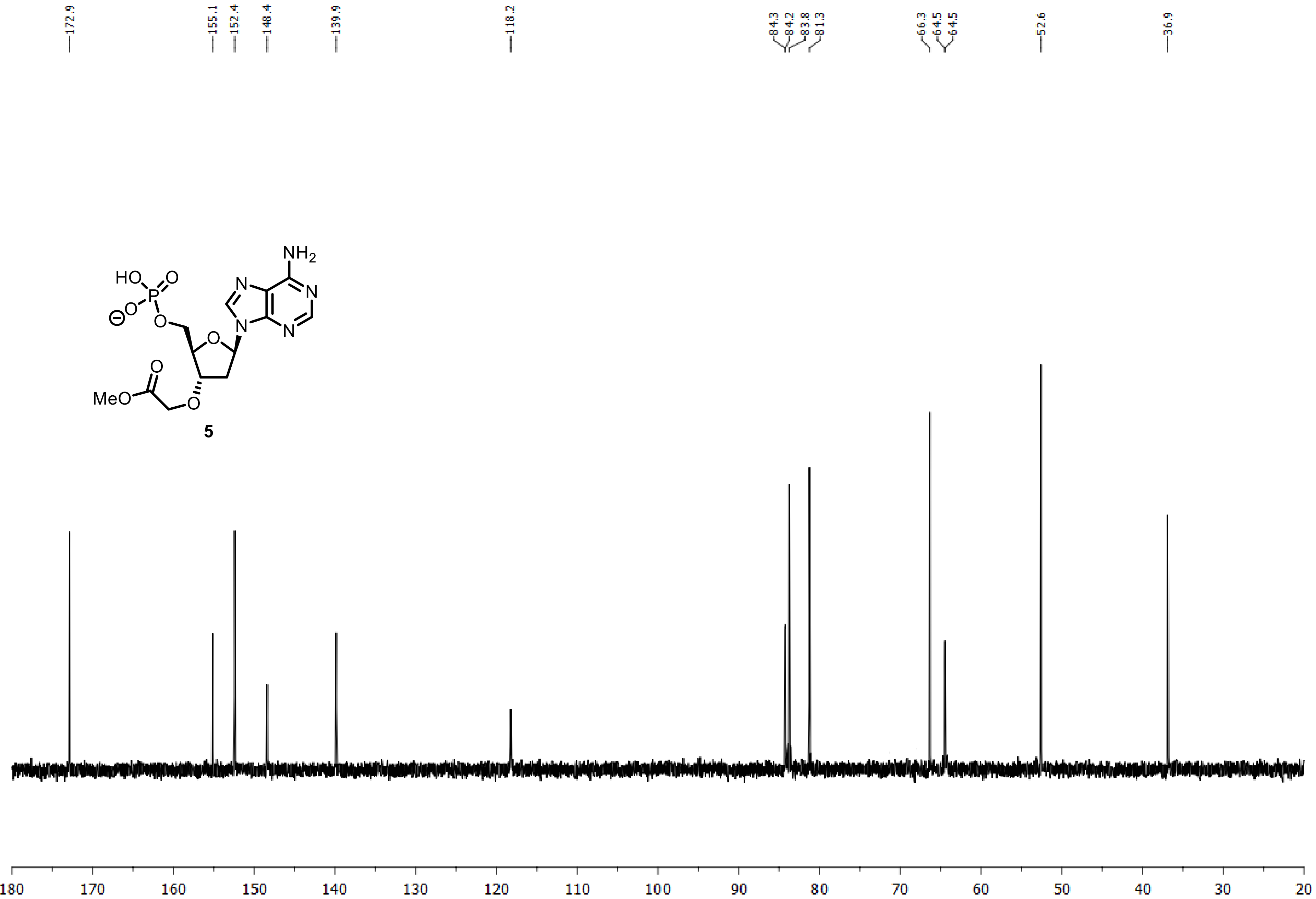
172.9
155.1
152.4
148.4
139.9
118.2
84.3
84.2
83.8
81.3
66.3
64.5
64.5
52.6
36.9
NH2
HO
O
P
O
O
N
N
N
N
O
MeO
5
180
170
160
150
140
130
120
110
100
90
80
70
60
50
40
30
20

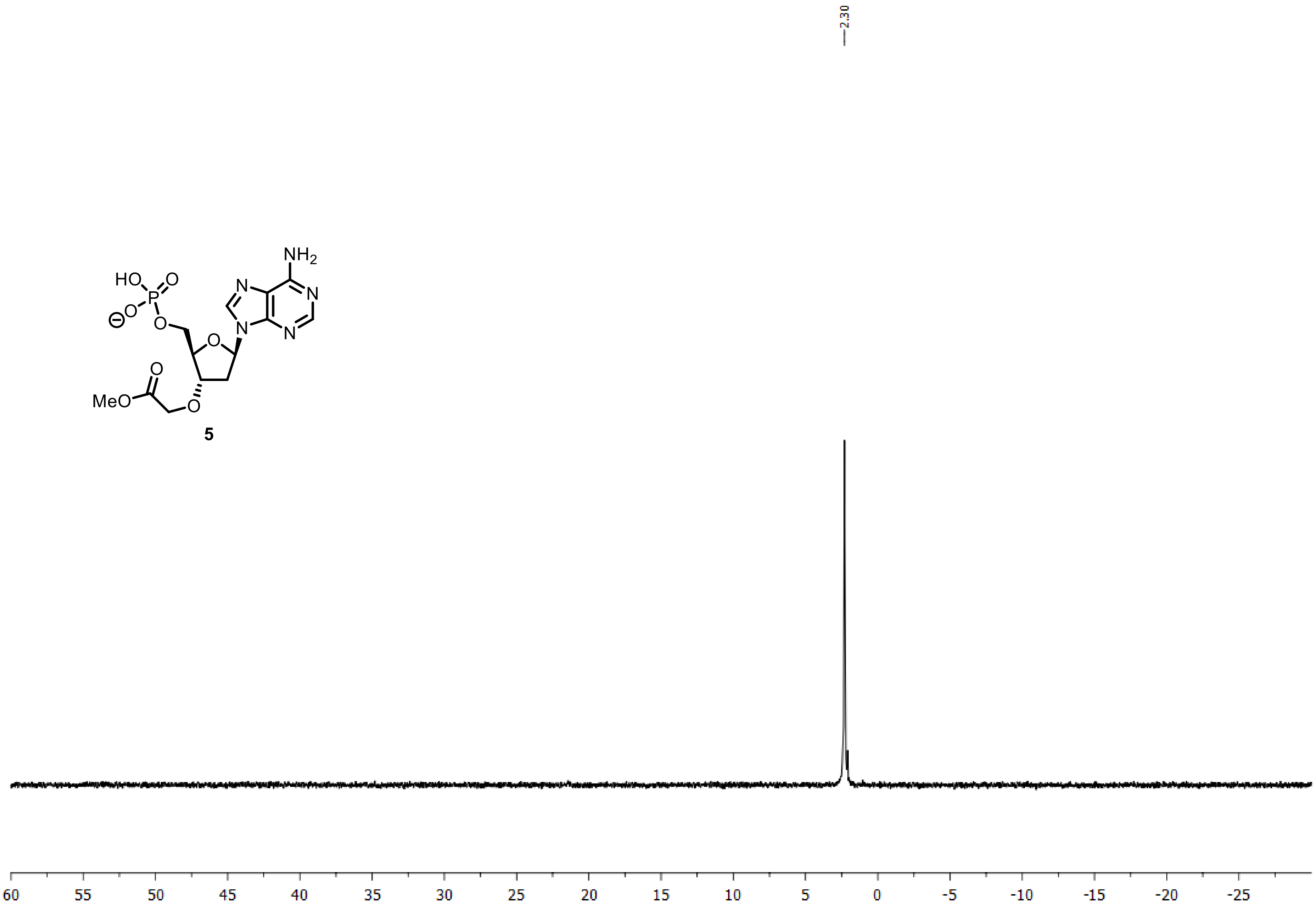

2.30
NH2
N
N
N
N
HO
O
P
O
O
O
O
MeO
O
5
60
55
50
45
40
35
30
25
20
15
10
5
0
-5
-10
-15
-20
-25

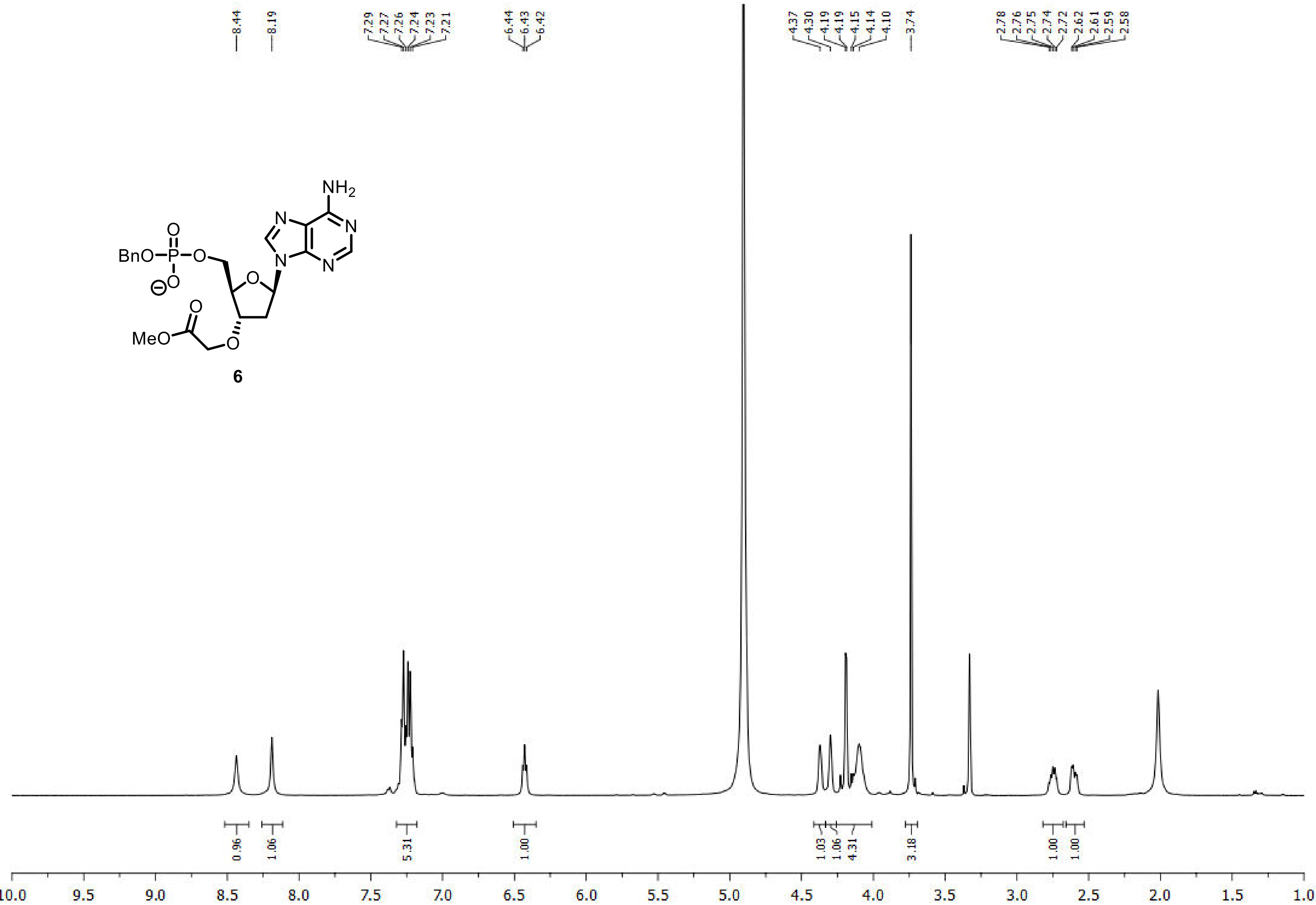
8.44
8.19
7.29 7.27 7.26 7.24 7.23 7.21
6.44 6.43 6.42
4.37 4.30 4.19 4.19 4.15 4.14 4.10
3.74
2.78 2.76 2.75 2.74 2.72 2.62 2.61 2.59 2.58
NH2
N
BnO
P
O
MeO
6
0.96
1.06
5.31
1.00
1.03
1.06
4.31
3.18
1.00
1.00
10.0 9.5 9.0 8.5 8.0 7.5 7.0 6.5 6.0 5.5 5.0 4.5 4.0 3.5 3.0 2.5 2.0 1.5 1.0

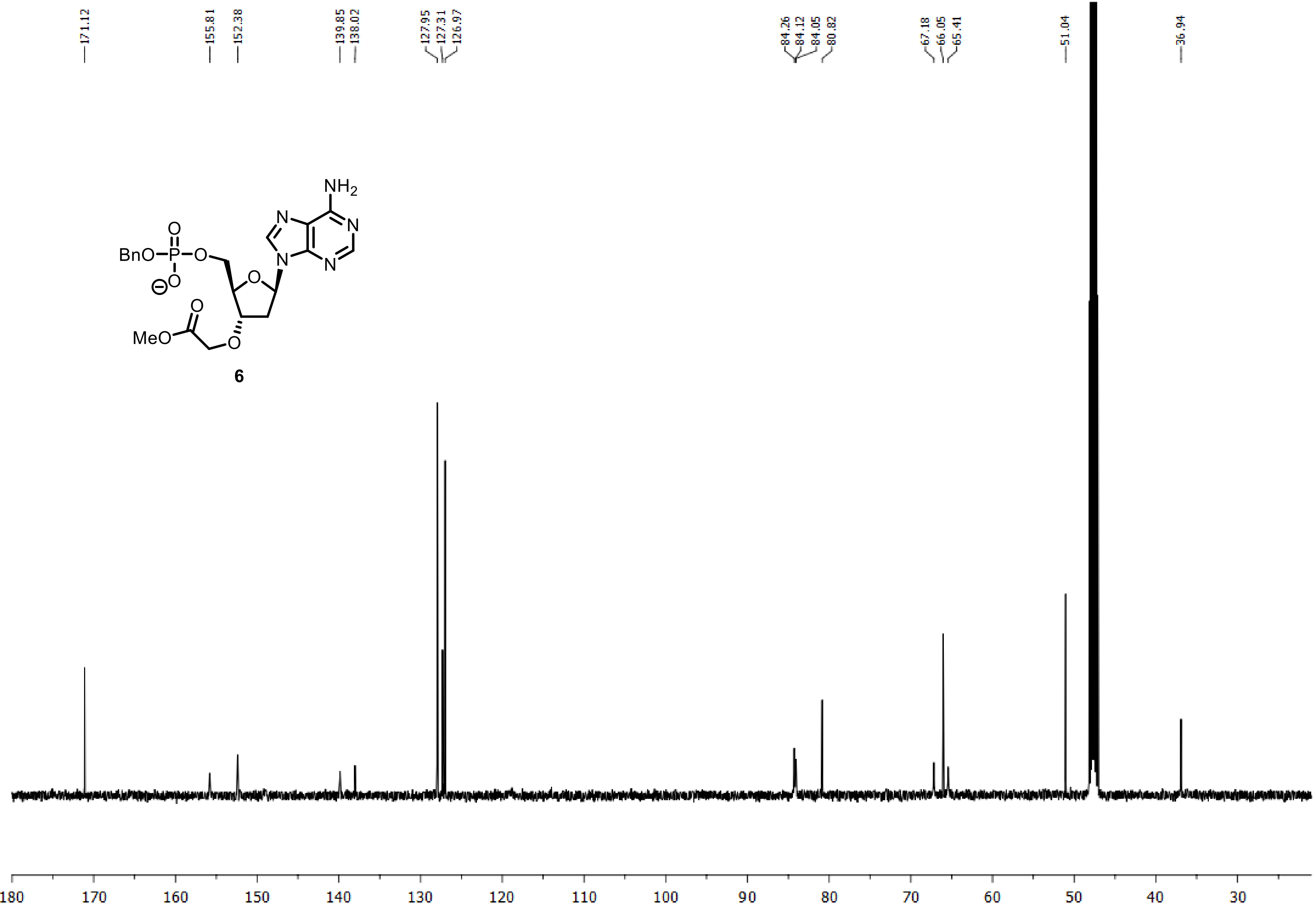
171.12
155.81
152.38
139.85
138.02
127.95
127.31
126.97
84.26
84.12
84.05
80.82
67.18
66.05
65.41
51.04
36.94
NH2
N
BnO
P
O
MeO
6
180
170
160
150
140
130
120
110
100
90
80
70
60
50
40
30

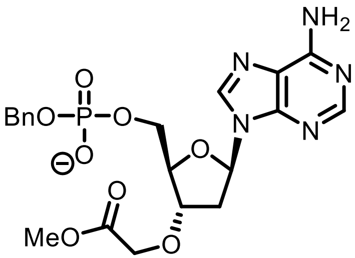

6

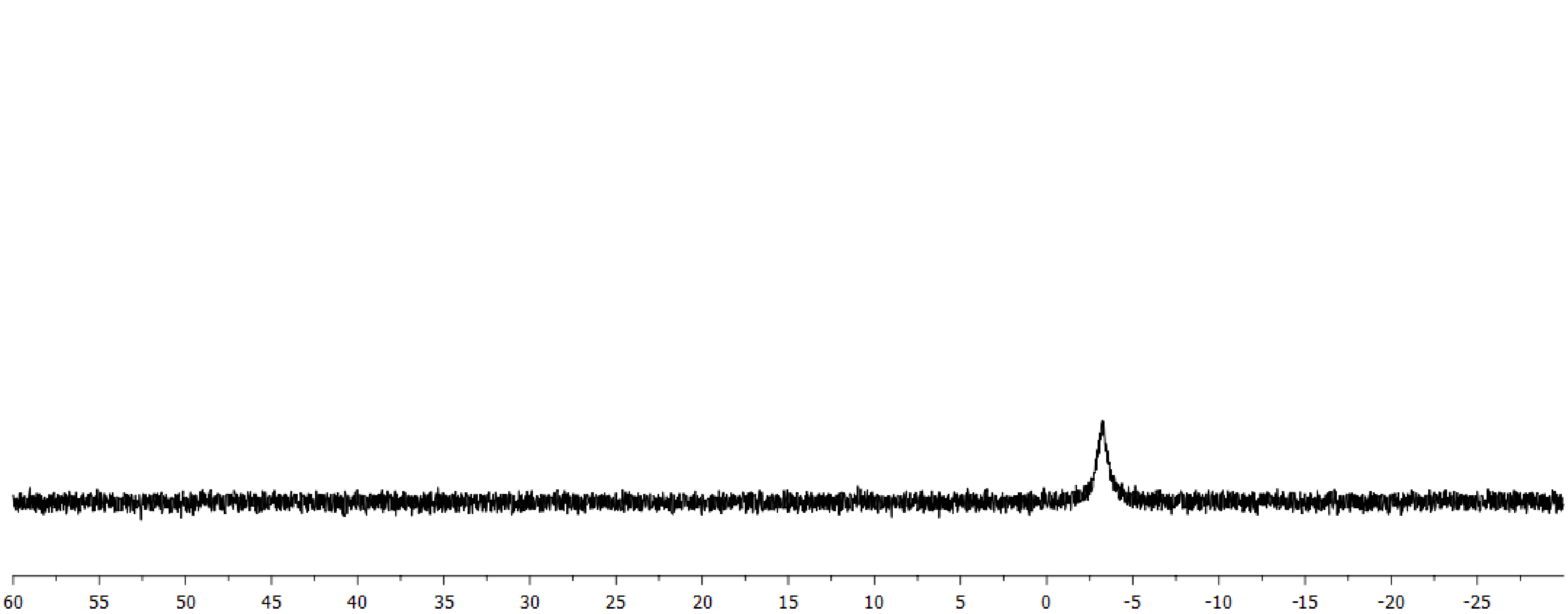

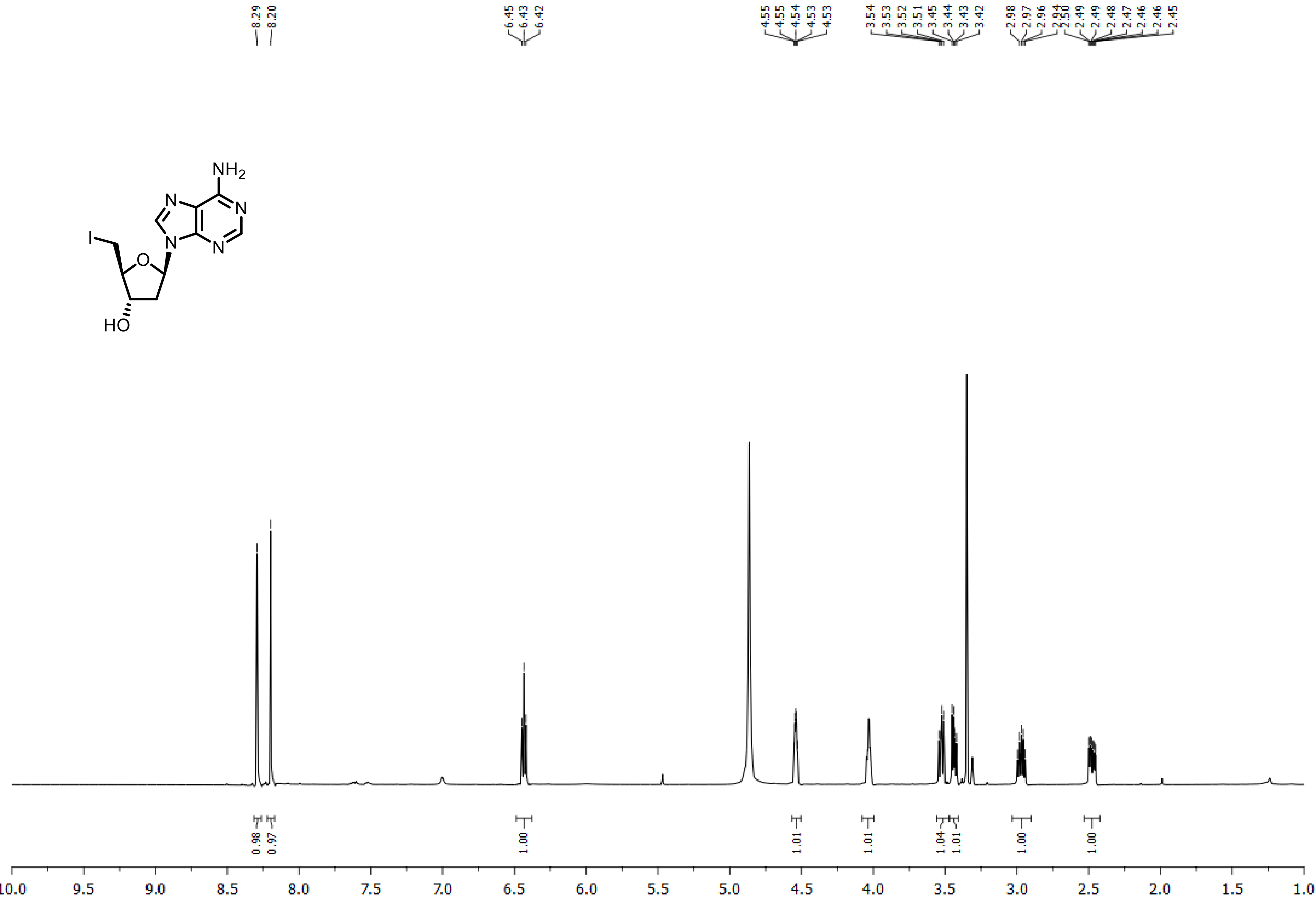
8.29
8.20
6.45
6.43
6.42
4.55
4.55
4.54
4.53
4.53
3.54
3.53
3.52
3.51
3.45
3.44
3.43
3.42
2.98
2.97
2.96
2.94
2.50
2.49
2.49
2.48
2.47
2.46
2.46
2.45
NH2
N
N
N
N
I
O
HO
0.98
0.97
1.00
1.01
1.01
1.04
1.01
1.00
1.00
10.0
9.5
9.0
8.5
8.0
7.5
7.0
6.5
6.0
5.5
5.0
4.5
4.0
3.5
3.0
2.5
2.0
1.5
1.0

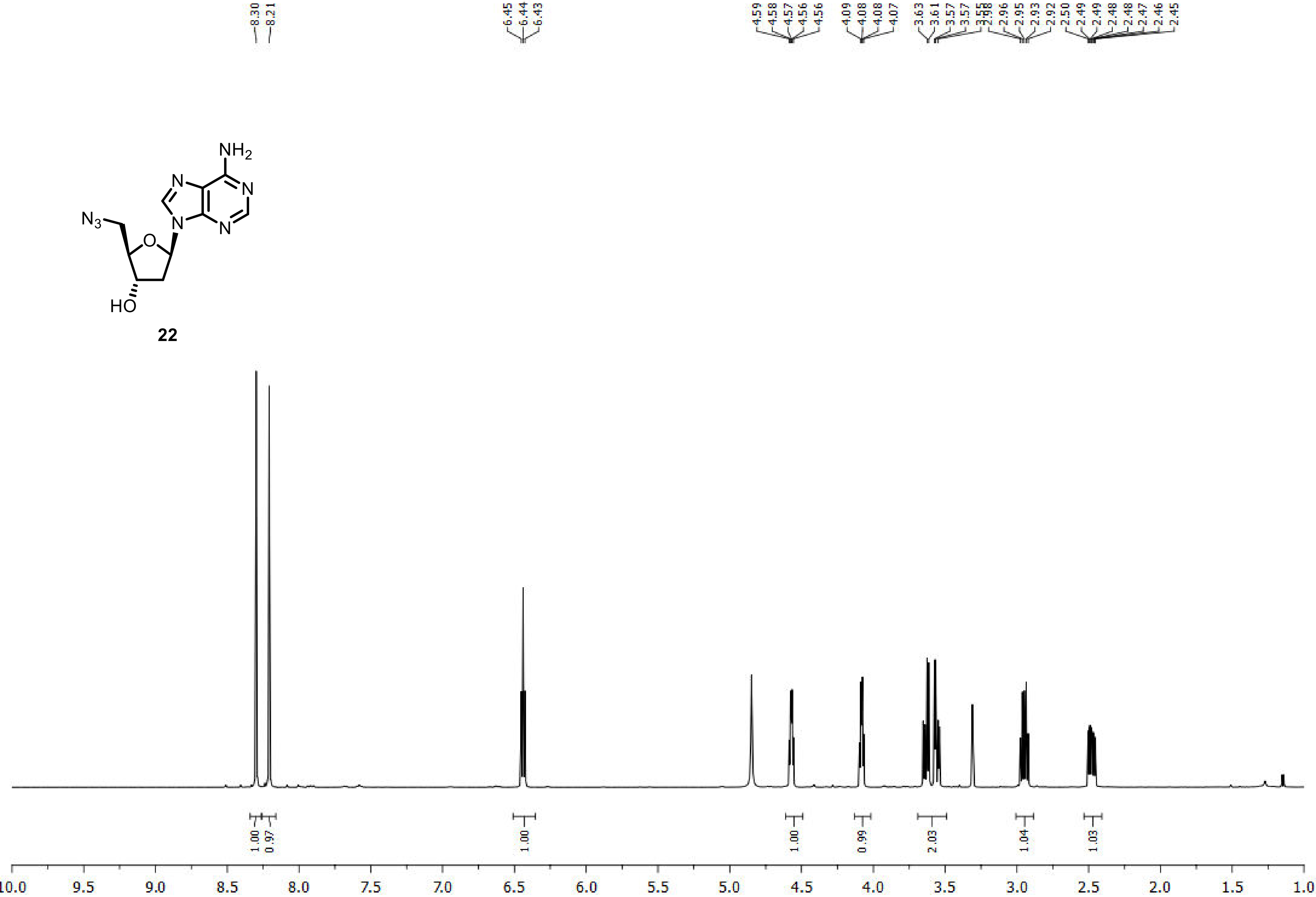

8.30
8.21
6.45
6.44
6.43
4.59
4.58
4.57
4.56
4.56
4.09
4.08
4.08
4.07
3.63
3.61
3.57
3.57
3.55
2.98
2.96
2.95
2.93
2.92
2.50
2.49
2.49
2.48
2.48
2.47
2.46
2.45
NH2
N
N
N
N
N3
O
HO
22
1.00
0.97
1.00
1.00
0.99
2.03
1.04
1.03
10.0
9.5
9.0
8.5
8.0
7.5
7.0
6.5
6.0
5.5
5.0
4.5
4.0
3.5
3.0
2.5
2.0
1.5
1.0

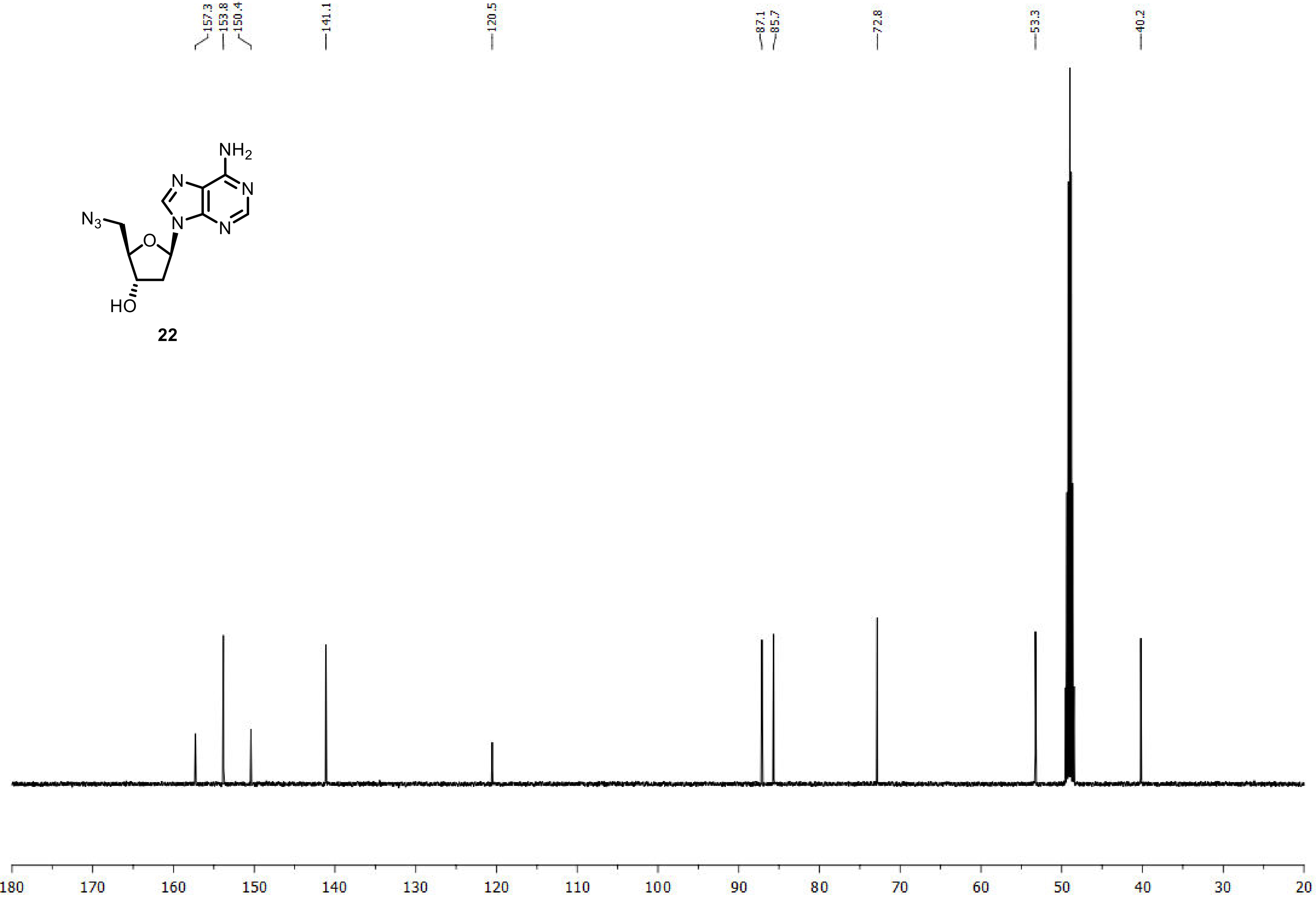
157.3
153.8
150.4
141.1
120.5
87.1
85.7
72.8
53.3
40.2
NH2
N
N
N
N
N3
O
HO
22
180
170
160
150
140
130
120
110
100
90
80
70
60
50
40
30
20

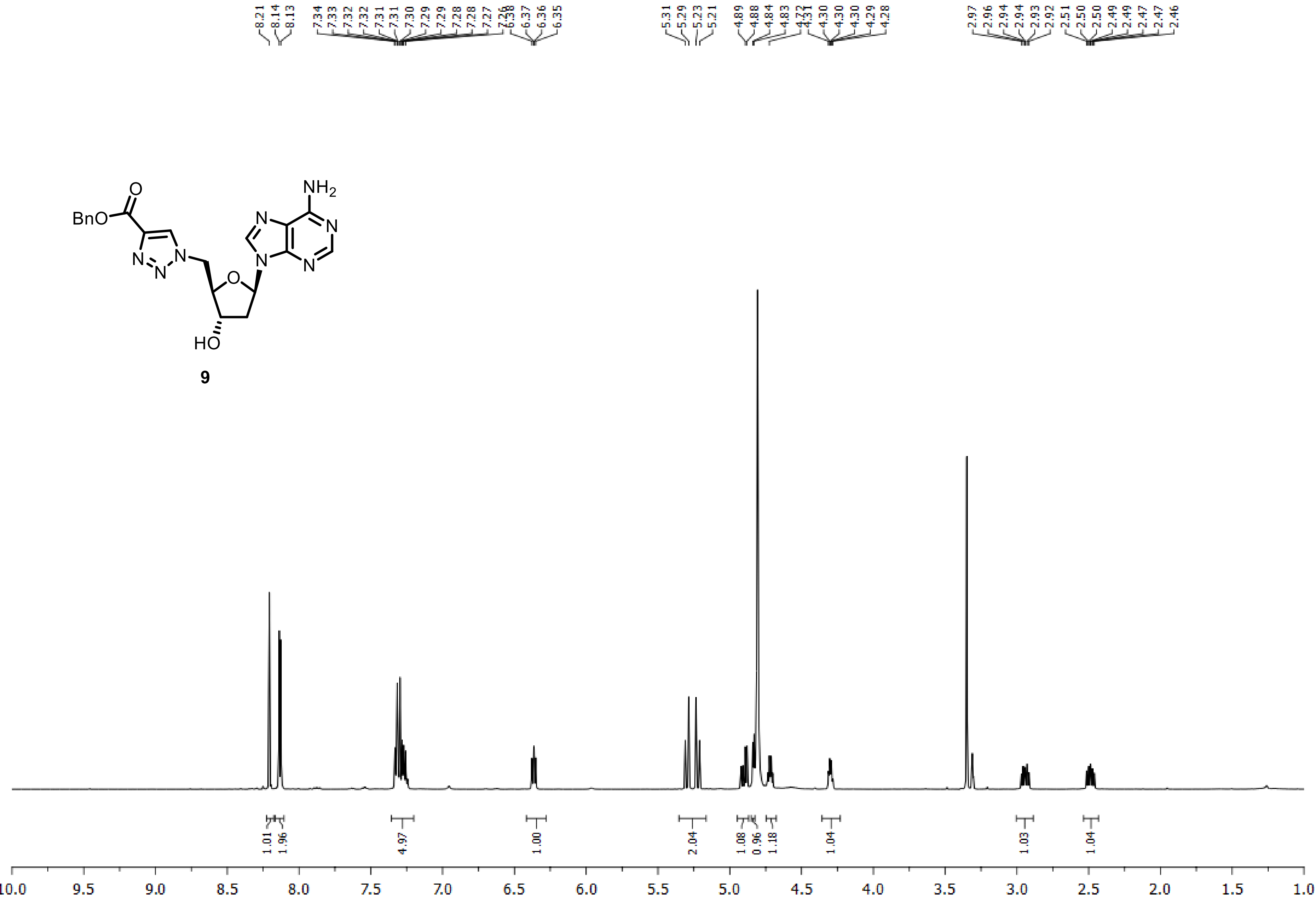

8.21 8.14 8.13 7.34 7.33 7.32 7.32 7.31 7.31 7.30 7.29 7.29 7.28 7.28 7.27 7.26 6.38 6.37 6.36 6.35
5.31 5.29 5.23 5.21 4.89 4.88 4.84 4.83 4.72 4.31 4.30 4.30 4.30 4.29 4.28
2.97 2.96 2.94 2.94 2.93 2.92 2.51 2.50 2.50 2.49 2.49 2.47 2.47 2.46
BnO
O
N
NH2
HO
1.01 1.96 4.97 1.00 2.04 1.08 0.96 1.18 1.04 1.03 1.04
10.0 9.5 9.0 8.5 8.0 7.5 7.0 6.5 6.0 5.5 5.0 4.5 4.0 3.5 3.0 2.5 2.0 1.5 1.0

9

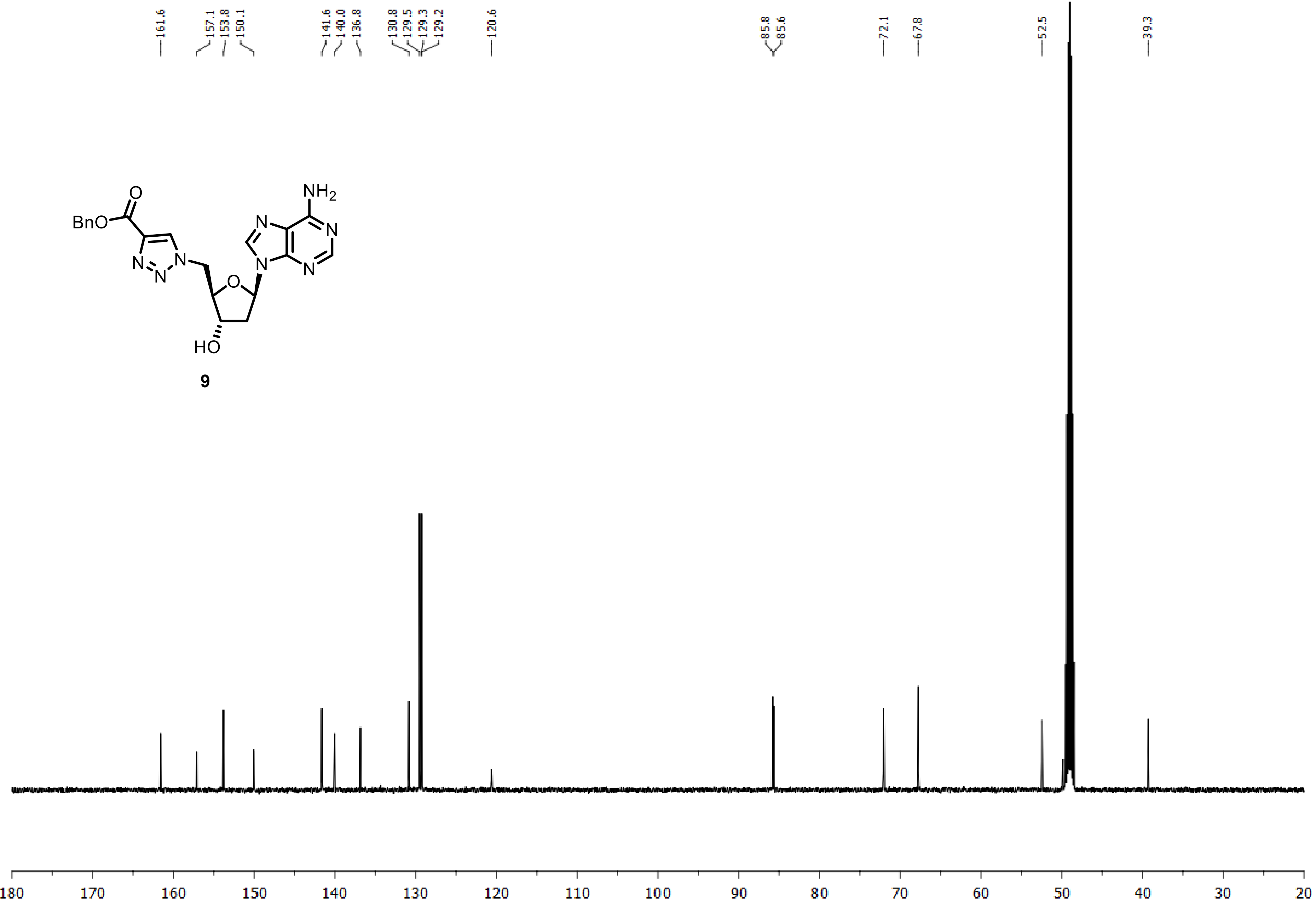
161.6
157.1
153.8
150.1
141.6
140.0
136.8
130.8
129.5
129.3
129.2
120.6
85.8
85.6
72.1
67.8
52.5
39.3
BnO
O
N
NH2
180
170
160
150
140
130
120
110
100
90
80
70
60
50
40
30
20

9

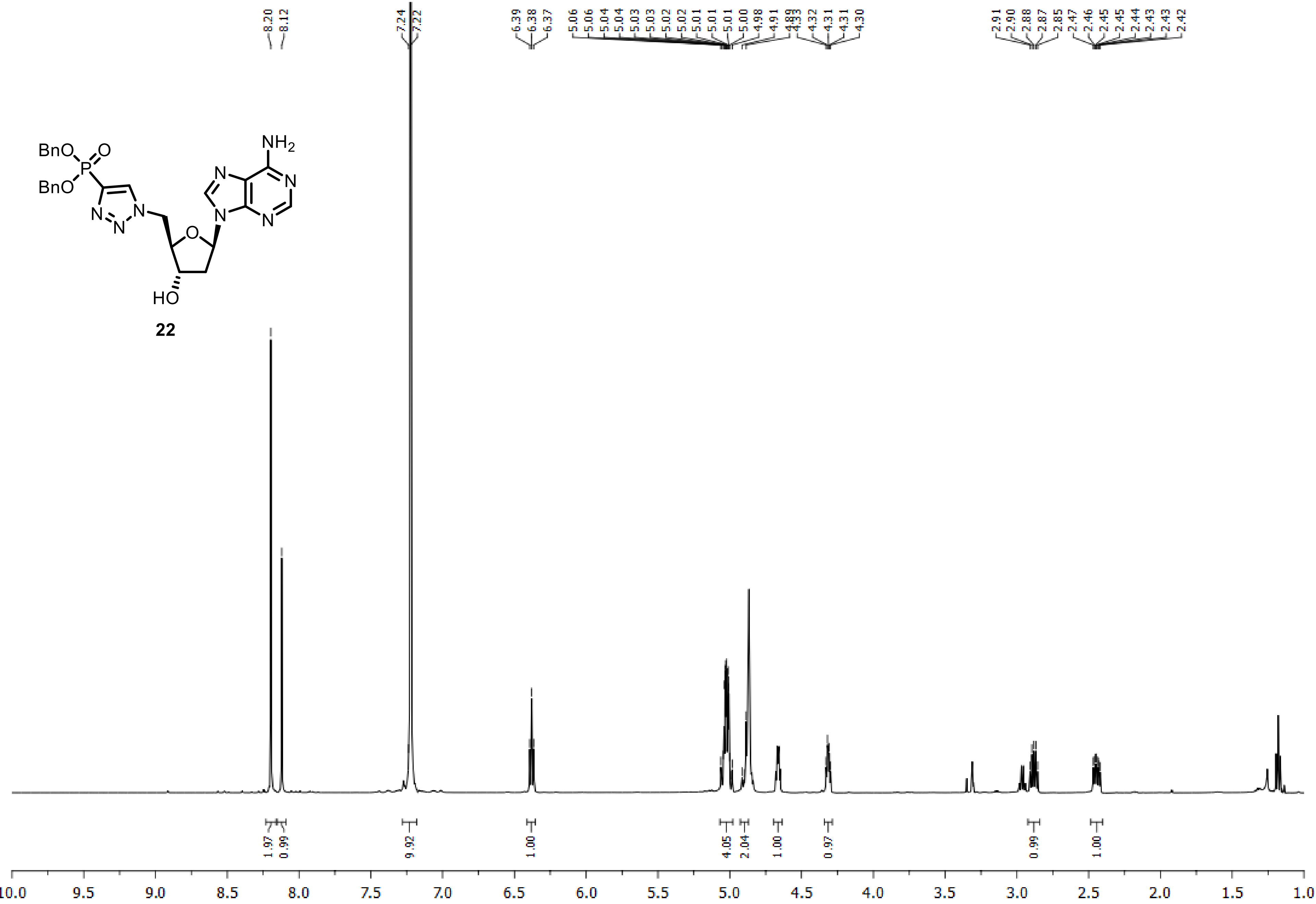
8.20
8.12
7.24
7.22
6.39
6.38
6.37
5.06
5.06
5.04
5.04
5.03
5.03
5.02
5.02
5.01
5.01
5.01
5.00
4.98
4.91
4.89
4.33
4.32
4.31
4.31
4.30
2.91
2.90
2.88
2.87
2.85
2.47
2.46
2.45
2.45
2.44
2.43
2.43
2.42
BnO
O
P
BnO
N
N
N
NH$_2$
N
N
N
N
O
HO
1.97
0.99
9.92
1.00
4.05
2.04
1.00
0.97
0.99
1.00
10.0
9.5
9.0
8.5
8.0
7.5
7.0
6.5
6.0
5.5
5.0
4.5
4.0
3.5
3.0
2.5
2.0
1.5
1.0

22

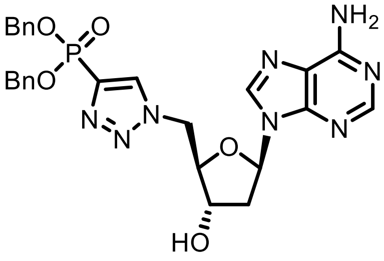

22

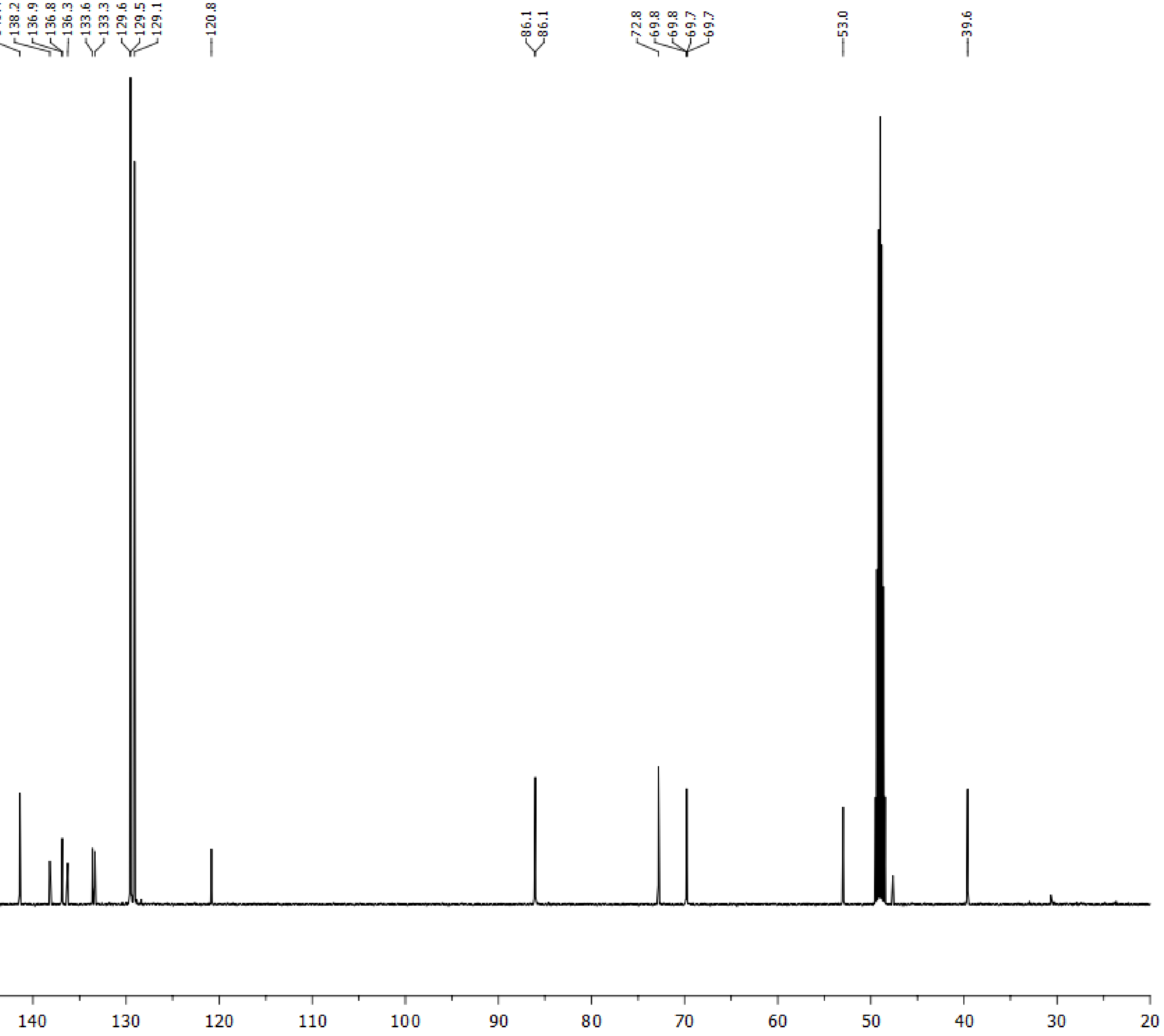
157.3
153.9
150.2
141.4
138.2
136.9
136.8
136.3
133.6
133.3
129.6
129.5
129.1
120.8
86.1
86.1
72.8
69.8
69.8
69.7
69.7
53.0
39.6
180
170
160
150
140
130
120
110
100
90
80
70
60
50
40
30
20

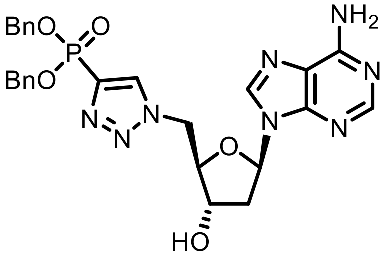
BnO
BnO
P
O
N
N
N
O
N
N
N
N
NH2
HO

22

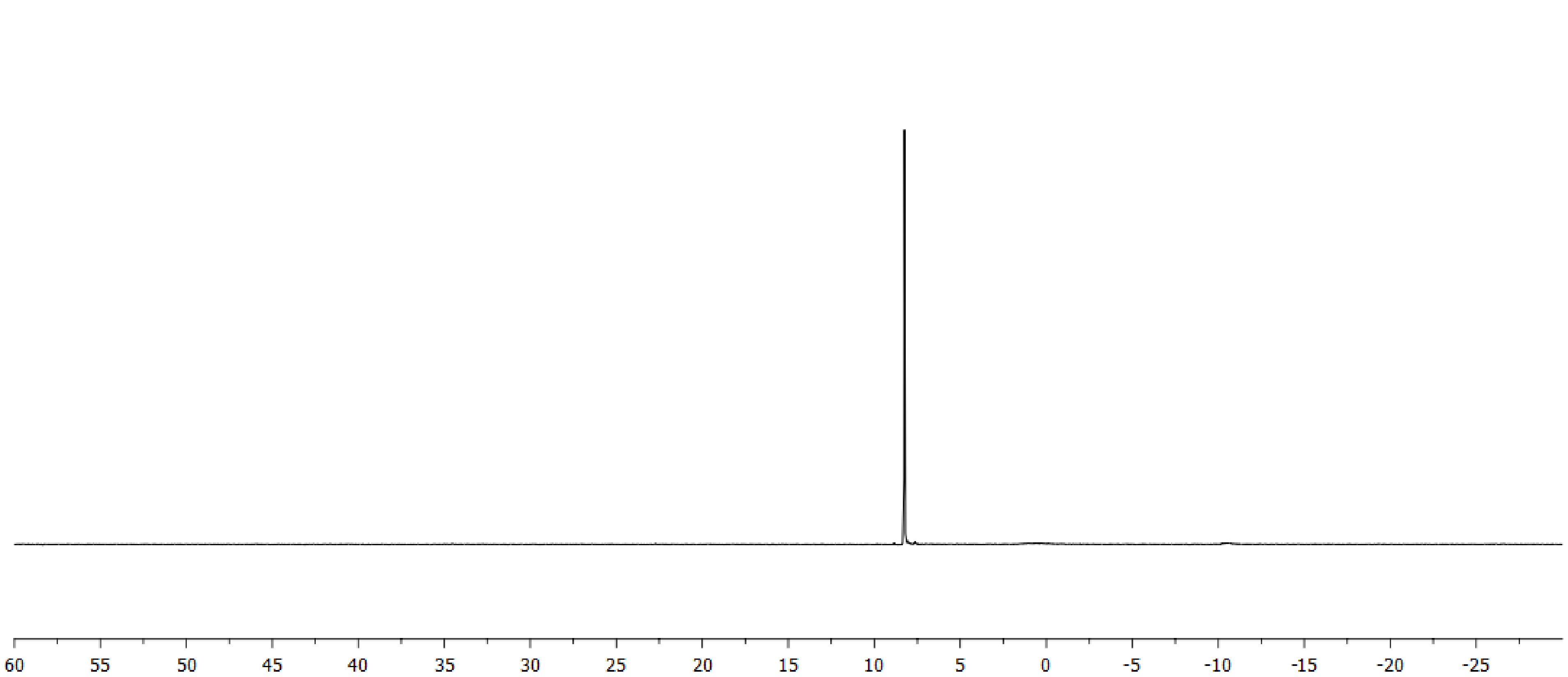
8.25
60
55
50
45
40
35
30
25
20
15
10
5
0
-5
-10
-15
-20
-25

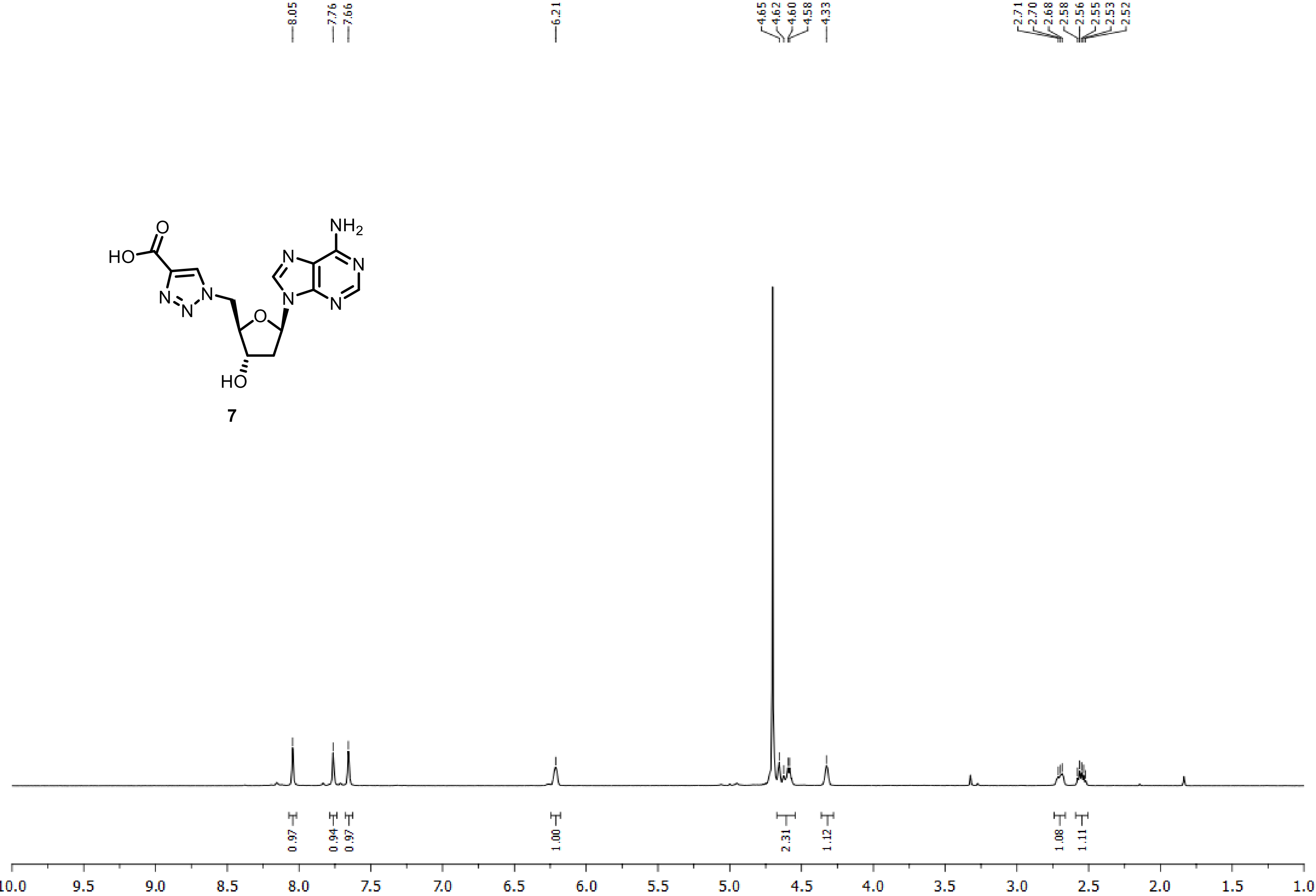
8.05
7.76
7.66
6.21
4.65
4.62
4.60
4.58
4.33
2.71
2.70
2.68
2.58
2.56
2.55
2.53
2.52
NH2
HO
O
N
HO
7
0.97
0.94
0.97
1.00
2.31
1.12
1.08
1.11
10.0 9.5 9.0 8.5 8.0 7.5 7.0 6.5 6.0 5.5 5.0 4.5 4.0 3.5 3.0 2.5 2.0 1.5 1.0

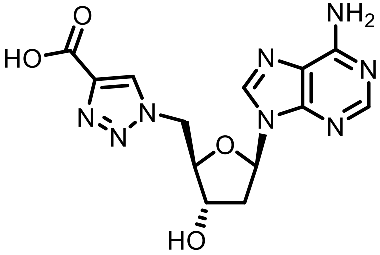

7

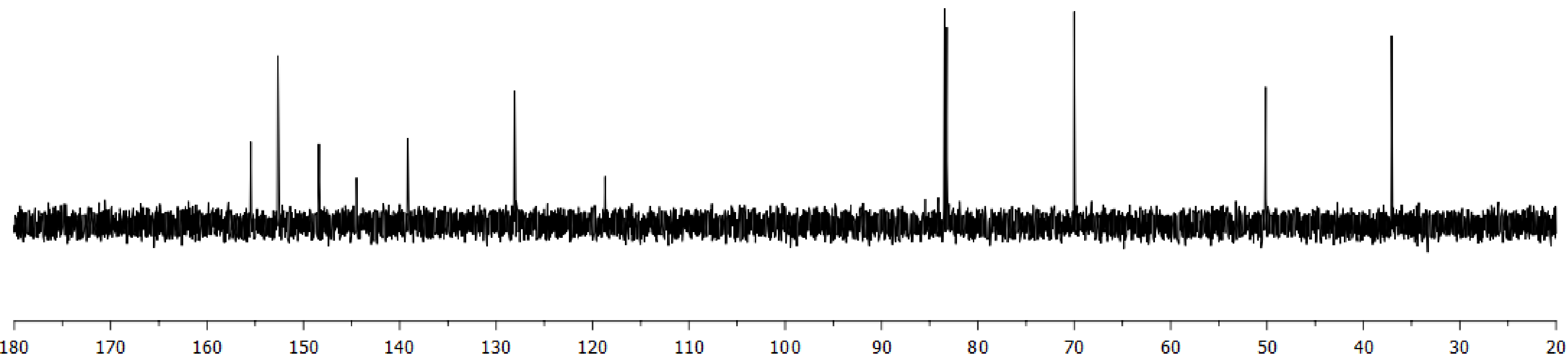
155.5
152.7
148.4
144.5
139.2
128.1
118.7
83.5
83.2
70.0
50.1
37.1
180
170
160
150
140
130
120
110
100
90
80
70
60
50
40
30
20

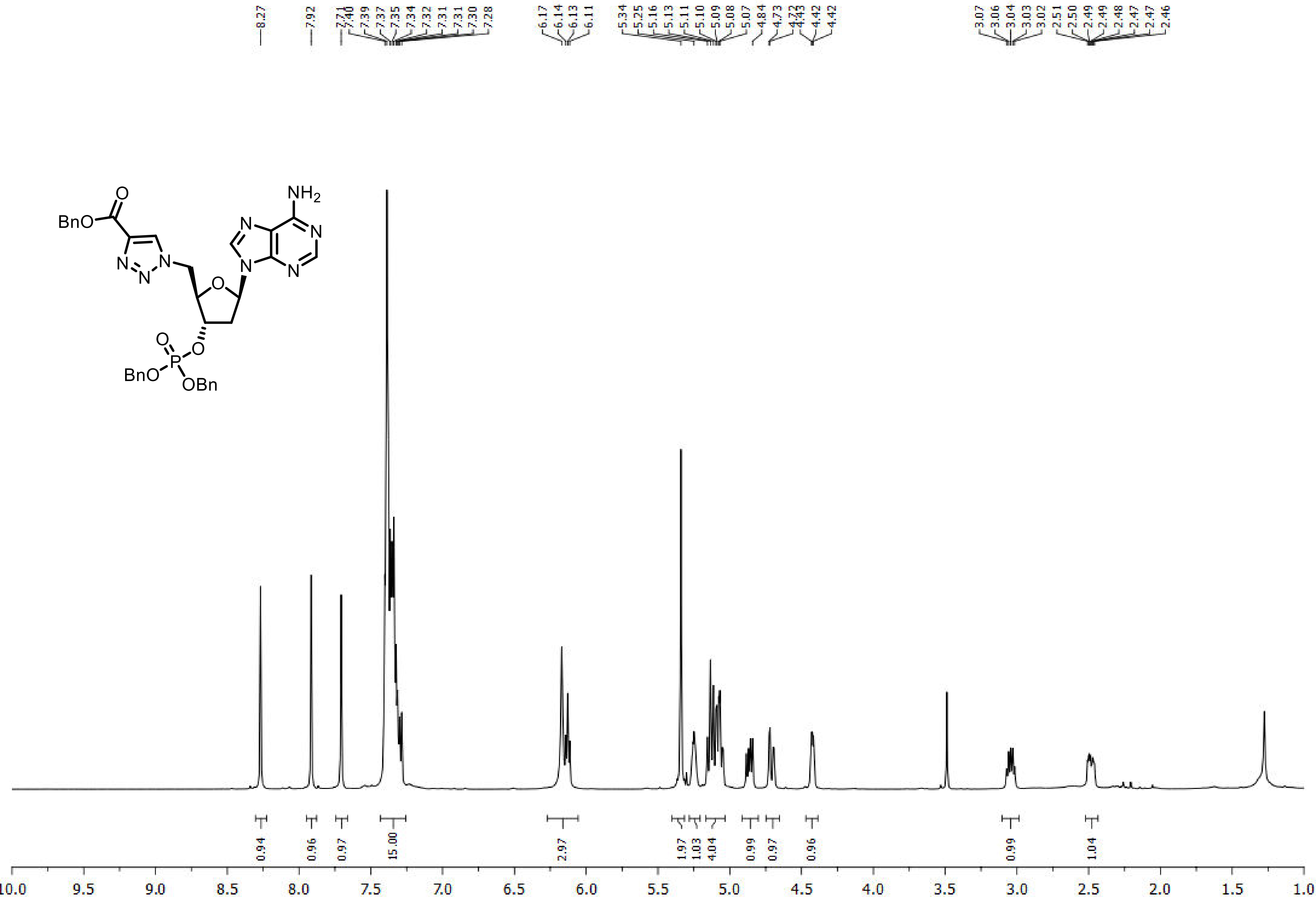

BnO
O
N
N
N
NH2
N
N
N
O
O
P
BnO
OBn
10.0
9.5
9.0
8.5
8.0
7.5
7.0
6.5
6.0
5.5
5.0
4.5
4.0
3.5
3.0
2.5
2.0
1.5
1.0

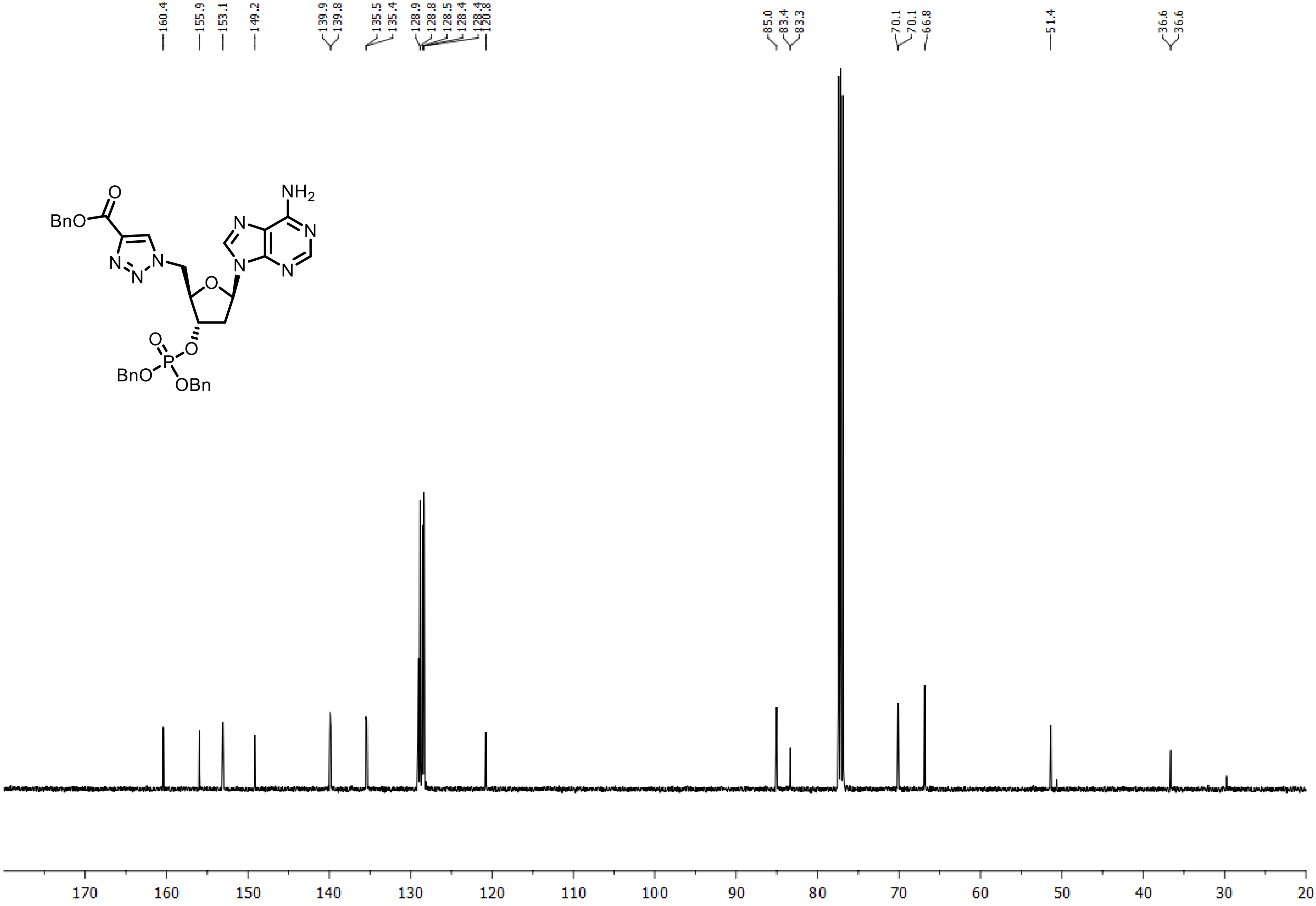
160.4
155.9
153.1
149.2
139.9
139.8
135.5
135.4
128.9
128.8
128.5
128.4
128.4
120.8
85.0
83.4
83.3
70.1
70.1
66.8
51.4
36.6
36.6
BnO
O
N
NH2
OBn
P
170 160 150 140 130 120 110 100 90 80 70 60 50 40 30 20

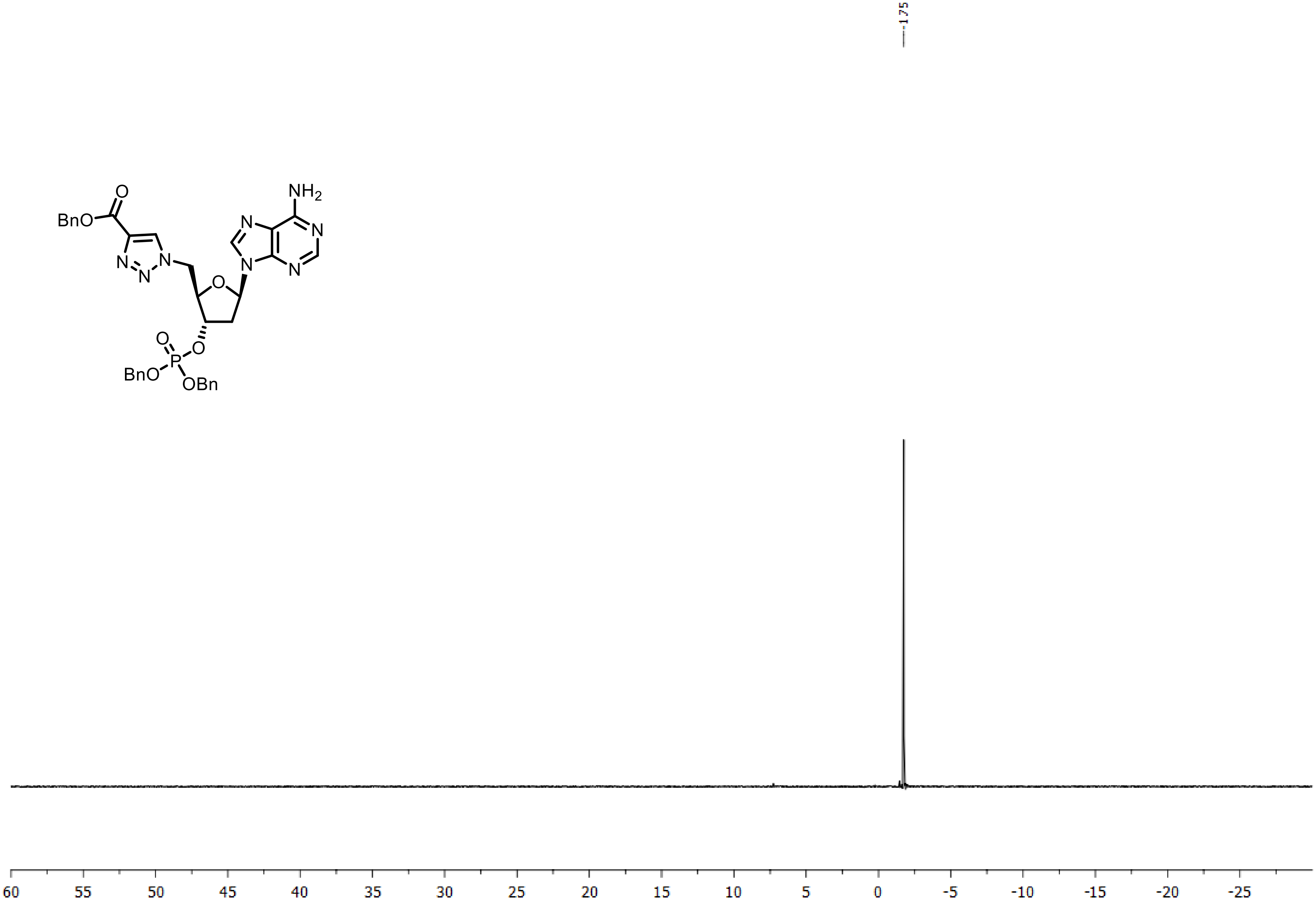
-1.75
BnO
O
NH2
N
BnO
OBn
60
55
50
45
40
35
30
25
20
15
10
5
0
-5
-10
-15
-20
-25

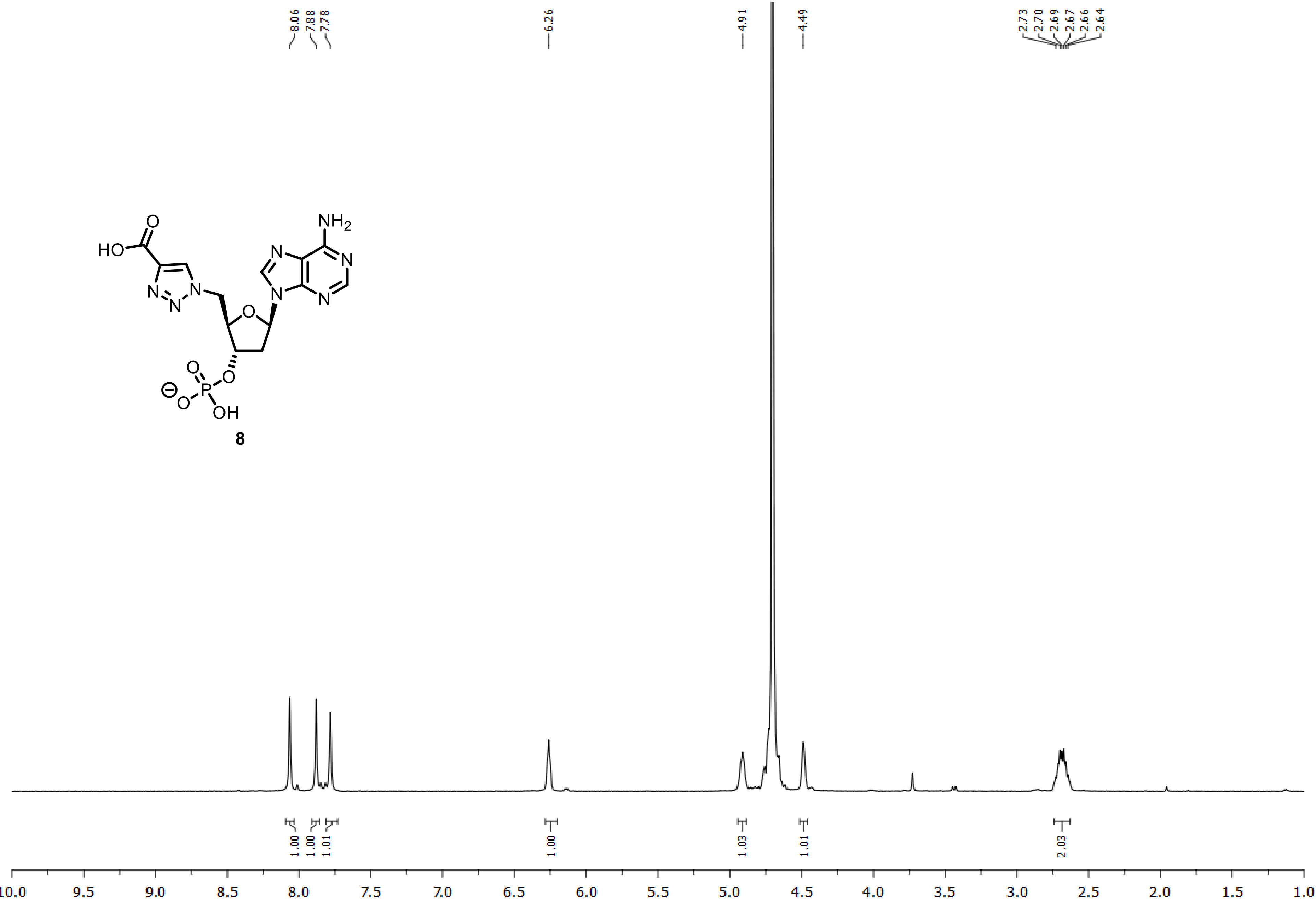
8.06
7.88
7.78
6.26
4.91
4.49
2.73
2.70
2.69
2.67
2.66
2.64
HO
O
NH2
N
N
N
N
N
N
N
O
O
P
O
O
OH
8
1.00
1.00
1.01
1.00
1.03
1.01
2.03
10.0
9.5
9.0
8.5
8.0
7.5
7.0
6.5
6.0
5.5
5.0
4.5
4.0
3.5
3.0
2.5
2.0
1.5
1.0

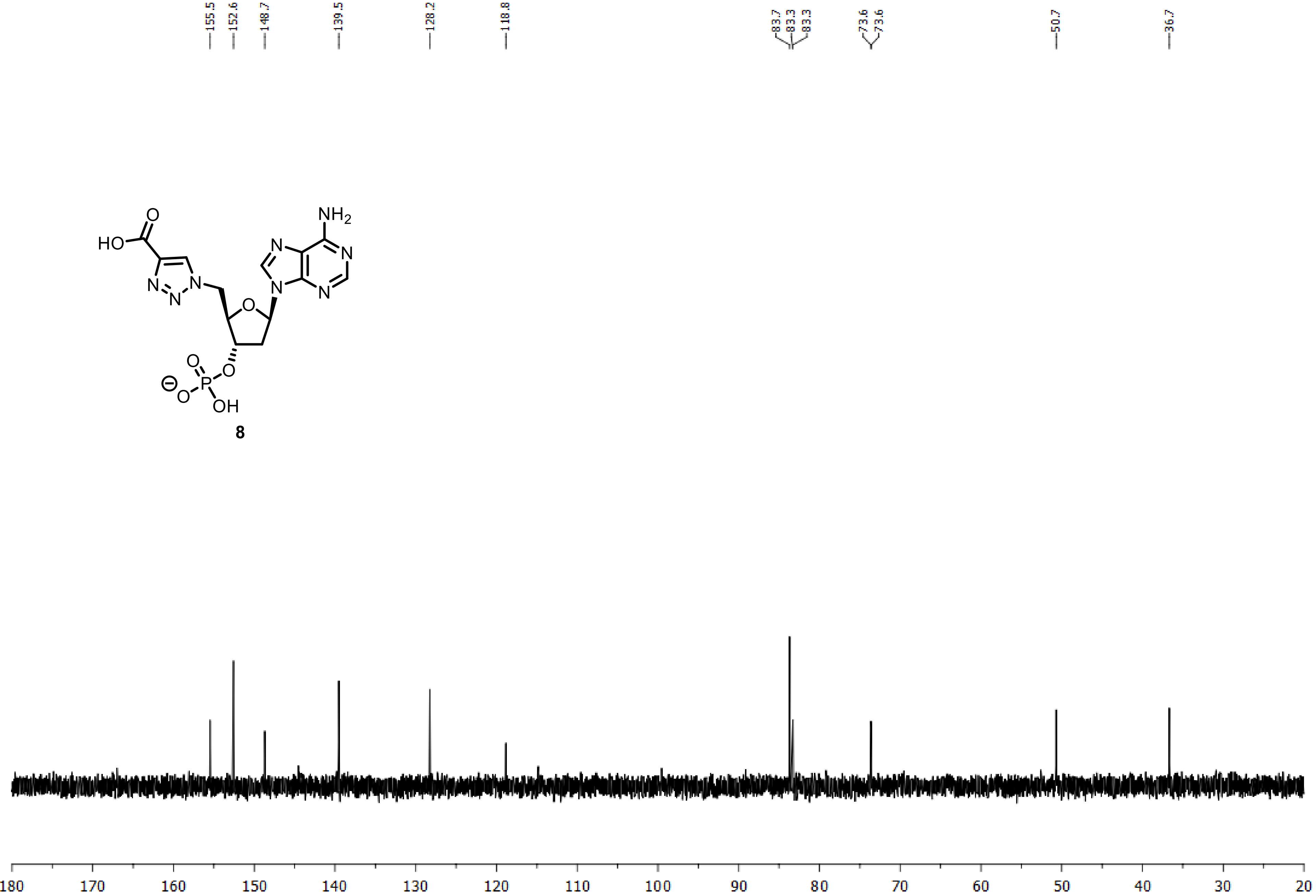
155.5
152.6
148.7
139.5
128.2
118.8
83.7
83.3
83.3
73.6
73.6
50.7
36.7
HO
O
N
N
N
NH2
N
N
N
N
O
O
O
P
O
OH
8
180
170
160
150
140
130
120
110
100
90
80
70
60
50
40
30
20

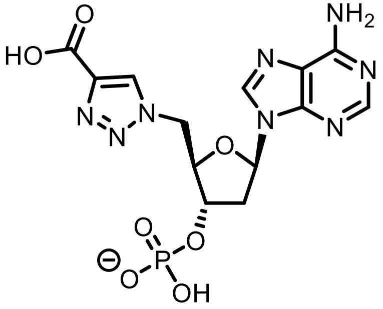

8

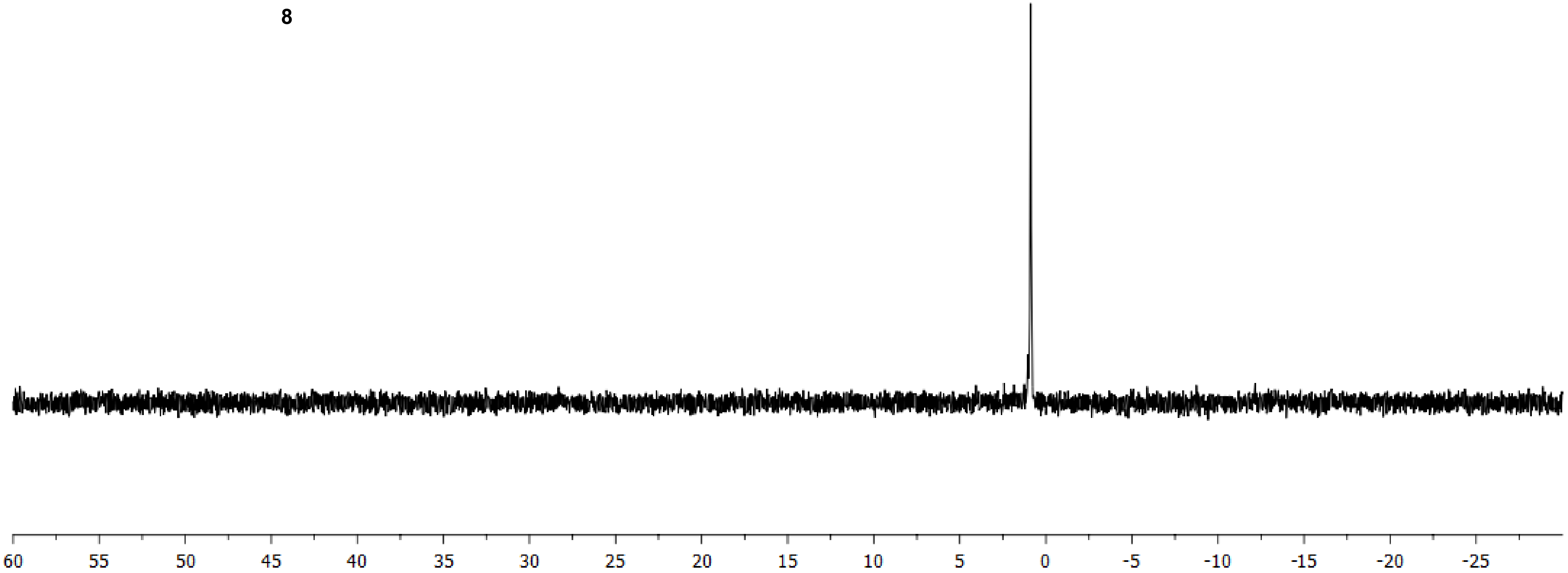

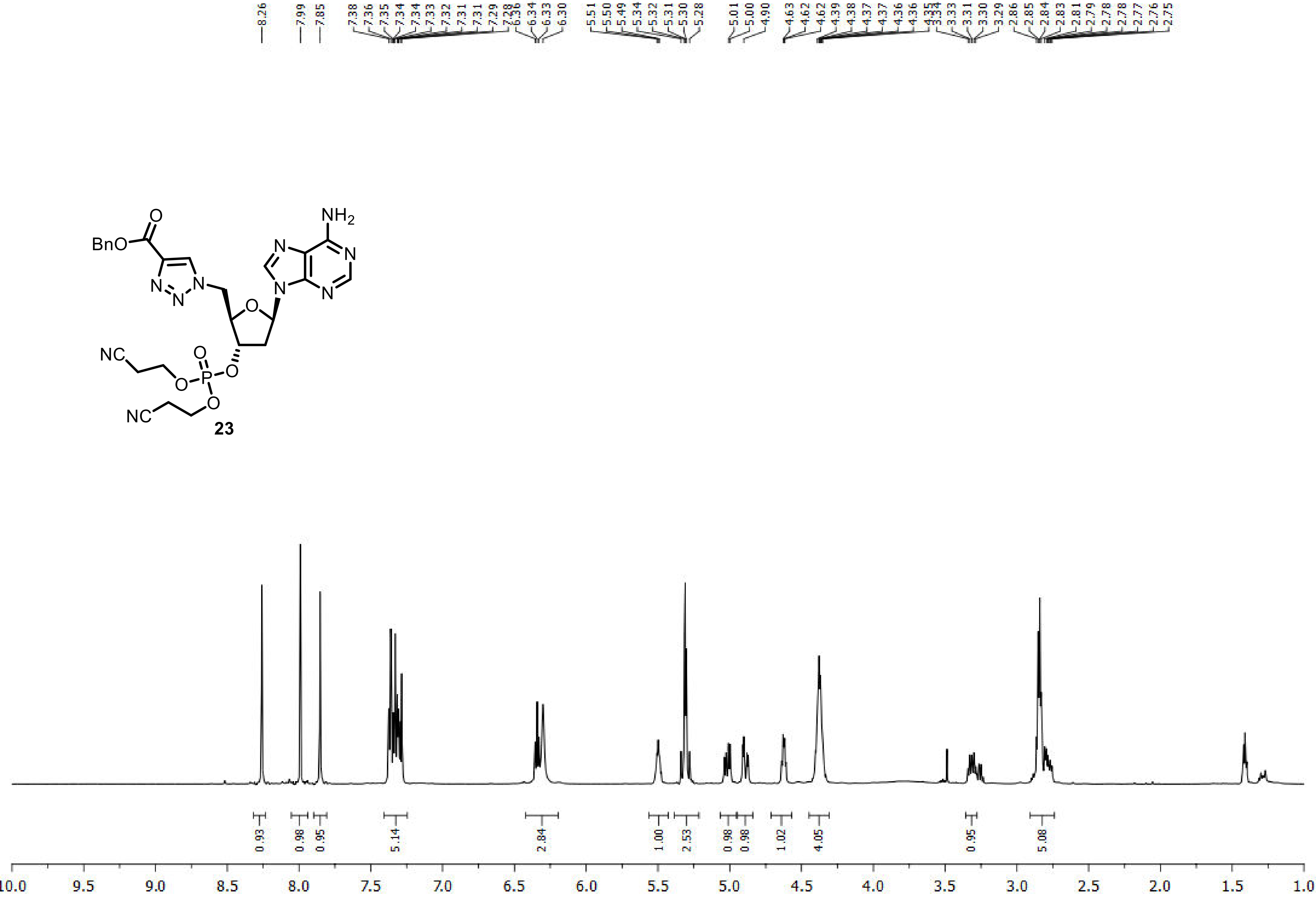

23

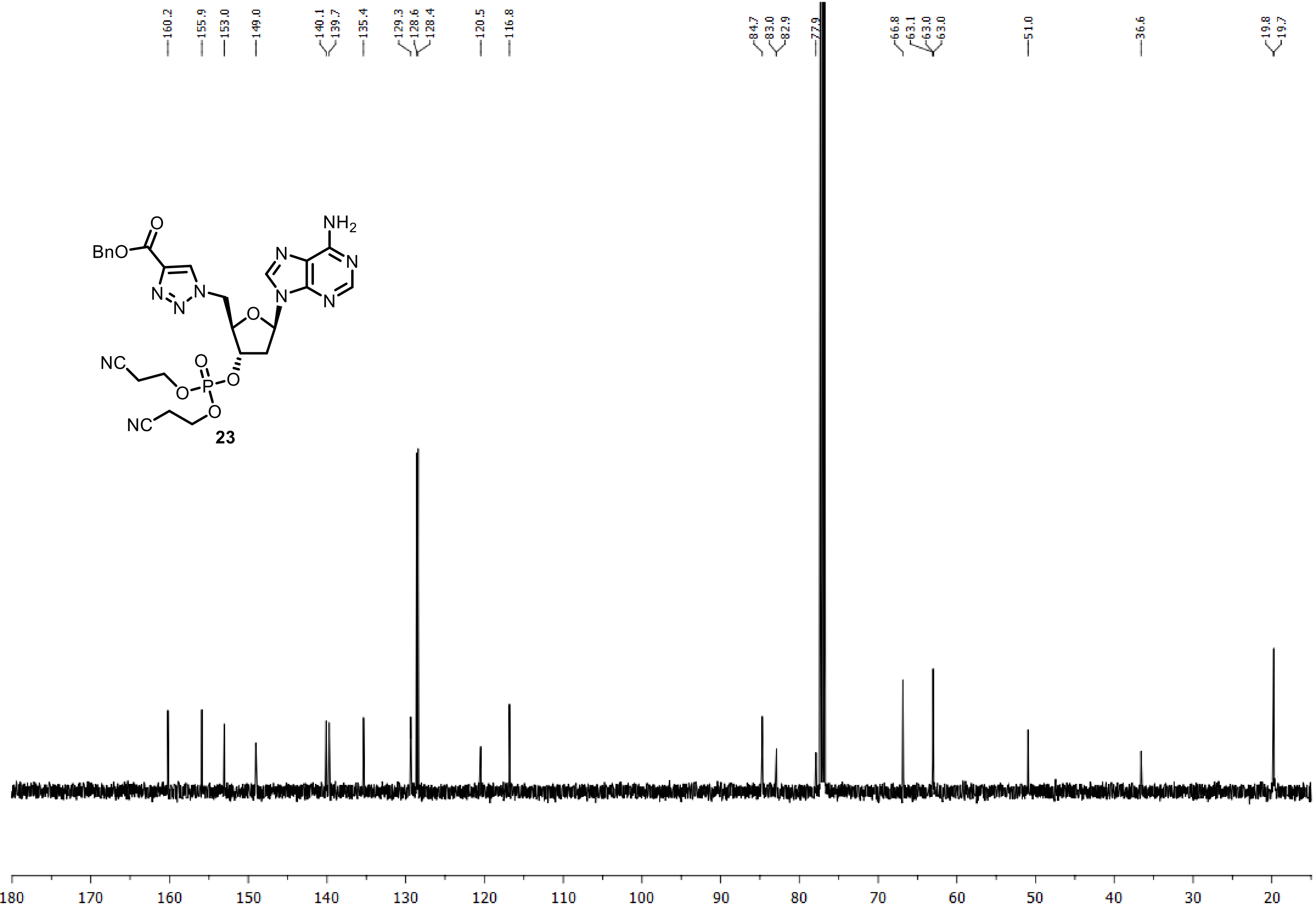
160.2
155.9
153.0
149.0
140.1
139.7
135.4
129.3
128.6
128.4
120.5
116.8
84.7
83.0
82.9
77.9
66.8
63.1
63.0
63.0
51.0
36.6
19.8
19.7
BnO
O
N
N
N
NH2
N
N
N
N
O
NC
O
P
O
O
O
NC
23
180
170
160
150
140
130
120
110
100
90
80
70
60
50
40
30
20

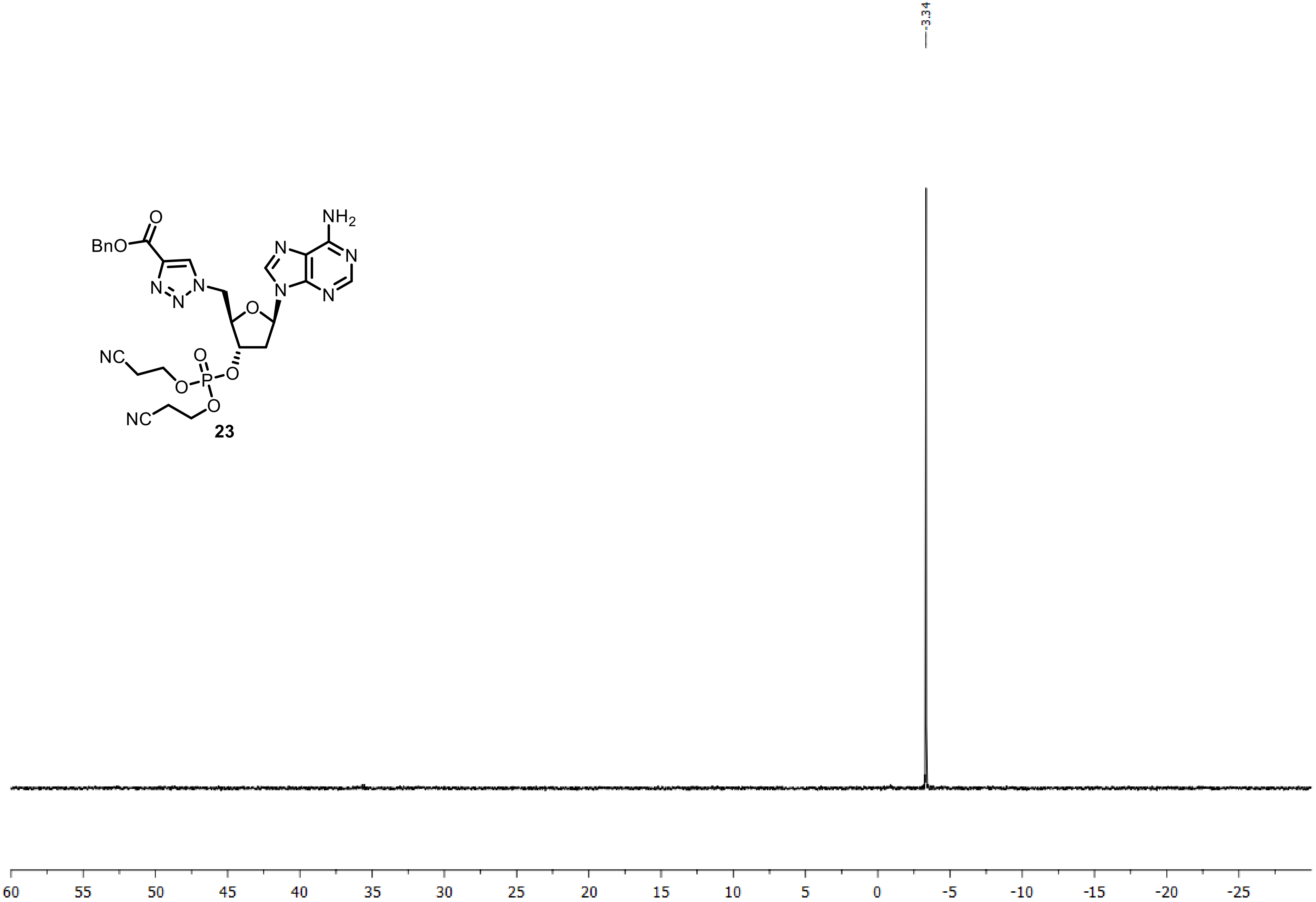
BnO
O
N
N
N
N
N
N
N
NH2
O
NC
O
O
P
O
O
NC
23
-3.34
60
55
50
45
40
35
30
25
20
15
10
5
0
-5
-10
-15
-20
-25

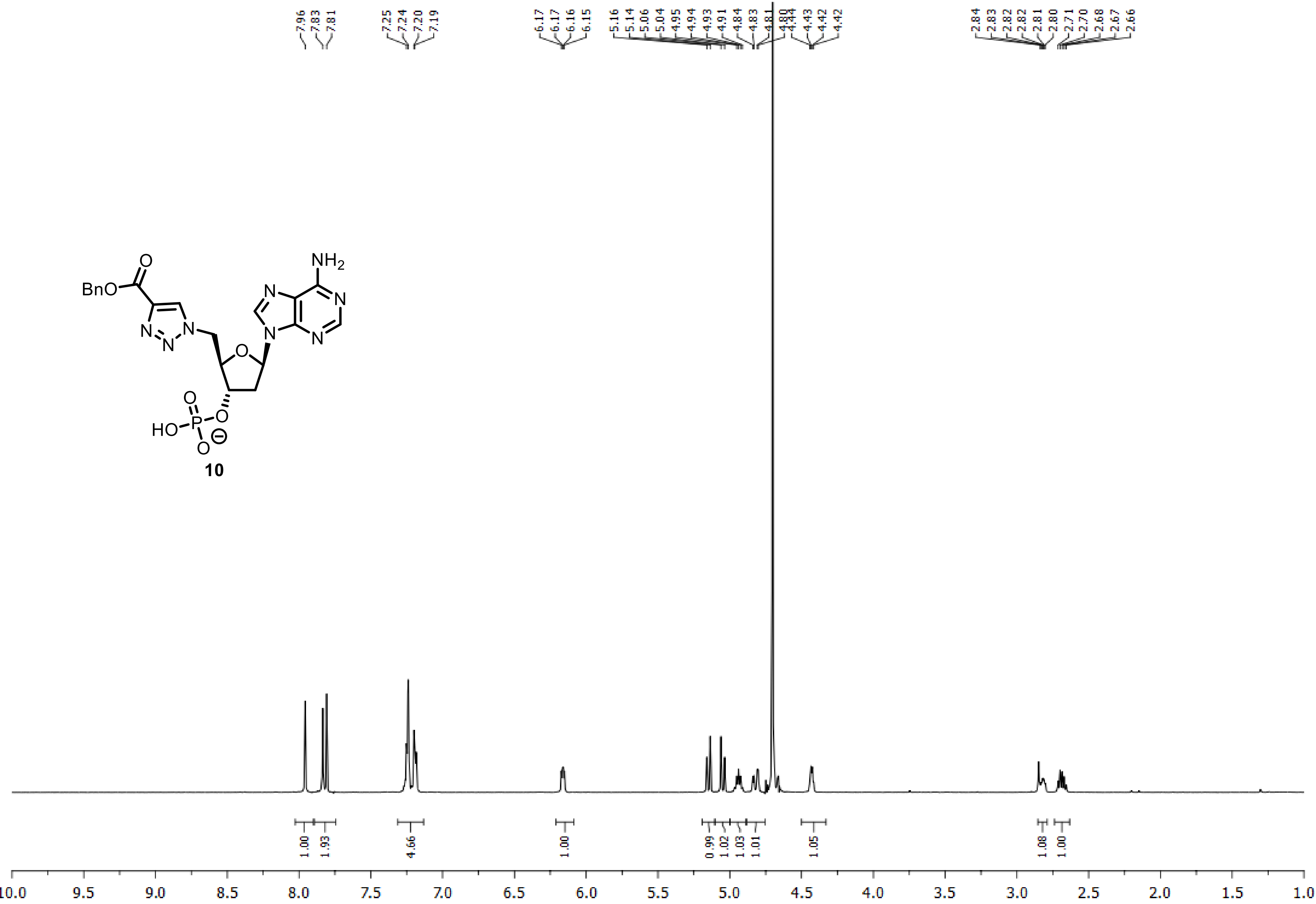
7.96
7.83
7.81
7.25
7.24
7.20
7.19
6.17
6.17
6.16
6.15
5.16
5.14
5.06
5.04
4.95
4.94
4.93
4.91
4.84
4.83
4.81
4.80
4.44
4.43
4.42
4.42
2.84
2.83
2.82
2.82
2.81
2.80
2.71
2.70
2.68
2.67
2.66
BnO
O
N
N
N
O
N
N
N
N
NH2
O
HO
P
O
O
10
1.00
1.93
4.66
1.00
0.99
1.02
1.03
1.01
1.05
1.08
1.00
10.0
9.5
9.0
8.5
8.0
7.5
7.0
6.5
6.0
5.5
5.0
4.5
4.0
3.5
3.0
2.5
2.0
1.5
1.0

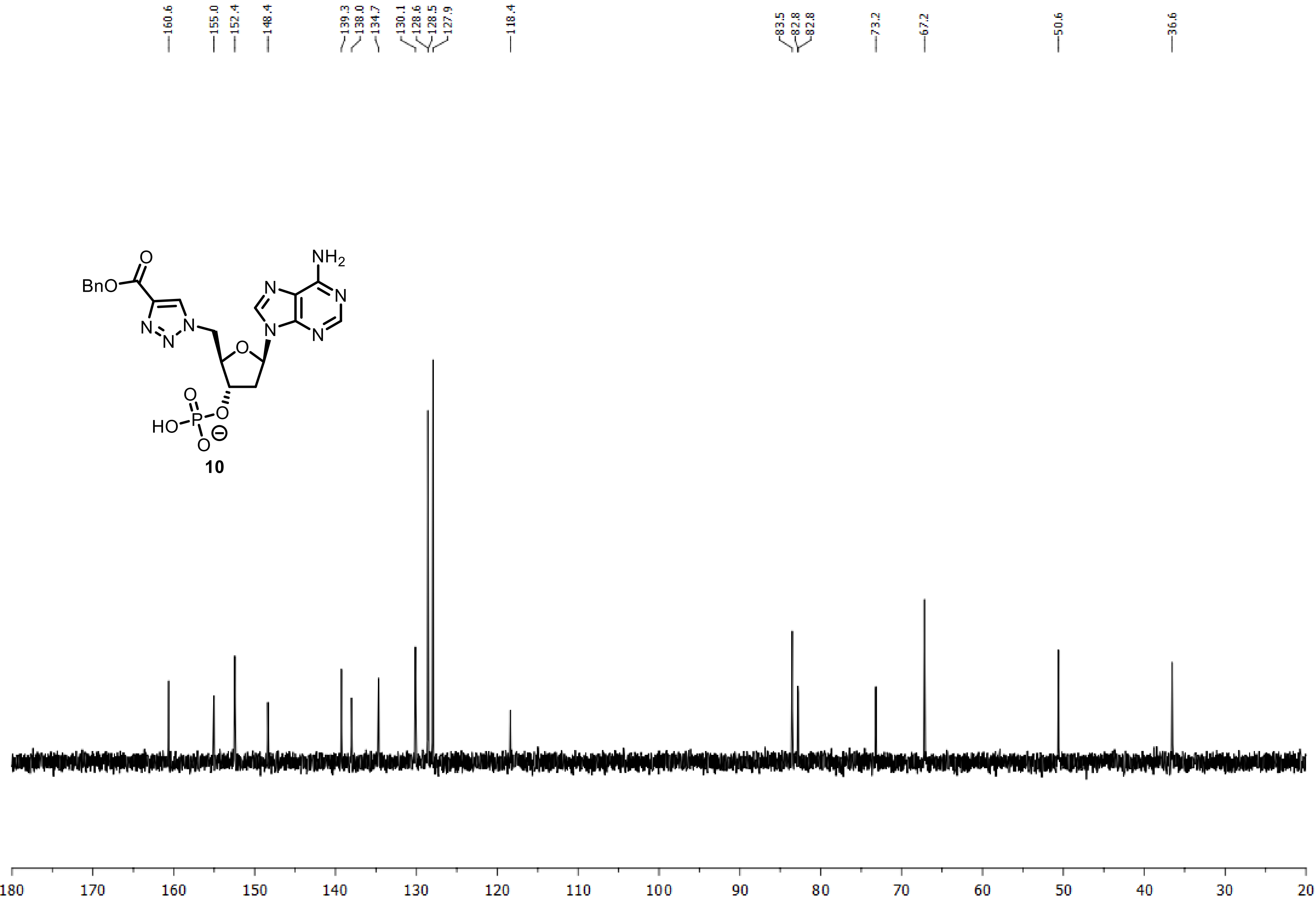
160.6
155.0
152.4
148.4
139.3
138.0
134.7
130.1
128.6
128.5
127.9
118.4
83.5
82.8
82.8
73.2
67.2
50.6
36.6
BnO
O
N
N
N
NH2
N
N
N
N
O
O
HO
P
O
O
10
180
170
160
150
140
130
120
110
100
90
80
70
60
50
40
30
20

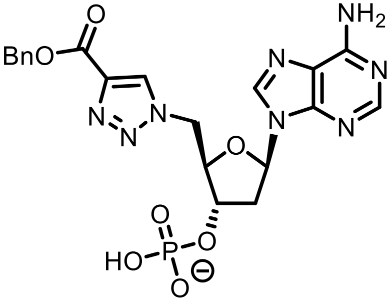

10

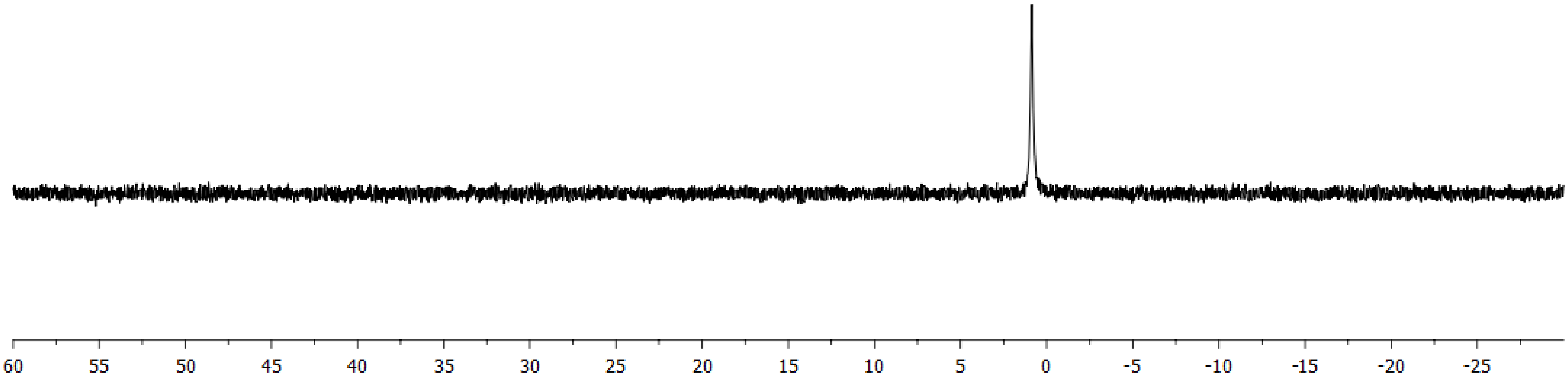

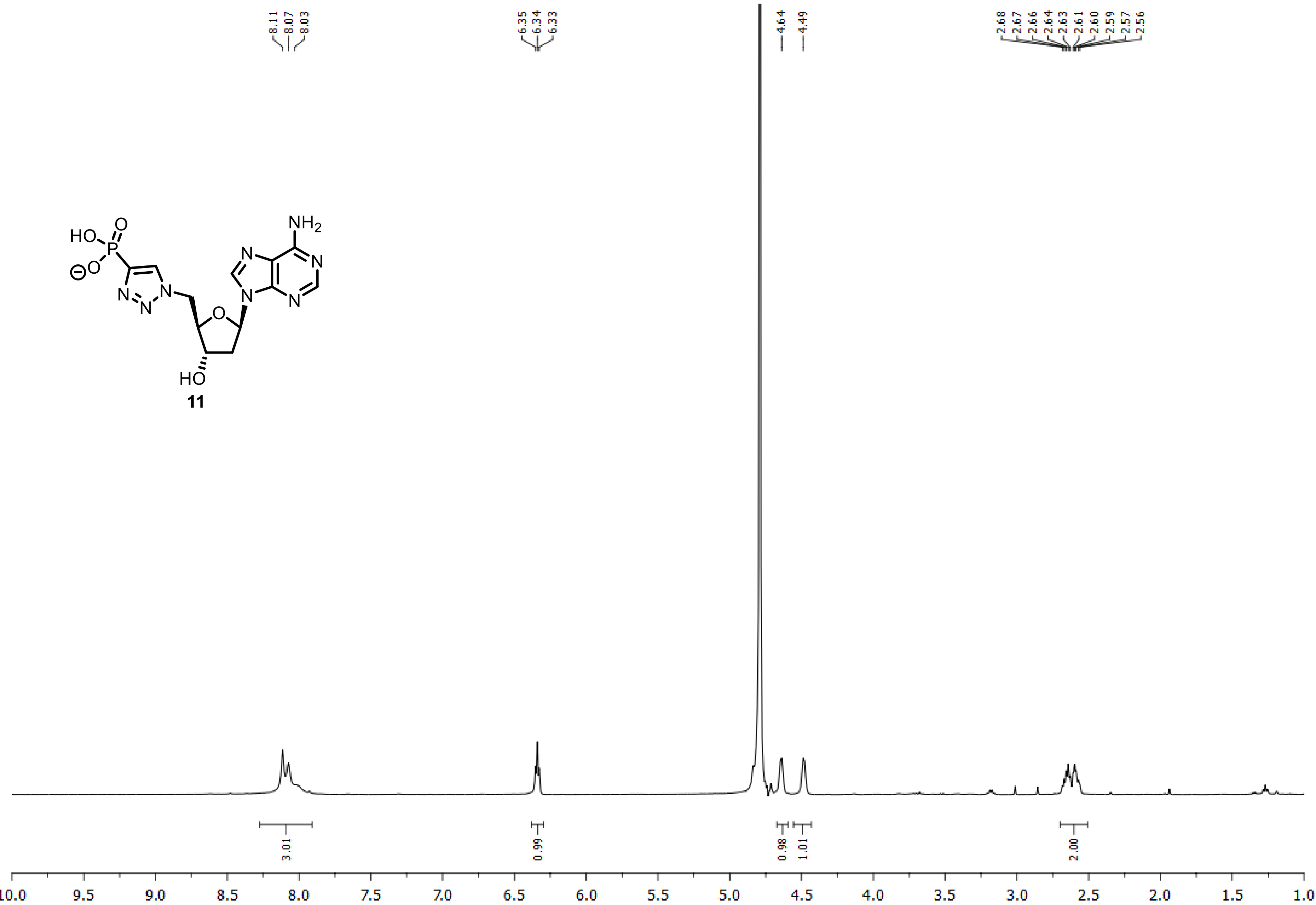
8.11
8.07
8.03
6.35
6.34
6.33
4.64
4.49
2.68
2.67
2.66
2.64
2.63
2.61
2.60
2.59
2.57
2.56
3.01
0.99
0.98
1.01
2.00
10.0
9.5
9.0
8.5
8.0
7.5
7.0
6.5
6.0
5.5
5.0
4.5
4.0
3.5
3.0
2.5
2.0
1.5
1.0
HO
O
P
O
N
N
N
O
HO
NH2
N
N
N
N
11

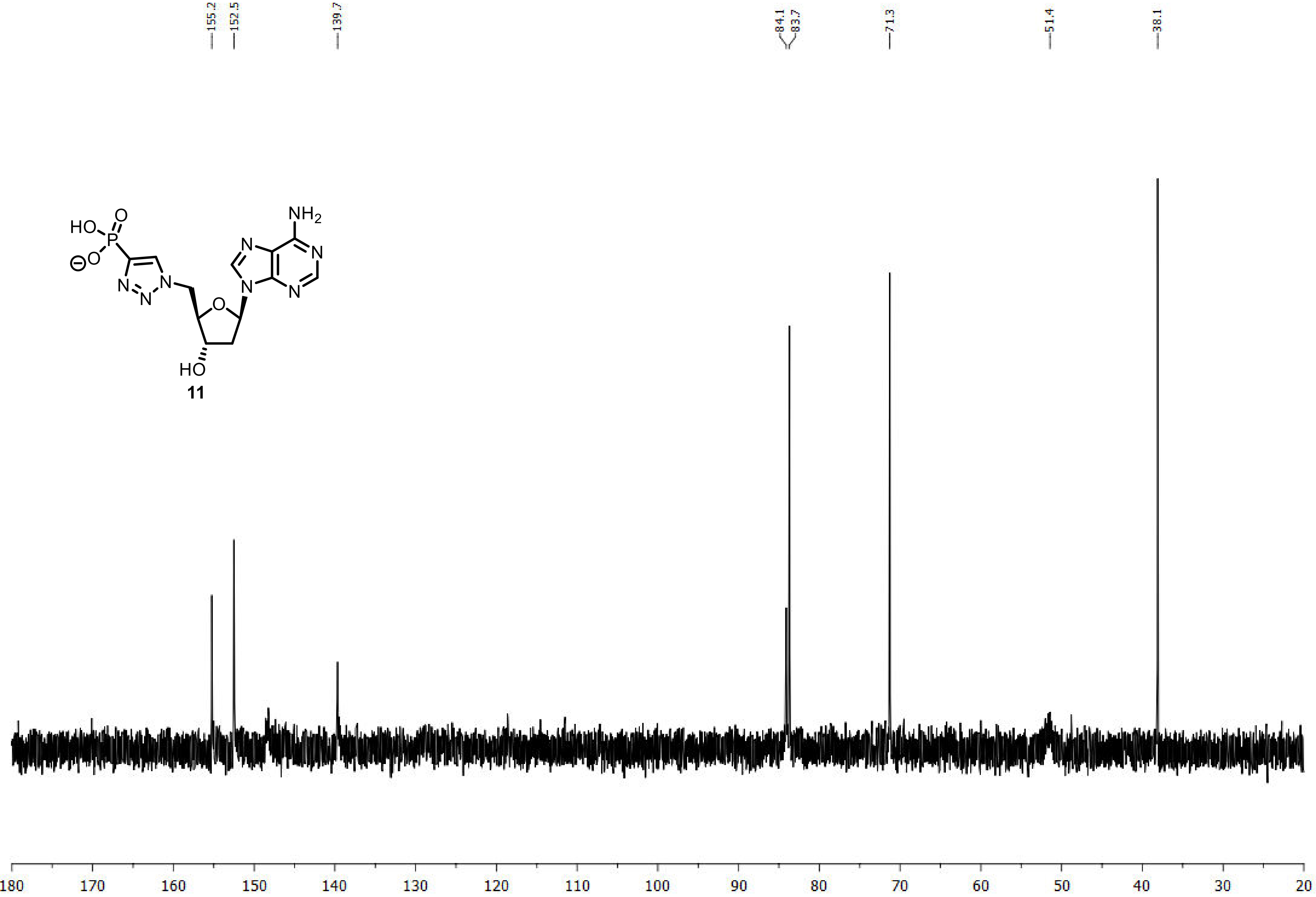
155.2
152.5
139.7
84.1
83.7
71.3
51.4
38.1
HO
O
P
O
N
N
N
NH2
11
180 170 160 150 140 130 120 110 100 90 80 70 60 50 40 30 20

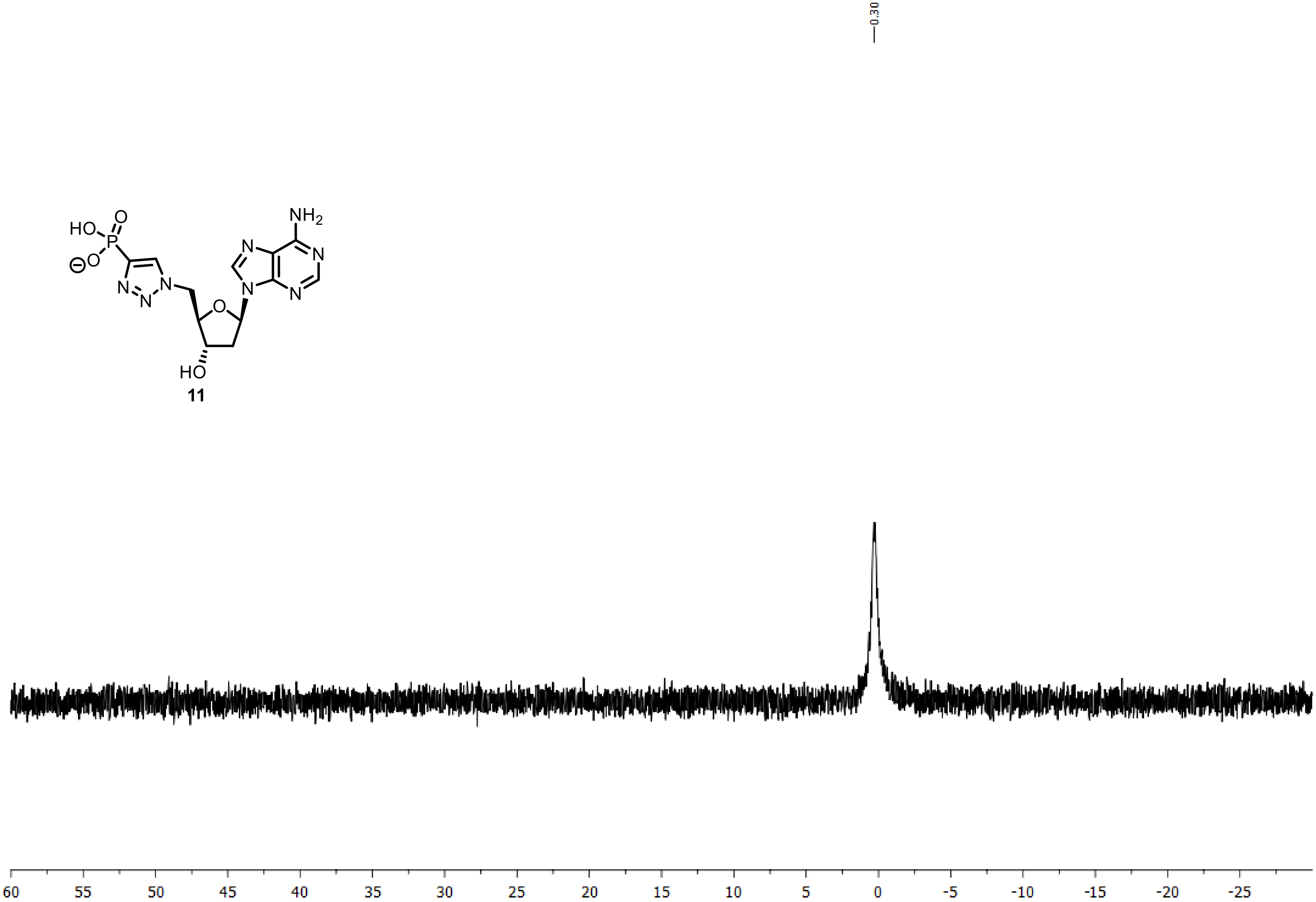

11
0.30
60 55 50 45 40 35 30 25 20 15 10 5 0 -5 -10 -15 -20 -25

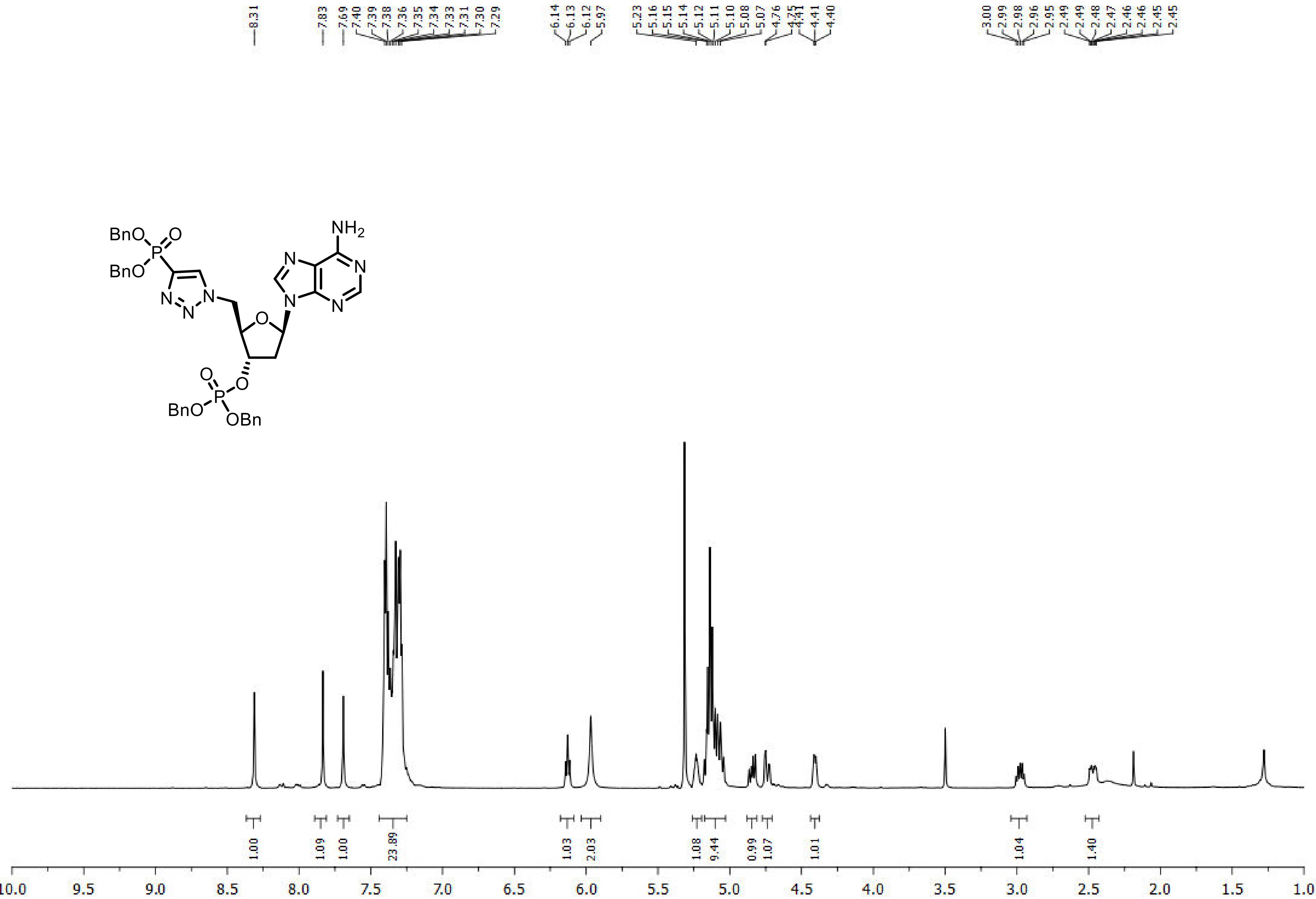

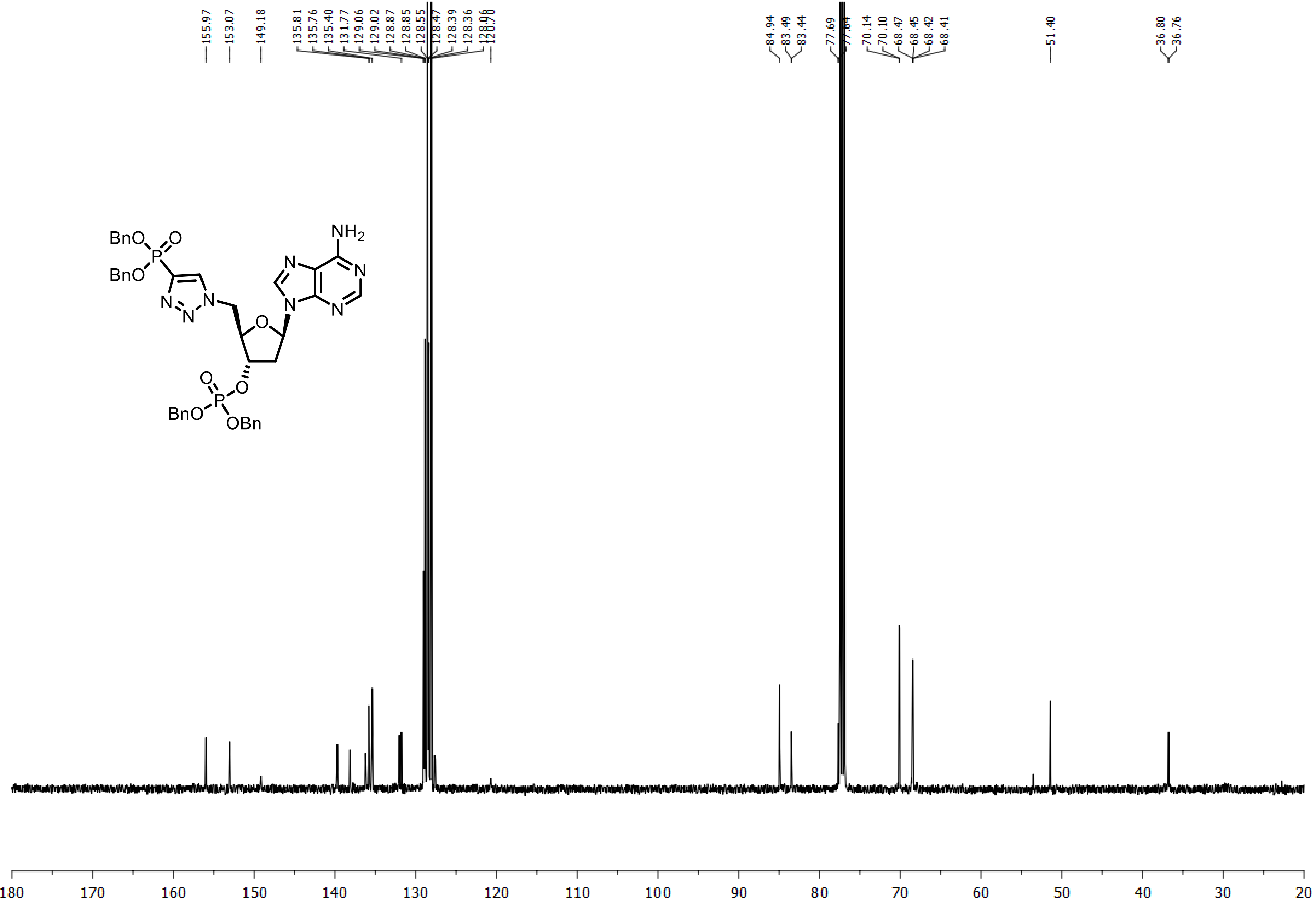
155.97
153.07
149.18
135.81
135.76
135.40
131.77
129.06
129.02
128.87
128.85
128.55
128.47
128.39
128.36
84.94
83.49
83.44
77.69
70.14
70.10
68.47
68.45
68.42
68.41
51.40
36.80
36.76
BnO
O
P
BnO
N
N
N
O
N
N
N
N
NH2
O
P
O
BnO
OBn
180
170
160
150
140
130
120
110
100
90
80
70
60
50
40
30
20

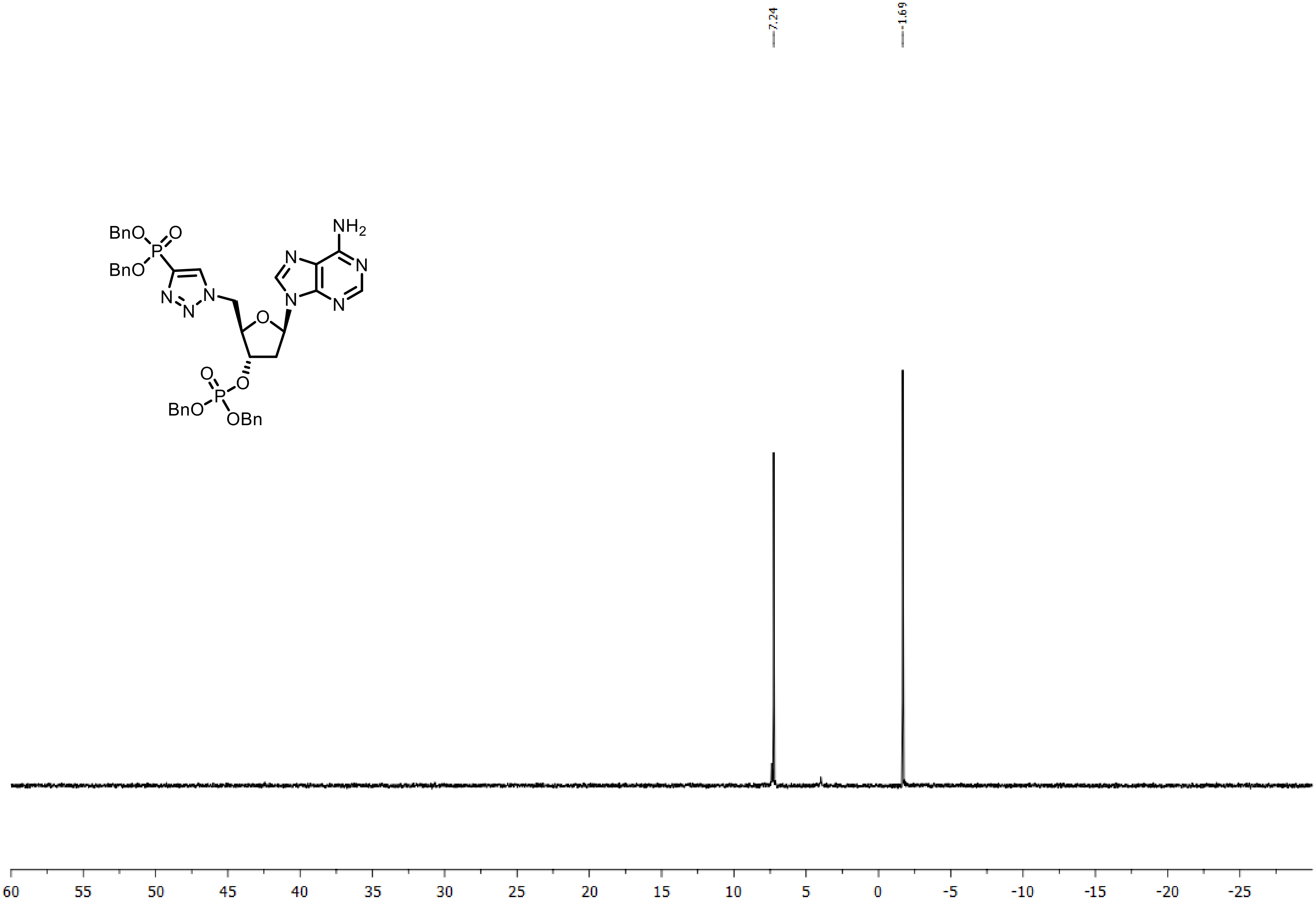
7.24
-1.69
BnO
O
P
BnO
N
N
N
NH2
N
N
N
N
O
O
P
O
BnO
OBn
60 55 50 45 40 35 30 25 20 15 10 5 0 -5 -10 -15 -20 -25

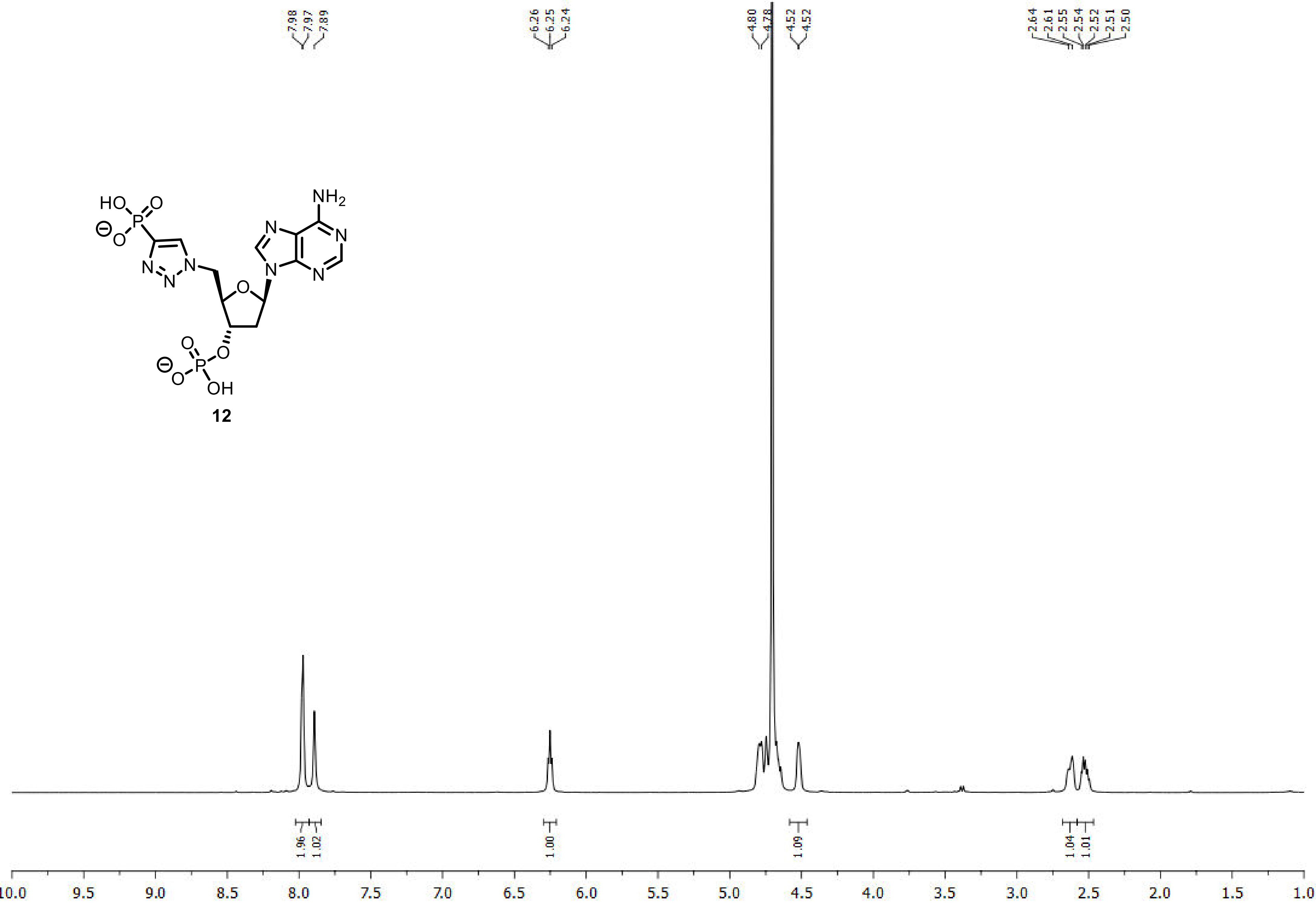
7.98
7.97
7.89
6.26
6.25
6.24
4.80
4.78
4.52
4.52
2.64
2.61
2.55
2.54
2.52
2.51
2.50
HO
O
P
O
NH2
N
N
N
N
N
N
N
N
O
O
P
O
OH
12
1.96
1.02
1.00
1.09
1.04
1.01
10.0
9.5
9.0
8.5
8.0
7.5
7.0
6.5
6.0
5.5
5.0
4.5
4.0
3.5
3.0
2.5
2.0
1.5
1.0

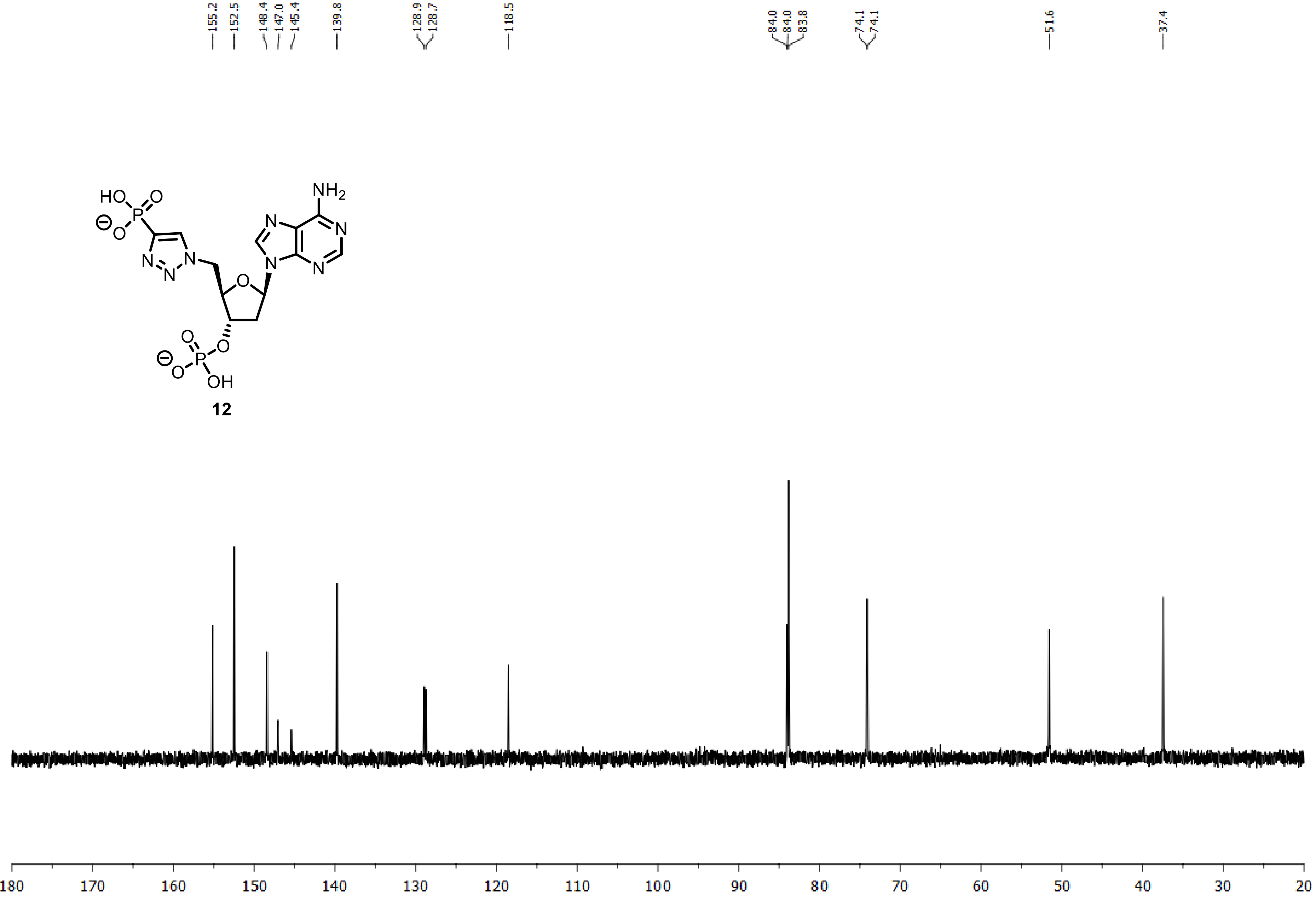
155.2
152.5
148.4
147.0
145.4
139.8
128.9
128.7
118.5
84.0
84.0
83.8
74.1
74.1
51.6
37.4
HO
O
P
O
N
N
N
O
NH2
N
N
N
N
O
P
O
OH
12
180
170
160
150
140
130
120
110
100
90
80
70
60
50
40
30
20

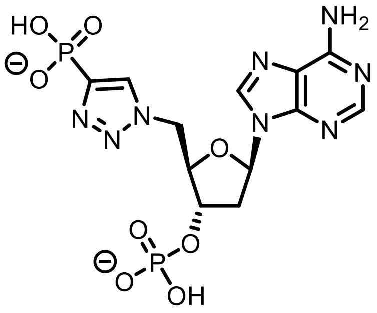
HO
O
P
O
N
N
N
NH2
N
N
N
N
O
O
O
P
O
OH

12

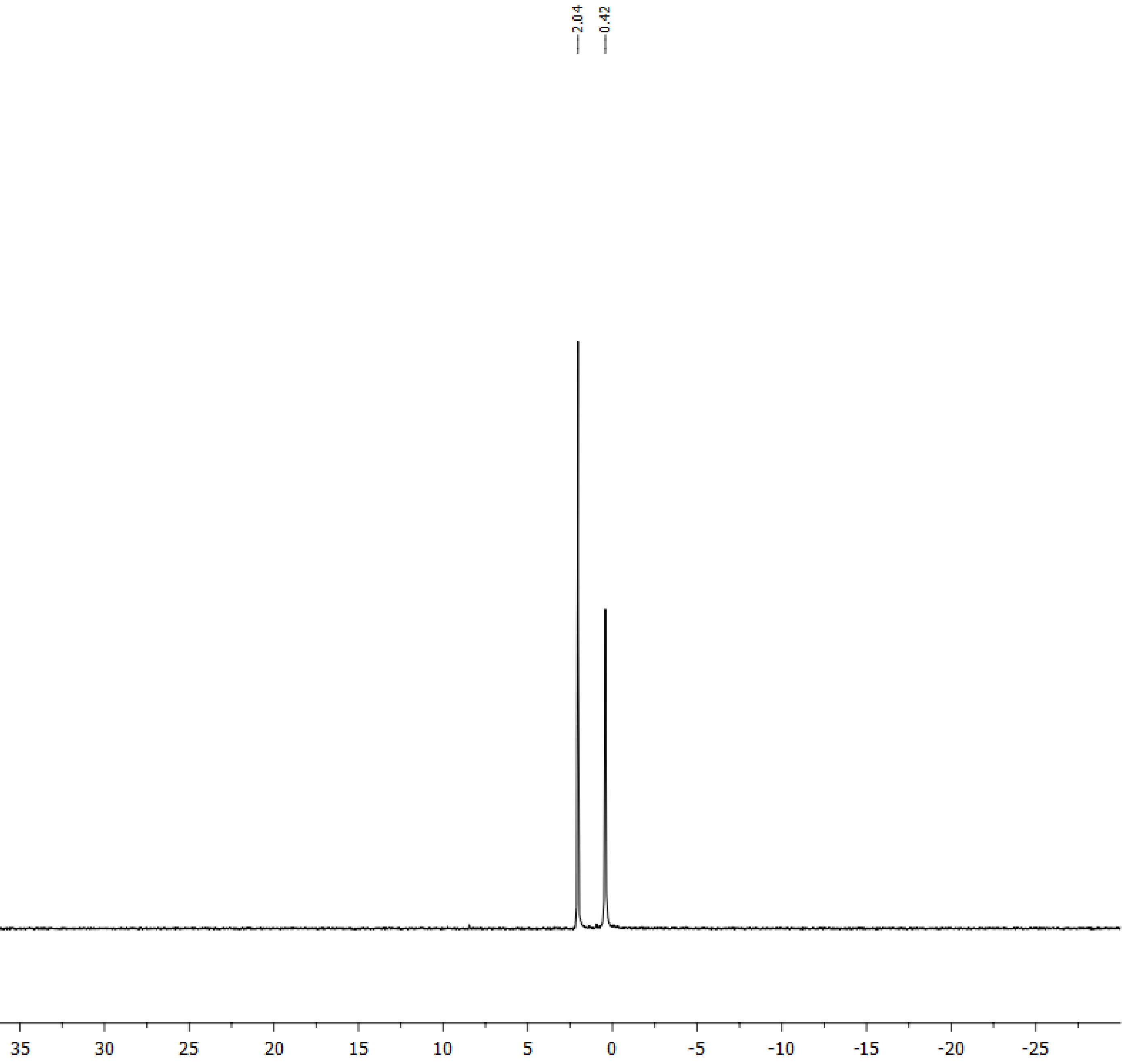
2.04
0.42
60
55
50
45
40
35
30
25
20
15
10
5
0
-5
-10
-15
-20
-25

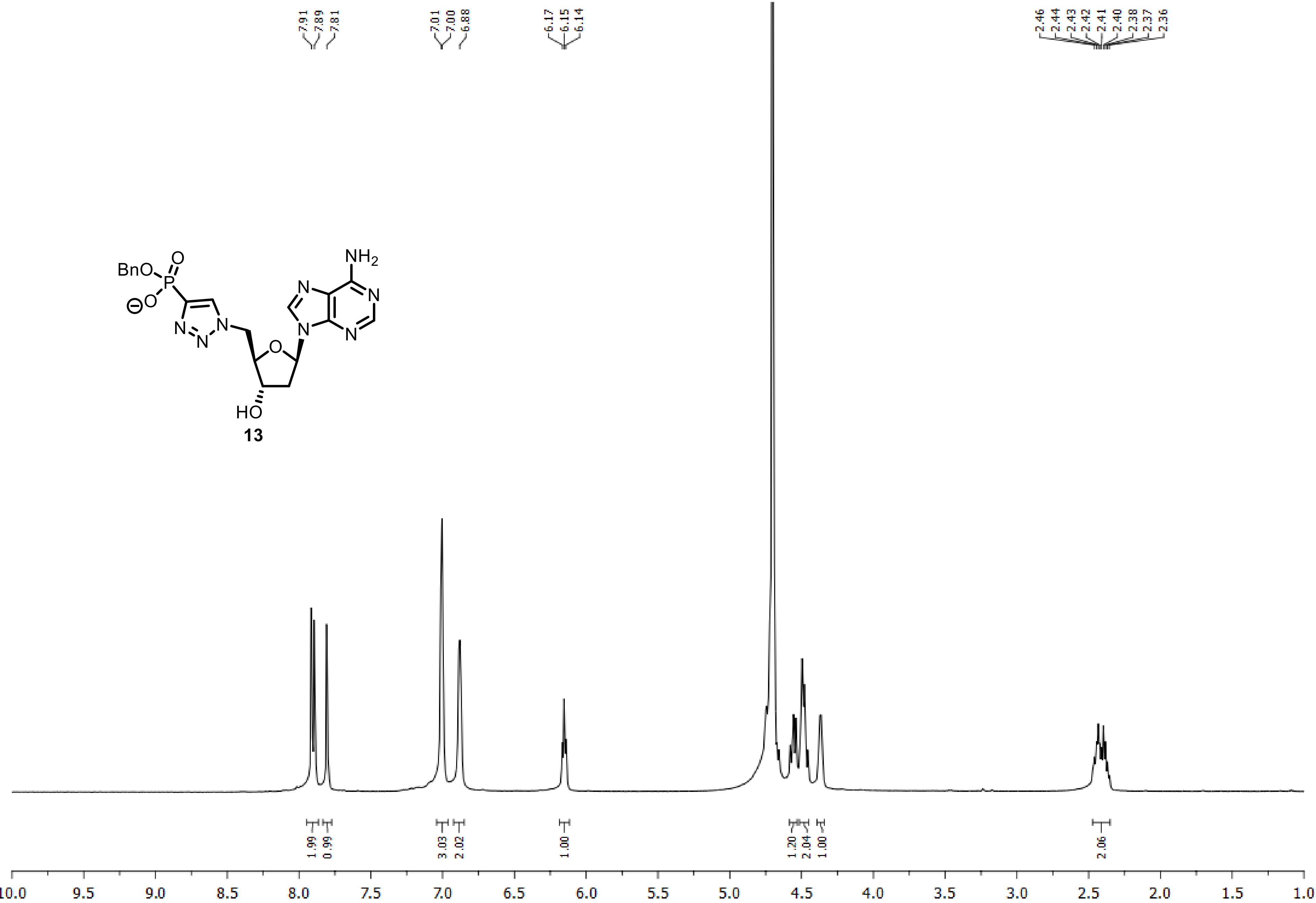
7.91
7.89
7.81
7.01
7.00
6.88
6.17
6.15
6.14
2.46
2.44
2.43
2.42
2.41
2.40
2.38
2.37
2.36
BnO
O
P
O
NH2
N
HO
13
1.99
0.99
3.03
2.02
1.00
1.20
2.04
1.00
2.06
10.0
9.5
9.0
8.5
8.0
7.5
7.0
6.5
6.0
5.5
5.0
4.5
4.0
3.5
3.0
2.5
2.0
1.5
1.0

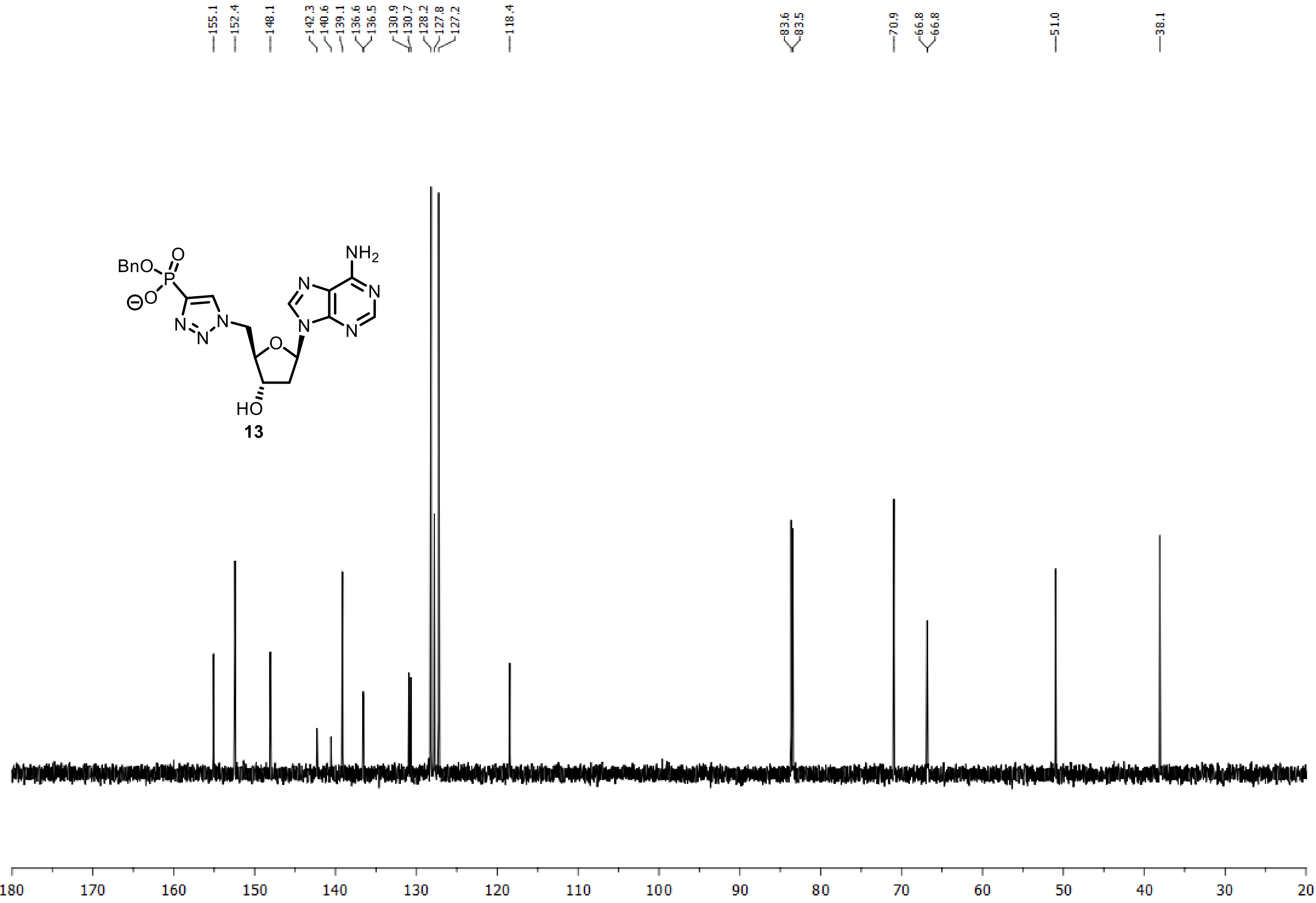
155.1
152.4
148.1
142.3
140.6
139.1
136.6
136.5
130.9
130.7
128.2
127.8
127.2
118.4
83.6
83.5
70.9
66.8
66.8
51.0
38.1
BnO
O
P
O
N
N
N
O
N
N
NH2
N
N
HO
13
180
170
160
150
140
130
120
110
100
90
80
70
60
50
40
30
20

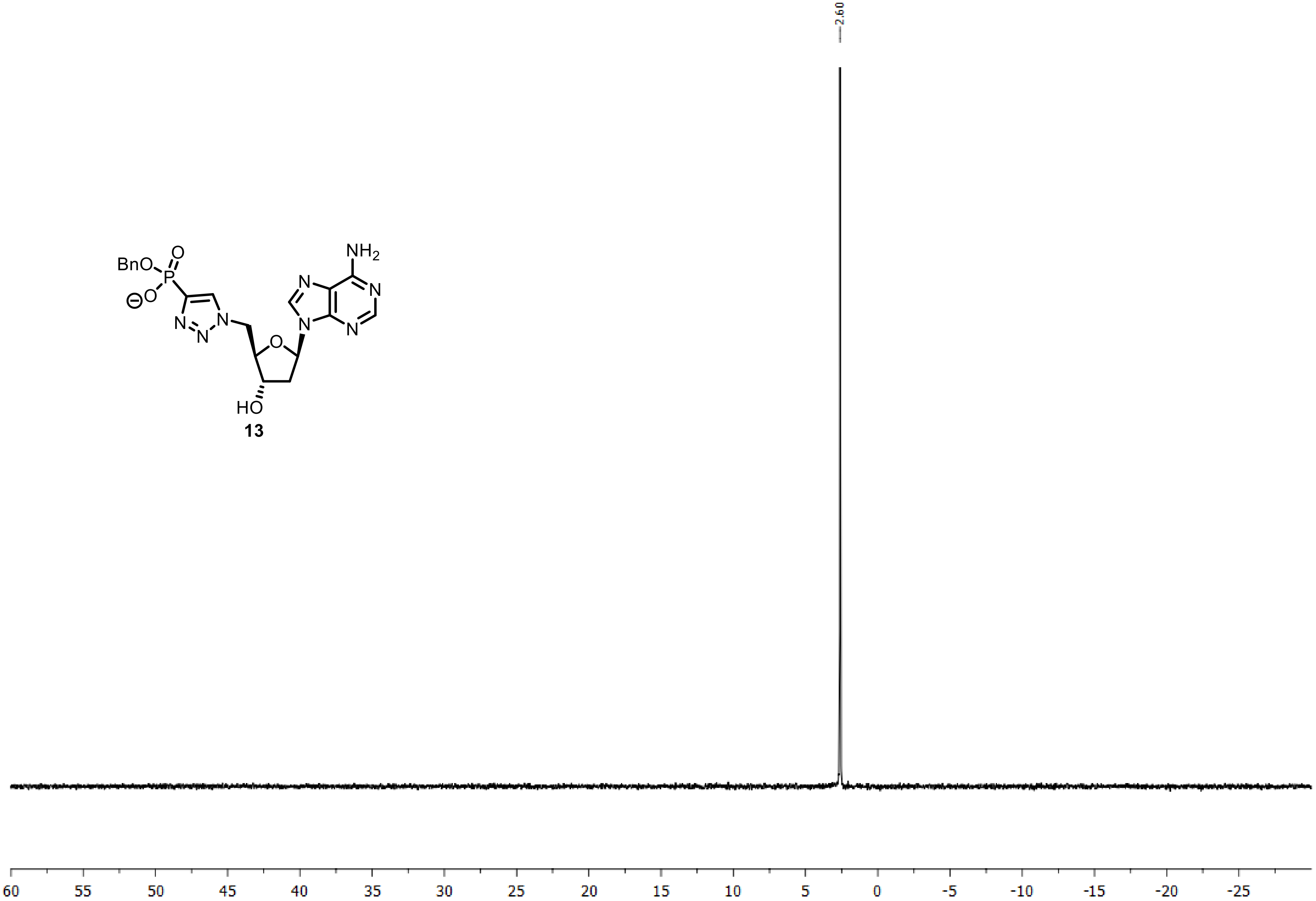
2.60
BnO
O
P
O
NH2
N
N
N
N
N
N
N
N
O
HO
13
60 55 50 45 40 35 30 25 20 15 10 5 0 -5 -10 -15 -20 -25

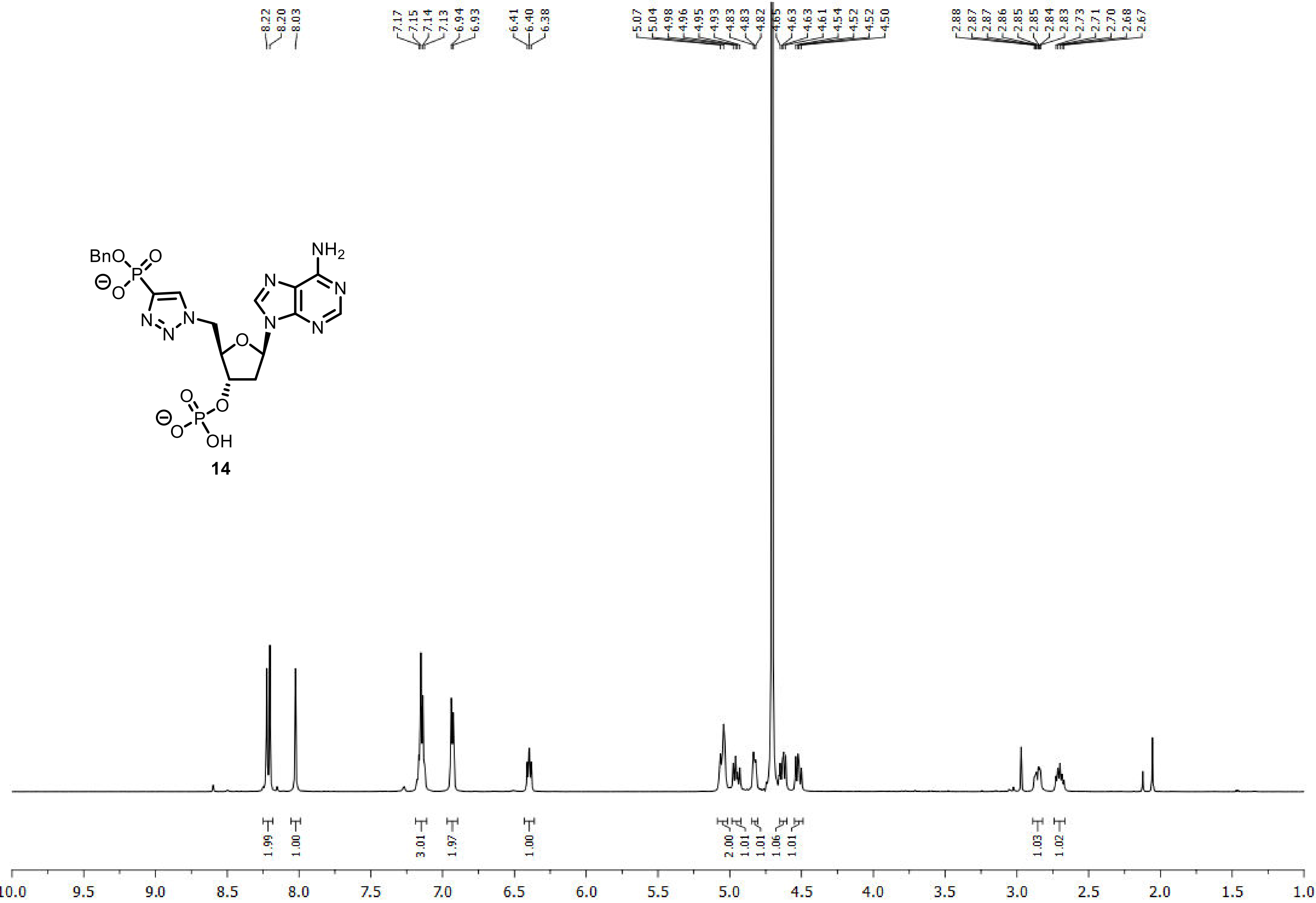
8.22
8.20
8.03
7.17
7.15
7.14
7.13
6.94
6.93
6.41
6.40
6.38
5.07
5.04
4.98
4.96
4.95
4.93
4.83
4.83
4.82
4.65
4.63
4.63
4.61
4.54
4.52
4.52
4.50
2.88
2.87
2.87
2.86
2.85
2.85
2.84
2.83
2.73
2.71
2.70
2.68
2.67
BnO
O
P
O
N
N
N
O
NH2
N
N
N
N
O
O
P
O
OH
14
1.99
1.00
3.01
1.97
1.00
2.00
1.01
1.01
1.06
1.01
1.03
1.02
10.0
9.5
9.0
8.5
8.0
7.5
7.0
6.5
6.0
5.5
5.0
4.5
4.0
3.5
3.0
2.5
2.0
1.5
1.0

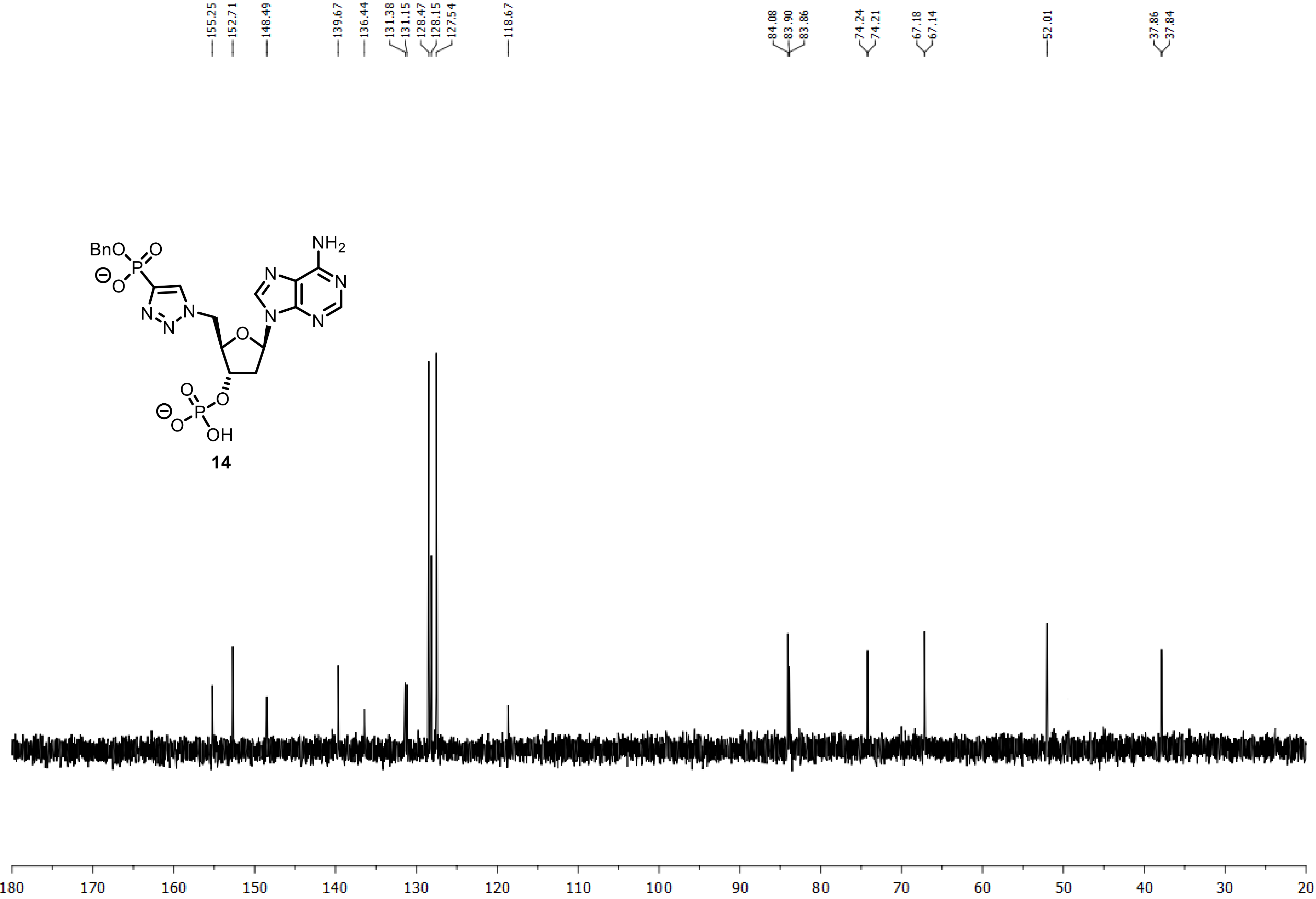
155.25
152.71
148.49
139.67
136.44
131.38
131.15
128.47
128.15
127.54
118.67
84.08
83.90
83.86
74.24
74.21
67.18
67.14
52.01
37.86
37.84
BnO
O
P
O
N
N
N
O
NH2
N
N
N
N
O
O
P
O
OH
14
180 170 160 150 140 130 120 110 100 90 80 70 60 50 40 30 20

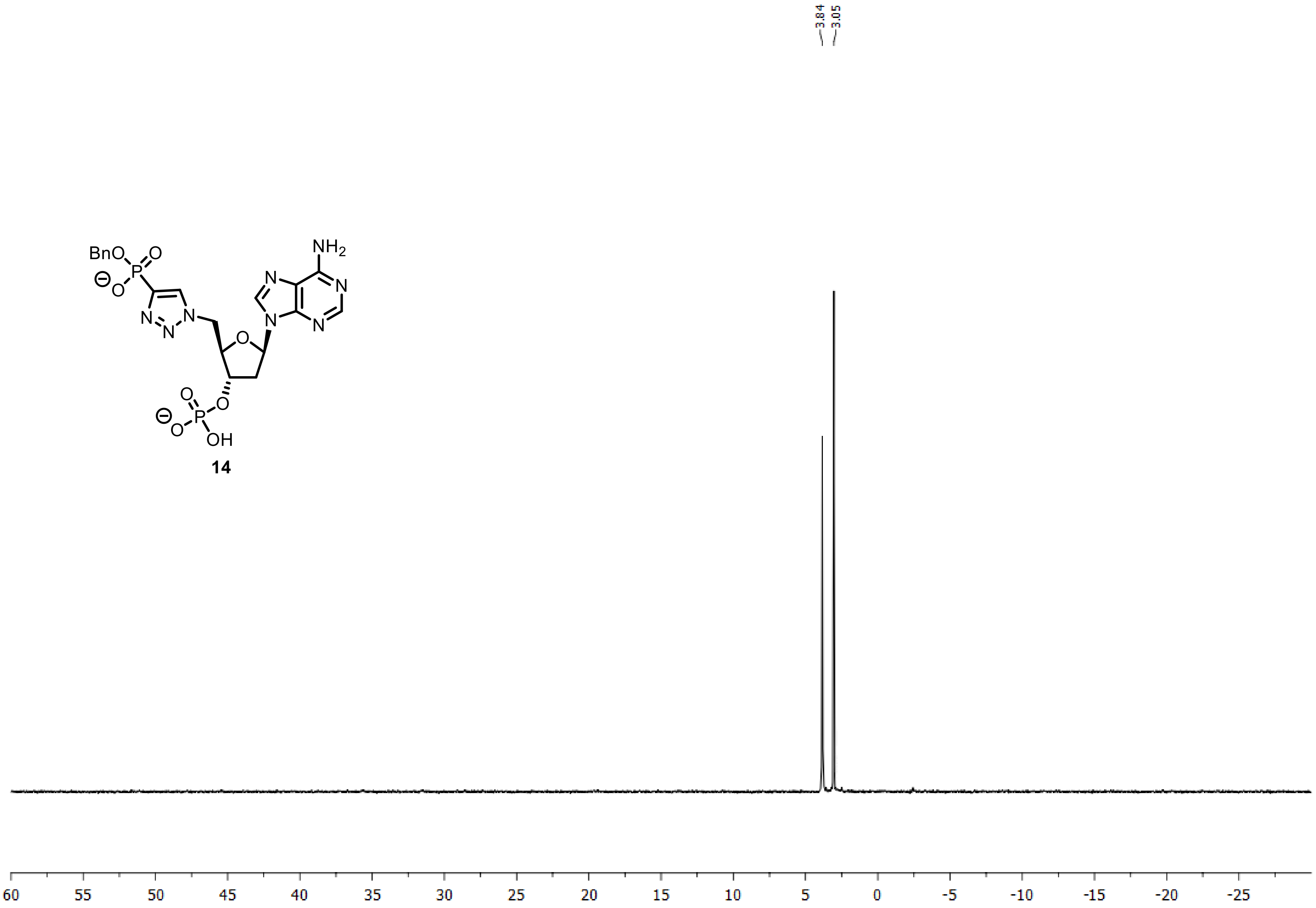

3.84
3.05
BnO
O
P
O
N
N
N
NH2
N
N
N
N
O
O
P
O
O
OH
14
60
55
50
45
40
35
30
25
20
15
10
5
0
-5
-10
-15
-20
-25
